# Genomes of *Strongylocentrotus franciscanus* and *Lytechinus variegatus*: are there any genomic explanations for the two order of magnitude difference in the lifespan of sea urchins?

**DOI:** 10.18632/aging.100889

**Published:** 2016-02-07

**Authors:** Petr V. Sergiev, Artem A. Artemov, Egor B. Prokhortchouk, Olga A. Dontsova, Grigory V. Berezkin

**Affiliations:** ^1^ Lomonosov Moscow State University, Department of Chemistry and A.N. Belozersky Institute of Physico-Chemical Biology, Moscow 119992, Russia; ^2^ Lomonosov Moscow State University, Faculty of Bioengineering and Bioinformatics, Moscow 119992, Russia; ^3^ Center ‘Bioengineering’, Russian Academy of Sciences, Moscow, 117312 Russia and National Research Center, Kurchatov Institute, Moscow 123098 Russia; ^4^ ESN Group, 123100, Moscow, Russia

**Keywords:** longevity, sea urchin, genomics, genome sequencing

## Abstract

Sea urchins are marine invertebrates of extreme diversity of life span. Red sea urchin *S. franciscanus* is among the longest living creatures of the Ocean. Its lifetime is estimated to exceed a century, while the green sea urchin *L. variegatus* hardly survives more than four years. We sequenced and compared the genomes of these animals aiming at determination of the genetic basis of their longevity difference. List of genes related to the longevity of other animal species was created and used for homology search among the genomic data obtained in this study. Aminoacid sequences of longevity related proteins of *S. franciscanus* and *L. variegatus* as well as from a set of model species, were aligned and grouped on the basis of the species lifespan. Aminoacid residues specific for a longevity group were identified. Proteins containing aminoacids whose identity correlated with the lifespan were clustered on the basis of their function.

## INTRODUCTION

Sea urchins belong to deuterostomes and as such are closer relatives to vertebrates than other invertebrate taxons such as insects and nematodes. Sea urchins are used as a convenient model for developmental biology. Fertilization of sea urchin eggs takes place in sea water and is followed by rapid development of a pluteus, a free floating larva possessing bilateral symmetry. Radially symmetrical adult body develops from the rudiment asymmetrically placed within the larva. Adult sea urchins possess calcite skeleton and live on seabed from tidal zone to the several kilometers deep.

Apart from complex development scheme, sea urchins attracted attention due to extreme longevity of some of their species. Red sea urchin, *S. franciscanus*, populating cold waters of Pacific coast of North America, was demonstrated to survive over a century [1]. Although *S. franciscanus* could not be cultivated in the lab for a century for direct observation, deposition pattern of radioactive carbon released to the Pacific upon nuclear tests [2] and skeleton growth rate studies using tetracycline labeling [1] allowed red sea urchin to climb the pedestal of the most long-lived marine animals [3]. At the same time, green sea urchin, *L. variegatus*, populating warm Caribbean sea hardly survive over four years [4]. Although direct difference in the senescence rates between red and green sea urchins is hard to demonstrate directly on the sole basis of field studies, these two related species might be the a convenient pair for comparative genetics of longevity.

In this report we aimed to obtain draft genome assemblies of *S. franciscanus* and *L. variegatus* and compare the sequence of their proteins related to longevity with longevity related proteins of other species. We used mapping of our sequencing data onto previously published complete genomic sequence of a purple sea urchin, *Strongylocentrotus purpuratus* [5].

## RESULTS

### Short read sequencing and assembly of *S. franciscanus* and *L. variegatus* genomes and mapping of protein coding genes of *S. franciscanus* and *L. variegatus*

**Figure 1:**
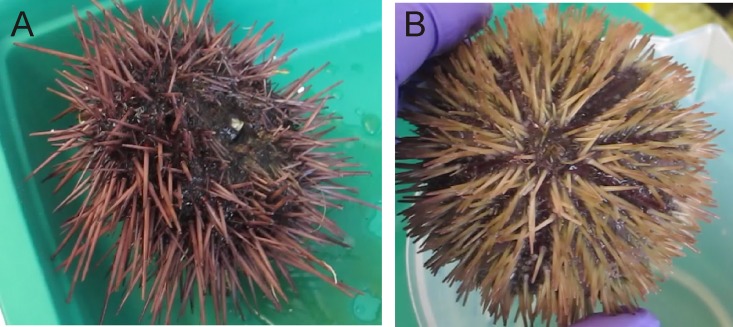
Sea urchins used in this study. (**A**) *S. franciscanus* (**B**) *L. variegatus*.

Gonads of female *S. franciscanus* (Figure 1A) and *L. variegatus* (Figure 1B) sea urchins were used for the preparation of total genomic DNA followed by massively parallel sequencing on Illumina HiSeq. Totally 63.4·10^9^ nucleotides were sequenced for *S. franciscanus* and 62.3·10^9^ nucleotides for *L. variegatus*. Given approximate genome sizes of 0.76 and 0.84·10^9^ nucleotides [6] these corresponds to 83X and 74X coverage. Genomes assembly resulted in 4,426,585 contigs with N50 size 506 nucleotides for *S. franciscanus* 5,107,105 contigs with N50 size 708 nucleotides for *L. variegatus*. Assembled genome size achieved was 0.6·10^9^ nucleotides and 1.3·10^9^ nucleotides for *S. franciscanus* and *L. variegatus* correspondingly, which approximately match the expected genome sizes of sea urchins. Contigs obtained for red and green sea urchin genomes were mapped onto the genome of *S. purpuratus* [5].

To construct multiple alignments of protein sequences in the studied sea urchins and the reference long- and short-lived organisms (*H. glaber, M. brandtii, M. musculus, S. purpuratus*), we used blast software to search for homologs of the proteins of interest. For every protein of interest, we combined the sequences of protein pseudo-scaffolds generated from *S. franciscanus* and *L. variegatus* assemblies, the reference protein sequence from *S. purpuratus* genome and the homologs from the reference long- and short-lived organisms (*H. glaber, M. brandtii, M. musculus*).

We next asked if the alignments contained amino-acid substitutions discriminating long- and short-lived organisms. In the first approach, we were looking for the positions containing one amino acid in all long-lived organisms and some other amino acid in all short-lived organisms. The positions were automatically detected and then manually validated: only the substitutions in the regions with good alignment of the neighboring positions were taken into account. This approach happened to be too rigid and tended to detect the substitutions to a similar amino acid (e.g. S-T or V-I). In the second approach we grouped the amino acids to similarity groups (i.e. C, STPAG, NDEQ, HRK, MILV, FYW) and searched for the positions having amino acids from different groups in long- and short-living organisms.

Genome assemblies of *S. franciscanus* and *L. variegatus* were used to map protein coding genes similar to those in the published *S. purpuratus* genome [5]. For gene prediction Gmap program was used [9].

## DISCUSSION

Phenotype is determined by a genotype. In this paradigm all explanations of the longevity difference between species and to certain extent even between individuals could be deduced from their genomes. In a golden dream, one could deduce a limited set of genetic variations which might be introduced to a certain genome to extend the lifespan of a creature. However, what sort of differences are we looking for? Even genomes of individuals who belong to the same species differ too much to allow easy determination of those differences that have an impact on longevity. E.g. genome-wide association studies, carried on different cohorts rarely came to the same candidate genes whose specific allelic variants are beneficial for longevity, ApoE and Foxo3A being only reproducible examples [10]. At the same time a number of single mutations or a small set of mutations may increase an average lifespan of a model organism by a substantial proportion, sometimes twice or even more. Mutations, affecting insulin related receptor/forkhead transcription factor pathway in *C. elegans* allowed to extend lifetime of a worm by a factor of five [11]. This finding correlates well with the observation that caloric restriction is one of the key environmental factors influencing longevity [12]. Do we have a hope that genetic difference between the related species that have drastically different lifespan could explain the longevity? If senescence is a consequence of entire developmental program of the individual [13] leading to the accumulation of undiluted poisonous by-products of metabolism [14] then genome as a whole encode the longevity. However, if senescence is a program beneficial for the survival of a population at the expense of individual, as was originally proposed by August Weizmann [15] and later rephrased by other scientists [16] than a limited set of genes might be found to determine longevity. In both cases, complete genomes of related species could be used to determine the basis of longevity.

A number of comparative studies were previously done to decipher genetic backgrounds of exceptional longevity. Naked mole rat, *Heterocephalus glaber*, has a lifespan of other 30 years and shows no increase in mortality with its age [17]. Sequencing of its genome allowed a direct comparison with the genome of related short living rodent, mouse [14]. Later on, the complete genomic sequence of exceptionally long living bat, *Myotis brandtii*, was deciphered [18]. Apart from genes obviously related to the adaptation to ecological niche, specific genetic variants were revealed for telomere maintenance and DNA integrity maintenance systems of naked mole rat [19]. Genes encoding a subset of insulin related receptor/forkhead transcription factor pathway components were found to differ *Myotis brandtii* from other species [14].

Complete genomes of a number of exceptionally long living species, including human [20] become available as well as genomic sequences of related short living species, which could be used for comparison. We decided to use genomic sequence of *S. franciscanus* and *L. variegatus* determined in our study to analyze variations distinguishing the species on the basis of their longevity. We selected a set of genes previously known to affect longevity (Table 1) of the model species and made alignments of their homologues from the set of organisms. We included human (*Homo sapiens*), naked mole rat (*Heterocephalus glaber*), bat (*Myotis brandtii*) and red sea urchin (*S. franciscanus*) into our set as long living organisms, while mouse (*Mus musculus*) and green sea urchin (*L. variegatus*) populated a list of short living species. Protein sequences of purple sea urchin *S. purpuratus* were also included into alignments as a reference. If a protein originally described to alter longevity was initially described in the species other than listed, its sequence is also included to the alignment. For all listed species the protein sequences most closely related to the query was taken for the alignment. It should be stated that some parts of the protein sequences might be misidentified due to the ambiguity in identification of juxtaposed contigs and that some marginally similar proteins could actually perform non-ortologues function.

**Table 1:** Proteins that could be related to longevity according to the literature data

		Residues that co-vary with longevity						Commentaries
Category	Protein	*H. sapiens*	*H. glaber*	*M. brandtii*	*S. franciscanus*	*L. variegatus*	*M. musculus*	
Mitochondrial proteins encoded in mitochondria	ND1							
	ND2	T156 W239	S I		A I	V T	M A	
	COX1							
	ND4L							
	COX2							
	ATP8							
	ATP6							
	COX3							
	ND3	L12 L15	S S		A T	V I	L L	
	ND4							
	ND5	I283	T		T	I	L	
	CYTB							
Mitochondrial proteins encoded in nucleus and could be related to longevity	CYTC							
	COX4							
	COX5B							COX5 mutation in *P. anserine* increase lifespan 10-times [31]
	COX6A							
	COX6B							
	COX6C							
	COX7C							
	p66Shc							Increases reactive oxygen species production [14]
Proteins involved in detoxification of reactive oxygen species	MnSOD							Overexpression extends lifespan of fly [35]
	CuZnSOD							
	CAT							
	Prdx							
	GPx							
Lipid transport proteins	ApoB	K720 I3433	K I	K I	K I	N P	E A	ApoE allelic variant is associated with increased lifespan in humans [39]. Since ApoE homologues were not identified in sea urchins, all apolipoproteins which are present in sea urchin were analyzed.
	ApoA							
	ApoH	R203 N253	R N	R N	K E	I S	L T	
	ApoD	K75 I138	K I	K V	R L	Q F	E F	
	ApoO							
	LDLR							
	VLDLR							
	CETP							
Proteins involved in amyloidogenesis	APP							Mutations cause predisposition to Alzheimer disease in humans [22].
	PSEN1	R42	R	R	R	Q		
	BACE1							
Telomere maintenance	TERT	G252 R342 T491 P702 D975	G R T P D		G K S P D	R Q I N S	R N L Q S	
	POT1	I198	I	V	V	S	T	
	TEP1							
Insulin/IGF1 signaling pathway	INSR							
	IGF1R							
	IRS1							
	PTEN							
	PI3K	H295 (γ isophorm)S275 (αisophorm)	H	H	K	Q	Q	
	PDK1							
	AKT1							
	SGK							
	FOXO1							
	FOXO3							
	FOXO4							
	MTOR							
	SIRT1							
	SIRT2							
	YWHAG							
Other proteins associated with longevity	clk-1	H L	H I	H L	K I	E Y	N F	Q117 F132 (*C. elegans*)
	daf-9							
	Mth							
	Indy	P S	P S	P S	S G	N N	Q N	E61 V193 (*D. melanogaster*)
	EXO1							

We used the created alignments (see Supplementary material) for identification of the aminoacid positions co-varied with longevity. Although it might be naïve to expect that single positions within a limited set of proteins could determine longevity, we decided to perform such kind of analysis to suggest hypotheses for further studies.

### Amyloid protein biogenesis

Alzheimer disease is one of the widely recognized factors limiting human longevity. In a brain of Alzheimer disease patients one can find an accumulation of beta-amyloid protein plaques [21] which are formed from a peptide excised from APP protein by β (BACE1) and γ-secretases (PSEN1). A number of mutations in APP and PSEN1 genes were identified as a cause of hereditary form of Alzheimer disease [22]. Although sea urchins have rather primitive nervous system we decided to search for APP, PSEN1 and BACE1 homologs in *S. franciscanus* and *L. variegatus* genomes. Only short patches of APP homolog in sea urchins display some similarity with mammalian APP preventing direct comparison of the β-amyloid part of the protein. However, both β- and γ-secretases could readily be identified in all sea urchins under study. Only one aminoacid residue was found to correlate with longevity in PSEN1 protein. Aminoacid corresponding to Arg42 of human PSEN1 is represented by arginine in other long living species, is substituted by glutamine in short living mice and green sea urchins. Position of this aminoacid residue is located in the area close to the region 79-291, carrying a number of mutation sites predisposing an individual for Alzheimer disease [22].

### Mitochondrial proteins and proteins involved in detoxification of reactive oxygen species

One of the most recognized theories of aging is a theory of oxidative damage [23]. Although originally proposed variant of the theory underwent several rounds of modification [16, 24], the main postulate of negative influence of reactive oxygen species on longevity [25] could still face some exceptions [26]. Positive role of reactive oxygen species in regulatory networks may be more beneficial than potential damage imposed by those reactants [27]. However controversial might be the issue of oxidative damage for senescence we included a set of relevant proteins into our analysis. Among the sequences of proteins encoded in the mitochondrial genome, ND2 subunit of NADH dehydrogenase possesses two aminoacid residues whose identity co-varies with longevity. Aminoacid 156 (human numbering) is represented by small aminoacid in long living species, threonine in human, alanine in red sea urchin, serine in naked mole rat. In contrast, short living mouse and green sea urchin contains large hydrophobic methionine and valine at this position. Opposite specificity is attributed to the aminoacids at position 239 (human numbering). Human ND2 contains tryptophan at position 239, naked mole rat and red sea urchin contains isoleucine, while mice and green sea urchin have small alanine and threonine at this position. Substitutions of proximal aminoacids 150 and 259 in human cause genetically inherited Leber optic neuropathy [28, 29].

In ND3 subunit of NADH dehydrogenase position 12 is occupied by small aminoacids serine and alanine in ND3 of naked mole rat and red sea urchin, while mouse and green sea urchin possesses large hydrophobic leucine and valine at this position. It should be noted that human ND3 also has a leucine at position 12, while human belongs to the species that have an increased lifespan. Similar rules act for the aminoacids at position 15 of ND3. Naked mole rat and red sea urchin contains serine and threonine in this place, while human, mouse and green sea urchins contains leucine or isoleucine. Mutation Thr114Ala in human ND3 was found to be associated with reduced risk of Parkinson disease development [30]. While mouse also has threonine at the position 114, long living naked mole rat possesses aspartic acid and red sea urchins have alanine, similar to people with reduced predisposition to Parkinson disease. In a position 283 of ND5 one can find threonine in naked mole rat and red sea urchin, while human, green sea urchin and mouse contain bulky isoleucine and leucine in this place. Mutation in the cytochrome c oxidase subunit of fungi *Podospora anserine* resulted in a 10-times increase in the lifespan [31]. We checked for the aminoacid positions that correlate with the lifespan in our species set and could not identify any. However, we noted that COX6B mutation Arg20His which was found in a family with 5-time reduced cytochrome oxidase activity [32] could also be found in the long living bat, *M. brandtii*. Protein p66Shc was demonstrated to increase reactive oxygen species production in mitochondria [33, 34]. Analysis of p66Shc sequences in the set of long and short living organisms revealed only differences explained by phylogenetic relations, and not by longevity.

A number of proteins aim in detoxification of reactive oxygen species. Among them, superoxide dismutases MnSOD, localized in mitochondria and CuZnSOD residing in the cytoplasm. Ectopic expression of CuZnSOD in fly allowed to extend its lifespan [35]. Mutation Ala16Val in human MnSOD leads to a 30-40% reduction of its activity resulting in cardiomyopathy and nephropathy [36-38]. Long living naked mole rat, similar to human, contains Ala16 residue, while short living mouse contains Val16, similar to humans, predisposed to the pathology. Unfortunately, we were unable to identify the sequence corresponding to this region of MnSOD in sea urchin genomes. We found no substitutions correlated with the lifespan in CuZnSOD, catalase, peroxiredoxin and glutathione peroxidase.

### Lipoprotein metabolism

Arthrosclerosis is an important human pathology with age dependent onset and high impact of human longevity. Accumulation of lipid plaques on the walls of blood vessels accompanied by local inflammation increases the risk of heart attack and stroke. Although lipid metabolism of sea urchins might be substantially different from those in mammals, sea urchins possess apolipoproteins which are used as lipid carriers. In humans, the main scaffold for lipid transport as low density lipoprotein particles is ApoB protein. To best of our knowledge none of the mutations in ApoB encoding gene are related to longevity. However, allelic variant of another lipoprotein scaffold protein, ApoE, was recognized as a marker of human longevity [39]. We included several apolipoproteins into our analysis. In the sequence of ApoB protein, aminoacid, corresponding to the aminoacid 620 (human ApoB numbering) is lysine in long living animals. Green sea urchins have asparagine in the equivalent position, while mouse has glutamic acid. Aminoacid residue 3433 of ApoB is isoleucine in long living organisms. This position is occupied by proline and alanine in short living green sea urchin and mouse. ApoH protein also contains two aminoacids, whose identity varies in consort with lifespan. Aminoacid 203 is occupied by positively charged arginine in human, naked mole rat and *Myotis brandtii*. Red sea urchin also has positively charged aminoacid, lysine, in the same position. Short living creatures, such as green sea urchin and mouse contain isoleucine and leucine at the same place. Aminoacid 253 is represented by asparagine in ApoH of human, naked mole rat and *Myotis brandtii* and glutamic acid in red sea urchin. In contrast, mouse and green sea urchins have threonine and serine in equivalent position. ApoD protein serves as a scaffold for high density lipoproteins. Its sequence harbors two aminoacids that are varied in concert with longevity. ApoD aminoacid 75 (human ApoD numbering) is positively charged in long living species. Human, naked mole rat and *Myotis brandtii* possess lysine, while red sea urchin has arginine at this place. Green sea urchin and mouse have glutamine and glutamic acid at this position. Aliphatic aminoacids isoleucine, valine and leucine could be found at the position 138 of human, naked mole rat, *Myotis brandtii* and red sea urchin. Green sea urchin and mouse have aromatic phenylalanine at this place. Other apolipoproteins analyzed in this study do not have any aminoacids co-varied with longevity.

### Insulin/IGF1 signaling

Caloric restriction is one of the known factors of increase in the lifespan [12]. It is sensed through the insulin/IGF1 signaling pathway. Mutations of the components of this pathway could increase longevity of model organisms up to several fold [11]. We analyzed protein sequences of the IGF1 receptor, PI3K, PTEN, PDK, AKT, TOR, SIRT in a set of long and short living organisms. Phosphatidylinositol kinase PI3K is acting downstream of insulin/IGF receptor and leads to increased biosynthetic and antiapoptotic activity. Mutation of PI3K homolog in *C. elegans*, age-1, doubled lifespan of this organism [40]. Mutations in PI3K gene were found in numerous cancers [41] as well as in individuals predisposed to Cowden syndrome [42], syndrome CLOVES [43] and megalencephaly [44]. Protein sequence of PI3K contains a position which co-varies with longevity. Residue 275 (human PI3K alpha numbering) is occupied with positively charged residues histidine (human PI3K gamma isoform, *H. glaber*, *M. brandtii*) and lysine (red sea urchin). Short living mouse and green sea urchin contain neutral glutamine at this position. It should be noted, however, that human PI3K alpha isoform and nematode age-1 protein contains serine at this position. No other components of insulin/IGF1 signaling pathway contained positions which vary in concert with longevity.

### Telomerase

Senescence of somatic cells in a culture [45] was insightfully associated with telomere shortening by A. Olovnikov [46], which was later demonstrated experimentally [47]. In the germ line, stem and cancer cells telomere length is maintained by telomerase [48]. Influence of telomerase activity on longevity is not as obvious as its influence on senescence of cell cultures. Telomerase is activated in majority of cancer cells and as such its excessive activity might cause increased risk of cancer development. Inhibition of telomerase activity in somatic tissues might be an evolutionary tradeoff between benefits of tissue renovation and risk of cancer. According to previously published work [49], telomerase activity is not ceased in somatic tissues of both long and short living sea urchins. Never the less, we analyzed genes encoding telomerase components in order to identify positions that co-vary with longevity.

The main catalytic component of telomerase is TERT, carrying enzymatic reverse transcriptase activity. In human, mutations Ala202Thr, His412Tyr, Val694Met, Tyr772Cys and Val1090Met leads to defect in bone marrow development [50]. Mutations Lys902Asn, Arg631Gln, Arg811Cys, Arg901Trp and Pro704Ser result in dyskeratosis [51-53], while yet another set of mutations cause pulmonary fibrosis: Arg865His, Val791Ile, Val867Met, Val170Met, Ala716Thr, Lys902Arg and Pro923Leu [14]. Comparison of TERT sequences of the long and short living organisms resulted in identification of several aminoacids that vary in concert with longevity. Position 252 is occupied by glycine in all long living organisms, while in short living organisms it is occupied by arginine. Positively charged aminoacids, lysine and arginine could be found in position 342 of human, naked mole rat and red sea urchin TERT, while green sea urchin and mouse TERT contain glutamine and asparagine at equivalent position. Position 491 is occupied by hydroxyl containing aminoacids, threonine and serine in TERT of human, naked mole rat and red sea urchin. Same position is populated by hydrophobic residues leucine and isoleucine in TERT of green sea urchin and mouse. Aminoacids 342 and 491 belong to the RNA binding domain of TERT. Catalytic, reverse transcriptase domain contains aminoacid 702, being proline in TERT of human, naked mole rat and red sea urchin. Short living green sea urchin and mouse contain asparagine and glutamine at the equivalent position. It is of note that mutations of neighboring proline 704 leads to dyskeratosis in human, which speaks in favor of functional value of the corresponding region of TERT. In C-terminal domain of TERT aminoacid 975 is represented by aspartic acid in long living organisms while short living organisms contain serine at this place. Thus, telomerase reverse transcriptase contains a largest set of positions that co-vary in agreement with longevity.

Pot1 protein binds telomeric repeats and protects telomeres from degradation [54]. Lack of Pot1 leads to senescence of cells in a culture due to telomere shortening. Aminoacid 198 of human and naked mole rat Pot1 is isoleucine. Another hydrophobic residue, valine, occupies the same position of *Myotis brandtii* and red sea urchin. At the same place in Pot1 of the short living mouse and green sea urchin we found threonine and serine.

### Other proteins, related to longevity

In a genetic screen for *Drosophila melanogaster* with increased lifespan a mutation in a gene Indy (I'm not dead yet) was found [55]. This gene codes for the transporter of tricarboxylic acid-cycle intermediates [56]. Although involvement of this gene in longevity was a matter of debates [57, 58], we decided to check if any of aminoacid residues of this protein vary in concert with longevity. Aminoacid, equivalent for *D. melanogaster* aminoacid 61 is a proline in human, *M. brandtii* and naked mole rat. Red sea urchin harbors serine at equivalent position, while short lived green sea urchin and mouse contain asparagine and glutamine. In originally described Indy protein of fly, glutamic acid might be found at this place. Aminoacid 193 (*D. melanogaster* numbering) is serine in Indy protein of human, naked mole rat, *M. brandtii* and glycine in *S. franciscanus*. Both green sea urchin and mouse have asparagine at the same position. It should be mentioned, that original mutations, found in long lived *D. melanogaster* were mapped to noncoding regions and only affected expression level of the gene. In our work we were not able to check expression level of homologous genes in sea urchins. Another Drosophila gene, which was fond in selection experiments towards longer living flies, *mth* [59], was also checked for positions that co-vary with longevity. Unfortunately, no aminoacids that vary in accordance with lifespan were found in our study.

Mutations in a *clk1* gene were found in experiments for selection of long living nematodes [11]. The product of this gene is involved in ubiquinone biosynthesis. Mutations of *clk1* lead to decrease in respiration and as a consequence to increase of the nematode lifespan [60]. Position 117 (*C. elegans* numbering) is occupied by histidine in human, naked mole rat and *M. brandtii*, while red sea urchin has lysine in the equivalent place. *C. elegans* has glutamine in the same position of Clk1, mouse has asparagine and green sea urchin has a glutamic acid.

Another gene related to longevity in *C. elegans* is *daf-9* [61]. This gene codes for cytochrome P450 that is involved in steroid hormone biosynthesis. Aminoacid 132 (*C. elegans* Daf9 numbering) was found to vary in concert with longevity. Aliphatic aminoacids leucine and isoleucine were found at this position of human, *M. brandtii*, naked mole rat and red sea urchin. Short living organisms contain aromatic residues at the same place, tyrosine in *L. variegatus* and phenylalanine in mouse and worm.

### Categories of proteins enriched with positions that co-vary with longevity

**Figure 2:**
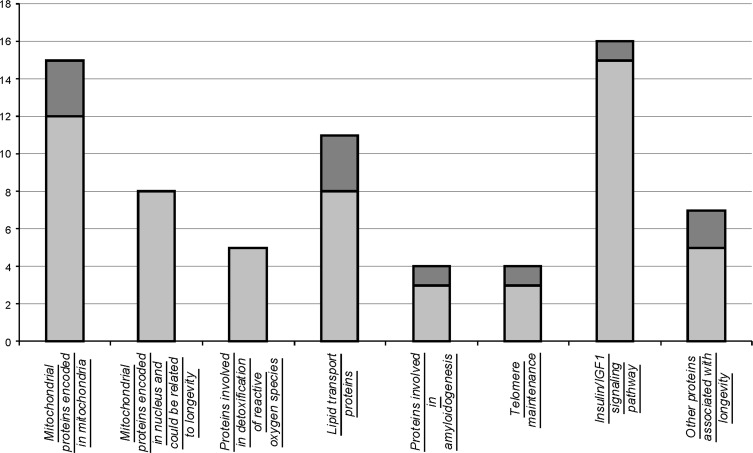
Number of proteins containing aminoacid positions that vary in agreement with longevity. Shown are numbers of analyzed proteins by category (light grey) and those that contained aminoacids that co-vary with longevity (dark grey).

Analysis of protein sequences in a representative set of species with high and low lifespan allowed us to reveal several aminoacid positions that co-vary with longevity. Although this approach is not guaranteed from mistakes originated from misalignment, identification of related proteins that have different function, it could present a framework of further hypothesis-driven experiments on longevity. Our analysis revealed (Figure 2) highly uneven distribution of proteins having aminoacid residues that co-vary with longevity among functional categories. Surprisingly, several categories of proteins were completely devoid of such positions. For example, nuclear encoded mitochondrial proteins and proteins involved in reactive oxygen species inactivation. Minimum of such aminoacids were found in the components of insulin/IGF1 pathway. Particularly enriched in positions that vary in coordination with longevity are categories of mitochondrial proteins, encoded in mitochondrial genome, lipid transport proteins, proteins involved in amyloidogenesis and system of telomere maintenance. Among other, catalytic subunit of telomerase, TERT holds absolute record of the frequency of such positions. Despite the fact, that somatic telomerase activity could be detected in short and long living sea urchins, telomerase reverse transcriptase might be involved in longevity due to more intricate mechanisms, such as maintaining the balance between support of tissue renovation and simultaneous restriction of unwanted proliferation of cancerous cells.

## METHODS

### Sample collection and sequencing

Red sea urchins, *S. franciscanus* were purchased from Marinus Scientific LLC, while green sea urchins, *L. variegatus* were purchased from Gulf Specimen Marine Labs. Samples of sea urchins eggs were collected and used for total genomic DNA purification. DNA samples were fragmented to the libraries of 200, 350 and 500 average fragment length and used for sequencing on Illumina HiSeq instrument. Genomes assembly was done with abyss software [7]. Contigs obtained were mapped onto the genome of *S. purpuratus* using blat software [8] with e-value cutoff 10^−10^. The homologs from *S. purpuratus*, a sea urchin with published genome, were used as a query to search for the contigs in our assemblies of *S. franciscanus* and *L. variegatus* genomes.

### Identification of proteins of interest in the genome assemblies of red and green sea urchins

To identify sequences of proteins which were previously reported to be related to longevity, we implemented in-house script that preformed the following procedures.

With *blastx* software (e-value threshold 10^−5^), the nucleotide sequences of contigs were translated in all possible frames to protein sequences and then aligned to protein sequences of *S. purpuratus*. This procedure yielded the blocks of similarity between regions of contigs and regions of reference proteins. Usually, many contigs were mapped to a single protein sequence. Based on *blastx* alignments, we constructed pseudo-scaffolds from the contigs mapped to single reference protein sequences. To generate a protein sequence of a pseudo-scaffold we took the regions of local blast alignments and combined them for all the contigs mapped to a protein according to the mapping location. If two or more contigs contained conflicting amino acids in a particular position, the amino acid was chosen from the contig with the best (lowest) *blastx* e-value. Gaps (‘-’) were introduced for the amino acids of a reference protein not covered by any contigs from the assembly. We assigned a quality score for every amino acid of a protein pseudo-scaffold representing −log10(*ev*) where ev was the e-value of the best blast hit covering the particular protein position. With *muscle* software, we performed multiple alignments of those sequences.

## CONCLUSIONS

Complete genome sequencing of *S. franciscanus* and *L. variegatus* allows a comparison between closely related long and short living species. Moreover, it adds to a list of species with exceptional longevity whose genome sequence was determined. In turn, it allowed comparison of representative set of proteins from short and long living creatures aiming at identification of positions that vary in agreement with longevity. Despite being certainly a sort of oversimplification, such an analysis might present a frame for further experimental validation of potential protein targets that might influence longevity.

Short read data have been deposited into the Short Read Archive (http://www.ncbi.nlm.nih.gov/sra) under the accession numbers SRX1316769 (*S. franciscanus*) and SRX1317962 (*L. variegatus*).

## SUPPLEMENTARY MATERIAL


