# Supplementary material for: Genomes of Strongylocentrotus franciscanus and Lytechinus variegatus: are there any genomic explanations for the two order of magnitude difference in the lifespan of sea urchins?
Source: Aging (Albany NY). 2016 Feb 7;8(2):260–71. doi: 10.18632/aging.100889 (PMC4789581; doi:10.18632/aging.100889)
Supplement: Supplementary file 1 [file aging-08-260-s001.doc]

**Supplementary material**

**Genomes of *Strongylocentrotus franciscanus* and *Lytechinus variegatus*: are there any genomic explanations for the two order of magnitude difference in the lifespan of sea urchins?**

**Petr V. Sergiev^1^, Artem A. Artemov^2,3^, Egor B. Prokhortchouk^3^, Olga A. Dontsova^1^, and Grigory V. Berezkin^4^**

*^1^Lomonosov Moscow State University, Department of Chemistry and A.N. Belozersky Institute of Physico-Chemical Biology, Moscow 119992, Russia*

*^2^Lomonosov Moscow State University, Faculty of Bioengineering and Bioinformatics, Moscow 119992, Russia*

*^3^Center ‘Bioengineering’, Russian Academy of Sciences, Moscow, 117312 Russia and National Research Center, Kurchatov Institute, Moscow 123098 Russia*

*^4^ESN Group, 123100, Moscow, Russia*

Alignments of longevity related proteins from long and short living organisms

Aligned line SIG, where “+” indicate positions whose identity vary is consort with longevity is shown where such positions were detected

AKT

>H sapiens

----------------------MSDVAIVKEGWLHKRGEYIKTWRPRYFLLKNDGTFIGY

KERPQDVDQREAPLNNFSVAQCQLMKTERPRPNTFIIRCLQWTTVIERTFHVETPEEREE

WTTAIQTVADGLKKQEEEEMDFRSGSPS-DNSGAEEMEVSLAK-PKHRVTMNEFEYLKLL

GKGTFGKVILVKEKATGRYYAMKILKKEVIVAKDEVAHTL-TENRVLQNSRHPFLTALKY

SFQTHDRLCFVMEYANGGELFFHLSRERVFSEDRARFYGAEIVSALDYLHSEKNVVYRDL

KLENLMLDKDGHIKITDFGLCKEGIKDGATMKTFCGTPEYLAPEVLEDNDYGRAVDWWGL

GVVMYEMMCGRLPFYNQDHEKLFELILMEEIRFPRTLGPEAKSLLSGLLKKDPKQRLGGG

SEDAKEIMQHRFFAGIVWQHVYEKKLSPPFKPQVTSETDTRYFDEEFTAQMITITPPDQ-

DDSMECVDSERRPHFPQFSYSASGTA---

>H glaber

----------------------MNDVAIVKEGWLHKRGEYIKTWRPRYFLLKNDGTFIGY

KERPQDVDQRESPLNNFSVAQCQLMKTERPRPNTFIIRCLQWTTVIERTFHVETPEEREE

WTTAIQTVADGLKRQEEELMEFQSGSPS-DNSGAEEMEVSLAK-PKHRVTMNEFEYLKLL

GKGTFGKVILVKEKATGRYYAMKILKKEVIVAKDEVAHTL-TENRVLQNSRHPFLTALKY

SFQTHDRLCFVMEYANGGELFFHLSRERVFSEDRARFYGAEIVSALDYLHSEKNVVYRDL

KLENLMLDKDGHIKITDFGLCKEGIKDGATMKTFCGTPEYLAPEVLEDNDYGRAVDWWGL

GVVMYEMMCGRLPFYNQDHEKLFELILMEEIRFPRTLGPEAKSLLSGLLKKDPKQRLGGG

SEDAKEIMQHRFFASIMWQDVYEKKLSPPFKPQVTSETDTRYFDEEFTAQMITITPPDQ-

DDNMECVDSERRPHFPQFSYSASGTA---

>M brandtii

-------------------------------------GEYIKNWRPRYFILKTDGSFIGY

KEKPQDVDL-PYPLNNFSVAKCQLMKTERPKPNTFIIRCLQWTTVIERTFHVDTPEEREE

WTEAIQAVADRLQRQEEERMSCCSPTSQIDNIGEEEMDASATH-HKRK-TMNDFDYLKLL

GKGTFGKVILVREKASGKYYAMKILKKEVIIAKDEVAHTL-TESRVLKNTRHPFLTSLKY

SFQTKDRLCFVMEYVNGGELFFHLSRERVFSEDRTRFYGAEIVSALDYLHS-GKIVYRDL

KLENLMLDKDGHIKITDFGLCKEGITDTATMKTFCGTPEYLAPEVLEDNDYGRAVDWWGL

GVVMYEMMCGRLPFYNQDHEKLFELILMEDIKFPRTLSSDAKSLLSGLLIKDPNKRLGGG

PDDSKEIMRHSFFSGVNWQDVYDKKLVPPFKPQVTSETDTRYFDEEFTAQTITITPPEKY

DED--GMDNESRPHFPQFSYSASGRE---

>S franciscanus

--------------MTTTMSVAEAPLTVIKEGWVQKRGEIAKITQKQLL-------FVFF

CLWP---------------AECQILKTEKPAPNTFKVRCLQWTTIIERTFSVSSADERER

KPSTHPTILTVLIKLACIRH---------IHIIAHYHHYAPYFIYPSLMTLDDFQLLKCL

GKGHFGKVIQVREKKTGELFAIKYLKKAVIVQKEAVRNVL-REQEILQALEHPFLVSLKY

SFQTADKLYFVMEYVNGGELFFHLQREKKFSEERARFYAAEIASALEYLHT-HDVIYRDL

KAENILLDCDGHVKLADFGLCKEGIAAGDTTSTFCGTPEYIAPEIIQEKPYGKAVDWWAL

GVLLYEMLVGTPPFYGESIFQLFSLILHDAISFPRWLSKEAHSILSALLKKHPKARLGSG

PEDAQKIKKHQFFRHIKWERLAARQIQPPFVPQVASETDTRYFDQEFTLEPVRLTPPGSG

ADTLTATLHEEQSQFEKFSYTDDVPLQK-

>S purpuratus

--------------MTTTMSVSEAPITVIKEGWVQKRGEYIKNWRPRYFQLKSNGDFVGY

KDKPKGKSGDSDALNNFTVAQCQILKTEKPAPNTFKVRCLQWTSFIERTFSVASNDERES

WVKALQGVTDSLTRQEREKENQEANA---ANGGKEDSNSAANEASKHKVTMDDFELLKCL

GKGTFGKVIQVREKKTGELFAIKVLKKEVIVQKDEVAHTL-TENRVLQKTSHPFLTSLKY

SFQTSDRLCFVMEYVNGGELFFHLSRERIFTEERTRFYGAEIVSALSYLHA-QDVIYRDL

KLENLLVDEEGHIKITDFGLCKENISYGDMTKTFCGTPEYLAPEILEDNDYGRAVDWWGT

GVVMYEMMCGRLPFYSRDHEVLFDLILTENVKFPTRLSNEAKSLLSGLLEKNPKKRLGCG

PDDAKEIMRHPFFATINWEDLYERKVRPPFLPKVENETDTRYFDQEFTLEPVRLTPPAAG

KDTLTATQHDEQSQFEKFSYTDDVPLQK-

>L variegatus

SILKCIRARQFAHKSQNISACSAVCFSIIAPAIYQQSIAHIISIQEQCI-------IAGY

QSYSI--------FANLSIAECQILKTEKPAPNTFKVRCFQWTTLIERTFSVSSSEERES

WVKALQGVADALHKQEQEQNQLAKNE---ANGGKNDSNSTVDDASKLKMTMEDFRLLKCL

GKGTFGKVILTRYKKTGKYYAIKVLKKADIVKKDEVAHVL-REQAILQAISHPFLFSLHW

THQTDTFLYMLLEYACGGELFYHLSQSARFSEERSKFYAAEIISALDYLHK-QNIIYRDL

KPENILLDEEGHIKIADFGLCKEGIGYGDKTSTFCGTPEYIAPEIILNKGYGLSVDWWSL

GVLLYEMLTGKPPFYGEDEDQLFDLILHDSPHYPRWLSKDAHSILKALLEKHPGKRLGCG

PKDAQEIREHQFFRSIDWEKLYERQIQPPFLPQVAHETDTRYFDQEFTLEPAKLTPPGAG

ELTL-CIVTGKGSELDLSLLDDFFCLECL

>M musculus

----------------------MNDVAIVKEGWLHKRGEYIKTWRPRYFLLKNDGTFIGY

KERPQDVDQRESPLNNFSVAQCQLMKTERPRPNTFIIRCLQWTTVIERTFHVETPEEREE

WATAIQTVADGLKRQEEETMDFRSGSPS-DNSGAEEMEVSLAK-PKHRVTMNEFEYLKLL

GKGTFGKVILVKEKATGRYYAMKILKKEVIVAKDEVAHTL-TENRVLQNSRHPFLTALKY

SFQTHDRLCFVMEYANGGELFFHLSRERVFSEDRARFYGAEIVSALDYLHSEKNVVYRDL

KLENLMLDKDGHIKITDFGLCKEGIKDGATMKTFCGTPEYLAPEVLEDNDYGRAVDWWGL

GVVMYEMMCGRLPFYNQDHEKLFELILMEEIRFPRTLGPEAKSLLSGLLKKDPTQRLGGG

SEDAKEIMQHRFFANIVWQDVYEKKLSPPFKPQVTSETDTRYFDEEFTAQMITITPPDQ-

DDSMECVDSERRPHFPQFSYSASGTA---

APOA

>H sapiens

--------------------------MEHKEVVLLLLLFLKSAAPEQSHVVQDCYHGDGQ

SYRGTYSTTVTGRTCQAWSSMTPHQHNRTTENYPNAGLIMNYCRNPDAVAAPYCYTRDPG

VRWEYCNLTQCSDAEGTAVAPPTVTPVPSLEAPSEQAPTEQRPGVQECYHGNGQSYRGTY

STTVTGRTCQAWSSMTPHSHSRTPEYYPNAGLIMNYCRNPDAVAAPYCYTRDPGVRWEYC

NLTQCSDAEGTAVAPPTVTPVPSLEAPSEQAPTEQRPGVQECYHGNGQSYRGTYSTTVTG

RTCQAWSSMTPHSHSRTPEYYPNAGLIMNYCRNPDAVAAPYCYTRDPGVRWEYCNLTQCS

DAEGTAVAPPTVTPVPSLEAPSEQAPTEQRPGVQECYHGNGQSYRGTYSTTVTGRTCQAW

SSMTPHSHSRTPEYYPNAGLIMNYCRNPDAVAAPYCYTRDPGVRWEYCNLTQCSDAEGTA

VAPPTVTPVPSLEAPSEQAPTEQRPGVQECYHGNGQSYRGTYSTTVTGRTCQAWSSMTPH

SHSRTPEYYPNAGLIMNYCRNPDAVAAPYCYTRDPGVRWEYCNLTQCSDAEGTAVAPPTV

TPVPSLEAPSEQAPTEQRPGVQECYHGNGQSYRGTYSTTVTGRTCQAWSSMTPHSHSRTP

EYYPNAGLIMNYCRNPDAVAAPYCYTRDPGVRWEYCNLTQCSDAEGTAVAPPTVTPVPSL

EAPSEQAPTEQRPGVQECYHGNGQSYRGTYSTTVTGRTCQAWSSMTPHSHSRTPEYYPNA

GLIMNYCRNPDAVAAPYCYTRDPGVRWEYCNLTQCSDAEGTAVAPPTVTPVPSLEAPSEQ

APTEQRPGVQECYHGNGQSYRGTYSTTVTGRTCQAWSSMTPHSHSRTPEYYPNAGL-IMN

YCRNPDPVAAPYCYTRDPSVRWEYCNLTQCSDAEGTAVAPPTITPIPSLEAPSEQAPTEQ

RPGVQECYHGNGQSYQGTYFITVTGRTCQAWSSMTPHSHSRTPAYYPNAGLIKNYCRNPD

PVAAPWCYTTDPSVRWEYCNL----TRCSDAEWTAFVPPNVILAPSLEAFFEQALTEETP

GVQDCYY-HYGQSYRGTYSTTVTGRTCQAWSSMTPHQHSRTPENYPNAGL-TRNYCRNPD

AEIRPWCYTMDPSVRWEYCNLTQCLVTESSVLATLTVVPDPSTEASSEEAPTEQSPGVQD

CYHGDGQSYRGSFSTTVTGRTCQSWSSMTPHWHQRTTEYYPNGGLTRNYCRNPDAEISPW

CYTMDPNVRWEYCNLTQCPVTESSVLATSTAVSEQAPTEQSPTVQDCYHGDGQSYRGSFS

TTVTGRTCQSWSSMTPHWHQRTTEYYPNGGLTRNYCRNPDAEIRPWCYTMDPSVRWEYCN

LTQCPVMESTLLTTPTVVPVPSTELPSEEAPTENSTGVQDCYRGDGQSYRGTLSTTITGR

TCQSWSSMTPHWHRRIPLYYPNAGLTRNYCRNPDAEIRPWCYTMDPSVRWEYCNLTRCPV

TESSVLTTPTVAPVPSTEAPSEQAPPEKSPVVQDCYHGDGRSYRGISSTTVTGRTCQSWS

SMIPHWHQRTPENYPNAGL-TENYCRNPDSGKQPWCYTTDPCVRWEYCNL----TQCSET

ESGVLETPTVVPVPSMEAHSEAAPTEQTPVVRQCYH-GNGQSYRGTFSTTVTGRTCQSWS

SMTPHRHQRTPENYPNDGL-TMNYCRNPDADTGPWCFTMDPSIRWEYCNL----TRCS--

---------DTEGTVV----APPTVIQVPSLGP-------------------PSE-----

-----------------------QDCMFGNGKGYRGKKATTVTG----------------

----TPCQEWAAQEPHRHSTFIPGTNK--WAGLEKNYCRNPDGDI---------------

---NGP---------WCYTMNPRK----------------------------LFDYCDIP

LCAS-SSFDCGK------PQVEPKKCPGSIVGGCVAHPHSWPWQVSLRTR----------

-FG-KHFC--GGTLISPEWVLTAAHCLKKSSRPSSYKVILGAHQE---------------

----------VNLESHVQEIEVSRLFLEPTQADIALLKLSRPAVITDKVMPACLPSPDYM

VTA-------------------------------------------------RTECYITG

W-----------------------GETQGTFGTGLLKEAQLLV----IENEVCNHYKY--

-------ICAEHLARGTDSCQGDSGG--PLVCFEKDKYILQ------GVTS---------

------------------------------------------------------------

------------------------------------------------------------

------------------------------------------------------------

---------------------------------------------------------WGL

GCAR-----------------------------P------------------------NK

PGVYARVSRFVTWIEGMMRNN-------------------

>H_glaber

MSLISCKSIISQDWDTVFQTLPGSPAMDHKEVILLFLLFLKS------------------

------------------------------------------------------------

--------------------------------------------------GQGIS-----

------------------------------------------------------------

-QDDYENTRGASLVSLTKKQLGA-------------ANIDEC------------------

-------------------------------------------------------AAKCE

EE--------------------------------------------------TEFVCRSF

Q-----------------------------------------------------------

------------------------------YHSKEE------------------------

---------------------------------------------QC-------------

------------------------------------------------------------

-------VIM----AENSKSSPIIRMRD--------------------------------

------------------------------------------------------------

--VILF------------------------------------------------------

---EKKVYLLECRTGNGKNYRGTMSRTKSGVTCQKWGATFPHRPNYSPGTHPAEGL-ENN

YCRNPD------------------------NDPEG-------------------------

------------------------------------------------------------

----PWCYTTNPDKRYDFCDI----PECED------------------------------

---ECMH-CLGENYMGKISTTVSGLECQAWDSQVPHAHGFIPSKHPNKNL-KMNYCRNPD

GELRPWCFTTDPNKRWEFCNISRC------------------------------------

------------------------------------------------------------

------------------------------------------------------------

------------------------------------------------------------

------------------------------------------------------------

------------------------------------------------------------

------TTP---------------PPPPGPVYQ-CLKGRGENYRGKVAVTVSGRTCQRWS

EQTPHKHNRTPENYLCKDL-EENYCRNPDRETAPWCYTTDSKVRWEYCAI----PSCESS

Q-----------LSAEHSDGPALP-EQTPVVQECYH-GNGQSYRGTSSITVTGKKCQSWS

SVRPHVHGKTPENYPNAGL-TMNYCRNPDVDKSPWCYTTDPSVRWEYCNL----KKCS--

---------ETEGSVV----ESQTTAQVPSVQA-------------------TSE-----

-----------------------PDCMVGYGKEYRGKMATTVAG----------------

----IPCQGWADQEPHKHRIFTPESNP--QAGLEENYCRNPDGDF---------------

---SGP---------WCYTMSPTK----------------------------LYDYCDVP

QCAP-SSYDCGK------PRVEPKKCPGRVVGGCVANPHSWPWQVSLRTR----------

-FELRHFC--GGTLIAPEWVLTAAHCLEKSLNPAFYKVILGAHQE---------------

----------INLESNVQRKDVTKLFLEPHRADIALLKLSSPAVITDKIIPACLPSSNYM

VAD-------------------------------------------------RTLCYITG

W-----------------------GETKGTYGAGLLKEAQLPV----IENKVCNRFEYLN

GRVKPTELCAGHLAGGTDSCQGDSGG--PLVCFEKDKYILQ------GVTS---------

------------------------------------------------------------

------------------------------------------------------------

------------------------------------------------------------

---------------------------------------------------------WGL

GCAR-----------------------------P------------------------NK

PGVYVRVSTFVKWIEEIMGTN-------------------

>M_brandtii

--------------------------MGRQELLLLLLLFLRP------------------

------------------------------------------------------------

--------------------------------------------------GHGDP-----

------------------------------------------------------------

-LDSYVNTQGASLITLTRKELPA-------------GSIAEC------------------

-------------------------------------------------------AAKCE

EE--------------------------------------------------TTFTCRSF

Q-----------------------------------------------------------

------------------------------YHSKEQ------------------------

---------------------------------------------HC-------------

------------------------------------------------------------

-------VVM----GDNSKSAPVLRMRD--------------------------------

------------------------------------------------------------

--VVLY------------------------------------------------------

---EKRIYLSECKTGNGRRYRGTVSKTKNGVACQKWSDHSPHIPRFSPTSHPLEGL-EEN

YCRNPD------------------------NDEKG-------------------------

------------------------------------------------------------

----PWCYTTDPATRFDYCTI----PECEE------------------------------

---ECMH-CSGENYEGTISRTKSGLECQAWDSQTPHAHGYIPAKFPSKNL-KMNYCRNPD

GEPSPWCFTTNPNKRWELCDIPRC------------------------------------

------------------------------------------------------------

------------------------------------------------------------

------------------------------------------------------------

------------------------------------------------------------

------------------------------------------------------------

------TTP---------------PPSSGPTHQ-CLKGRGESYVGKVAVTLSGHTCQRWS

EQTPHKHNRTPENFPCKNL-EENYCRNPDGESAPWCFTTNSKVRWEHCKI----PSCESS

Q-----------LSPEQAEATVPP-EQTPVVQECYH-GNGQSYRGTSSTTITGRKCQSWS

SMTPHRHSKTPDQIPNAGL-TMNYCRNPDGDKGPWCYTTDPSVRWEFCNL----KKCS--

---------ETQGAVMNTPPEVPPVS--PQGSS-------------------EAEAGPDI

GEPAAAALVPHPRSTEPDLRPLPADCMLGNGKGYRGKKATTVAG----------------

----TACQAWAAQEPHRHSIFTPETNP--RAGLEKNYCRNPDGDV---------------

---NGP---------WCYTTDPKK----------------------------LFDYCDVP

QCAS-SSFDCGK------PKVEPKKCPGRVVGGCVANPHSWPWQVSLRTR----------

-FR-MHFC--GGTLLSPDWVLTAAHCLERSSRPSSYKVILGAHHE---------------

----------QNLEADVQERDVSKLVLEPTRADIALLKLSSPAIITDKVIPACLPSADYV

VAD-------------------------------------------------RTVCYITG

W-----------------------GETQGTFGAGLLKEAQLPV----IENKVCNRYEYLN

GRVKSTELCAGLLAGGADSCQGDSGG--PLVCFEKDKYILQ------GVTS---------

------------------------------------------------------------

------------------------------------------------------------

------------------------------------------------------------

---------------------------------------------------------WGL

GCAR-----------------------------P------------------------NK

PGVYVRVSRFVKWIEETMKNN-------------------

>S franciscanus

--------------------------RAQETYLIL-------------------------

------------------------------------------------------------

------------------------------------------------------------

------------------------------------------------------------

----------DSLVLPTV------------------------------------------

-------------------------------------------------------PNECK

LD----------------------------------------------------------

------------------------------------------------------------

------------------------------------------------------------

------------------------------------------------------------

------------------------------------------------------------

------------------------------------------------------------

------------------------------------------------------------

------------------------------------------------------------

--------------PTGVNYRGNLSQTLSGRECQSWTSQYPHEHSRTPSNYPDAGLGDHN

YCRNPD------------------------GEFTA-------------------------

------------------------------------------------------------

-----WCYTTDPSVRWEYCAIGDPSPECSSA-----------------------------

---YCYNDPYGTIYRGELSQTRSGKECQSWTSQSPHEHSRTPSNYPDAGLGDHNYCRNPD

GEFTAWCYTTDPDVRWEYCAIG--------------------------------------

------------------------------------------------------------

------------------------------------------------------------

------------------------------------------------------------

------------------------------------------------------------

------------------------------------------------------------

------------------------DPSEECTDD----PTGTIYRGELSQTLSGKTCQSWT

SQYPHEHSRTPSNYPDAGLGDHNYCRNPDGEFTAWCYTTDPDVRWEYCAIGDFSPECTSK

Y--------------------------------CYDDPKGTIYRGELSQTLSGKECQSWT

SQSPHEHSRTPSNYPDAGLGDHNYCRNPDGEFTAWCYTTDPDVRWEYCAIGLPQEECTVG

HLDVTKYLISQGAEVNRGDNDGRTALHIAALKGHLDVTKYLISQGA------EVNKGDND

GRTALHLAAQEGH--LDISKKAGQEGRVLETTDGHGSVKVKVRGQTLKYHPAALILLQGA

EVNKGDNDGWTALHSAAFNGHLDVTKYLISQGAEVNKGDNDGWTALHIAAQNGHLDVTKY

LISQGAEVNKGDNDGRTALHSAAQEGHLDVTKYLISQGAE-------VEMVDNHDQTPLI

AAAQNGHLDVTKYLISQGAEVNKGDNDGRTALHSAAQNGHLDVTKYLISQGAEVNKGDND

GRTALHSAAQNGHLDVTKYLISQGAEVNKGDNDGRTALHLAAQNGHLDVVKYLISQGAEV

NKGDNDGRTPLHLALQLGYQSIADLFIDHSSADLRLRNNDAFPPLHYAAKKNCLEMVKLL

VAKDPSLATIEKAHRYTPLHVAAINNHVDIVRVLIELPNCDL-TTIKASQGHSTPLHLAT

WEGYTEVIELLVSHGAEVNVKDKDGDTMLHLTIKRRVSGKKFVEDTPTLQKVCKEIGDPA

ITTPRDAIIAFFIRHGSDVTIKNNNGHTPFDLLDDERVLLNALMRVAGITP-DELSESLM

ECCTPIR----LRYQSFIYKNSHHTPSSFATDADFEGVHRISPDELNLGETVGRGGFGEV

KKAVWRGTTVAVKIISTAGSKKAEIEKEVSIHKRARHPNIVSLMAVGHRIGQALIVMEFI

DGMDLYDVIFRKK-----VFYENRRKISGKESKERKIPQKDEPHQVRIIFGNISGIPQVE

RASRRAYLCDLGLAHIKTRSSMSLSTSVGGPRGTITHMPPEMVQKEGKAWAGPGNDIWSI

ACTFLELFVEQRLWEGVEFMALLRMMLKPAAVPP--ILKKVKPEVRTILKPCFEKEPLKR

PSAEVLLEQFQKLQQSSK----------------------

>S_purpuratus

-------------------------------MAQLFLYMALA------------------

------------------------------------------------------------

------------------------------------------------------------

------------------------------------------------------------

----------ATFVLVSS------------------------------------------

-------------------------------------------------------QSDCT

DD----------------------------------------------------------

------------------------------------------------------------

------------------------------------------------------------

------------------------------------------------------------

------------------------------------------------------------

------------------------------------------------------------

------------------------------------------------------------

------------------------------------------------------------

--------------PNGTIYRGELAQTFSGKTCQSWNSQNPQRHSRTPGNYPDAGLGDHN

YCRNPD------------------------SAFTA-------------------------

------------------------------------------------------------

-----WCYTTDPDTRWEYCTIGDFSQECTNP-----------------------------

---ECYHQSPGADYRGEVSTTRNGRECQNWTSQSPHGHSRTPENYPTSGLGDHNLCRNPD

GEDFAWCYTTDPSVRWEFCNIG--------------------------------------

------------------------------------------------------------

------------------------------------------------------------

------------------------------------------------------------

------------------------------------------------------------

------------------------------------------------------------

------------------------LPEKNCTDD----PNGTIYRGELAQTFSGKTCQSWN

SQTPQGHSRTPANYPNAGLGDHNYCRNPDGAFTAWCYTTDPDTRWEYCTIGDFSPECTNP

E--------------------------------CYHQSPGADYRGEVSTTRNGRECQNWT

SQSPHGHSRTPDNYPTSGLGDHNLCRNPDGEEFAWCYTTDPSVRWEFCNIGLPEEVLAL-

FQRVREFPPSGFGEEEGGEPDGYPCCTIDHHGEGAPVVDFKAGDRV------RIS-ANID

MVKDLHRKNEERNDAIELSKKAGQEGRVLESTDGHGSVKVKVKGQTLKYHPAALILLPKR

D---GDDEEEEEEEEEKVEDKKEEKEKEEKVEVKKDKEEKEDGEKRHDDKKKKSDEEYEN

IITNAPQVGSK----PVPIPVPAGPADKDVMHRLVEKMTEQLKDAGVSELVTTSEEKQMV

KAVAAGNLEKVKDFLRQNPKLANASHSSRSALQVACHEGSLPIVQLLVDAHASLEHKDGD

GDTALTFAVIGNNPSVAEFLMERKAYVNTSNKRRRTPMHISAHKDHKSCVEALIKHGGSV

NMQDNDGNLPIHLAIKQKSTSLGALFIDHPSADLRLRNNDAFPPLHYAAKRNCLEMVKLL

VAKDPSLATIEKDDRYTPLHVAAINNHIDIVRVLIELPNCDL-TTINAGHGHSTPLHLAT

WQGYTEVIELLVSHRAEVNVKDKDGDTMLHLTVKRRVSGKKFVHDTPTLQKVCKEIGDPA

ITTPRDAIIAYFIRHGSDVTIKNNNGHTPFNLLENEPVLLNALMRVAGITPINEIPIAAD

EAVDKTRKEEKKKDKEERSPSAEKAPKPEKKQADFEGVHRISPEELKLGKTIGQGGFGEV

KKAVWRGTTVAVKIISTAGSKEDEVEKEVSIHKRARHPNIVSLMAVGHRIGQVLIVMEFI

DGMDLHDVIFRKKKDGITLTPELKMSITVGLLSALTFLHASKILHLDIKPSNV----MVE

RASRRAYLCDLGLAHIKTRSSMSLSTSMGGPRGTITHMPPEMVQREGKAWAGPGNDIWSI

ACTFLELFVEQRLWEGAEFMVLLRTLLKPASVPP--ILKKVKTEVRTILKPCFDKEPLKR

PSAEVLLEQFQKLQQSSP----------------------

>L variegatus

--------------------------------LQFFSIFLTK------------------

------------------------------------------------------------

------------------------------------------------------------

------------------------------------------------------------

----------FKFFTFAE------------------------------------------

-------------------------------------------------------THTCE

LD----------------------------------------------------------

------------------------------------------------------------

------------------------------------------------------------

------------------------------------------------------------

------------------------------------------------------------

------------------------------------------------------------

------------------------------------------------------------

------------------------------------------------------------

--------------PSGTNYRGYLSTTRSGKTCQSWTSQTPHGHSRTPDNYPDAGLGNHN

YCRNPD------------------------REFTA-------------------------

------------------------------------------------------------

-----WCYTTDPEVRWEYCAIGDLDPECTDP-----------------------------

---ECYKDPSGTDYRGNVSTTRSGKTCQAWTSQTPHGHSRTPDNYPDAGLGNHNYCRNPD

REFTAWCYTTDPDTRWEYCNIG--------------------------------------

------------------------------------------------------------

------------------------------------------------------------

------------------------------------------------------------

------------------------------------------------------------

------------------------------------------------------------

------------------------LPDPECTED----PSGTDYRGYVSTTRSGKTCQAWT

SQTPHGHSRTPDNYPDAGLGNHNYCRNPDREFTAWCYTTDPDTRWEYCNIGDLSPECTDP

E--------------------------------CYKDPSGTDYRGNVSTTRSGKTCQAWT

SQTPHGHSRTPDNYPDAGLGNHNYCRNPDREFTAWCYTTDPDTRWEYCNIGLPQSDCA--

-----DFLLQGGSHIFFFKLE--ICCS--NLRERTAISTFLISKSVGLKLFLKAKIEKKK

GKKPLLAAAQRLH--IDLPIILISQGAEVNKRNFTGWTALHSAAFIGHLDVNKYLISQGA

EINRGETDGWTALHFAAKNGHLDITQYLISQGAQVNRGNITGSTALHIAAENGHLDVTKY

LISQGAEVNKGDNDGWTALHSAAENGHLDVIKYLISQGAD-------VNKGDNDGWTALH

RAAENGHLDVTKYLISQGAEVNKGDNDGWTALHIAAQNGHLDVVKYLISQGAEVNKGDND

GWTALHIAAENGHLDVTKYLISQGAEVNKGDNDGWTALHIAAQNGHLDVTKYLISQGAEV

NKGDNDGWTALHIAIKNGHLDVVKYLISQGAAALKASTASGETALHYAAKRNCLRVVKLM

VAKDPSLALIEKDDGYTPLHVAAINNHLEISRVLINLL-CKIPATIDAGEGHSTPLHLAT

WQGYSELVELLVSHGAAINLKDKDGDTVLHLSIRRRKSGQKFVQDTPIKSSVCKEVKETA

ITTPRDALIAYFIQHGSDVTIKNKNGHTPFELLDEEPLLLNALMRVAGIKP-DKVIIAQC

MIVIALH-QMHCKAQSHCYNQIYSIPQAISIPSDFEGVHIIQVCDLDMGRMVGKGQFGVV

HHASWRGTTVAVKKISIAGSRKEEVEKEVAIHRRARHPNIVSLMAVGHRIGQALIVMEFI

DGMDLNDVIFKKKKDGITLTKELKMSTTLGLLSALTYLHASKILHLDIKPSNV-MVSSIY

LHAAKGILYEYILSVLYQ-----------------IYISPQFI-------PSPCSHSSHI

ILQHYQLI-------GTLHKIISTISYQSHGIWPKSILSTI-SHMSYIINQC----TLDK

SISSHHIIQFGLLSMSSHGINFGPLSGHLHMGINLVYYQV

>M_musculus

--------------------------MDHKEVILLFLLLLKP------------------

------------------------------------------------------------

--------------------------------------------------GQGDS-----

------------------------------------------------------------

-LDGYISTQGASLFSLTKKQLAA-------------GGVSDC------------------

-------------------------------------------------------LAKCE

GE--------------------------------------------------TDFVCRSF

Q-----------------------------------------------------------

------------------------------YHSKEQ------------------------

---------------------------------------------QC-------------

------------------------------------------------------------

-------VIM----AENSKTSSIIRMRD--------------------------------

------------------------------------------------------------

--VILF------------------------------------------------------

---EKRVYLSECKTGIGNGYRGTMSRTKSGVACQKWGATFPHVPNYSPSTHPNEGL-EEN

YCRNPD------------------------NDEQG-------------------------

------------------------------------------------------------

----PWCYTTDPDKRYDYCNI----PECEE------------------------------

---ECMY-CSGEKYEGKISKTMSGLDCQAWDSQSPHAHGYIPAKFPSKNL-KMNYCRNPD

GEPRPWCFTTDPTKRWEYCDIPRC------------------------------------

------------------------------------------------------------

------------------------------------------------------------

------------------------------------------------------------

------------------------------------------------------------

------------------------------------------------------------

------TTP---------------PPPPSPTYQ-CLKGRGENYRGTVSVTVSGKTCQRWS

EQTPHRHNRTPENFPCKNL-EENYCRNPDGETAPWCYTTDSQLRWEYCEI----PSCESS

A------------SPDQSDSSVPPEEQTPVVQECYQ-SDGQSYRGTSSTTITGKKCQSWA

AMFPHRHSKTPENFPDAGL-EMNYCRNPDGDKGPWCYTTDPSVRWEYCNL----KRCS--

---------ETGGSVV----ELPTVSQEPSGPS-------------------DSE-----

-----------------------TDCMYGNGKDYRGKTAVTAAG----------------

----TPCQGWAAQEPHRHSIFTPQTNP--RAGLEKNYCRNPDGDV---------------

---NGP---------WCYTTNPRK----------------------------LYDYCDIP

LCASASSFECGK------PQVEPKKCPGRVVGGCVANPHSWPWQISLRTR----------

-FTGQHFC--GGTLIAPEWVLTAAHCLEKSSRPEFYKVILGAHEE---------------

----------YIRGLDVQEISVAKLILEPNNRDIALLKLSRPATITDKVIPACLPSPNYM

VAD-------------------------------------------------RTICYITG

W-----------------------GETQGTFGAGRLKEAQLPV----IENKVCNRVEYLN

NRVKSTELCAGQLAGGVDSCQGDSGG--PLVCFEKDKYILQ------GVTS---------

------------------------------------------------------------

------------------------------------------------------------

------------------------------------------------------------

---------------------------------------------------------WGL

GCAR-----------------------------P------------------------NK

PGVYVRVSRFVDWIEREMRNN-------------------

>SIG

------------------------------------------------------------

------------------------------------------------------------

------------------------------------------------------------

------------------------------------------------------------

-------------+----------------------------------------------

------------------------------------------------------------

------------------------------------------------------------

------------------------------------------------------------

------------------------------------------------------------

------------------------------------------------------------

------------------------------------------------------------

------------------------------------------------------------

------------------------------------------------------------

------------------------------------------------------------

------------------------------------------------------------

------------------------------------------------------------

------------------------------------------------------------

------------------------------------------------------------

------------------------------------------------------------

------------------------------------------------------------

------------------------------------------------------------

------------------------------------------------------------

------------------------------------------------------------

------------------------------------------------------------

------------------------------------------------------------

------------------------------------------------------------

------------------------------------------------------------

------------------------------------------------------------

------------------------------------------------------------

------------------------------------------------------------

-----------------------------------------+------------------

------------------------------------------------------------

------------------------------------------------------------

------------------------------------------------------------

------------------------------------------------------------

------------------------------------------------------------

------------------------------------------------------------

------------------------------------------------------------

------------------------------------------------------------

------------------------------------------------------------

------------------------------------------------------------

------------------------------------------------------------

------------------------------------------------------------

------------------------------------------------------------

APOB

>H sapiens

--------------------MDPPRPALLALLALPALLLLLLAGARAEEEML--ENVSLV

CPKDATRFKHLRKYTYNYEAESSSGVPGTADSRSATRINCKVELEVPQLCSFILKTSQCT

LKEVYGFNPEGKALLKKTKNSEEFAAAMSRYELKLAIPEGKQVFLYPEKDEPTYILNIKR

GIISALLVPPETEEAKQVLFLD-TVYGNCSTHFTVKTRKGNV-ATEISTERDLGQCDRFK

PIRTGISPLALIKGMTRPLSTL---ISSSQSCQYTLDAKRKHVAEAICKEQHLFLPFSYK

NKYGMVAQVTQTLKLEDTPKINSRFFGEGTKK---MGLAFESTKSTSPPKQ--AEAVLKT

LQELKKLTISEQNIQRANLFNKLVTELRGLSDEAVTSLL----------------PQLIE

VSSPITLQALVQCGQPQCSTHILQWLKRVHANPLLIDVVTYLVALIPEPSAQQLREIFNM

ARDQRSRATLYALSHAVNNYH------KTNPTGTQELLDIANYLMEQIQDDCT---GDED

YTYLILRVIGNMGQTMEQ-------LTPELKSSILKCVQSTKPSLMIQKAAIQALRKMEP

KDKDQ-EVLLQTFLDDASPGDKRLAAYLMLMR-SPSQADINKIVQILPWEQNEQVKNFVA

SHIANILNSEELDIQDLKKLVKEALKESQLPTV-MDFRKFSRNYQLYKSVSLP-SLDPAS

AKIEGNLIFDPNNYLPKESMLKTTLTAFGFASADLIEIGLEGKGFEPTLEALFGKQGFFP

DSVNKALY-WVNGQVPDGVSKVLVDHFG-------------YTKDDKHEQDMVN----GI

MLSVEKLIKDLKSK------------EVPEARAYLRILGEELGFASLHDLQLLGKLLLMG

ARTLQGIPQMIGEVIRKGSKNDFFLHYIFMENAFELPTGAGLQLQISSSG--VIAPGAKA

GVKLEVAN-MQAELVAKPSVSVEFVTNMGIIIPDFARSGVQMNTNFFHESGLEAHVALKA

GKLKFIIPSPKRPVKLLSGGNTLHLVSTT-KTEVIPPLIENRQSWSVCKQV--FPGLNYC

TSGAYSNASSTDSASYYPLTGDTRLELELRPTGE-IEQYSVSATYELQREDRALVDTLKF

VTQAEGAKQTEATMTFKYNRQSMTLSSEVQIPDFDVDLGTILRVNDESTEGKTSYRLTLD

IQNKKITEVALMGHLSCDTKEERKIKGVISIPRLQAEARSEILAHWSPAKLLLQMDSSAT

AYGSTVSKRVAWHYDEEKIEFEWNTGTNVDTKKMTSNFPVDLSDYPKSLHMYANRLLDHR

VPQTDMTFRHVGSKLIVAMSSWLQKASGSLPYTQTLQDHLNSLKEFNLQNMGLPDFHIPE

NLFLKSDGRVKYTLNKNSLKIEIPLPFGGKSSRDLKMLETVRTPALHFKSVGFHLPSREF

QVPTFTIPKLYQLQVPLLGVLDLSTNVYSNLYNWSASYSGGNTSTDHFSLRARYHMKADS

VVDLLSYNVQGSGETTYDHKNTFTLSCDGSLRHKFLDSNIKFSHVEKLGNNPVSKGLLIF

DASSSWGPQMSASVHLDSKKKQHLFVKEVKIDGQFRVSSFYAKGTYGLSCQRDPNTGRLN

GESNLRFNSSYLQGTNQITGRYEDGTLSLTSTSDLQSGIIKNTASLKYENYELTLKSDTN

GKYKNFATSNKMDMTFSKQNALLRSEYQADYESLRFFSLLSGSLNSHGLELNADILGTDK

INSGAHKATLRIGQDGISTSATTNLKCSLLVLENELNAELGLSGASMKLTTNGRFREHNA

KFSLDGKAALTELSLGSAYQAMILGVDSKNIFNFKVSQEGLKLSNDMMGSYAEMKFDHTN

SLNIAGLSLDFSSKLDNIYSSDKFYKQTVNLQLQPYSLVTTLNSDLKYNALDLTNNGKLR

LEPLKLHVAGNLKGAYQNNEIKHIYAISSAAL-SASYKADTVAKVQGVEFSHRLNTDIAG

LASAIDMSTNYNSDSLHFSNVFRSVMAPFTMTIDAHTNGNGKLALWGEHTGQLYSKFLLK

AEPLAFTFSHDYKGSTSHHLVSRKSISAALEHKVSALLTPAEQTGTWKLKTQFNNNEYSQ

DLDAYNTKDKIGVELTGRTLADLTLLDSPIKVPLLLSEPINIIDALEMRDAVEKPQEFTI

VAFVKYDKNQDVHSINLPFFETLQEYFERNRQTIIVVLENVQRNLKHINIDQFVRKYRAA

LGKLPQQANDYLNSFNWERQVSHAKEKLTALTKKYRITENDIQIALDDAKINFNEKLSQL

QTYMIQFDQYIKDSYDLHDLKIAIANIIDEIIEKLKSLDEHYHIRVNLVKTIHDLHLFIE

NIDFNKSGSSTASWIQNVDTKYQIRIQIQEKLQQLKRHIQNIDIQHLAGKLKQHIEAIDV

RVLLDQLGTTISFERINDVLEHVKHFVINLIGDFEVAEKINAFRAKVHELIERYEVDQQI

QVLMDKLVELAHQYKLKETIQKLSNVLQQVKIKDYFEKLVGFIDDAVKKLNELSFKTFIE

DVNKFLDMLIKKLKSFDYHQFVDETNDKIREVTQRLNGEIQALELPQKAEALKLFLEETK

ATVAVYLESLQDTKITLIINWLQEALSSASLAHMKAKFRETLEDTRDRMYQMDIQQELQR

YLSLVGQVYSTLVTYISDWWTLAAKNLTDFAEQYSIQDWAKRMKALVEQGFTVPEIKTIL

GTMPAFEVSLQALQKATFQTPDFIVPLTDLRIPSVQINFKDLKNIKIPSRFSTPEFTILN

TFHIPSFTIDFVEMKVKIIRTIDQMLNSELQWPVPDIYLRDLKVEDIPLARITLPDFRLP

EIAIPEFIIPTLNLNDFQVPDLHIPEFQLPHISHTIEVPTFGKLYSILKIQSPLFTLDAN

ADIGNGTTSANEAGIAASITAKGESKLEVLNFDFQANAQLSNPKINPLALKESVKFSSKY

LRTEHGSEMLFFGNAIEGKSNTVASLHTEKNTLELSNGVIVKINNQLTLDSNTKYFHKLN

IPKLDFSSQADLRNEIKTLLKAGHIAWTSSGKGSWKWACPRFSDEGTHESQISFTIEGPL

TSFGLSNKINSKHLRVNQNLVYESGSLNFSKLEIQSQVDSQHVGHSVLTAKGMALFGEGK

AEFTGRHDAHLNGKVIGTLKNSLFFSAQPFEITASTNNEGNLKVRFPLRLTGKIDFLNNY

ALFLSPSAQQASWQVSARFNQYKYNQNFSAGNNENIMEAHVGINGEANLDFLNIPLTIPE

MRLPYTIITTPPLKDFSLWEKTGLKEFLKTTKQSFDLSVKAQYKKNKHRHSITNPLAVLC

EFISQSIKSFDRHFEKNRNNALDFVTKSYNETKIKFDKYKAEKSHDELPRTFQIPGYTVP

VVNVEVSPFTIEMSAFGYVFPKAVSMPSFSILGSDVRVPSYTLILPSLELPVLHVPRN-L

KLSLPDFKELCTISHIFIPAMGNITYDFSFKSSVITLNTNAELFNQSDIVAHLLSSSSSV

IDALQYKLEGTTRLTRKRGLKLATALSLSNKFVEGSHNSTVSLTTKNMEVSVATTTKAQI

PILRMNFKQELNGNTKSKPTVSSSMEFKYDFNSSMLYSTAKGAVDHKLSLESLTSYFSIE

SSTKGDVKGSVLSREYSGTIASEANTYLNSKSTRSSVKLQGTSKIDDIWNLEVKENFAGE

ATLQRIYSLWEHSTKNHLQLEGLFFTNGEHTSKATLELSPWQMSALVQVHASQPSSFHDF

PDLGQEVALNANTKNQKIRWKNEVRIHSGSFQSQVELSNDQEKAHLDIAGSLEGHLRFLK

NIILPVYDKSLWDFLKLDVTTSIGRRQHLRVSTAFVYTKNPNGYSFSIPVKVLADKFIIP

GLKLNDLNSVLVMPTFHVPFTDLQVPSCKLDFREIQIYKKLRTSSFALNLPTLPEVKFPE

VDVLTKYSQPEDSLIPFFEITVPESQLTVSQFTLPKSVSDGIAALDLNAVANKIADFELP

TIIVPEQTIEIPSIKFSVPAGIVIPSFQALTARFEVDSPVYNATWSASLKNKADYVETVL

DSTCSSTVQFLEYELNVLGTHKIEDGTLASKTKGTFAHRDFSAEYEEDGKYEGLQEWEGK

AHLNIKSPAFTDLHLRYQKDKKGISTSAASPAVGTVGMDMDED-DDFSKWNFYYSPQSSP

DKKLTIFKTELR--VRESDEETQIKVNWEEEAASGLLTSLKDNVPKATGVLYDYVNKYHW

EHTGLTLREVSSKLRRNLQNNAEWVYQGAIRQIDDID--VRFQKAASGTTGTYQEWKDKA

QNLYQELLTQEGQASFQGLKDNVFDG-LVRVTQEFHMKVKHLIDSLIDFLNFPRFQFPGK

PGIYTREELCTMFIREVGTVLSQVYSKVHNGSEILFSYFQDLV----------ITLPFEL

RKHKLIDVISMYRELLKDLSKEAQEVFKAIQSLKTTEVLRNLQDLLQFIFQLIEDNIKQL

KEMKFTYLINYIQDEINTIFSDYIPYVFKLLKENLCLNLHKFNEFIQNELQEASQELQQI

HQYIMALREEYFDPSIVGWTVKYYELEEKIVSLIKNLLVALKDFHSEYIVSASNFTSQLS

SQVEQFLHRNIQEYLSILTDPDGKGKEKIAELSATAQEIIKSQAIATKKIISDYHQQFRY

KLQDFSDQLSDYYEKFIAESKRLIDLSIQNYHTFLIYITELLKKLQSTTVM--NPYMKLA

PGELTIIL

>H glaber

MGARAAAQRVRVRARPQGTGMGRPRPALLLLLLL---LLLLDAGARAEDEAPERESVSPS

CPKEATRFKHLRKYIYSYEAETSSGVPGTADSRSATKVSCKVELEVPQLCSFILKTSQCA

LKEVYGFNPEGRVLLKKTKTSEAFAAAMSRYELRLAIPEGKQAVLYPEENEPAHILNIKR

GIISALLVPPETEEAQQELFLD-TVYGNCSTHTAVKTRKGSV-ATEMSTERNLQQCSGFQ

PASTGASPLALIKGLARPLSTL---ISSSQSCQYTLDPKRKHVSEATCKEQHLFLPFSYK

NKYGMMTQVTQTLKLEDTPKINSRFFGGGTQK---VGLAFESTKSPSLPKQ--AEAVLKT

LQELKKLSTSEQNAQRANLFNKLVTNMRGLSSEAITSLL----------------PQLIE

VSSPITLQALVQCGQPQCYTHVLQWLKSEKAHPLLIDAVTYLVALIPDPSVQRLREIFSI

AKEQQSRATLYALSHVANNYF------EATRTVDPDLQDIINYLQEQIQDQCA---GDED

HAYLILRVIGNMGRTLES-------IAPMIKSSVLSCIRSPKGSVLIQKAAIQALRKMEL

GYEDR-ELLLQTFADSTSPVEKRLAAYLMLMK-QPSQSDISSVIQLLPQEESEQVKNFVA

SHVANILNSEELYVGDLKTLVKEALKDSQLPTV-MDFRKYSRNYHVSKSVSLP-SLDPVL

AKIEGNLIFDPNNYLPKESMLKTTLTIFGFASADLFEIGLEGKGFEPTLEALFGKQGFFP

DSVSKALY-WVNGQVPDRVSQVLVDHFG-------------YTKDDKHEQDMVS----GI

MPIVDKLIRDLQSR------------DMPEARAYLRILGRELGFFKLQDLQLLGKLLLSG

ARTLQGIPQMISEAIREGSEHDLFLHYIFMDNTFELPTGVGLQLQVSSSG--IISPGSKA

GLKLQVAN-MQADLLMKPAVSVEFVTNMGVIVPDFARSGVQMNTNFFHESGLEARVALKA

GQLKVIIPSPKRPVKLFSGSNTLHLISTT-KTEVIPPLMENRQSWSACRPF--LTGLHYC

TAGAYSNASSTESASYYPLTGDTRYELELKPTGE-VEQYSASATYELQREDKALVDRLKF

VIQAEGVKQAEATLMFKYNRRSRTLSTEMQIPDLDVNLGTILRVSDESSKDKRSYKLNLD

IQNKKITEVALSGHISHDTKGEGKIKGVISIPRLQAEARSEILSHWSPTKLLLQMDSSAT

AYGSTVSKRVAWRYDDEKVEFEWNTGTNVDTKKVASNFPVDLSDYLRSLYMHANGLLHHR

IPQTDMSFWDMGSKVIVTTNTWLQEASSSLPYAQILQDHLSSLTELDLQKMSLPYFHIPD

NLFLKSDGRVKYTLNKNSIEVDIPLPFGGKSSKDLKMFDTIRTPALNFKSVGFYLPSREF

HIPTFTIPELYQLRVPLLGVLDLSTNVYSNLYNWSASYTGGNTSRDHFTLQARYHIKADS

AVNLLSYSLQGSGETTYDHRNTFTLSCDGSLRHKFLDSNVKFSHVEKLGNNPASKGVLTF

DASTTWGPQVSASVHLDSKKKQHLYVRDVKIDGQFGASSFYAKGTYSLTCQGDPATGQLS

GESDLSFNSSYLQGTNQITGRYEDGALSLTSTSDLQGGTLKNTASLKYENYELTLKSDTN

GKSENFAIRNRVDLTFSKQNALLHSEYEADYKSLKFFSLLSGSLNSHGVELNADILGTDK

VNTAAHKATLSIGRSGISTSATTNLKYSPLFLENELNAELGLSGASMKFTANGRFREHST

KFTLDGKAALTEVSLGSVCQAMVLGVDSKNIFNFRISGEGLKLSNDLKGSYGEITLDHAN

SLNTAGLSLEFSSKLDNIYSSDKFYKQHFNFQLQPFSLVTTLNNDLKYSALGLTSSGKLR

LEPLKLNVDGNLKGAYQNNEIKHIFTISYVDI-SASYKASTIARIQGMQFSHQLNTGIAG

LVASLDTSTNYHSDSLRFSSALHSAVAPFALAINAHTNGNGKLALWGEHTGQLYSKFLLK

AEPLALTFSHDYKGSTSHSLLSRESIGMALDHKLSALLTPSEQTGTWKLKTQLNSNEYSQ

DFDAYNTKDKIGVELSGRGLADLTVLDSPIKVPFFLSEPINIIDTFRMKDTVQQPQEFTI

AAAIKYDKNQDVHTISLPFFKSLPEYLEKNRLMIITALEAMQKELKSLNIDQFVGRYRAG

LGKLPQQVNDYLTAFNWESQVASGKEKLTAFAENYRITENDVQTALNNAKISLNENLSQL

QTYVIQFDQYIKDNYDLHDLKIAIVRIIDRIIENLKILDEHYHIRENLVRTIHNAYLFIE

NIDFSKMGSSTASWIQNVDTQNQIRIQIQEKLQQARTQIQNIDIQELAAKLKQQVEAIDT

RALLNQVRTTIPFQRIKDIIEHVKYIVISLIEDFEVTEKINAFRAIAHELIKKYEVDQQI

QVLLDKSVQLARQYKLKDTVQKLSNLLQRVDMKDCYKKLLVFIDDIIRQLKAFSFKNFME

EVNQFLDALVKKLRSFDYHQFVDETNHKIREVTQIINAELQVLDLPQKAEALKLFVEDVR

VMLSAFLEKLKDTKITVFVDWLQDALNTAYLVPIKAKFQETLEDIRDRIYQMDIQQELER

CLSLVGQVYSTLVTYVSDWWVLAAKNLTDFAEQYSIQGWAENVKALVEQGFTVPRIQLIL

GTVPAFEVSLRALQEASFQTPIFTVPLTDLRIPSVRINFKELKDIKIPPRFSTPEFTVLN

TFHIPSFTVDFLEIKLKIIRTIDQMLSSEFQWPVPEVYVGDLKVEDILPARLFLPNFHLP

EITIPEFLIPNLNLTEFQVPDLHIPEFQLPPISHSAEIPALGKLNSILKIQSPLFTLDAK

ADIHNITTSEHQTGTEVSIAAKGESKFEVFNFDFQANAQLLDPKTNPLLLKESMNFSSKH

LRMEHKSEILFSINAIEGKSDTVASFSTEKNTLELNNGVVVKMKGQLTLDNHMKYFHKLS

IPKLDFSSKADLRNDLKTLLKVGHITLTSSGAGSWKWACPSFSDEGTHESQINFTVEGPL

ASLGLANKINSKHLRVTQNLAYESHFLNFSKFEIRSDVESQHVGHSILTAKGTALLGKGK

AEITGDHSAHLSGKVIGTLKNSLFISAQPLEITASTNNEGNLKVSFPLRLTGKIDFLNNY

ALLLSPSAHQTSWQASAGFNQYKYNHNFSASNNENSIDAHVSINGDANLDFLNIPLTIPE

MNLPYTTFKTPSLKDFSIWEETGLKEFLKTTKQSFDLNVKIQYKKNKDKHTIAVPLGVFY

KFISQNVNSLDSYFENVRDNALDFLTTSYNELKIKLDEYKAERSLNKLPRTFPRPGYTIP

IVNVEVTPFSVEMLPSGHMIPKAVHTPSFTIPGFDFYVPSYMLSPPSIELPDLHIPRNLL

KLSLPDFKGLSTVNNIFIPAMGNITYDFSFKSSVITLNTNAGLYNQSDIVAHFLSSSSFV

IDALQYKLEGTSRLTRKRGLKLATALSLSNKFVEGSHDSTISITKKIMEASATTTAKVHI

PVLRMNFKQELNGNTKSKPTVSSSIELTYDFNSPKLFSSAKGAIDHKLSLESLTSYFSIE

SSTKGDIEGLVLSREYSGAIASEASTYLSSKATRSSVKLQGSSKVDGIWNVEVKENFAGE

ATLRRMYAMWEHSMKNHLQPYSGFSTHGEHTSKATLELSPGSVSALIQVRAREINSVLDI

PHLGQEVALNANTKNQKFSWKSEVQGQLGTLQNHVQLSNDQKEARFDIAGSLEGRLHFLK

FITLPVYDKSLWDLLKLDVTTSSQKRQYLHASTALVYTKNPKGYPFSVPVQELTDEFIVP

GLKLSDLSSDFATPTFRVPFTDLQIPSYKLDFNEIRIYKKLSTSPFALNLPTLPKVKFPQ

VDVLTKYSEPEDSSVPFFEITIPDVQLTVSQFTLPKSLSVGSAVLDLSEVANMIGDFELP

TITVPEQTIEIPSMKFSVPAGVFIPFFGALTARFGVASPLYNATWNVGLKNKADHVETFL

DSTCSSTLQFLEYDLNVVGTHSLEDGMLICKIKGTLAHRDFSAEYKEVDKYKGLKAWEED

VNLDILSPAFTELHLRYREDENSMSFSAASPAVGTVGLDLETEGDRSFKGNLYFQPQSSP

GRKLNIFKTEWR--YQESDDKIQMKFNWEEEAASRFLGSLKDNVPKATGAFYDYVDKYHQ

EYTGLSLKDASSKLRRSLRESAEGAYQRARRQINKMD--VGLQRAASSTIGTYQEWKEKA

QDLYQELLAEEGQASSQRLKDKVFDS-LFQATQEARMAAKCMMDSSIGFLQLTRIQLPGI

AGTYTGEELCTMVIKEIGKVLSWAYSNIHSELEKLLSYAQDLVEKSELIKDLQITFPFYS

ESYKLTEVILKSRKLLEFLSKNVQDVFIDLQSIKLSETLGDLQSSLQIIFERIEEEIKYL

KETRFIHPIN----AIDTIFNTYIPFAFRVLKESLYLNLTKFNEAVQNNLQVASQGLQQT

HQYLKALREEYFDPSIVGWTVKYYALEEKMVDLVMDLVIALKNLHSTYTAYAASSASQLS

ALVEQFVHKDVQEYLSILADADRRGKEKIAELSNSAQQVIKTLTTAMKEIVSDYLEQFIS

KLQEFLDQFPDYYEKFIAESQRLIDLAIQSYHVILSYITELLKKLQAATASGGNPYVKLA

PGELTITY

>M brandtii

--------------------MGPSGPALLALL-----LLMITAGARAEDEVL--ENVNPS

CSKGTTRFKHLRKYVYNYEAESSSGAPASTDSKSVTKINCKVELEVPQLCSFILKTSQCT

LKEVYGFSPEGQALLKKAKNSEEFSAAMAKFELRLATPEGKQVLLYPDKEEPKHILNIKR

GIISALLVPPETEEDKQMLFLD-TVYGNCSSDVTVKTRKGNV-ATEISIERNLEKCDHFK

PVSTGVSPLALIKGLTQPLSTK---ISSSQSCQYTLDPKRKHVSEAICQEQHLFLPFSHK

DKYGMMALVTQTLKLEDTPKINSRFFDEGTEE---VGLTFETTKSTSPPKQ--AEAIVKT

LQELQKLHVSEQNAQRANLFSRLVTELRGLNNEAVASLL----------------PKLGE

VSSPITLQALIQCGQPQCYTHILQWLRNEKANPLLIDVVTYLVALIPGPSAQRLQEIFNT

AKEQQSRATFYALSHVIHNYH------KTHPTATQELLDIADYLLEQIQDDCT---GNED

HTYLSLRVIGNIGGTLQK-------LTPRLTSSVLKCIKSTAPSLLIQKAAIQALRKMEN

KDEVR-EVLLQTFLDEAAPGDKRLAAYLMLMR-DPSQSDISKVTQLLPGEQNEQVKNYVA

SHVANILNSDELYVQSLKELVKEALKDSQLPTV-MDFRKFSRNYHFSKSVSLP-SLTPFS

TKLEGNLIFDPDNYLPKESMLKTTLTVFGFESADLFELGLEGKGFEPTLEAFFGKQGFFP

DSVNKALY-WVDGKVPDRVSEVLEDHFG-------------YTKDDKREQDMVK----GI

MGNVEKLIKDLKSK------------DFPEARAYLRILGEELGFVKLDDLQLLGRLLLNG

VRTLKGIPHMVAEALKKGSKNDLFLHYIFMDNAFELPTGLGLPLQVSSSG--VLAPGIQA

GVKLEVKDKMQMELVTKPSVSVEFVTNMGIVIPDFARNGVQMNTNFFHETGLEARVGLNL

GHLKFSIPAPKRPVKLFSVSNTMHLVSTT-KTELIPPLKENRNSWSTCKPF--FTGLNYC

TTGTYSNTS-----SYSPLTGDTRYELELRPTGE-VEQYSASATYNLQKEGKARVDTLEF

VAQAEGVKQTEATMTIKYNRQTMTLTSETQIPDFGIDLGTVLRVSDESAEEKTSYKLTLD

IQNMKITEVAFTGHISYDKKEEAKIKGVLSVPRLQAEARSETLAQWSPTKLLLQMDSSAT

AYGSTISKKLAWRYDEEKIEFEWNAGTDVDTKKAASSFPVDLSDYPKSLHMYTNRLLDHR

VPKTDMTFRHMGSKLMVATNTWFQKAFASLPYTQNLQEY---LKQLNLQKMELPEFHIPE

NLFLKSDGRVKYTFNKNSVKIEIPLPLGGKSSKDLKMLETVRTPVISLPFLGFYQPPQEF

QLPNFTIPKLYELRVPHLGVLDLSTNIYSNLYNWSASYTGGNTSTDHFSLQARYHMKADS

AVDLLSYSVQGSGETTYDHRNTFTLSCGGSLHHKLLDSNVKFSHVEKMGNNPASKGLLTF

DASSPWGPQMSASVHLDSKQKEQLYIKEVKIDGQFRVSSFYANGAYGLSYQKDPASGQRT

GESNLRFNSTYLQGTNQIIGRYEDGTFSLTSSSDLQDGIIKNTASLKYENYELTVKSDTN

GQYQDFTTSNKVELAFSTQKASLSSVYQADYKSLRFFTQLSGSMNPHGLELNAEILGTDK

INTGAHKATLRIARDGMSTSATTNLKYSPLMLENELNAALGLSGASVKLTTNGRFREHNA

KFSLDGKAALTEVSLGSAYQAMILGVDSKNIFNFKINQEGLKLSNDMMGSYAEMKLHHTH

SLNMAGLSLDFSSKLDNIYSSDKSYKQTFNLQLQPYSLVTTLNNDLEFNDLDLTNHAKLR

LEPLKLNVGGNIKGAYRNDEIKHIYTLSYADL-SASYKADTVAKVQNTEFSHRLSTNIAG

LASTTDISTSYNSDSLHFSNVFHSVMAPFTVTVDVHTNSNGKLVLWGEHTGQLYSKFLLK

AEPLALTFSHDYKGSTSHHLMSKRSVSTALDNKVGALLTPAEQTATWKLKTQVNKNEYTQ

EFDAYNTKDKVGMDLTGRALADLTMLDSPIEVPFLVRDPVNVIDALEIKDTIDQPQEFTM

VASVKYDKNQDVHTINLPFFNLLPEHFEKSRIIIITALEAIQRELKHIDIDQFVMKYRAA

LDKFSEKVNVQLNEVNWETQVSRAKEKLTAFIKNYGITENDLKMALDNAQINFNETLSQL

QTYVIQVDQYIN-----YDVKAAIAKIIDQIIEKLNILDKHYRIRVTLVKVIHNLHLFIE

KVDFNKVGSNTVSWIQNVTDRYQIRIQVQEKLQQLKTQIQNFDMQHLAEKLKQQIEAIDV

RVLLDQLRAAISFQKIKQMIEHVKNYVGKLIEDFEVTKKINAFRTMVHKLINRYKIEQHI

QILIDKSVQLAQKYKLKETLQKLGNVLKQVGIKDHFEKLVGFIDDAVTQLKALSFKNFIE

EVNRFLDMLISKLRSFDYHRFIDETNNKIREVTRIINEEIQALDLPQKAKALKLFVEDIK

AAVSVHLENLNSTKITLFIDWLQEALRSPSLNHMKAKFQETLEYIRDQMYKMDLQQKVQQ

CLLQVGEIYNTIVTYISDLWSLAVKNLTDFAEQYSMQDWAENLKALVEQGFIVPEIQTIF

VTIPSFEVSLRALQEATYQTPEFIVPLTDLKVPSVQINFKKLEDIKVPSRFSTPEFTVLN

TFHIPSFTIDLVEIKAKIIRIIDQMLNSELQWPVPEVYLKDLMDTDTILARITLPDFHVS

EITIPEFVIPKLDLKAFQIPDLHIPEFQLPKISQTIEVPTFGKLHSVLKIQSPLFTLDAN

ADIHNVTTSVNEAGITASITAKGHSKFEVLKFDLQANAQLSYPKMKPLVLKESVSFSSKY

LNTEHKSEVLFFENTFEGKSNTVAGLQTEKNTMELSNGVLIKINNQITLDSNTKYFHKLN

IPKLDFSSQADLHDEIKTLWDSEKIAWTSSGRAAWKLACPKFSDEGTRESQISFTMRGLL

TSFEFSNKINSKHLRVNQTLAYESGFLNYYQLEIQSQVESQHVGHSILTAKGTAQLSERK

VEVTGNHDAQLNGKVSGNLKNSLFFSAIPFEITASTNNEGNLKVIFPLKLTGKIDFLNNY

ALFLSPNAQQASWQAGARFNQYKYNQNFSAGNNENSTDAHIGISGEANLDFLNSPLTIPE

MTLPYTGFKIPQVKDFSLWEKTGLKEFLKTTKQSFDLSVKAQYKKNKDKHSIPIPLDVFY

VFINQNINSFNRHFEKVRGKALNLFTKSYNEAKIKLNKYMVEKSLNKEPMTLQIPGYTIP

VVNTELSPFTAKMWTFGF----EIRTPNLTTPDSGFYVPSYAIVLPSLELPALNVPRNFL

KFSLPELKVLS-IPNILIPAMGNITYDFSFKSSVITLNTNVGLYNQSDIVAHFLSSSSSV

IDALQYKLEGSSSLTRKRGLKLATALSLSNKYVEGNHDSTISLTKKTMEASVTTTANVQI

PILRMNFKQELNGNTKSKPTVSSSIELKYDFNSTKLYSTATGTVNHKFSLESLTSYFSIE

LSNKGDIKGSVLSREYSGMIASEVSTYLNSKGTRSSVKLQGASKVDRLWNLEVKESFAGE

ASLQRIYATWEHNTKNHVQVL-YPLTSGEHSGKATLELSPWKMSALAQVHASQLSSFLDL

NHLGQEMSLNVNPENQKASWKGEVQVHSLSLQNDVQLSNDQDETRLDIAGSLEGYLKFLD

SIALPVYDKTLWEFFKLDVTTSKDRKQYFHASTALVYTKNPKGYTFSVPVQELADKFVIP

GLRLNNLNSVLLTPAFQVPFTDLQVPSYTLDFSEIKIYKKLSTSPFSLNVPALPKVKFPE

VDVLTKYSGPEDSSVPFFEITVPGFQLTMPQFTYPNSISVGATVLDLNEMAGKIADFRLP

NITVPDQTIEIPSIKFSIPSGIYIPSFGALTARFGVASPLYNATWNAGLKNKEDHVETYL

DSTCSSIIKFLEYDLKGIGTYKIEDGMFVYKAKGTFTHRDISVEYKEDAKLQEYPDWKVE

VLLDITSPVFTDVHIHCHAREN-VFYSAASPAIGTVVLDLGSD-NTTITYNFYYRPQSSP

DKNLSIFKIEYRHPYSESNEKFQIKVNWEKEAVSQLLSSLKDNVPKATGALYDYVNKCHQ

EYTGLNLREASLKLRRGLQNKAEETYQGALILIDKVNKTLEFKRVARGAARTYQLWNDKV

QILYQDLLDQEGHIDLQRLQNKVLNI-LINITREYETIVRHVIAWFIDYLQLTRLQLPGR

ASTNTADELSTLVMRGVGKILLQVHLNIHNGLEILFSYVQGLMEKSELIKDLKIKFPFDS

RSLKLTNITLEYGKQLNSFSQMVQRVLNALKSNKTTVILGDLQRYLEGVFEYIREEINDL

KMKRLTVIKH----KIKVIFDSCIQYVSQFLKENLGLDVGKLHELVQNKFQEASQELEQL

HQYIKALRKEYLDSSVVSWTLKYYEYEEKVINLVKKLVHALKYFHSKYIVNAAGIASQLS

NEAEQLVQQAIPKYLSILVDADGKGKEKIVELFTSAKEIIKSWTAAMKEIISDYHQQFKY

IPQNFSDRLFDYYEKFIAESQRLIDLSIQTYHMFLIYITGLLKELQSTTVYDLSRYLKIA

PGELTITF

>S franciscanus

-------------------HILNAKRGILSMI----------------------------

----------------------------------------QLDLEADD-------VDQIT

L------------------------EEMSAHDLVFRTENGRIVEVLAHPDEPLHILNAKR

GILSMIQLDLEADDVDQITLNEVSVHGNCSSEMTVTRRDDKQRPRKLQVVTDLNNC-MLR

KQPENMTRWQSFKDIVSNMVIMDKVINSSKTCSYDLKGTTGEFRGIQCEEVHSFWPMNFN

SESGVRTFIQQSLISTIIAPLNSFKVDPAVSRSTCLRYEHDSKEIGKKPDDITADQAMDI

LKALVKSSREEVKMESPRLFGRWRAALEGMNNDTIHEVFDRAYNCSAYSQTCRDNPDKEK

IAREYLLDGLLPCTTMACFSVKNYAIAKGVVPKPLASAWMVTMALQNYPTLELMSEMLRM

AQYSPSQITIFPLSIMVHNYWMNNEEIHTADTVPEPIVKTIRYLKSLIGNDCTVSAEKER

LALVAIKAIGNIGEPVERYDQLTFIFSERLSPVLISCTKNKQIPRDISKAAIQSMRRFVM

SDQLRSQVISELFKDTTLDVPQRIAAYLITMKANPSVSELTDIWNFLKTEPVNQMKAFCY

SHIQNIIESSEPTLQEVLFLI-ISLKLKLLQEYPKDIRKYSRFFELSKNLTEPWRNNTVA

AQLESDIIFSPESYLPQEVMFHYVLNALG-KSWDIFE-------FEPIIESVFGPEGFFP

EVKLQTMFDTIDEKFLSKVREFAEAQLGNWVTPTSEPDNEVFNKKGKYGKKMKRQSPIKC

IQVIDVIHENTMNKLNQLHYLVGMEPSATDATVFLRLLGNEFGFVSASDVLALIPYTMGI

YEELMNKTKQVPQLLSQGLNLNTTRSLVFMDKSFILATGMGMPLNCSINGTAVVSLRMKS

QFSINLPKIIAAGYVA-PSGAVEVLATMGIGLPTKVFTGVMANATVYHSSKIAGNITIDG

THLKINLNNTDRPMNLFNYSTTYNLLKANGDIEFIPGVLQDAVQSQRCTNVSKVFGVDLC

VSWAYPNASYVAEAPRFPFSGPNSFNVTLVNYFRALKAYTIDAQYKQIREGGA-------

------------------------------------------------------------

------------------------------------------------------------

--------------------------------------PAPIS-----------------

-------FSSIGRR----------------------------------NKQNAPAFT---

-----------------------------------KLIDTVR--------VSFYAPGNK-

----------------------LTRNITSLV------------SVDRLRKTAKW------

---------------------TFTV--------------------------PEVKNFIMF

------------------------------------------------------------

------------------------------------AGILNETTDS--------------

------------------------------------------------------------

------------------------------------------------------------

------------------------------------------------------------

------------------------------------------------------------

---------------------------------SSSYRA---------------------

--------------------VFNVSAAENTASVD----------------------FLVR

NE----------------TSLAQKNLTAVFNASINSL-----------------------

------------------------------------------------------------

--------------------------------------------------------YWAA

VGQFVKQ-----------------------------------------------------

------------------------------------------------------------

--------SNNGDWNAHLTDTY--------------------------------------

------------------------------------------------------------

-----------------------------------------YWDNETPLYKQLLFP----

------------------------------------------------------------

--------------------WTGYQIN-------HAKYQTS-------------------

--------------------TITARRIT--------------------RG----------

--------------------------------PVVQRDLKM-------------------

---------------------------GILDWA---------------------------

------------------------------------------------------------

------------------------------------------------------------

------------------------------------------------------------

---FDFNTVQTVTDELKELTVHTQISNAT-----WVF---------------------PL

LDVTLRNETKVNGYRLEGQVARNTHHI-FAEYEVTKE-----------------------

------------------------------------------------------------

--------------------PRETNRNFRMG-----LRTDVLVNNPDASMFLNSPVLL--

----------------ELFGETSLID-----------------------EPVVIDGGANF

RILDQS------------------------------------------------------

------------SLTEEHLIQTSIFVPN---------------------------PRN--

-------------------PQTHLTYDFDLRLE------NATISNQKD------------

-----YSLELTT----------------------------------------------PI

ILMQMN------------------------------------------NVNFLHLMIDME

S---------------------------------------------------------GT

STLDHTYTLYD-------------------------------------------------

------------------------------------------------------------

------------------------------------------------------------

------------------------------------------------------------

----TRYSKP--------------------QVTVSNSIGAGWK----KPSANWVMDIATP

SM-------------------VYFQNYTAL------------------------------

--------------------YSSENGL------------QFFYENSQDSSVYDLLDLILT

DNLSVK------------------------------------------------------

--------------------------------------------PSAVSMYTSYIHQL--

---------ISTNVSNSLVVDGPVTEMRPRL----------------------------V

PMIVRDILNKTFRRDFSSVKDLVLNKFIFNVTS---------------------------

---------STITLRQSANMSSPIIANMSASHYIFLS--QSLFDQ---------KLSFAA

EQKRL-----------------------------------NISAGLNHSLLIEDREITLK

TEGDVAV-------RTGLRMSTLTSVNVTISKPDTPFDLAALH-----------------

----------------------YTEVNSPLIR----------------------FSTRSS

S------NGNFHEVLNMAANHN--------------------------------------

--------------------------------------TSMTSRL---------------

-GSFDVIG

>S purpuratus

---------------------MRAVSFLLVSLLLATAVVAGPFGTRFGQEV----EPELE

CS-ELTRFKEGYSYTFNYEAETESGIKGAGPGSTST-MTCNIAIEAPERCRNVLKVTSCS

L-----------------DGNPQLGDEMSAHDVVYRTDKGRIIEVVAHPDEPIHILNAKR

GILSMIQLDLEADDVDQITLNEVSVHGNCSSEMTVTKRDDRQRPRKLQVVTDLNNC-MLR

KRAENMTRWQSFKDIVLNMTVMDKFINSSKTCSYDLKGTTGEFRGIECEEVHSFWPMNFN

SDSGVRTYIQQRLISNTIENLNSFKVDPAISRSTCLSYELDPKDVGKKPDDVTADQAMDI

LKALVKSSREEVRMESPRLFGRWRAALEGMNNETIHEVFDRAYNCSAYSQTCRDNPDKEK

IAREYLLDGLLPCTTMACFSVKNYAIAKGVVPKPLASAWMVTMALQNYPTLELMSEMLRM

AQYSPSQITIFPLSIMVHNYWMNNEEIHTAATLPEPIIKTIRYLKSLVGNDCTVSAENER

LALVALKAIGNIGEPVERYDQLTFIFSERVSPVLISCTKNKQIPRDISKAAIQSMRRFVM

SDQLRSQVISELFKDTTLDVPQRIAAYLITMKANPSVSELTGLWSFLKTEPVNQMKAFCY

SHIQNVVESSEPTLQELKKNLKIAMIGEELPEYPKDIRKYSRFFELSKNLTEPWRNNTVA

AQLESDVIFSPESYLPQEIMFHYVLNALG-KSWDIFETGIEMKGFEPIIESVFGPEGFFP

EVKLQTMFETIDEKILSKVREFAEAQLGNWVTPTSEPDNEVYNKQGKYGKKMKRQSSFGE

PQVVDMIHENTMNKLNQLHYLVGMEPSATDATVFLRLLGNEFGFVSASDVLALIPYTMGI

YEELLNKTKQIPQLLSQGLNLNTTRSFVFMDKSFIVPTGMGMPLNCSINGTAVVSLRMKS

QFSINLPKIIAAGHVA-PSGAVEVLATMGIGLPTKVFTGVMANATVYHSSMLAGNITIDG

THLKINLNNTDRPMNLFNYSTTYNLLKANGDIEFIPGVLQDAVRSQRCTNVSKVFGVDLC

VSWAYPNASYVAKAPRFPFSGPNSFNVTLINTDQALKAYTIDAQYKQIREGGA-------

------------------------------------------------------------

------------------------------------------------------------

--------------------------------------PAPMS-----------------

-------FKSIGKR----------------------------------SKQNAPAFT---

-----------------------------------KLIDTVR--------VSFYAPGEE-

----------------------LTRNITSLV------------SVDRLRKTAKW------

---------------------TFTV--------------------------PEVKNFIMF

------------------------------------------------------------

------------------------------------AGILNETTDS--------------

------------------------------------------------------------

------------------------------------------------------------

------------------------------------------------------------

------------------------------------------------------------

---------------------------------SSSYRA---------------------

--------------------VFNVSAAENTASVD----------------------FLVR

NE----------------TSLAQKNLTAVFNASINSL-----------------------

------------------------------------------------------------

--------------------------------------------------------YWAA

VGQFFKP-----------------------------------------------------

------------------------------------------------------------

--------SNNGDWNAHLTDTY--------------------------------------

------------------------------------------------------------

-----------------------------------------YWDNETPLYKQLLFP----

------------------------------------------------------------

--------------------WTGYQIN-------HAKYQTS-------------------

--------------------TITARRVT--------------------RG----------

--------------------------------PMVKRDLKM-------------------

---------------------------ATLDWA---------------------------

------------------------------------------------------------

------------------------------------------------------------

------------------------------------------------------------

---FDFNTVQTVTDELKELTVHTQISNAT-----WEF---------------------PL

LDVTLRNVTNVNGYRLEGQVARSTHHI-FAEYDVTKE-----------------------

------------------------------------------------------------

--------------------PRETNKNFRMG-----LRTDVLVNNPDVSMFLNSPVLL--

----------------KLFGETALID-----------------------EPVVIDGGVNF

RILDQS------------------------------------------------------

------------SLTEERLIQTSIFVPN---------------------------PRS--

-------------------PQTHLTYDFDLRLE------NATISDRKD------------

-----YSLELTT----------------------------------------------PI

TLLKIN------------------------------------------NINLLHLMIDME

S---------------------------------------------------------GT

STLDHTYTLYD-------------------------------------------------

------------------------------------------------------------

------------------------------------------------------------

------------------------------------------------------------

----TRYSKP--------------------QVTVSNSIGAGWK----KPLANWALTIETP

SS-------------------VYLQNYTAL------------------------------

--------------------YTSDNGL------------QFFYENSQDSSLSDLLNFIEI

SNWSVR------------------------------------------------------

--------------------------------------------PSAISMYTSYIHQL--

---------ISTNVSNSLVLGGPVTEMRPRL----------------------------V

PMIMRDMLNKSFSRDFSSVKDLVLNKFIFNVTS---------------------------

---------STLTLRQSANMSSPAIANLSASHYIFLS--QSLFEQ---------KLSFAA

EQKRL-----------------------------------NISAALNHSLLIEDREITLK

TEGDLAV-------RTGLRMNTLTSVNVTMSKPDTPFDLAALH-----------------

----------------------HTEVNSPLIR----------------------FSTRSS

S------NGGFREVLNMVANHN--------------------------------------

--------------------------------------TSMTSRM---------------

-GSFDVIV

>L variegatus

------------------------------------------------------------

--------------------------------------------ESPERCKHVLKVTSCH

L-----------------DGNNQLGEEMSAHDLVFRTENGRIIDVLAHPDEPLHILNAKR

GILSMVQLDFEADDVDQITMNEVSVHGNCSSEMNVTQRDERQRPRKLEVVTDLNNC-MLR

KRPENMTRWQSFKDIVFNQTIMDKVINSSKICRYDLKGTTGEFKGIECEETHSFWPMNFN

SESGVRTFIRQNLISTNIDWLNSFKVDPAVTRSTCLRYEHDPKEVSQKPNDVTADQAMDI

LKALVKISREEIKMESPRLFGRWRAALEGMTNETIHEVFDRAYNCSEYSQTCRDNPDKFP

LYREYLLDGLLPCTTMACFSVKNYAIAKGIIPKPLANAWMVTMALQNYPTLELMSEMLRM

AQYSPSQITIFPLSIMVHNYWVNNEEIHTAEELPEPIIKTIRYLKSLIGNDCTVSAENQR

LALIFIQAIGNIGEPVERYDQLAFIYSERVSPVLIECTKNKQIPSDISKAAIQSMRRFVM

SDQLRSQVISELFKDTSLDVPQRIASYLIIIKATPSISELTEIWNFLKTEPVNQIKAFCY

SHIQNVIESSEPTLQRLKENLKTAMNGEQLPEYPKDIRKYSRFFELSKNLTEPWRNNTVA

AQLESGIIFNPESYLPQEVMFHYVLNALG-KSWDIFETGIEMKGFEPIVESVFGPEGIFP

EVSLQTMFETIDEKFLSKVREFAEAQLEKWAPQPAETENEVFSKKDTIGKKMKRQTQIGE

PQVIDVIHENTMNKLNQLHYLVGMEPSATDATVFLRLLGNEFGFVSAGDVLSLIPYTMGI

YEEFMNKTKQIPQLLSQGLNINTTRSFVFMDKTFVLPTGMGMPL--------LLSSFLRF

FCSSQFSQCFLNSYIC--SGAVEVLATMGIGMPTKVFTGVMANATVYHSSKIEGNVTIDG

THLKINLNNTDRPMNLFNYSTTYNLMKANGEIEYIPGVLQDAVRNQRCTNVSRVFGVDLC

VSWAYPNASYVAEAPRFPFTGPNSFNVTLVNTDQALKAYTIDAQYKQIRKGGA-------

------------------------------------------------------------

------------------------------------------------------------

--------------------------------------PSPIT-----------------

-------FSSIGRR----------------------------------NNRNTQETS---

-----------------------------------NIIDTVR--------VSFYAPGEE-

----------------------LTRNITSLM------------SVDRLRKTAKW------

---------------------TFTV--------------------------PEVKNFIMF

------------------------------------------------------------

------------------------------------AGLLNETTDS--------------

------------------------------------------------------------

------------------------------------------------------------

------------------------------------------------------------

------------------------------------------------------------

---------------------------------SSTYRA---------------------

--------------------VFNVSAAENTASVD----------------------FLVR

NE----------------TSVSQKNLTAVFNASINNL-----------------------

------------------------------------------------------------

--------------------------------------------------------YWAA

VGQFVKQ-----------------------------------------------------

------------------------------------------------------------

--------AESGDWNAHVTDTY--------------------------------------

------------------------------------------------------------

-----------------------------------------YWDDETPLYKQLLFP----

------------------------------------------------------------

--------------------WTGYQIN-------HAKYQTS-------------------

--------------------TIAAKRIT--------------------RG----------

--------------------------------PIVQRDLKM-------------------

---------------------------STLGWA---------------------------

------------------------------------------------------------

------------------------------------------------------------

------------------------------------------------------------

---FDFDTVQTVTDELKEFTVHTQISNST-----WEF---------------------PL

LDFILRNETKVNGYRLEGQVARNTHHV-FAEYDFTKE-----------------------

------------------------------------------------------------

--------------------QRETNRNFRMG-----LRTDVLVNNPDAAMFLNSPALL--

----------------SLFGETALID-----------------------EPVVIDGGMDF

RILDQS------------------------------------------------------

------------SLTEEHLIQTSIFVPN---------------------------PRN--

-------------------PQTHLTYDFDLRFE------NATISNQKD------------

-----FSVESIF----------------------------------------------HP

ILIQSN------------------------------------------IVNSLHLFIDME

S---------------------------------------------------------GA

SSLDHTYTLYD-------------------------------------------------

------------------------------------------------------------

------------------------------------------------------------

------------------------------------------------------------

----TRYSKP--------------------QVTVSNIIAAGWK----KPSANWVMDIETP

SM-------------------VYYQNYTAL------------------------------

--------------------YSRENGL------------QCFYENSQDSSFSDLLDFTEI

SNLSVR------------------------------------------------------

--------------------------------------------PSAVSMYWSYLHQL--

---------LSTNVSNSLAIEGPITEMRPRV----------------------------V

PMVVRDILNKTYNKDFQIVKDLILNKFIFNVTS---------------------------

---------SSLTLRQSANISSPVFANMSASHYIFLS--RNLFDQ---------QLTFVA

EQKRL-----------------------------------NISAGLNHSLLIEGREVTLK

TEGGLTA-------RTALRMNTLTSVNVTILKEDTPFDLAALH-----------------

----------------------HTEVDSPLIK----------------------FSTRSS

S------NGDFHGVLNMVANHN--------------------------------------

--------------------------------------TSMTSRL---------------

-GSFDVIG

>M musculus

--------------------MGPRKPALRTPLLLLFLLLFLDTSVWAQDEVL--ENLSFS

CPKDATRFKHLRKYVYNYEAESSSGVQGTADSRSATKINCKVELEVPQICGFIMRTNQCT

LKEVYGFNPEGKALMKKTKNSEEFAAAMSRYELKLAIPEGKQIVLYPDKDEPKYILNIKR

GIISALLVPPETEEDQQELFLD-TVYGNCSTQVTVNSRKGTV-PTEMSTERNLQQCDGFQ

PISTSVSPLALIKGLVHPLSTL---ISSSQTCQYTLDPKRKHVSEAVCDEQHLFLPFSYK

NKYGIMTRVTQKLSLEDTPKINSRFFSEGTNR---MGLAFESTKSTSSPKQ--ADAVLKT

LQELKKLSISEQNAQRANLFNKLVTELRGLTGEAITSLL----------------PQLIE

VSSPITLQALVQCGQPQCYTHILQWLKTEKAHPLLVDIVTYLMALIPNPSTQRLQEIFNT

AKEQQSRATLYALSHAVNSYF------DVDHSRSPVLQDIAGYLLKQIDNECT---GNED

HTFLILRVIGNMGRTMEQ-------VMPALKSSVLSCVRSTKPSLLIQKAALQALRKMEL

EDEVR-TILFDTFVNGVAPVEKRLAAYLLLMK-NPSSSDINKIAQLLQWEQSEQVKNFVA

SHIANILNSEELYVQDLKVLIKNALENSQFPTI-MDFRKFSRNYQISKSASLP-MFDPVS

VKIEGNLIFDPSSYLPRESLLKTTLTVFGLASLDLFEIGLEGKGFEPTLEALFGKQGFFP

DSVNKALY-WVNGRVPDGVSKVLVDHFG-------------YTTDGKHEQDMVN----GI

MPIVDKLIKDLKSK------------EIPEARAYLRILGKELSFVRLQDLQVLGKLLLSG

AQTLQGIPQMVVQAIREGSKNDLFLHYIFMDNAFELPTGAGLQLQVSSSG--VFTPGIKA

GVRLELAN-IQAELVAKPSVSLEFVTNMGIIIPDFAKSSVQMNTNFFHESGLEARVALKA

GQLKVIIPSPKRPVKLFSGSNTLHLVSTT-KTEVIPPLVENRQSWSTCKPL--FTGMNYC

TTGAYSNASSTESASYYPLTGDTRYELELRPTGE-VEQYSATATYELLKEDKSLVDTLKF

LVQAEGVQQSEATVLFKYNRRSRTLSSEVLIPGFDVNFGTILRVNDESAKDKNTYKLILD

IQNKKITEVSLVGHLSYDKKGDGKIKGVVSIPRLQAEARSEVHTHWSSTKLLFQMDSSAT

AYGSTISKRVTWRYDNEIIEFDWNTGTNVDTKKVASNFPVDLSHYPRMLHEYANGLLDHR

VPQTDVTFRDMGSKLIVATNTWLQMATRGLPYPQTLQDHLNSLSELNLLKMGLSDFHIPD

NLFLKTDGRVKYTMNRNKINIDIPLPLGGKSSKDLKMPESVRTPALNFKSVGFHLPSREV

QVPTFTIPKTHQLQVPLLGVLDLSTNVYSNLYNWSASYTGGNTSRDHFSLQAQYRMKTDS

VVDLFSYSVQGSGETTYDSKNTFTLSCDGSLHHKFLDSKFKVSHVEKFGNSPVSKGLLTF

ETSSALGPQMSATVHLDSKKKQHLYVKDIKVDGQFRASSFYAQGKYGLSCERDVTTGQLS

GESNMRFNSTYFQGTNQIVGMYQDGALSITSTSDLQDGIFKNTASLKYENYELTLKSDSS

GQYENFAASNKLDVTFSTQSALLRSEHQANYKSLRLVTLLSGSLTSQGVELNADILGTDK

INTGAHKATLKIARDGLSTSATTNLKYSPLLLENELNAELGLSGASMKLSTNGRFKEHHA

KFSLDGRAALTEVSLGSIYQAMILGADSKNIFNFKLSREGLRLSNDLMGSYAEMKLDHTH

SLNIAGLSLDFFSKMDNIYSGDKFYKQNFNLQLQPYSFITTLSNDLRYGALDLTNNGRFR

LEPLKLNVGGNFKGTYQNNELKHIYTISYTDLVVASYRADTVAKVQGVEFSHRLNADIEG

LTSSVDVTTSYNSDPLHFNNVFHFSLAPFTLGIDTHTSGDGKLSFWGEHTGQLYSKFLLK

AEPLALIVSHDYKGSTSHSLPYESSISTALEHTVSALLTPAEQTSTWKFKTKLNDKVYSQ

DFEAYNTKDKIGVELSGR--ADLSGLYSPIKLPFFYSEPVNVLNGLEVNDAVDKPQEFTI

IAVVKYDKNQDVHTINLPFFKSLPDYLERNRRGMISLLEAMRGELQRLSVDQFVRKYRAA

LSRLPQQIHHYLNASDWERQVAGAKEKITSFMENYRITDNDVLIAIDSAKINFNEKLSQL

ETYAIQFDQYIKDNYDPHDLKRTIAEIIDRIIEKLKILDEQYHIRVNLAKSIHNLYLFVE

NVDLNQVSSSNTSWIQNVDSNYQVRIQIQEKLQQLRTQIQNIDIQQLAAEVKRQMDAIDV

TMHLDQLRTAILFQRISDIIDRVKYFVMNLIEDFKVTEKINTFRVIVRELIEKYEVDQHI

QVLMDKSVELAHRYSLSEPLQKLSNVLQRIEIKDYYEKLVGFIDDTVEWLKALSFKNTIE

ELNRLTDMLVKKLKAFDYHQFVDKTNSKIREMTQRINAEIQALKLPQKMEALKLLVEDFK

TTVSNSLERLKDTKVTVVIDWLQDILT-----QMKDHFQDTLEDVRDRIYQMDIQRELEH

FLSLVNQVYSTLVTYMSDWWTLTAKNITDFAEQYSIQNWAESIKVLVEQGFIVPEMQTFL

WTMPAFEVSLRALQEGNFQTPVFIVPLTDLRIPSIRINFKMLKNIKIPLRFSTPEFTLLN

TFHVHSFTIDLLEIKAKIIRTIDQILSSELQWPLPEMYLRDLDVVNIPLARLTLPDFHVP

EITIPEFTIPNVNLKDLHVPDLHIPEFQLPHLSHTIEIPAFGKLHSILKIQSPLFILDAN

ANIQNVTTSGNKAEIVASVTAKGESQFEALNFDFQAQAQFLELNPHPPVLKESMNFSSKH

VRMEHEGEIVFDGKAIEGKSDTVASLHTEKNEVEFNNGMTVKVNNQLTLDSHTKYFHKLS

VPRLDFSSKASLNNEIKTLLEAGHVALTSSGTGSWNWACPNFSDEGIHSSQISFTVDGPI

AFVGLSNNINGKHLRVIQKLTYESGFLNYSKFEVESKVESQHVGSSILTANGRALLKDAK

AEMTGEHNANLNGKVIGTLKNSLFFSAQPFEITASTNNEGNLKVGFPLKLTGKIDFLNNY

ALFLSPRAQQASWQASTRFNQYKYNQNFSAINNEHNIEASIGMNGDANLDFLNIPLTIPE

INLPYTEFKTPLLKDFSIWEETGLKEFLKTTKQSFDLSVKAQYKKNSDKHSIVVPLGMFY

EFILNNVNSWDRKFEKVRNNALHFLTTSYNEAKIKVDKYKTENSLNQPSGTFQNHGYTIP

VVNIEVSPFAVETLASSHVIPTAISTPSVTIPGPNIMVPSYKLVLPPLELPVFHGPGNLF

KFFLPDFKGFNTIDNIYIPAMGNFTYDFSFKSSVITLNTNAGLYNQSDIVAHFLSSSSFV

TDALQYKLEGTSRLMRKRGLKLATAVSLTNKFVKGSHDSTISLTKKNMEASVRTTANLHA

PIFSMNFKQELNGNTKSKPTVSSSIELNYDFNSSKLHSTATGGIDHKFSLESLTSYFSIE

SFTKGNIKSSFLSQEYSGSVANEANVYLNSKGTRSSVRLQGASKVDGIWNVEVGENFAGE

ATLQRIYTTWEHNMKNHLQVYSYFFTKGKQTCRATLELSPWTMSTLLQVHVSQLSSLLDL

HHFDQEVILKANTKNQKISWKGGVQVESRVLQHNAQFSNDQEEIRLDLAGSLDGQLWDLE

AIFLPVYGKSLQELLQMD-----GKRQYLQASTSLLYTKNPNGYLLSLPVQELADRFIIP

GIKLN-------------------------DFSGVKIYKKLSTSPFALNLTMLPKVKFPG

IDLLTQYSTPEGSSVPIFEATIPEIHLTVSQFTLPKSLPVGNTVFDLNKLANMIADVDLP

SVTLPEQTIVIPPLEFSVPAGIFIPFFGELTARAGMASPLYNVTWSAGWKTKADHVETFL

DSMCTSTLQFLEYALKVVETHKIEEDLLTYNIKGTLQHCDFNVEYNEDGLFKGLWDWQGE

AHLDITSPALTDFHLYYKEDKTSLSASAASSTIGTVGLDSSTD-DQSVELNVYFHPQSPP

EKKLSIFKTEWR--YKESDGERYIKINWEEEAASRLLGSLKSNVPKASKAIYDYANKYHL

EY-------VSSELRKSLQVNAE----HARRMVDEMN--MSFQRVAR----------DTY

QNLYEEMLAQKSLSIPENLKKRVLDS-IVHVTQKYHMAVMWLMDSFIHFLKFNRVQFPGY

AGTYTVDELYTIVMKETKKSLSQLF----NGLGNLLSYVQNQVEKSRLINDITFKCPFFS

KPCKLKDLILIFREELNILSNIGQ------QDIKFTTILSSLQGFLERVLDIIEEQIKCL

KDNESTCVAD----HINMVFKIQVPYAFKSLREDIYFVLGEFNDFLQSILQEGSYKLQQV

HQYMKALREEYFDPSMVGWTVKYYEIEENMVELIKTLLVSFRDVYSEYSVTAADFASKMS

TQVEQFVSRDIREYLSMLTDINGKWMEKIAELSIVAKETMKSWVTAVAKIMSDYPQQFHS

NLQDFSDQLSSYYEKFVGESTRLIDLSIQNYHVFLRYITELLRKLQVATANNVSPYIKLA

QGELMITF

>SIG

------------------------------------------------------------

------------------------------------------------------------

------------------------------------------------------------

------------------------------------------------------------

------------------------------------------------------------

------------------------------------------------------------

------------------------------------------------------------

------------------------------------------------------------

------------------------------------------------------------

------------------------------------------------------------

------------------------------------------------------------

-------------------------+----------------------------------

------------------------------------------------------------

------------------------------------------------------------

------------------------------------------------------------

------------------------------------------------------------

------------------------------------------------------------

------------------------------------------------------------

------------------------------------------------------------

------------------------------------------------------------

------------------------------------------------------------

------------------------------------------------------------

------------------------------------------------------------

------------------------------------------------------------

------------------------------------------------------------

------------------------------------------------------------

------------------------------------------------------------

------------------------------------------------------------

------------------------------------------------------------

------------------------------------------------------------

------------------------------------------------------------

------------------------------------------------------------

------------------------------------------------------------

------------------------------------------------------------

------------------------------------------------------------

------------------------------------------------------------

------------------------------------------------------------

------------------------------------------------------------

------------------------------------------------------------

------------------------------------------------------------

------------------------------------------------------------

------------------------------------------------------------

------------------------------------------------------------

------------------------------------------------------------

------------------------------------------------------------

------------------------------------------------------------

------------------------------------------------------------

------------------------------------------------------------

------------------------------------------------------------

------------------------------------------------------------

------------------------------------------------------------

------------------------------------------------------------

------------------------------------------------------------

------------------------------------------------------------

------------------------------------------------------------

------------------------------------------------------------

------------------------------------------------------------

------------------------------------------------------------

-----------------------------------------------------------+

------------------------------------------------------------

------------------------------------------------------------

------------------------------------------------------------

------------------------------------------------------------

------------------------------------------------------------

------------------------------------------------------------

------------------------------------------------------------

------------------------------------------------------------

------------------------------------------------------------

------------------------------------------------------------

------------------------------------------------------------

------------------------------------------------------------

------------------------------------------------------------

------------------------------------------------------------

------------------------------------------------------------

------------------------------------------------------------

------------------------------------------------------------

------------------------------------------------------------

------------------------------------------------------------

APOD

>H sapiens

-MVMLLLLLSALAGLFGAAEGQAFHLGKCPNPPVQEN-------------FDVNKYLGRW

Y----EIEKIPTTFENGR-CI--QANYSLMENGKIKVLNQELRADGTVNQIEGEATPVNL

TEPAKLEVKFSWFMPSAPYWILATDYENYALVYSCTCIIQ-LFHVDFAWILARNPNLPPE

TVDSLKNILTSNNIDVKKMTVTDQVNCP--------------------------------

------------------------------------------------------------

-------------------------KLS--------------------------------

--

>H_glaber

-MVTMLLLLSALAGLVAEAEGQAFHLGKCPTPPVQEN-------------FDVNKYLGRW

Y----EIEKIPVSFEKGN-CI--QANYSVKENGNIKVLNQELRSDGTVNQIEGEATQSNI

TDPAKLGVKFFQLMPSAPYWVLATDYDNYALVYSCTNIIW-LFHIDHVWILGRNRYLPPE

TVTYLKDILTSNSIDIEKMTITDQVNCP--------------------------------

------------------------------------------------------------

-------------------------DFL--------------------------------

--

>M_brandtii

KMVTALLLLSALAGLFRAAEGQAFHLGKCPTPPVQEN-------------FDVNKYLGRW

Y----EIEKIPVSFEKGS-CI--QANYSLMENGNIKVINQELRSDGTVNQIEGEASQSNF

TEPAKLGVKFFWLMPSAPYWVLATDYENYALVYSCTTIVW-LFHVDHVWILGRNPYLPPE

TVTHLKDILTSNNIDIEKMTITDQGNCP--------------------------------

------------------------------------------------------------

-------------------------EFL--------------------------------

--

>S franciscanus

----------------------VFGFGGCPRVRVARD-------------FDLDRYFGRW

F----PITKFYTLYEDGLTCV--SAEYRPGDDGSIRVINSGRTQDGDVTAIEGVAFQPNP

EEGAKLQVQFLRGQTPGDYWVLDVEYDRYALVHSCSEYLRDLIYVQNTWIIARDRRMSEE

DYDDAMRYLASMGINTRKLKKAEQRKCP----------------------YYRPRPSEEP

ETERPRPSEEPGTDRPRPSEEPGTDRPRPSEEPGTDRPRPSEEPGTDRPRPSEGPETERP

RPSEGPETE-PQPSEIPGSERPQPHRIKESERHPPHRTAETAR-HPLLSSALARNSFVSS

CL

>S_purpuratus

---MRVLGLLLLLGFASFSSAQVFGFGGCPRVRVARD-------------FDVDRYFGRW

F----EITKFYTFYQDGLTCV--SAEYQPGDDGSIRVINSGRTQDGDVTTIEGVAFQPDP

EEGAKLQVQFFRGQTPGDYWVLDVEYDSYALVHSCSEVFYGWFNIQSNWLLARDRMPSEE

VIEDALRKLGEQGINVQKLKRTNQRNCEGEPPRPTEWPVTHRPRPSERPGTDRPRPSEEP

ETERPRPSEEPGTDRPRPSEGPGTDRPRPSEEPETERPRPSEEPGTDRPRPSEEPETERP

RPSEEPETERPRPSEEPSTDRPRPSEEPETERPRPSEEPGTDRPRPSEEPEAFRK-----

--

>L variegatus

-------------------YEREISSAPQPQQKPQKRKKQSKKQLAHCLYIPMFKYFHRL

FCKDPNLLMLYTIIMSKHIYFHHEGEYHFPSP---QALPPKKKQEPNKQNAISIIVYTDI

Q--IFLSVFHHSSQTPGEFWVLDVDYDSYALVHGCTEIFFGWFNVQNNWVLARDRRPSEE

DIQDALRKLTEQGINVKKFIRADQTDCE--------------------------------

-TEPPSPTETPGTSLTRNS-----------------------------------------

-------------SDYCKVNAPRVTRFSIYFIIVPS------------------------

--

>M_musculus

-MVTMLMFLATLAGLFTTAKGQNFHLGKCPSPPVQEN-------------FDVKKYLGRW

Y----EIEKIPASFEKGN-CI--QANYSLMENGNIEVLNKELSPDGTMNQVKGEAKQSNV

SEPAKLEVQFFPLMPPAPYWILATDYENYALVYSCTTFFW-LFHVDFVWILGRNPYLPPE

TITYLKDILTSNGIDIEKMTTTDQANCP--------------------------------

------------------------------------------------------------

-------------------------DFL--------------------------------

--

>SIG

------------------------------------------------------------

-----------------------------------+------------------------

--------------------------------------+---------------------

------------------------------------------------------------

------------------------------------------------------------

------------------------------------------------------------

--

APOH

>H sapiens

------------------------------------------------------------

------------------------------------------------------------

------------------------------------------------------------

------------------------------------------------------------

------------------------------------------------------------

------------------------------------------------------------

------------------------------------------------------------

------------------------------------------------------------

------------------------------------------------------------

------------------------------------------------------------

------------------------------------------------------------

---------------------------------MISPVLI--------------------

------------------------------------------------------------

------------------------------------------------------------

------------------------------------------------------------

------------------------------------------------------------

------------------------------------------------------------

------------------------------------------------------------

------------------------------------------------------------

------------------------------------------------------------

------------------------------------------------------------

------------------------------------------------------------

-------------------------------------------------------LFSSF

LCHVAIA-----------------------------------------------------

------------------------------------------------------------

------------------------------------------------------------

------------------------------------------------------------

------------------------------------------------------------

--------------------------------------GRTCPKPDDL------------

--------PFSTVVPLKTF--------YEPG---EEITYSCK------------------

------------------PGYVSRG-----------------------------------

------------------------------------------------------GMRKFI

CPLTGLWP---------------------------------------------INTLKCT

--------------------PRV-------------------------------------

-------------CPFAGILENGAVRYTTFEYPNTISFSCNTGFYLNGADSAKCTEEGKW

SPELPVCAPIICPPPS-----------IPTFA----------------------------

----TLRVYKPSA-------------GNNSLYRDTAVFECLPQHAMFGNDTITCTTHGNW

TK----------------------------------------------------------

LPECREVKCPFPSRPDNGFVN---------------------------------------

------YPAKPT----------LYYKDKATFGCHDGYSLDGPEEIECTKLGNWSAM-PSC

K---------------ASCKVPVKKATVVYQGERVKIQEKF----KNGMLHGDKVSFF--

-CKNKEKKCSYTEDAQCIDGTIEV--PKCFKEHSSLAFW---------------------

------------------------------------------------------------

------------------------------------------------------------

--------KTDA-------------SD---------------------------------

------------------------------------------------------------

------------------------------------------------------------

------------------------------------------------------------

----------------------VKPC----------------------------------

------------------------------------------------------------

------------------------------------------------------------

------------------------------------------------------------

------------------------------------------------------------

------------------------------------------------------------

------------------------------------------------------------

------------------------------------------------------------

------------------------------------------------------------

------------------------------------------------------------

------------------------------------------------------------

------------------------------------------------------------

------------------------------------------------------------

------------------------------------------------------------

-------

>H_glaber

------------------------------------------------------------

------------------------------------------------------------

------------------------------------------------------------

------------------------------------------------------------

------------------------------------------------------------

------------------------------------------------------------

------------------------------------------------------------

------------------------------------------------------------

------------------------------------------------------------

------------------------------------------------------------

------------------------------------------------------------

---------------------------------MTSPVLV--------------------

------------------------------------------------------------

------------------------------------------------------------

------------------------------------------------------------

------------------------------------------------------------

------------------------------------------------------------

------------------------------------------------------------

------------------------------------------------------------

------------------------------------------------------------

------------------------------------------------------------

------------------------------------------------------------

-------------------------------------------------------LLSGF

LCHVAIA-----------------------------------------------------

------------------------------------------------------------

------------------------------------------------------------

------------------------------------------------------------

------------------------------------------------------------

--------------------------------------GQTCPQPDDV------------

--------PFSTVTPLKTS--------YDPG---EQIVYFCK------------------

------------------PGYLSQG-----------------------------------

------------------------------------------------------GMRRLT

CPFTGIWP---------------------------------------------LNTLKCT

--------------------PRV-------------------------------------

-------------CPFAGILDNGAVRYTTFEYPNTISFACNSGYYLNGTNSAQCTEEGKW

SPELPVCAPITCPPPP-----------VPKFA----------------------------

----TLRIYKPSA-------------RNNSLYRDRAVFKCLPHHAMFGNDTIACTAHGNW

TE----------------------------------------------------------

LPECREVKCPFPSRPENGFVN---------------------------------------

------YPAKQV----------LLYKDKATYGCHDTYNLDGPEEVECTKLGNWSAQ-PSC

K---------------ASCKLSVKKATVLYQGERVKLQERF----KNGMLHGDKVSFF--

-CKNKEKKCSYTEEAQCVDGTIEI--PKCFKEHSSLAFW---------------------

------------------------------------------------------------

------------------------------------------------------------

--------KTDA-------------WD---------------------------------

------------------------------------------------------------

------------------------------------------------------------

------------------------------------------------------------

----------------------IMPC----------------------------------

------------------------------------------------------------

------------------------------------------------------------

------------------------------------------------------------

------------------------------------------------------------

------------------------------------------------------------

------------------------------------------------------------

------------------------------------------------------------

------------------------------------------------------------

------------------------------------------------------------

------------------------------------------------------------

------------------------------------------------------------

------------------------------------------------------------

------------------------------------------------------------

-------

>M_brandtii

------------------------------------------------------------

------------------------------------------------------------

------------------------------------------------------------

------------------------------------------------------------

------------------------------------------------------------

------------------------------------------------------------

------------------------------------------------------------

------------------------------------------------------------

------------------------------------------------------------

------------------------------------------------------------

------------------------------------------------------------

---------------------------------MISPALI--------------------

------------------------------------------------------------

------------------------------------------------------------

------------------------------------------------------------

------------------------------------------------------------

------------------------------------------------------------

------------------------------------------------------------

------------------------------------------------------------

------------------------------------------------------------

------------------------------------------------------------

------------------------------------------------------------

-------------------------------------------------------LFSSF

LCHVAVA-----------------------------------------------------

------------------------------------------------------------

------------------------------------------------------------

------------------------------------------------------------

------------------------------------------------------------

--------------------------------------GRTCPRPDDV------------

--------PFATVTPLRSS--------YDPG---EQIMYFCK------------------

------------------PGYVSRG-----------------------------------

------------------------------------------------------GMRRFT

CPLTGIWP---------------------------------------------INTLRCT

--------------------PRV-------------------------------------

-------------CPFAGILENGAVRYTTFEYPNTISFSCNNGFFLNGTKSAQCTEEGIW

SPNLPVCSPVTCPPPP-----------TPKFA----------------------------

----VLSAYEPLA-------------GNRSVYGKQAVFKCLPNYAMFGNDTATCTAHGNW

TE----------------------------------------------------------

LPECREVKCPFPIRPDNGFVN---------------------------------------

------YPAKEA----------LSYKDKATYGCHDTYVLDGPEEVECNKFGNWSAQ-PSC

KGEEKAILEPMTSGSMASCKVSVKKATVIFEGERVKIQEKF----KNGMLHGQKVSFF--

-CKNKEKKCSYTEDAQCIDGTFEV--PSCFKERQRCALWIRKLCEPSGTGAGIMGRKNRN

LYAKLLLHMLKRGVIEGPFIHRPEPGTLKTLPSYMSIYFDEPNPAQAKSSSPEGLPDWVM

GELGTSEHKLNESWKLSSGDSYSLVQSPTDGHREQYTAQLQRTSHSRSPTYREDGQHVTQ

KSCEVHLKKSAVSLDDSDIEARLNSWNLGIENPRYLRQKPIPVSLMTPKFSLRKSSSFHE

DHLLSRMHEKELDMKTKMIEAKCHEEKLKLQQKHDADVQKILERKNSEIDELKILYKTKQ

NETEETVRKLEKKVQTLIRDCQVIRETKENQISELKKMCEQSTEFLNNDWEKKLHNAVAE

MEREKFDLQKRHTESIQELLEDTNARLRRMEGEYTAQTQATASPFPLPAVVRHSSLFSMC

PASLMCAGTTTCVQAIVAAFRALPPCPLKNSNLVFRVLPLSSQYGNRDSVPKNQMVKELE

SRVQQLTGEAENSNLQKQKLIQEKLELERCYQITCSELQEVKARRNSLHKEKD-HLMNDY

EQSVKLLQNKYEADINLLKKEHTLSVSKASGTIKELEQNVCQLKLQLQESELQRKQQLQD

QENEFQMEKSNLKRTYEKKVHDLQSELDKEKEDTQKKIYKFEEALKSVSPIPYVSDVFIL

TECMILTIQHLDSRCNLVRSRQFGTGKPGLTQSVQKLKTWKVTMMKVGRAAEVLSSYADA

KRRTAQMHSEKRRSYEKCGQSVEKLRLFRPHWRETLTESSHLYSLIGSCSLPLLTTTFPI

MVSPFVTVNAGMPLSYSFFYPLLLILQLTLTSFSYILTPREKEEQLARVTEVQRLQAQQA

DAALEEFKRQVELNSEKVYAEMKDQMEKVEADLTRSRSLREKQSKEFLWQMEDVKQRYEQ

QIVELKLEHEQEKTHLLQQHNAEKDSLVRDHERDIENLEKQLRAANMEHENQIQEFKKRD

SQVIADMEAQVHKLREELINVNTQRKQQLVELGLLREEEKQRAARDHEATVSKLKAESEK

MKIELRKTHAAETEMTLEKANCRLKQIEKEYTQKLAKSLQTISELQTAISALKEESSRQQ

LAAERRLQDVTQKFEDEKKQLIRDNDRAIKALRDELEN------------RSNQVRCAEK

KLQHRDLEAQEQITY---IRQEYETKFKGLMPASLRQELEDTISSLKSQVNFLQKRASIL

Q-------------------EELTTYQGRRYGRDQNAC----------------------

-------

>S franciscanus

SGYEGVDCEIEIDECASNPCQNGATCVDEVDGYTCTCLSGYEGTNCEIEIDECASNPCQN

GATCVDVIDGYSCSCAPGFEGDNCEIDIDECASNPCENGATCIDGVNGYTCDCLPGFEGT

NC-EIDINEC-----------ASNPCQNGATCIDGVNGY----SCSCLPGYEGDTCEID-

-IDECASNPCQNGATCVDGVNGYTCE--CAPGFEGVF---------CEIDIDECSSSPCQ

NGATCIDLVN---------GYTCSCLPGFE-GQKVLQGARANSTQAVFLLTDGLSNGPNP

VPVAVSLKDDGVEVFSFGIRDGYIPELLQMASEKKDEHCYILDSFAEFEALARRALHEDL

RMGEFIEETPEKCSSRCLGENHCCDVMASCRCGTHTGSYDCVCPAGFYG-----------

-----------SGLRGK-CTCVP--GFEGINCEIEIDECASDPCQNGATCIDRINGYTCD

CLPGFEGDHCEIDIDEC-VIHCPLLQRPENGEFVECSNVVNGACAIRCNRGYEVTGSSIR

LCFSNGTWSGEGFDCRVKTCEVLIAPLNGSLQCTSDDYAFGTVCSATCDTGFKLIGSHSR

RCLPIAHWDGLPVHCRAIQCPTLPPIPHGYMTPSHCSTQRQDYGNM-CKI---ACTQGFY

RSGPRKKE------CMANGSWNNKFDPSQACIDYISPSLECPTAISTVTSPHQDRVVLEW

DVPRPTDNSGEMPLLHAIPNVHPPWTFPLGQWNITYSATDQSGNIAQCVFSVIVEDKEPP

SIR---------RCRSPSTFITSQKEHRVTWEEPLFSDNSGGDITVTRSHDPGSVFLFGT

TLVEYLATEAALTTKSYFIYIMFQRNLCQKPRDPVGGSAICFHTTQGMNCSVTCGPGYAM

STRPRKLYFCALNGTWYPDGKVPWDDCSETHHAKNVLQPMTFLYETSDCDTAFARNIERI

LATNLEEINIDECASSPCQNGATCVDLINGYDPIPFLRGATSSLQQTSPGADSRLALQGA

GEVSVTAHRLNILQSPNLFVNILVTASASPNETATLASGTEQTYQMVVEVAASVPEQIQS

NFDNGTIVTGTNRSSERTFQVNRITVYRENIQPVCSHCEINIDECASNPCVQCIPGTYFD

IVTLSCEYCEIGTYQNQIGQLSCLHCPNGTSTVNTKSTSLASCKAYCEPGSKSSTGVELC

EL----CPLNTYQPNRGSTQCLECPRRRHTPSHGAWKLSDD-----EAYCPRGKTSINGL

APCLPCPAGTFKMSQRSSYCLLCPEDYPLSNP-SIPITYC-SSANCQCPAGYTGTLCEID

IDECASNPCQNGATCVDLINGYECQCAPGFTGTNCEIDIDECASNPCQNGATCVDLINGY

TCQCPPGFTGTNCEIDIDECASNPCQNGATCVDLINGYTCQCPPGFTGTNCEIDIDECAS

NPCQNGATCVDLINGYTCQCAPGFTGTNCEIDIDECASNPCQNGATCVDLINGYTCQCPP

GFTGTNCEINIDECASNPCQNGATCVDLINGYTCSCPPGFTGVHCETEINECASMPCQNG

GICEDQVLESDFPHQTAVTISFWMFTDNTGNSGTVFSYAVSDSSDNALTVTDYSGFVLKV

NEESIYSDISARDANWHYISVTWTSETGAWAFYLDGSEAASGTGLSTGTFVKGRGMFIVG

QEQDTYNGSYTHKESFIGEITYLTVWDRVLGAAEIYDLGRTCHKPSDVIIAWPDFLSGME

GQIEKRNNTFCKGCPRPNAPEAGFVKGYQPSRPLTSVTYHCQLGFELPRHEVTRAECQIS

GEWSPGDRAYCDIINCGYPGHLKHGYVIGRMYLYGDQVFYTCVENFTLVGPLARECMETG

YWSGFTPECGVVFCREPPHVSNAVVFSSEGERPVHMAFPRQSATYQCARGYRLIGLPNVT

CQIDGEWTAPPTCRIVTCPPLTSSPNSMLITTNGSVFSSIAEFRCITGYRMTGVNVLQCQ

ETGRWDNAEPLCTILSCGHPPNVPLTTSTYDRVTYGSSVWYTCNEGYEADRTVIHCGGNG

LWEPSLPECRRIQCPMPPAVANGSMIGGDFGFNATVIYDCDRGYTIAGNREISCMSNGQW

SSSPPVCQAVSCGKPPKMDNHQLAADQSTEFTYGMTVEYTCPTGYLFHSDSPELTCGADG

RWVGTLSDCIPVSCGPPNDILNSKIQGQDYTYNQTVVYACKTGFILSGVSVITCLASGQW

TESSLRCDIVECGEPPALLHAVASYRRLFNESVTYTCQSGYVTRNAPHLKCLGNGSWSVV

SQVCKPISCGPLSLPLHGEVTTTNITLQGIARYSCWHGYTLAGSSTRKCSESGSWSVSSQ

ECKPISCGPLSLPLHGEVTTTNITLDGIARYSCNHGYLLIRSRTRTCSESGEWSGSTPEC

Q--------------LVECGSPPLAENAYFLGNNFTFNNTLIYFCADGFIFGDDVVSMET

TCQADGRWSEEEIRCEAVSCGMPVTPPQGVIVGTNFSYL---------------------

-----------------------------------------------NSVQFMCIPGYVI

TGSESATCTSNGTWS-HLDTRCKPLDCGSPPDINNAEFELNTSTYGSTVAYQCDVGYMAA

SELMIKCDETGV-------------WSGNVTNTSCQPVPCLEIPLVYNGSIE--GNIRVI

YGDIVRARCWTGYTIAGESVRKCLGNGS-----WSVVSQVCKPISCGPLSLPLHGEVTTT

NITLDGIARYSCNHGYLIIGSSTRKCSESGEWSGSSPECQP----ISCGPLSLPLHGEVT

TTNITLDGIARYSCNHGYLLIGSSTRTCSESGEWSGSTPECQPISCLIPILPTHGIAITI

NIRYLDIARYKCEPGYRLIESARRRCQASGEWSGYEPKCQPIECSVEEAFPLGIVIRNYI

VFYLDKVRYRCWTGYTIAGESVRKCLGNGSWSVVSQVCKPISCGPLSLPLHGEVTTTNIT

LEGIARYSCNHGYLLIGSRTRTCSESGEWSGSTPECQPIECPSPQRIRHGTLQMNTQVP-

-----------IYRSSVTYRCWTGYTIAGESVRKCLGNGSWNVVSQVCKPISCGPLSLPL

HGEVTTTNITLSGIARYRCWTGYTIAGESVRKC-------------LGNGSWSGSSQECQ

PISCGPLSLPLHGEVTTTNITLDGIARYSCNHGYLLIESRTRTCSESGEWSGSTPECQPI

TCHQPDDVEHGIIINLGNMQLLDEIQYRCWTGYTIAGESVRKCLGNGSWSVVSQVCKPIS

CGPLSLPLHGEVTTTNITLNGIARYSCWHGYTIAGSSVRKCSESGSWSGSSPECQPISCG

PLS----LPLHGEVTTTNITLQGIARYSCNHGYLLIGSRTRTCSESGEWSGSTPECQPNP

CQALPRIPHAIYPADKTRFIYGDIVQARCWTGYTIAGESVRKCLGNGSWSVVSQVCKPIS

CGPLSLPLHGEVTTTNITLGEIARYSCNHGYLLIGSRTRTCSESGEWSGSLPECQPITCE

DLAAPKNASYQSTSVSFGSAVVIQCDNGFKLVGTSSIQCQSAGQWSHPPPRCEAIECLIP

PAIDHGIAIFISNSYGDSVTFKCHPGYYLVGSSSIRCQAQGTWSQYPPKCTAFGSLASLE

RPVCVLPCLHGGKCVGPYSCECPYGFTGSRCEHRCEICVNEC-------QSHPCLIGATC

VC-----

>S_purpuratus

-------------------MARYYYLISVLLPFLCFSLSFVRGL------------PARA

PDLKLLLASGINIS----FPDEHRQLEF---ADYPQADGLVSQPRPADDPLP-LPLIQSL

SYLDIDLEQSHHELYEALWRNADVPMDTSSATKIDVLGYLLKKQVEKLRRVSNGQVELVF

LVDSSASVGIENFFNELKFVKKLLADFTVAPDATRVAIITFSSKHRVELNVNQLDNSIAG

KHHHKCALLNEDLPKITYVGGGTYTKGAFELAKKVLQGARANSTQAVFLLTDGLSNGPNP

VPVAVSLKDDGVEVFSFGIRDGYIPELLQMASEKKDEHCYILDSFAEFEALARRALHEDL

RMGEFIEETPEKCSSRCLGENHCCDAMASCRCGTHTGSYDCVCPAGFYG-----------

-----------SGLRGK-CTPCPIGSYKRDALPGGINTCLPCPVQNHVSPFNNTSPNMCV

CKDGYRWD---IATHRCKVIHCPLLQRPESGEFVECSNVVNGACAIRCNRGYEVTGSSIR

LCFSNGTWSGEGFDCRVKTCEVLTAPLNGSLQCTSDDYAFGTVCAATCDTGFKLIGSHSR

RCLPIAHWDGLPVHCRSIQCPTLPPIPHGFMTPSHCSTQRQDYGNI-CKI---ACTQGFY

RRGPRKKE------CMANGSWNNKFDPSQACMDYIAPSLECPTAISTVTSPHQDRVVLEW

DIPRPTDNSGEMPLLRAIPNVHPPWTFPLGQWNITYSATDQAGNIAQCVFSVTVEDREPP

SIR---------RCRSPSTFITSQREHRVTWEEPLFSDNSGGDITVIRSHDPGSVFLLGT

TLVEYLATDEAGNTRSCVIYVDVQQNLCQKPRDPVGGSAICFHTTQGMNCSVTCGPGYAM

STRPRKLYFCALNGTWYPDGKVPWDDCSETHHAKNVLQPMTFLYETSDCDTVFARNIERI

LATNLEEPIRNYCGENVQCELSSVKANCEDRDPIPFLQGAPSSLQQTSPGADSRLALQGA

GEVSVTAHRLNILQSPNLFVNIVVNASASPNETAALTSPTDQTYQMVVEVAASVPEQIQS

NFDNGTIMTNSNRSSERTFQVNRITVYREDIQPVCSAGAVRRGEGEGAKCVQCIPGTYFD

IVTSSCEYCGIGTYQNQIGQLSCLHCPNGTSTVNTKSTSLADCKAHCEPGSKSLTGLELC

EL----CQLNTYQPNRGSTQCVECPRRRHTPSHGARKLSDC-----KAYCPRGKTSVNGL

TPCLPCPAGTFKISQRSSYCLLCPEDYPLSNPGSITITYCRNSANERSPAGRMMSLRSLP

FSECFSSPCRNGGSCESSPGGYVCTCPAGYTGPRCETDINECETAMCMNNGSCRDLIDGY

MCHCPRGFTGRYCSEDVDECRTNPCHNGATCIDGPQYYECVCPDGYTGRNCEEDVDDCAL

DPCYNFGTCTDFIGGFQCTCQAGYLGDLCEMEVDECLSYPCDNNATCKDMVNEFRCECLA

GFSGTLCEINIDECATTPCLNGATCKDLIAGIECVCMPGYTGVFCEEEMTWDFNMVFDRG

GIDDFVVLERDFPTLTAVTISFWMLTDNTGNSGTVFSYAVSDSSDNALTVTDYSGFVLKV

NEETIVSDITANDGRWHHIVATWDSNGGTYTMYKDGVQQANGTGLSNGMSIPGGGTLILG

QDQDTLGGGFTHLESFIGEITYLTVWERALGAAEIYDLGRTCQKPSDVIMAWPDFLSGME

GQIEKRNNTFCQGCPYPTAPEAGFVKGYQPSRPLTSVTYHCQLGFELPRNEVTRAECQVS

GEWNPGDRAYCDRINCGYPGHLKHGYVIGRMYLYGDQVFYTCVENFTLVGPLARECMETG

YWSGFTPECKYVFCGEPPHVPNAVVFSSEGERPVHMAFPRQSATYQCARGYHLIGLPNVT

CQIDGEWTAPPTCRIVSCPPLTSSPNSMLITTNGTVFSSIAEFRCITGYRMTGMNVLQCQ

ETGRWDNAEPLCKILSCGHPPNVPLATSTYDGLTYGSSVWYTCNEGYEADRTVIHCGGNG

VWEPSLPECRRIQCSMPPAVANGSMIGGDFGFNATVIYDCDRGYTVAGNREISCMSNGQW

SSSPPVCQAVSCGEPPKMNNHQLVADQSPEFTYGITVEYTCPSGYLFDSDSTELTCGAGG

HWVGTLSDCIPVSCGPPNDIPNSNIKGQDYTYNQTLVYTCKTGFILSGVSVITCLASGQW

TESSVRCDIVECGEPPAVPHAVASHGRLYNESVTYACQAGYVTRNAPHLRCSAEGQWWGD

LPVCEPITCEVPLAPDHGFIVGTNWSFQSEIVMECDLGYRLHGEATRQCQSSGQWSNTSA

ECILIFCPEPPVIRHGNAHFDNLIYGSSVTYKCHEGYILEGESSRNCSAQGEWTQEPPSC

K--------------LLECGSPPLVENAYFLGDNFTFNNTLIYFCDDGFIFGGDVVSMET

TCQADGRWSEEEIRCEAISCGMPVTPPQGFILGTNFSYL---------------------

-----------------------------------------------NSVQFVCNPGYVL

TGSKSATCTSNGTWS-HSDTRCKPLDCGSPSDVNNAEFELNTSTYGSTVAYQCDVGYKAI

SKLMIECDETGV-------------WSGNVTNTSCQPVPCLEIPLVYNGLIE--GEGLFV

YGDIVRAHCDEGFKYQTLQEMRCSENGI-----WDPANITCVPITCPPVLPPDNGFIAVD

SNSYGEVMVFDCNEGFSIDGNDTLLCEEDGEWNGDMPQCLP----IICEDLAIPQNGSVQ

STGVSFGSQVVYTCDDGFELVGTSVTECAGQGVWSHPPPVCTLIECPMPINPAHGIAIFL

NNRYLDDVSFGCEPGYHLVGLARRRCQANGTWSGYEPKCRPIECRVEEAFPLGIVIRNHT

VFNGDKVLYSCNPGYMIAGQSFRICGNDGILSGQQPTCIRVTCGPPPNLLLGNVTLPSDR

YESNATYSCSLGFTLDGPSSRQCLSNGSWSFAHQDCRVIECPSPPPIRHGTLQMNTKIP-

-----------IYRSSVTYTCDVGYAVKGESMLTCDEHGSWSNTMPSCELVSCGQPPFLE

KGIVSFDVTTFSSQATYRCWTGYIIAGESVRTC-------------LANGSWSLVSQVCK

PISCGPLSLPLHGEVTNTNITLDGIASYSCDHGYVLIGSRTRTCSVSGEWSGSTPECQPI

TCQQPDDVEHGIIINLGNMQLLDEIQYRCNEGYKMRGEDRRICQADQTWSGSLPACRPIS

CDTPPIVINGVRSYKAITFNSTAVYDCHSGYTMHGVQVVRCTSDGSWLPYPPQCRPVKCG

PLT----NPHNGKVILESKVYQSQVHYICHDGYSTNQPRSRTCLANGTWSEKEPQCSPNP

CQALPRIPHVIYPADKTRFVVGDFVQLECENGFQPSSEGMAECLPDQTWSLTEFSCDPLE

CLPPESPENGHAEYRTLFFGSIVTYTCNAGYTLLGRATSSCNANQTWSNPLPLCHLVTCP

DLPASENMVYLSTSNAVGDAVTISCSNGFKLVGTSRILCTSEGTWSHSLPRCEADGCLPP

PVIDNGRMFGESFSPGDVLSFRCNRGFYRVGSSSIRCMSDNTWNDSIPKCIAFGSLASLE

RPVCVLPCLHGGKCVGPYSCECPYGFTGSRCEH--VLCTTRCIGGARCKTRHP-----NC

RT-----

>L variegatus

-------------------MMGMYYLINILLLFMCFEVSYERRL------------PVRA

PDLKLLLDSGINVS----FPDEHRQLDF---ADYPQADGLASQPRPADDPLP-LPLLQSL

SYLNIDLERSHHELYEALWRNADVPMDTTSATKIDVLGYLLKKQVEKLRRVSNGQVELVF

LVDSSASVGIENFFNELKFVKKLLADFTVAPDATRVAIITFSSKHRVELNVNQLDNSIPG

KHHHKCALLNEDLPMITYVGGGTYTKGAFELAQKILQGARGNSTQAVFLLTDGLSNGPNP

VPVAASLKEDGVEVFTFGIRDGYIPELLEMASEKKDEHCYILDSFAEFEALARRALHE--

------------ASNPCQNGATCVDGI---------NGYLCICLPGYEGDNCEIEIDECE

SNPCQNGATCDDGINGYFCCCCPIGSYKREALPGGINTCLPCPDQNHVSPLNNTSPNMCV

CKDGYRWDI--ATINEC-VIHCPLLQRPENGEFVECSNVVNGACAIRCNRGYEVTGSSIR

LCFSNGSWSGEDFDIRVKTCEVLTAPLNGSLQCTSDDHAFGTVCAATCDTGFKLIGSQSR

RCLPIAHWDGLPVHCRGMNGATCDDIINGY-----CCCCLPGYEGINCEINIDECSSNPC

QNGATCIDGINGYCCCCLPGYE-----GVTCTDYMPPSLECPSDINTVTSPHMDRVVLEW

EVPRPIDNSGEMPLLHALPNVYPPWTFPLGHWNITYSATDQSGNIAQCVFSVIVEGRDCQ

NGATCIDLINGYRCNCLPGF--EGRNCEINIDECLSNPCLNGA-----TCDDGINGYECC

CLPGFDGVCCEINIDEC--YLLISENLCEKPRDPVGGSAICFHTTQGMNCSVTCGPGYAM

STRPRNQYFCALNGTWYPDGKVPWDDCAETHHANNVLQPMTFLYETSKCDSVFARNMEKL

LATNLEEPVRDECSSNPCQNGATCIDLINGYDPIRFLHGASSSLQQSGSGSDSRLALQGG

GEVSLTAQRLQILQSPNLFVNIVVNGDECSSSPCQNGATCEQTYQMVVQVAASVPGQIQS

NLANGTIVSSTNRTLDRSFQIDRITVYREDIQPECINCEINIDECSSSPCVQCIPGTYFD

MMTSSCEYCEIGQYQNLIGQLTCIHCPNGTSTVSTKSTSLADCK-----GPPGFEGVH-C

EINIDECASN---PCLNGATCIDGINGYICICLPGFEGVNCEINIDEAYCPRGKTSANGL

TPCLPCPAGTFKMSQRSSYCLLCPEDYPLSNPGSITIIYCRNSYKCQCPAGFNGTLCEID

IDECSSNPCQNGATCIDLINGYTCECLPGFEGTNCETDIDECSSNPCQNGATCIDLINGY

TCECAPGFTGTNCEIDIDECSSNPCQNGATCVDLINGYTCQCAPGFTGTNCEIDIDECSS

NPCQNGATCVDLINGYTCQCAPGFTGTNCEIDIDECSSNPCQNGATCIDLINGYTCQCLP

GFEGTNCEINIDECASNPCLNGATCVDLINGYTCQCAPGYEGTNCETEINECASMPCQNG

GVDDYVLLEHDFPTLTAVTISFWMYTDNEGNYGTVFSYAVSDTIDNALTIKDYTGFVLSV

NEENIVSDITGNDGRWHHIVATWDSNGGTYTMYKDGVLEANGTGLASGTSVPGGGKLIIG

QDQDALGGGFAHIESFIGEITFLTVWDRVLGADEIYELGRTCEKPSDVIIAWPDFLSGIE

GQIKQVNNTFCQGCPRPTMPEMGFVKGYLPGRPLTSVTYHCNLGFEMPRHEVRRAECQIS

GEWSPGDRAYCDIINCGYPGHLKHGYVIGRMYLYGDQVFYTCVENFTLVGPLTRQCMENG

YWSAFTPQCRIIYCREPQQVPNAVIFSSEGARPVHMAFPHQNATYQCAQGYRLIGSPNVT

CQIDGEWTAPPTCRIVTCPPLASPLNSMLITTNGSVFTSIAEYRCITGYRMTGANVLQCQ

ETGSWDNPIPTCTIMSCGHPPDVSHATSSYEGVTYGNSVVYACHDGYEADWTVSHCGANG

QWEPSPPECRRIQCSMPPAIANGSMTGGDYRFNSSVTYDCDHGYIINGDREISCMSNGQW

SSSPPFCQAVPCGKPPEMDIHQPLVDQVTEFTYGMTVRYTCKIGYLFASDIIELTCGADG

EWIGTLSDCDPISCGAPNEIPNSSFEGEDYSFNHTVTYYCKTGFIRSGDPVVRCLASGQW

MESSVRCDIIQCEDPPAVLHAVVSHERLYSESVTYSCDPGYKIAGQAFRICGNQGIWAGE

LPTCIPVTCPPPPDLDNGFISVKNDSYGSSVTYSCNPGYTLQGEALRQCLSDGSWSGISP

TCIPVKCPPPPTPQNGSVTLDNLSYGSQATYSCDDGYTLSGQASRQCSANGSWSQEPPDC

T--------------PIECGNPPLIEHGIYIGKNFTFNSTLVYFCADGFIFGEDVVSMET

TCQSDGRWSEEVIECEAVTCGMPPIPPHSIIIGSNFSYL---------------------

-----------------------------------------------DSVQYECELGFVP

SGEGMVECLPDGSWS-TVEPSCDPLECPRPEAPENGHAEYRNLSFGSIVTYSCDTGYTLS

GQAMAQCDSSGQ-------------WS-EINIDECCSHPCQNGATCCD-MINGYGEGKFV

YGSIVRYDCHQGFKLPGVHKMRCSQYGI-----WDPLNITCIPITCPPVLPMDNGFISVK

SNSYGESVTFSCEEGFSLVGNARLLCEEEGQWSGDLPRCIP----VTCDPPLPPDNGFIQ

SKSVSYGSSVVYACNNGFELVGAAFLRCTGDGQWSGDLPRCIPVTCGPPPTPQNGNVILP

NDSYGSQVTYQCDLGYELVGPASRQCLSNGTWSGSQPDCIPIPCAAPEAIPHGIYIQHKT

VQYGSQIEYACDPGYKIAGQSRRICQADGIWSGPQPECIPVSCPPPPMPAHGIAILINDK

YESEATYQCSPGYRLDGPTSRQCLSNGSWSFSNHNCRVIECPSPQPIEHGTFAMNTQVP-

-----------IYKSKLTYTCDVGYTVRGDHILTCDEQGSWSNPLPSCQPVSCPQPPILY

HGIVSFINTSYLSKVTYSCNPGYTLAGAAFRIC-------------TSNGSWSGEQPTCI

PVSCGPPPDPLNGNVTLPNDKYESNATYSCSLGYTLDGPRSRQCLSNGSWSGSTPDCQPI

SCAHPEDIEHGIIISHSNFVILDKVQYSCEPGYKISGQAMRICDPDQSWSGSLPSCIPVS

CPPPPALENGHVSYRADSYGSIVTYSCDTGYTLAGQAFRICTSNGSWSGEQPTCIPVSCG

PPP----DPAHGNVTLPNDKYESNATYSCSLGYTLVGPRSRQCLSNGTWSGSTPDCQPIP

CAALPLIPHAIYPPDKTNFVVGDFVQLECENGFQPSAEGMVECLPQQSWSTVEPSCIPIE

CPPPPAPDNGIASYKATSYGSSVTYDCDTGYTLVGQAMLSCDENGQWSGDLPRCEPVSCD

PLPNPQHGFVISNSNSYGSQVVYACDNGYELVGARLRTCTANGSWSGNPPRCQPVSCGPL

PLPQHGIVISESNSYGSQVFYSCNDGYELVGSRSRTCQLNGTWSGSTPRCIAFGSIAALE

RPVCVLPCLHGGKCVGPYTCQCPYGFTGSRCSISCHLSATII-------SSLP-----RC

SCHYLYH

>M_musculus

------------------------------------------------------------

------------------------------------------------------------

------------------------------------------------------------

------------------------------------------------------------

------------------------------------------------------------

------------------------------------------------------------

------------------------------------------------------------

------------------------------------------------------------

------------------------------------------------------------

------------------------------------------------------------

------------------------------------------------------------

---------------------------------MVSPVLA--------------------

------------------------------------------------------------

------------------------------------------------------------

------------------------------------------------------------

------------------------------------------------------------

------------------------------------------------------------

------------------------------------------------------------

------------------------------------------------------------

------------------------------------------------------------

------------------------------------------------------------

------------------------------------------------------------

-------------------------------------------------------LFSAF

LCHVAIA-----------------------------------------------------

------------------------------------------------------------

------------------------------------------------------------

------------------------------------------------------------

------------------------------------------------------------

--------------------------------------GRICPKPDDL------------

--------PFATVVPLKTS--------YDPG---EQIVYSCK------------------

------------------PGYVSRG-----------------------------------

------------------------------------------------------GMRRFT

CPLTGMWP---------------------------------------------INTLRCV

--------------------PRV-------------------------------------

-------------CPFAGILENGIVRYTSFEYPKNISFACNPGFFLNGTSSSKCTEEGKW

SPDIPACARITCPPPP-----------VPKFA----------------------------

----LLKDYRPSA-------------GNNSLYQDTVVFKCLPHFAMIGNDTVMCTEQGNW

TR----------------------------------------------------------

LPECLEVKCPFPPRPENGYVN---------------------------------------

------YPAKPV----------LLYKDKATFGCHETYKLDGPEEAECTKTGTWSFL-PTC

R---------------ESCKLPVKKATVLYQGMRVKIQEQF----KNGMMHGDKIHFY--

-CKNKEKKCSYTVEAHCRDGTIEI--PSCFKEHSSLAFW---------------------

------------------------------------------------------------

------------------------------------------------------------

--------KTDA-------------SE---------------------------------

------------------------------------------------------------

------------------------------------------------------------

------------------------------------------------------------

----------------------LTPC----------------------------------

------------------------------------------------------------

------------------------------------------------------------

------------------------------------------------------------

------------------------------------------------------------

------------------------------------------------------------

------------------------------------------------------------

------------------------------------------------------------

------------------------------------------------------------

------------------------------------------------------------

------------------------------------------------------------

------------------------------------------------------------

------------------------------------------------------------

------------------------------------------------------------

-------

>SIG

------------------------------------------------------------

------------------------------------------------------------

------------------------------------------------------------

------------------------------------------------------------

------------------------------------------------------------

------------------------------------------------------------

------------------------------------------------------------

------------------------------------------------------------

------------------------------------------------------------

------------------------------------------------------------

------------------------------------------------------------

------------------------------------------------------------

------------------------------------------------------------

------------------------------------------------------------

------------------------------------------------------------

------------------------------------------------------------

------------------------------------------------------------

------------------------------------------------------------

------------------------------------------------------------

------------------------------------------------------------

------------------------------------------------------------

------------------------------------------------------------

------------------------------------------------------------

------------------------------------------------------------

------------------------------------------------------------

------------------------------------------------------------

------------------------------------------------------------

------------------------------------------------------------

------------------------------------------------------------

------------------------------------------------------------

------------------------------------------------------------

------------------------------------------------------------

------------------------------------------------------------

------------------------------------------------------------

------------------------------------------------------------

------------------------------------------------------------

------------------------------------------------------------

------------------------------------------------------------

----+-------------------------------------------------------

---------------------------------------------------+--------

------------------------------------------------------------

------------------------------------------------------------

------------------------------------------------------------

------------------------------------------------------------

------------------------------------------------------------

------------------------------------------------------------

------------------------------------------------------------

------------------------------------------------------------

------------------------------------------------------------

------------------------------------------------------------

------------------------------------------------------------

------------------------------------------------------------

------------------------------------------------------------

------------------------------------------------------------

------------------------------------------------------------

------------------------------------------------------------

------------------------------------------------------------

------------------------------------------------------------

------------------------------------------------------------

------------------------------------------------------------

------------------------------------------------------------

------------------------------------------------------------

APOO

>H sapiens

--MFKVIQRSVGPASLSLLTFKVYAAPKKD------SPPKNSVKVDELSLYSVPE-GQSK

YVEEARSQLEESISQLRHYCEPYTTWCQETYSQTKPKMQSLVQWGLDSYDYLQNAPPGFF

PRLGVIGFAGLIGLLLAR-GSKIKKLVYPPGFMGLAASLYYPQQAIVFAQVSGERLYDWG

LRGYIVIEDLWKENFQK-----------PGNVKNS-------------------------

-------------PGTK-------------------

>H glaber

--MFKVLQKSVGLASLSLLTFRVYAAPKKD------WPRKNSMKIDELSLYSVPE-GQPK

YMEEPRTQLEESISQLRHHCEPYTSWCQETYSQTKPKMQSLVQWGLDGYEYLQNAPPGFF

PRLGVIGFAGLVGLVFTR-GSKIKKLVYPPVFMGLAASIYYPQQAITFVQVSGEKLYDWG

LRGYIVVEDLWKENFQK-----------PGNVKSS-------------------------

-------------PGNK-------------------

>M_brandtii

MAAVRMRKLTIMPAGLIFASISVHAAKEEE-------SKKQLMKPEQLPIYASPP-LQSK

YVEEQPGNLQMGFASIRTTTGRYIGWCKGVYVFVKNGIMDTVQFGKDAYVYLKDPPRDFL

PKIGVITVSGLAGLVSARKGSRFKKIAYPLGLATLGATVCYPVQSVIIAKVTGKKAYATS

QKMYEAVKSLWTKDNKKESLPEPKEKIKPENSAETEIPAKTAHDPKHSVGLPTELRSEMK

TKSEFTSGATQFMPDPKLMDHGQSHPEDVDMYSTRS

>S franciscanus

IVLFYVIHLRL--------------------------PSYQQKKPKDLPLYSEPKQTEYQ

LIMEGPSALREMVGTVREQVWRFSDTFNVSNEYTGCFKDPL---GHSVVKFIK-TEEGFY

PRAGVISIAGLAGLVLARRGGFIRKVLYSGGLMTATASLCYPYQAVRI----GKAEIEWV

KTK---AADLYKGDTAT-----------PAVSLDINIFEKALFWPG----------IICP

PIQNHIESSAQIQPGLYLMQYSALCGAHKAQ-----

>S_purpuratus

MSRQVFVALRYGSMPMLGLGLLQVKAGENTVGGEDMTPVTCLVKPKDLPVYSEPEQTEYQ

LIPEGPSALRDMVGTVREQIWRFSDTFNGAISFTQRTYKSAEQTTQSVVKFIK-TEEGFY

PRAGVISIAGLAGLVLARKGGFFRKVLYSGGLMTATASLCYPYQAVRI----GKAEIEWV

QTK---AADLYKGATST-----------PAVEKVAATEAKTEEDTK----------AETS

SSEESAEKVEESKSGTSLEDHGQSKPEDKDMYTTRS

>L variegatus

-KIQVFVALRYGSMPM----------------------------------LGL-------

-----------GLLQVRAGESPGELYFEGVIQFIK-------------------TEEGFY

PRAGVISIAGLAGLVLARKGGFIRKVVYSGGLMTATASLCYPYQAVRI----GKAEIEWV

KTK---AADLYKGDTSS-----------PAVSLEIELFDAL-------------------

-------------QALK-------------------

>M musculus

--MFKVIQRSVGPASLSLLTFRVYAAPKKD------SPHKSYMKIDELSLYSVPE-GQSK

YVEEPRTQLEENISQLRHHCEPYTSFCQEIYSHTKPKVDHFVQWGVDNYNYLQNAPPGFF

PRLGVIGFAGFVGLLFAR-GSKIKKLVYPPFFMGLGASVYYPQQAITIAQITGEKLYDWG

LRGYIVIEDLWKQNFQK-----------PGNVKNS-------------------------

-------------PGNK-------------------

APP

>H sapiens

----------MLPGLALLLL-------------AAWTARA-----LEVPTDGNAGLLA--

--EPQIAMF-------CGRLNMHMNVQNGKWDSDPSGTKTCIDTKEGILQYCQEVYPELQ

ITNVVEANQPVTIQNWCKRGRKQCKTHPHFVIPYRCLVGEFVSDALLVPDKCKFLHQERM

DVCETHLHWHTVAKETCSEKSTNLHDYGMLLPCGID-KFRGVEFVCCPLAEESDNVDSAD

AEEDDSDVWWGGADTDYADGSEDKVVEVAEEEEVAEVEEEEADDDEDDEDGDEVEEEAEE

PYEEATERTTSIA-TTTTTTTESVEEVVREVCSEQAETGPCRAMISRWYFDVTEGK-CAP

FFYGGCGGNRNNFDTEEYCM--AVCGSAMSQSLLKTTQEPLARDPVKLPTTAASTPDAVD

KYLETPG--DENEHAHFQKAKERLEAKHRERMSQVMREWEEAERQAKNLPKAD-------

KKAVIQHFQEKVESLEQEAANERQQLVETHMARVEAMLNDRRRLALENYITALQAVPPRP

RHVFNMLKKYVRAEQKDRQHTLKHFEHVRMVDPKKAAQIRSQVMT-HLRVIYERMNQSLS

LLYNVPAVAEEIQDEVD-------------ELLQKEQNYSDD------------------

------------------------------------------------VLANMISEPRIS

YGNDALMPSLTETK------TTVELLPVNGEFSLDDLQPWHSFGADSVPANTENEVEP--

-----VDARPAA---DRGLTTRPGSGLTNIKTEEISEVKMDAEFRHDSGYEVHHQKLVFF

AEDVGSNKGAI------------------IGLMVGGVVIATVIVITLVMLKKK-QYTSIH

HGVVEVDAAVTPEERHLSKMQQNGYENPTYKFFEQMQN

>M_brandtii

MLKAGLIHNGELARIELFLLWLPSPAFPGVSPPGPWIAITNPNLFFQVPTDGNAGLLA--

--EPQVAMF-------CGKLNMHMNVQNGKWESDPSGTKTCIGTKEGILQYCQEVYPELQ

ITNVVEANQPVTIQNWCKRGRKQCKTHGHMVIPYRCLVGEFVSDALLVPDKCKFLHQERM

DVCETHLHWHTVAKETCSEKSTNLHDYGMLLPCGID-KFRGVEFVCCPLAEESDNVDSAD

AEEDDSDVWWGGADADYADGSEDKVVEVTEEEEVADVEEEEAEDDEDDEDGEEVEEDTEE

PYEEATERTTSIATTTTTTTTESVEEVVREVCSEQAETGPCRAMISRWYFDVTEGK-CAP

FFYGGCGGNRNNFDTEEYCM--AVCGSVMSQSLLKTTQEPLPRDPVKLPTTAASTPDAVD

KYLETPG--DENEHAHFQKAKERLEAKHRERMSQVMREWEEAEHQAKNLPKAD-------

KKAVIQHFQEKVESLEQEAANERQQLVETHMARVEAMLNDRRRLALENYITALQAVPPRP

RHVFNMLKKYVRAEQKDRQHTLKHFEHVRMVDPKKAAQIRSQVMT-HLRVIYERMNQSLS

LLYNVPAVAEEIQDEVD-------------ELLQKEQNYSDD------------------

------------------------------------------------VLANMISEPRIS

YGNDALMPSLTETK------TTVELLPVNGDFSLDELQPWHPFGVDSVPANTENEVEP--

-----VDARPAA---DRGLTTRPGSGLTNIKTEEISEVKMDAEFRHDSGYEVHHQKLVFF

AEDVGSNKGAIIGLMVFFAEDVGSNKGAIIGLMVGGVVIATVIVITLVMLKKK-QYTSIH

HGVVEVDAAVTPEERHLSKMQQNGYENPTYKFFEQMQN

>H_glaber

----------MLPGLSQLLI-------------LCASSRA-----TEVPTDGNAGLLA--

--EPQIAMF-------CGKLNMHMNVQNGKWEPDPSGTKTCIGSKEGILQYCQEVYPELQ

ITNVVEANQPVTIQNWCKRSRKQCKTHPHFVIPYRCLVGEFVSDALLVPDKCKFLHQERM

DVCETHLHWHTVAKETCSEKSTNLHDYGMLLPCGID-KFRGVEFVCCPLAEESDNIDSAD

AEEDDSDVWWGGADTDYADGSEDKVVEVTEEEEVADVEEEEADDDEDVEDGDEVEEEPEE

PYEEATEKTTSIA-TTTTTTTESVEEVVREVCSEQAETGPCRAMISRWYFDVTEGK-CAP

FFYGGCGGNRNNFDTEEYCM--AVCGSVMSQSLLKTTGEPVSQDLVKLPTTAASTPDAVD

KYLETPG--DENEHAHFQKAKERLEAKHRERMSQVMREWEEAERQAKNLPKAD-------

KKAVIQHFQEKVESLEQEAANERQQLVETHMARVEAMLNDRRRLALENYITALQAVPPRP

RHVFNMLKKYVRAEQKDRQHTLKHFEHVRMVDPKKAAQIRSQVMT-HLRVIYERMNQSLS

LLYNVPAVAEEIQDEVD-------------ELLQKEQNYSDD------------------

------------------------------------------------VLANMISEPRIS

YGNDALMPSLTETK------TTVELLPVNGEFSLDDLQPWHPFGVDSVPANTENEVEP--

-----VDARPAA---DRGLTTRPGSGLTNIKTEEISEVKMDAEFRHDSGYEVRHQKLVFF

AEDVGSNKGAI------------------IGLMVGGVVIATVIVITLVMLKKK-QYTSIH

HGVVEVDAAVTPEERHLSKMQQNGYENPTYKFFEQMQN

>S franciscanus

----MENFIKMATNMAFYLV-------------LLFAI-------LSVASAGYLQALAAG

AFDPSSSRFELRVAVRKGFFNKYADILSGQWLTDTSAEYTSDATE--VLNYCKTRKYRYC

RPKYAHPIKP-YISRFPSCATDHQ-QHPHPYLFKIPKVGPFESDALMVYNVCKFYHKHDE

NTCREPEYWKGVS--------LLISFSFRWIPRVIYLQYKRVIFPIYQISC---------

-----------------------SYIMSYRGASGTQGQKKELEAS---------------

------------------------------------------CYWSQMYLKAKIPK-LRI

-----CACQSPPISASCYCYIPAVAKPQITSNILD--------DP---------------

-YFSEQGLAGRVEKKEYSNAKGRLSSNQKAKMARVMQQWQEAQEHYETLKAKDPQAAEQM

RKEMTQRFEQTIGTMETENAEETEELREAHQVHIAVSLREKINASYEAYRFTVDVPKPQS

KKILHSLRRYLHAIQKTRQHYITHYKHLRKTDPMKADQHKRFTLN-KLKTLDFEVAQSVD

LLQNLPDLYAELKPKVSIYSSHKICIHPQRPIQHKVHKHPQQ---------------RIS

QPSRKSLSPYIYSIQYKLYARIQIHIMLYRSQSSASNYSYSLFILQEYFTCRIICAPTVS

KSFETKPSTMAKTSPAQTKPSPSQTKPRTSTATQTEPEPPTPSRDTEPEIEQETEIQVPA

EKVQIVAGKPIESYLDDELTAPLDTEVVKPQTLIIAQKPKAKVEKSAAAARVEKQRLV-S

RQDVNSIDQSSTKKIP----SLRYEPMMAFGLACGVLAVATVLVIAVLIVRRKTRRTPVN

SGFTEIDPNLTVEQRHIVAMQQNGYENPTYKYFD-MQ-

>S purpuratus

----MENLIKMASNMALYLV-------------LLFAI-------LSVASAGYVEALAAG

AFDPSSSRFELRVAVRKGYFNKYADILSGQWLTDTSAEFTADATE--VLNYCKTKYPDTE

INNIVDSSQPILIDQWCPVNGDAC-TSSAKVTYYRCLVGPFESDALMVYNVCKFYHKHDE

NTCREPEYWKGVADEDCRGKKMGINSTGMLLPCQTD-KFKGVEYTCCPPP----------

-----------------------TYRPMTAGEEETEVEEEEEEAE---------------

-------------------------------AEAAAAGGETQAQVQEE-LPVQAPA-PAP

--------ERDNV----------VAKPQITSNILD--------DP---------------

-YFSEQGLAGRVEKKEYSNAKGRLSSNQKAKMARVMQQWQEAQEHYETLKAKDPEAAEQM

RKEMTQRFEQTIGTMETENAEETEELREAHQVHIAVSLREKINASYEAYRFTVDVPKPKS

KKILHSLRRYLHAIQKARQHYITHYKHLRKTDPMKADQHKRFTLN-KLKTLDIEVAQSVD

LLQNLPDLYAELKPKVD-------------ALLDSTHDTPEDAALIRATEEEEEVEEEEV

MIAQAKVTEAPPAPQRTELEPVAEITEPEVADEVISQAEEEELEEVEEFLEEDTEAPTVS

RSFETKPSTLAKTSPAQTKPSPSQTKPRTSTATQTEPEPPTPSRDTEPEIEQETEIQVPA

ENVQIVAVKPIESYLDEELTAPLDSDVVKPQTLIIAQKPKAKVEKSAAAARMEKQRLV-G

RQDVNSIDNSVTKKRM----SLRYEPMMAFGLACGVLAVATVLVIAVLIVRRKTRRTPVN

SGFTEIDPNLTVEQRHIVAMQQNGYENPTYKYFD-MQ-

>L variegatus

----MENFIKMASNVAFYLV-------------LLLAI-------LSVASAGYVQALAAG

AFDPSSSRVELRVAVRKGYFNKYADIISGQWLTDTSAEYTSDPTE--ILNYCQQKYPNAE

VNNIVDSSQPTRIDQWCPVNGDEC-TSSAKVTYYRCLVGPFESDALMVYNVCKFYHKHDE

TTCREPEYWKGVSDEDCRAKKMGINSTGMLIPCKTD-KFK--------------------

---------------------------YKREENMKCIQGHIIKESVS-------------

-------------------------------CPARSKAPEIEYQIINYYIKYTRAQACSP

---------ESQIQHIRYEY--SVDEPQVKSNILD--------DP---------------

-YFSEQGLTGRVEKKEYANAKSRLSANQKAKMARVMQQWQEAQEHYETLKAKDPEAAEKM

RKEMTERFEQTIGTMETENAEETEELREAHQVHIEVSLREKINASYEGYRFTVDVPKPKS

CR----LREIWIAI--IIQIYLIY-----------ACEYSKYSLPINIKCQPPQICQAAS

LM----DIIIQIQPS---------------QVLHRMQKKIQD------------------

-----------------------------------------------PPKNKKTRFLTVS

KSTETKPSTQAKTSPAQTKPSPMQTKPRTSTATHTEPEPPTPSRVTGSEVEQETEIQVPA

EKVQILAVKPIESYLDEELTAPVDTDVVKPQTLIVAQKPQAKVEKSAAAARVEKQRLV-S

RQDVNS----VTKKRM----QLRYEPMMAFGLACGVLAVATVLVIAVLIVRRKTRRTPVN

SGFTEIDPNLTVEQRHIVAMQQNGYENPTYKYFD-MQ-

>M_musculus

----------MLPSLALLLL-------------AAWTVRA-----LEVPTDGNAGLLA--

--EPQIAMF-------CGKLNMHMNVQNGKWESDPSGTKTCIGTKEGILQYCQEVYPELQ

ITNVVEANQPVTIQNWCKRGRKQCKTHTHIVIPYRCLVGEFVSDALLVPDKCKFLHQERM

DVCETHLHWHTVAKETCSEKSTNLHDYGMLLPCGID-KFRGVEFVCCPLAEESDSVDSAD

AEEDDSDVWWGGADTDYADGGEDKVVEVAEEEEVADVEEEEADDDEDVEDGDEVEEEAEE

PYEEATERTTSTA-TTTTTTTESVEEVVREVCSEQAETGPCRAMISRWYFDVTEGK-CVP

FFYGGCGGNRNNFDTEEYCM--AVCGSVSTQSLLKTTSEPLPQDPDKLPTTAASTPDAVD

KYLETPG--DENEHAHFQKAKERLEAKHRERMSQVMREWEEAERQAKNLPKAD-------

KKAVIQHFQEKVESLEQEAANERQQLVETHMARVEAMLNDRRRLALENYITALQAVPPRP

HHVFNMLKKYVRAEQKDRQHTLKHFEHVRMVDPKKAAQIRSQVMT-HLRVIYERMNQSLS

LLYNVPAVAEEIQDEVD-------------ELLQKEQNYSDD------------------

------------------------------------------------VLANMISEPRIS

YGNDALMPSLTETK------TTVELLPVNGEFSLDDLQPWHPFGVDSVPANTENEVEP--

-----VDARPAA---DRGLTTRPGSGLTNIKTEEISEVKMDAEFGHDSGFEVRHQKLVFF

AEDVGSNKGAI------------------IGLMVGGVVIATVIVITLVMLKKK-QYTSIH

HGVVEVDAAVTPEERHLSKMQQNGYENPTYKFFEQMQN

ATP6

>H sapiens

MNENLFASFIAPTILGLPAAVLIILFPP--LLIPTSKYLINNRLITTQQWLIKLTSKQMM

TMHNTKGRTWSLMLVSLIIFIATTNLLGLL-PHSF-TPTTQLSMNLAMAIPLWAGTVIMG

FRSKIKNALAHFLPQGTPTPLIPMLVIIETISLLIQPMALAVRLTANITAGHLLMHLIGS

ATLAMST---INLPSTLIIFTILILLTILEIAVALIQAYVFTLLVSLYLHDNT

>M musculus

MNENLFASFITPTMMGLPIVVAIIMFPS--ILFPSSKRLINNRLHSFQHWLIKLIIKQMM

LIHTPKGRTWTLMIVSLIMFIGSTNLLGLL-PHTF-TPTTQLSMNLSMAIPLWAGAVITG

FRHKLKSSLAHFLPQGTPISLIPMLIIIETISLFIQPMALAVRLTANITAGHLLMHLIGG

ATLVLMN---ISPPTATITFIILLLLTILEFAVALIQAYVFTLLVSLYLHDNT

>S purpuratus

MTASILGQFFPETLFFIPMNVFSMAFCLSWLVFIYPVNWAPSRFQSIWLGFRSNILEMIF

QNTSPNTAPWAGLIAGVFVLILLVNVLGLFPPYAFQSPTSNISLTYSLGFPLWMAINILG

FYLAFNSRLSHLVPQGTPSALIPLMVWIETLSLFAQPIALGLRLAANLTAGHLLIFLLST

AIWLLSSSLMISVP----ILIIFILLFVLEIGVACIQAYVFTALIHFYLQQNI

>L variegatus

VTPRIFGQFFPETLFFIPMNVFSMVFALSWLAFIYPTKWAPSRFQSVA-SFRENVLEMIF

QNTRPSTAP-AGLITAVFVIILSVNVLGLF-P-AF-TATRHISLTYRLGFPL-MAVKILG

FYLAFKRRLSHLVPQGTPNALIPLMV-IETLRLFAQPIALGLRLAANLTAGHLLIFLLST

AILLSSS-PMIRIP----IFVIFVLLFVLEIGVACIQAYVFTALVHFYLQQNI

>S franciscanus

VTPRIFGQFFPETLFFIPMNIFSMVFALS-LIFIYPTK-APSRFQSIWLRFRENILEMIF

QKTRPKTAP-AGLITGIFII-------------AF-TATRHISLTYRLGFPL-MAVKILG

FYLAFNSRLSHLVPQGTPSALIPLMV-IETLSLFAQPIALGLRLAANLTAGHLLIFLLST

AILLSSS-LMVSIP----IFIIFVLLFVLEIGVACIQAYVFTALVHFYLQQNI

>259020178__H_glaber 109 origGI 343403917

MNENLFAPFSTPTLMGLPIIIIIMMFPS--MLLSRPNRLINNRLTSLQQWAIQMTLKQLM

TMHNTKGRSWTLMLTSLILFISSTNLLGLL-PYTF-TPTTQLSMNLGMAIPLWAGTVILG

FRHKTKKSLAHFLPQGTPNPLIPMLVLIETISLFIQPVALAIRLTANITAGHLLMHLISK

AVLTLIS---INITTALITFTILILLTMLEFAVAMIQAYVFTLLVSLYLNDNT

ATP8

>H sapiens

-MNFALILMINTLLALLLMIITFWLPQLNGYMEKSTPYECGFDPMSPARVPFSMKFFLVA

ITFLLFDLEIALLLPLPWALQTTNLPLMVMSSLLLIIILALSLAYEWLQKGLDWTE

>226346498__H_glaber

-MNMTISLLMNFSLASILMLIAFWLPQMDAYAEKLSPYECGFDPMGSARLPFSMKFFLIA

ITFLLFDLEIALLLPLPWASQTNNLTLMLTVSLMLISVLALGLFYEWSQKGLEWDK

>S franciscanus

MTTIIFLFGITIAVATVFGLAAHALPRRAGDKEKNSPYECGFDPLNSARLPFSFRFFLVA

ILFLLFDLEIALLFPLPAAELITNPSTLIPISLIFMIILALGLVFE-VKGGLE-AE

>226346498__S_purpuratus

MTTIIFLFSITIAVAVVLGLAAHALPNRTSDSEKSSPYECGFDPLNSARLPFSFRFFLVA

ILFLLFDLEIALLFPLPAASLITPPSTLIPISMVFMVILTLGLVFEWINGGLEWAE

>L variegatus

MTPIIFIFLITIVVAIVFAAAAHILPKRSKDREKTSPYECGFDPLNSARLPFSFRFFLVA

ILFLLFDLEIALLFPLPAAKSSTPPSILIPISLIFMIILALGLVFEWIKGGLE---

>M musculus

-MNLYTVIFINILLSLTLILVAFWLPQMNLYSEKANPYECGFDPTSSARLPFSMKFFLVA

ITFLLFDLEIALLLPLPWAIQTIKTSTMMIMAFILVTILSLGLAYEWTQKGLEWTE

BACE1

>M brandtii

----------MTVRRRWQG------------------RPPISEDTGDLGVGEAGKQAQRR

RRSFGPVPDHKVYSEDQLCKLTYSAHISLYNYTGCILSFPEKSCLQILVDTGSSNFAVAG

APHPYIDSYFDTQRSSTYRP--KGFDVTV-------KYTQGSWTGSVGEDLVTITKGFNT

SFLVNIATIFESENFFLPGIQWNGILGLAYAALAKPSSSLETFFDSLVTQAGIPNVFSMQ

MCGAGLPVDGSG----------TNGGSLVLGGIEPTLYKGDIWYTPIKEEWYYQIEILKL

EVGGQSLNLDCREYNADKAIVDSGTTLLRLPHKVFDAVVEGVARASL--IPEFSDGFWTG

SQLACWANSET---PWSYFPKISIYLREENSSRSFRITILPQLYIQPMMR-AGLNYECYR

FGISPST--NALVIGATVMEGFYVIFDRARKRVGFAASTCAEIAGTP------VSEISGP

FSTD-DIASNCVPTLPLNEPILWIVSYTLMSVCGIILLTLIILLL---LPVRC-------

---RHGPRDPEVVNDESSLVRHRWK

>H glaber

----------MAPALPWLLLWVGSGVLPAHGTQHSI-RLPLRSG---LAGAPLGLRLPRE

TD---EELGRRGSFVEMVDNLRGKSGQGYYVEM-TVGSPPQTL--NILVDTGSSNFAVGA

APHPFLHRYYQRQRSSTYRDLRKGVYV---------PYTQGKWEGELGTDLVSIPHGPNV

TVRANIAAITESDKFFINGSNWEGILGLAYAEIARPDDSLEPFFDSLVKQTHVPNLFSLQ

LCGAGFPLNQSE-------AVASVGGSMIIGGIDHSLYTGSLWYTPIRREWYYEVIIVRV

EINGQDLKMDCKEYNYDKSIVDSGTTNLRLPKKVFEAAVKSIKAASS--TEKFPDGFWLG

EQLVCWQAGTT---PWNIFPVISLYLMGEVTNQSFRITILPQQYLRPVEDVATSQDDCYK

FAISQSS--TGTVMGAVIMEGFYVVFDRAQKRIGFAVSACHVHDEFR------TAAVEGP

FVTL-DMEDCGYNIPQTDESTLMTIAYVMAAICALFMLPLCLMVCQWRC-LRCL------

---RHQHDD---FADDISLLK----

>S franciscanus

----------VVEVYESMVVIVITIKEITVIITIVV-----------IMLLHVGL-----

-----HVVVLVVIIIVIIIIITMIVIMIILIMI-IIVMIMIVIINNNYIDTGSSNFAVAA

ASHNAISTYYRRNESSTYED--QGTYVKV-------PYTQGEWSGDLGQDLVQIASLGNQ

SFQANIAAITESQQFFLNGSRWQGILGLGYAEIARVRTLARSWFRCLTSQTSIQDIFALQ

MCGALASTNDTNLGSSADGPVEEVIGSMNIGGLDPSMYHGTMQYAPLRDEWFYEVIMTDI

KVGNESLGLDCKE-----------VRNFRLYYVLLD-------------MPDVPSNFWTG

MVLMCPTDSTLPNEPYHWFPTLTLDLQSTNQGQAFSLVVSPQVLLIWII--IIIIGVCSA

YKILGAL--QGAVIGAVIMEGFYVVFDRENKRVGFARSTCPGYILCPSYCVAYKIPTTLH

HAQISDAADCGYDRSTSYDPALTVTAYVLAGICLVCLIPVIVFALTHQINKRCKGRRGRG

VVNHHRFDQEGLADNDPDTVP----

>S purpuratus

MHFSLPTSRIVVVVPAAAICIVCVLIETCTAARSHVYTIPLRKGKETSFAETVGEPV---

-----RTNQVNVSVEEQKNNIRGRPGLGYYIEV-DIGTPPQKL--NVLIDTGSSNFAVAA

SSHNAISTYYRRNESSTYED--QGTYVKV-------PYTQGEWSGDLGQDLVQIASLGNQ

SFQANIAAITESKMFFLNDSRWQGILGLGYAEIARPDSSVEPFFDSLTSQTSIQDIFALQ

MCGALASTNDTNLGSSADGPVEEVIGSMNIGGLDASLYHGTMQYAPLRDEWFYEVIMTDI

RVGNDSLGLDCKEYNFDKTIVDSGTTNLRLPVRVFEAITNAIKAHTTKHMPDVPSEFWTG

MNLMCPTDSTSPYEPYHWFPTLTLDLQSTNQGQAFSLVVSPQQYLRRDYD-HEDKKNCFK

FAIAPSTNHAGAVIGAVIMEGFYVVFDRENKRVGFARSTCPGACEKTGTCVGNSPLITEA

FNIDFDASDCGYDRSTSYDPALTITAYVLAAICLVCLIPVIVFALTHQINKRCKGRRGRG

VVNHHRLDQEGLAENEPNSDP----

>L variegatus

------DNKYCISSFIFNKIQLKVIVMPSVINQSGVFFIQYNFEKTIVDSGTTNLRLPER

V----FEATLNVSVDDQKNNIRGRPGLGYYIEV-DIGTPPQKV--SVLIDTGSSNFAVAA

SSHDAISTYYKRNESLRIKH--YPNYIIFAPAFLYEAFLLFYFGIGPSRRLFHIAH--AL

HYYYSICYV-----FFISLS-----IHLSLSLLIQPDSTVEPFFDSLTSQTSIQDIFALQ

MCGALASRNDTSLESSPHQPIEEMKSSQVIGGIDSSLYNGTMQYSPLRDEWFYEVIMTDI

KVGNDSLNLDCKQYNFEKTIVDSGTTNLRLPERVFEATKNAIRAHTAKDMPDVPADFWTG

LVLMCPTDNTLPYEPYHWFPTLTLDLQSTNQGQAFSLVVSPQQYLRRDYD-HEDKKNCFK

FSISPSSNKAGATIGAIVMEGFYVVFDREKKRVGFARSTCPGFSLFF-FLFFPKIYILLP

FFLIIDASDCGFDRSNSYDPALTITAYVLAAICLVCLLPVIVFAVSHQFNKRCKGHGGRG

VVNHHRLNQESLAENDISYPW----

>M musculus

----------MAPALHWLLLWVGSGMLPAQGTHLGI-RLPLRSG---LAGPPLGLRLPRE

TDEESEEPGRRGSFVEMVDNLRGKSGQGYYVEM-TVGSPPQTL--NILVDTGSSNFAVGA

APHPFLHRYYQRQLSSTYRDLRKGVYV---------PYTQGKWEGELGTDLVSIPHGPNV

TVRANIAAITESDKFFINGSNWEGILGLAYAEIARPDDSLEPFFDSLVKQTHIPNIFSLQ

LCGAGFPLNQTE-------ALASVGGSMIIGGIDHSLYTGSLWYTPIRREWYYEVIIVRV

EINGQDLKMDCKEYNYDKSIVDSGTTNLRLPKKVFEAAVKSIKAASS--TEKFPDGFWLG

EQLVCWQAGTT---PWNIFPVISLYLMGEVTNQSFRITILPQQYLRPVEDVATSQDDCYK

FAVSQSS--TGTVMGAVIMEGFYVVFDRARKRIGFAVSACHVHDEFR------TAAVEGP

FVTA-DMEDCGYNIPQTDESTLMTIAYVMAAICALFMLPLCLMVCQWRC-LRCL------

---RHQHDD---FADDISLLK----

CAT

>H sapiens

-MADSRDPASDQMQHWKEQRAAQKADVLTTGAGNPVGDKLNVITVGPRGPLLVQDVVFTD

EMAHFDRERIPERVVHAKGAGAFGYFEVTHDITKYSKAKVFEHIGKKTPIAVRFSTVAGE

SGSADTVRDPRGFAVKFYTEDGNWDLVGNNTPIFFIRDPILFPSFIHSQKRNPQTHLKDP

DMVWDFWSLRPESLHQVSFLFSDRGIPDGHRHMNGYGSHTFKLVNANGEAVYCKFHYKTD

QGIKNLSVEDAARLSQEDPDYGIRDLFNAIATGKYPSWTFYIQVMTFNQAETFPFNPFDL

TKVWPHKDYPLIPVGKLVLNRNPVNYFAEVEQIAFDPSNMPPGIEASPDKMLQGRLFAYP

DTHRHRLGPNYLHIPVNCPYRARVANYQRDGPMCMQDNQGGAPNYYPNSFGAPEQQPS-A

LEHSIQYSGEVRRFNTANDDNVTQVRAFYVNVLNEEQRKRLCENIAGHLKDAQIFIQKKA

VKNFTEVHPDYGSHIQALLDKYNAEKPKNAIHTFVQSGSHLAAREKANL

>H glaber

-MADSRDPASDQMKLWKEQRAAQKPDVLTTGGGNPIGDKLNIITAGPRGPLLVQDVVFTD

EMAHFDRERIPERVVHAKGAGAFGYFEVTHDITKYSKARVFEHIGKKTPIAVRFSTVAGE

SGSADTVRDPRGFAVKFYTEDGNWDLVGNNTPIFFIRDAILFPSFIHSQKRNPQTHLKDP

DMVWDFWSLRPESLHQVSFLFSDRGIPDGHRHMNGYGSHTFKLVNAKGEAVYCKFHYKTD

QGIKNLSVEEAARLSQEDPDYGLRDLFNAIANGNFPTWTFYIQVMTFDQAETFPFNPFDL

TKIWPHKDYPLIPVGKLVLNRNPVNYFAEVEQIAFDPSNMPPGIEPSPDKMLQGRLFSYP

DTHRHRLGPNYLQIPVNCPYRARVANYQRDGPMCVNHNQGGAPNYYPNSFSAPVEQRY-A

LEHTSRCSAEVRRYNSADEDNVTQVRTFYTEVLNEPERRRLCENIAGHLKDAQLFIQKKA

VKNFTDVHPDYGACIQALLDKYNTEKPKNTIHTFVQAGSHLAAKEKANL

>M brandtii

-MGDSRDPASDQMKHWKEQRNTQKPDVLTTGAGIPIGDKLNTMTTGPRGPLLVQDVVFTD

EMAHFDRERIPERVVHAKGAGAFGYFEVTHDITKYSKAKVFEHIGKRTPLAVRFSTVAGE

SGSADTVRDPRGFAVKFYTEDGNWDLVGNNTPIFFIRDAILFPSFIHSQKRNPQTHLKDP

DMVWDFWSLRPESLHQVSFLFSDRGIPDGHRHMNGYGSHTFKLVNANGEAVYCKFHYKTD

QGIKNLSVEEAARLSHEDPDYGLRDLFNAIATGNCPSWTFYIQVMTFSQAESCPFNPFDV

TKVWPHKDYPLIPVGKMVLNRNPVNYFAEVEQLAFDPSNMPPGIEPSPDKMLQGRLFSYP

DTHRHRLGPNYLQIPVNCPYRARVANYQRDGPMCVVDNQGGAPNYYPNSFSAPEHQPKLA

LESSTRVSGDVRRYNTANDDNVSQVRTFYTCVLNEEERKRLCENIAGHLKDAQLFIQRKA

VKNFSDVHPDYGARIQALLDKYNAEKPKNAIHTFMQHGSHLAAREKANL

>S franciscanus

SPNLQARPQYHPPPQ---PIPQGKTDRLTTSTGCPIDNKLATMTAGARGPVLIQDFVFTD

EMSHFGRERIPERVVHAKGGGACGYFETTHDISKYCKAAPFESVGKKTPVAIRFSTVGGE

AGSADTARDPRGFAVKFYSEDGNWDLVGNNTPIFFIRDPIFFPSFIHTQKRNPVTHLQDP

DMFWDFITLRPEATHQVSFLFSDRGTPDGFRHMNGYGSHTFKLRNKDGGYVFCKFHFKCD

QGIKNFNRHRAGELSASDPDYAIRDLYNAIATGNFPSLCLHIQVMTQEQADKHRDNPFDL

TKVWSQKEFPLLPVGKMVLNLNPKNYFAEVEQIAFSPAHMIPGIEPSPDKMLQGRLFSYS

DTHRHRLGTNYLQIPVNCPFAARTRSYQRDGPQCVTDNQGGAPNYFPNSFTGPVDSKN-Y

EQCKFSCPGEAARYETGDDDNYTQAGIFWRDVLSEADREATVDNMASHIKDAAEYLQKRT

VSDNASVF----------------------------------------L

>S purpuratus

--MANRDKAARQLEDHCTA-QKGKSDRLTTSTGCPIDNKLATMTAGARGPVLIQDFVFTD

EMSHFGRERIPERVVHAKGAGAFGYFETTHDISKYCKAAPFESVGKKTPVAIRFSTVGGE

SGSADTARDPRGFAVKFYSEDGNWDLVGNNTPIFFIRDPMFFPSFIHTQKRNPVTHLKDP

DMFWDFITLRPEATHQVSFLFSDRGTPDGYRHMNGYGSHTFKLRNKDGEYVFCKFHFKCD

QGIKNLNRHRAGDLSATDPDYAIRDLYNSIATGNFPSWSLHIQVMTQEQADKHRDNPFDL

TKVWSQKEFPLLPVGKMVLNLNPKNYFAEVEQIAFSPAHMIPGIEPSPDKMLQGRLFSYS

DTHRHRLGTNYLQIPVNCPFAARTRSYQRDGPQCVTDNQGGAPNYFPNSFTGPTDSKS-Y

EQTKFTCPGEAARYETGDDDNYTQAGIFWRDVLSEADREATVDNMASHIKDAAEYLQKRT

TQASATAN----------------------------------------L

>L variegatus

IYEIGICPLCFRLPLY----TVKEKNRLTTSTGCPIDNKLATMTAGARGPVLLQDFVFTD

EMAHFGRERIPERVVHAKGAGAFGYFETTHDISKYCKAAPFESIGKKTPVAVRFSTVGGE

SGSADTARDPRGFAVKFYSEDGNWDLVGNNTPIFFIRDPIFFPSFIHTQKRNPVTHLKDP

DMFWDFITLRPESTHQVSFLFSDRGTPDGYRHMNGYGSHTFKLRNKQGEYVFCKFHFKCD

QGIKNLNRHRAGELSAGDPDYAIRDLYNAIATNNFPSWSLHIQVMTQEQADKHRDNPFDL

TKVWSQKEYPLIPVGKMVLNLNPKNYFAEVEQIAFSPAHMIPGVEPSPDKMLQGRLFSYS

DTHRHRLGTNYLQIPVNCPFAARTRSYQRDGPQCVTDNQGGAPNYFPNSFSGPVDSKD-Y

QQCPFEVSGVAQRYETGDDDNYSQPGIFWRDVLSEADREATVDNMASHIKDAADYLQKRA

VSSLPSSE-----------------------------------------

>M musculus

-MSDSRDPASDQMKQWKEQRASQRPDVLTTGGGNPIGDKLNIMTAGSRGPLLVQDVVFTD

EMAHFDRERIPERVVHAKGAGAFGYFEVTHDITRYSKAKVFEHIGKRTPIAVRFSTVTGE

SGSADTVRDPRGFAVKFYTEDGNWDLVGNNTPIFFIRDAILFPSFIHSQKRNPQTHLKDP

DMVWDFWSLRPESLHQVSFLFSDRGIPDGHRHMNGYGSHTFKLVNADGEAVYCKFHYKTD

QGIKNLPVGEAGRLAQEDPDYGLRDLFNAIANGNYPSWTFYIQVMTFKEAETFPFNPFDL

TKVWPHKDYPLIPVGKLVLNKNPVNYFAEVEQMAFDPSNMPPGIEPSPDKMLQGRLFAYP

DTHRHRLGPNYLQIPVNCPYRARVANYQRDGPMCMHDNQGGAPNYYPNSFSAPEQQRS-A

LEHSVQCAVDVKRFNSANEDNVTQVRTFYTKVLNEEERKRLCENIAGHLKDAQLFIQKKA

VKNFTDVHPDYGARIQALLDKYNAEKPKNAIHTYTQAGSHMAAKGKANL

CETP

>H sapiens

-----------MLAATVLTLALLGNAHAC---------------------SKGTSHE-AG

IVCRITKPALLVLNHETAKVIQTAFQRASYPDITGEKAMMLLGQVKYGLHNIQISHLSIA

SSQVELVEAK-SIDVSIQNVSVVFKGTLKYGYTTAWWLGIDQSIDFEIDSAIDLQINTQL

TCD-SGRVRTD--APDCYLSFHKLLLHLQGEREPGWIKQLFTNFIS-FTLKLVLKGQICK

E-INV-ISNIMADFVQTRAASILSDGDIGVDISLTGDPVITASYLESHHKGHFIYKNVSE

D--LPLPTFSPTLLGDSRMLYFWFSERVFHSLAKVAFQDGRLMLSLMGDEFKAVLETWGF

N---TNQEIFQEVVGGFP-SQAQVTVHCLKMPKISCQNKGVVVNSSVMVKFLFPRPDQQH

SVAYTFEEDIVTTVQASYSKKKLFLSLLDFQITPKTVSNLTESSSES-VQSFLQSMITAV

GIPEVMSRLEVVFTALMNSKGVSLFDIINPEIITRDGFLLLQMDFGFPEHLLVDFLQSLS

-------------------

>H glaber

-----------MLAAALLSLALLSSAHPC---------------------STGPPYV-AD

IVCHITKPAFLVLSQETTKVIHTAFRWASYPDIKGEKAMVLLGHVKYGLHNIQISHLSIA

GSQVELAEAK-SIDISIQNVSVVFTGTLNYGYTSAWGLSIDRFVDFEIDSAIDLXINTQL

ICD-SGRVRTD--APDCHLSFHKLLLYLHGEHEPRWIKRLFTNFIS-FTLKLALKGQICK

E-INV-ISNIMADFVQSRATNILSDGDXGVDISLTGLSVITATYLESHRKGHFIYKSISE

D--LVLPTFSPSLLGDSRMLYFWFSEQVLDSFAKATFQDGHLMLSLMGEKFKAALETQAL

N---INQETFAELFSSFPPSQTQVTVHCLTRPRISCQNKGI-IVSSVMVKFLFPHPDGQH

SVAHTFEEDIITTIQASYSRKKLFLSLLDFQITPKTASNEAESCSEA-VRSFLQSLITTV

GIPEVMSRLQVALTALMNSKGLHFFDLINPEIITQDGSLLLQMDFGFPEHLLVDFLQSLS

-------------------

>M brandtii

-----------MARFAALFLALLAGAHAA-------------------------AEI-PG

CKIRITSKALELLKQEGLRFLEQELETVTIPDLGGHE-----GHFYYNISEVTVTEMQLT

SSELHFQPEQ-ELVLQTSNSSLGLRFRRQLRY---WIFYDGGYINAS-AQGISIHTALQL

SRDPTGRIKVA--NVSCQASVSKLYAAFEG--TFKIVYQFLSTFVT-SGMHFLLNQQICP

V-IHHAGMVLLNSLLDTVPVRSSVDNLVGIDYSLVKDPVVSTSYMDMEFRGAFFPLG-QG

NWSLPNRAVEPQLQEEERMVYVAFSEFFFDSALESYFQAGALQLSLVGDKVPKDLDMLLR

AS--YFGSIVLLQSPAVIDSPLKLELRVVAPPYCTIKPSGTTVSVTASVTVALVPPNQPE

VQLSSMIMDARLSAKMALQGKALRIHLDLRKFRIYSNQSALESLALIPLQAPLKTMLQFG

VMPLLNERTRRGVQIPL----PEGMDFVREVVTNHAGFLTIAADLHFAKGLREVIEKNRP

ANTMDSGVPSAPPPSTAAV

>M musculus

----------MKAGTGPLLSTLLGLLFLS---------------------IQGTGGVNPG

VVARITDKGLAYAAKEGLVALQRELYKITLPDFSGDFKIKAVGRGQYEFHSLEIQNCELR

GSSLKLLPGQ-GLSLAISDSSIGVRGKWKVRK---SFLKLHGSFDLD-VKGVTISVDLLL

GMDPSGRPTVS--ASGCSSRICDLDVHISG--NVGWLLNLFHNQIE-SKLQKVLENKVCE

M-IQKSVTSDLQPYLQTLPVTAEIDNVLGIDYSLVAAPQAKAQVLDVMFKGEIFNRNHRS

PVATPTPTMSLP-EDSKQMVYFAISDYAFNIASRVYHQAGYLNFSITDDMLPHDSGIRLN

TK--AFRPFTPQIYKKYPDMKLELLGTVVSAPILNVSPGNLSLAPQMEIEGFVILPTSAR

EPVFRLGVVTNVFASLTFNNSKVTGMLHPDKAQVRLIESKVGMFNVNLFQAFLNYYLLNS

LYPDVNAELAQGFPLPL----PRHIQLHDLDFQIRKDFLYLGANVQYMRV----------

-------------------

>L variegatus

RRSISIPPKTVMARLKKISPIIIMSIQSKYCAYCYISPYSYY----IKWKLAGWNKL-PG

FKARITQKGLDFVRDVGIHMLQTKIQQLTIPDIHGKADVK-VGHIYYDVTKMHITTFSIP

SATLTTNPTAGGLTLKTSGVSIKVHGDWHYSTHGLAIATIQFIVCHYVSDAEAL---TGC

GVDATGRPSISSQASDCSFSVGGLDIKLHG--GARFVCKLLDLFLSAFGFALCLNFSLFV

Y-VSL-YFYLLLLYSICAIVIAPVEDIIEIDYSLVESPSFNGS-INTAHQGEVYPLGNKT

ECPLPVPQIPPD-SDVSRMVSIWITEYLPNSAGYVLQNIGFLQYNVTPDNVSSHKSKIIY

AKS-SLSLIISQISKMYPNMAMQINVNSTKAPVVKVASTGVQAVLVAYCSPYAILPNKTK

AYLFTLSVVILPDLPYYLAPHPLSHQAMHCSIHQLTRYKA---HSYLLIDYFLTPLVRIY

GRTRVIKNAK------------KFVQYNNRIAGERKIYLFVTGHCGSPFKPISE------

-------------------

>S franciscanus

--MVWSGVGSNMFRGSLMSPILAGFLVVCCILGSSANRNVYRKVGPYKWKLNGWNKE-PG

FKARISNIAFISVRDVGIQMLQKQIQQLTIPDIHGSADIG-IGKVKYDVTSIHITAFSIP

IASLTTNPAAGGLTLKTSGIYLKVHGDWHYKIGGLSVSIILMHLLLN-DTLSPIFTSLNA

GVDTTGRPNISSKASDCSFNIGSLDVDLHG--GARYCT--YIRRSC-GRRHGCGGGPYCA

IPIAQPCIRSSQYYIFQIAVIAPVGDVIEIDYSLVESPSFNGS-ISTCFQGEVYPIGNQT

ECPLPVSQIPPD-SDTSRMVFIWITEYLPNSAGYVLQNIGFLQYNVTPENVSRRHTRLIM

TCLSVVKHNF-QISKMYPNMAMQINVNSTKAPAVKIAPTGIEAVLVADCSPYAVQPNKTL

AYLFTLSMVI------SFSHNFFLLKII---VALSLRCKALEVHPQKGIQSLLFGMEPTI

IYSGVN------------------------------------------------------

-------------------

>S purpuratus

--MVRSGVGSDMFSGSSMSPILAGFLVVCCVLGSSANRNVYRKVGPYKWKLNGWNKE-PG

FKARISQKGLDFLRDVGIQMLQKQIQQLTIPDIHGSADIG-IGDVKYDVTNIRITTFSIP

TASLTPDPAAGGLTLKTSGINLKVHGDWHYKIGGFIPVSDSGDFDAS-ASSISLAVTLRI

GVDATGRPNISSKASDCSFNIGGLDVDLHG--GASWLYNLFDDKIG-DAIKDSLNGQICD

TVIDE-VNGSLEDELKDLQVIAPVGDVVEIDYSLVESPSFNGS-ISTAHKGEVYPIGNTT

ECPLPVPQIPPD-SDISRMVFIWITEYLPNSAGYALQNVGFLQYNVTPENVPAEEKNYLN

TSNFALDLLIPQINKMYPNMAMQINVNSTKAPVVKVASTGVQAVLVGDFIPYAILPNKTL

AYLFTLSVTISAKVSVNFTGNNITWNISSFSTTMKAVRSAVGPFFTSALQLAVNTAIKVD

LIPDLNKLGAVGAPLPQ----LDGFNFTKPVVSLGEGFLKIGTDLTYTP-----------

CLK1

>H sapiens

MSCAGAAAAPRLWRLRPGARRSLSAYGRRTSVRFRSSGMTLDN-----ISRAAVDRIIRV

DHAGEYGANRIYAGQMAVLGRTSVGPVIQKMWDQEKDHLKKFNELMVTFRVRPTVLMPLW

NVLGFALGAGTALLGKEGAMACTVAVEESIAHHYNNQIRTLMEEDPEKYEELLQLIKKFR

DEELEHHDIGLDHDAELAPAYAV----LKSIIQAGCRVAIYLSERL

>H glaber

----------------------IAAYGKKIGLRFCSSGMTLDN-----INWAAMDRIIRV

DHAGEYGANRILAGQMAVLGRTSVGPVIQKMWDQEKAHLKKFSELMVAFRVRPTVLMPLW

NVTGFALGAGTALLGKEGAMACTVAVEETIAHHYNNQIRTLMEEDPEKYEELLQLIKKFR

DEELEHHDTGLSHDAELAPAYAI----LKSVIQAGCRAAIYLSERL

>M brandtii

------------------------------------------------------------

------------------------------------------------------------

-------------------MACTVAVEESIAHHYNNQIRTLMEEDPEKYEELLQVIKKFR

DEELEHHDIGLEHDAELAPAYAI----LKKVIQAGCSAAIYLSERF

>S franciscanus

MQNLTSIRYCVLRQYKLRMSRAIVEV-KGQRSASSLSGLT---------RREMIDRIIRV

DHAGEVGADRIYAGQLAVLKNTSVGPLIQEMWDQEKHHMETFERLIPERRVRPTALLPIW

NVAGFVLGHQSL----------------AICKYFSNITCKLL------------------

---------------------------LPFQIP-------------

>S_purpuratus

MQNLTSIRYCVLRQYKLRMSRAIVEV-KGQRSASSLSGLT---------RREMIDRFIRV

DHAGEVGADRIYAGQLAVLKNTSVGPLIQEMWDQEKHHKETFERLIPERRVRPTVLLPIW

NVAGYVLGAGTALMGKEAAMACTVAVEESIGQHYNDQIRALIEDDPEVHKELLEIIKQFR

DEELEHLNTGLENDAKKVLVRLSWVRKLQWLAQ-------------

>L variegatus

MQNLGSMRYCVLRQYKLRAMRAVTEV-KGQRSASSMSGLT---------RKEMIDRIIRV

DHAGEVGADRIYAGQLAVLKNSSVGPLIQEMWDQEKHHKETFERLIPEHRVRPTALLPIW

NVAGFVLGAGHALIGKEAAMACTVAVEESIGEHYNEQIRALIEDDPEVHKELLEIIKEFR

DDELEHLNTGLENDAKKV---------IRIYAPAVFRSAIS-----

>C elegans

--------------------------MFRVITRGAHTAAS---------RQALIEKIIRV

DHAGELGADRIYAGQLAVLQGSSVGSVIKKMWDEEKEHLDTMERLAAKHNVPHTVFSPVF

SVAAYALGVGSALLGKEGAMACTIAVEELIGQHYNDQLKELLADDPETHKELLKILTRLR

DEELHHHDTGVEHDGMKAPAYSA----LKWIIQTGCKGAIAIAEKI

>M musculus

MSAAGAIAAASVGRLRTGVRRPFSEYGRGLIIRCHSSGMTLDN-----INRAAVDRIIRV

DHAGEYGANRIYAGQMAVLGRTSVGPVIQKMWDQEKNHLKKFNELMIAFRVRPTVLMPLW

NVAGFALGAGTALLGKEGAMACTVAVEESIANHYNNQIRMLMEEDPEKYEELLQVIKQFR

DEELEHHDTGLDHDAELAPAYAL----LKRIIQAGCSAAIYLSERF

>SIG

~~~~~-------------------------------------------------------

------------------------------------------------------------

-------------------------------+----------------------------

COX1

>H sapiens

MFADRWLFSTNHKDIGTLYLLFGAWAGVLGTALSLLIRAELGQPGNLLGNDHIYNVIVTA

HAFVMIFFMVMPIMIGGFGNWLVPLMIGAPDMAFPRMNNMSFWLLPPSLLLLLASAMVEA

GAGTGWTVYPPLAGNYSHPGASVDLTIFSLHLAGVSSILGAINFITTIINMKPPAMTQYQ

TPLFVWSVLITAVLLLLSLPVLAAGITMLLTDRNLNTTFFDPAGGGDPILYQHLFWFFGH

PEVYILILPGFGMISHIVTYYSGKKEPFGYMGMVWAMMSIGFLGFIVWAHHMFTVGMDVD

TRAYFTSATMIIAIPTGVKVFSWLATLHGSNMKWSAAVLWALGFIFLFTVGGLTGIVLAN

SSLDIVLHDTYYVVAHFHYVLSMGAVFAIMGGFIHWFPLFSGYTLNQTYAKIHFAIMFIG

VNLTFFPQHFLGLSGMPRRYSDYPDAYTTWNVLSSVGSFISLTAVMLMIFMIWEAFASKR

KVLMVEEPSMNLEWLYGC-PPPYHTFEE--PVYMKS-

>M brandtii

----------------TLYLMFGAWAGMVGTALSLLIRAELGQPGALLGDDQIYNVIVTA

HAFVMIFFMVMPIMIGGFGNWLVPLMIGAPDMAFPRMNNMSFWLLPPSFLLLLASSMVEA

GAGTGWTVYPPLAGNLAHAGASVDLAIFSLHLAGVSSILGAINFITTIINMKPPALSQYQ

TPLFVWSVLITAVLLLLSLPVLAAGITMLLTDRNLNTTFFDPAGGGDPILYQHLF-----

------------------------------------------------------------

------------------------------------------------------------

------------------------------------------------------------

------------------------------------------------------------

-------------------------------------

>H glaber

MFINRWLFSTNHKDIGTLYLLFGAWAGMTGTALSILIRTELGQPGTLLGDDQIYNVIVTA

HAFIMIFFMVMPIMIGGFGNWLVPLMIGAPDMAFPRMNNMSFWLLPPSFLLLLASSMVEA

GAGTGWTVYPPLAGNLAHAGASVDLTIFSLHLAGVSSILGAINFITTIINMKPPAMSQYQ

TPLFVWSVLITAVLLLLSLPVLAAGITMLLTDRNLNTTFFDPAGGGDPILYQHLFWFFGH

PEVYILILPGFGMISHIVTYYSGKKEPFGYMGMVWAMMSIGFLGFIVWAHHMFTVGMDVD

TRAYFTSATMIIAIPTGVKVFSWLATLHGGNIKWSPAMLWALGFIFLFTVGGLTGIVLAN

SSLDIVLHDTYYVVAHFHYVLSMGAVFAIMAGFVHWFPLFSGYTLDPTWAKIQFSVMFIG

VNMTFFPQHFLGLSGMPRRYSDYPDAYTMWNTLSSMGSLISLTAVMMMVFMIWEAFASKR

EVLMTEMTSTNLEWIHGC-PPPFHTFEE--PTYIKM-

>S franciscanus

MQLRR-LFSTNHKDIGTLYLIFGA-AGMVGTAMSVIIRAELAQPGSLLKDDQIYKVVVTA

HALVMLFFMVMPIMIGGFGN-LIPLMIGAPDMAFPRMKNMRF-LIPPSFIL---------

------------------------------------------------------------

------------------LPVLAGAITMLLTDRNINTTFFDPAGGGDPILFQHLF-FFGH

PEVYILILPGFGMISHVIAHYSGKREPFGYLGMVYAMIAIGVLGFLVWAHHMFTVGMDVD

TRAYFTAATMIIAVPTGIKVFR-MATLQGSNLQCETPLLWALGFVFLFTLGGLTGIVLAN

SSIDVVLHDTYYVVAHFHYVLSMGAVFAIFAGFTH-FPLFSGYSLHPL-GKVHFFIMFVG

VNLTFFPQHFLGLAGMPRRYSDYPDAYTL-NTISSIG---SVVAMLFFLFLI-EAFASQR

EGITPEFSHASLE-QYTSFPPSHHTFDETPSTIIIVK

>S purpuratus

MQLSRWLFSTNHKDIGTLYLIFGAWAGMVGTAMSVIIRAELAQPGSLLNDDQIYNVVVTA

HALVMIFFMVMPIMIGGFGNWLIPLMIGAPDMAFPRMNNMSFWLIPPSFILLLASAGVEN

GAGTGWTIYPPLSSNITHAGSSVDLAIFSLHLAGASSILGLINFITTIINMRTPGMSLDR

LPLFVWSVFVTAFLLLLSLPVLAGAITMLLTDRNINTTFFDPAGGGDPILFQHLFWLFGH

PEVYILILPGFGMISHVIAHYSGKREPFGYLGLVYAMIAIGVLGFLVWAHHMFTVGMDVD

TRAYFTAATMIIAVPTGLKVFSWMAKLQGSNLQWSLPLLWTLGIVFLFTLGGLTGIVLAN

SSIDFVLHDTYYVVAHFHYVLSMGAVFAIFAGFTHWFPLFSGYSLHPLWGKVHFFIMFVG

VNLTFFPQHFLGLAGMPRRYSDYPDAYTLWNTISSIGSTISVVAMLFFLFLIWEAFASQR

EGITPEFSHASLEWQYTSFPPSHHTFDETPSTIIIVK

>L variegatus

MQLRR-LFSTNHKDIGTLYLIFGA-AGMVGTAMRVIIRAELAQPGSLLKDDQIYNVVVTA

HALVMIFFMAMPIMIGGLGN-LIPLMIGAPDMAFPRMKKMRF-LVPPSFILLLASAGVER

GAGTG-TIYPPLSSKIAHAGGSVDLAIFSLHLAGASSILASINFITTIINMRTPGMSFDR

LPLFV-SVFVTAFLLLLSLPVLAGAITMLLTDRNINTTFFDPAGGGDPILFQHLF-FFGH

PEVYILILPGFGMISHVIAHYSGKREPFGYLGMVYAMIAIGILGFLV-AHHMFTVGMDVD

TRAYFTAATMIIAVPTGIKVFR-MATLQGSNLQ-ETPLLWALGFVFLFTLGGLTGIVLAN

SSIDVVLHDTYYVVAHFHYVLSMGAVFAIFAGFTH-FPLFSGYSLHPL-GKVHFFIMFVG

VNLTFFPQHFLGLAGMPRRYSDYPDAYTL-NTVSSIGSTISVVAMLFFLFLI-EAFASQR

EGITPEFSHASLEWQYTSFPPSHHTFDETPSTIIIVK

>M musculus

MFINRWLFSTNHKDIGTLYLLFGAWAGMVGTALSILIRAELGQPGTLLGDDQIYNVIVTA

HAFVMIFFMVMPMMIGGFGNWLVPLMIGAPDMAFPRMNNMSFWLLPPSFLLLLASSMVEA

GAGTGWTVYPPLAGNLAHAGASVDLTIFSLHLAGVSSILGAINFITTIINMKPPAMTQYQ

TPLFVWSVLITAVLLLLSLPVLAAGITMLLTDRNLNTTFFDPAGGGDPILYQHLFWFFGH

PEVYILILPGFGIISHVVTYYSGKKEPFGYMGMVWAMMSIGFLGFIVWAHHMFTVGLDVD

TRAYFTSATMIIAIPTGVKVFSWLATLHGGNIKWSPAMLWALGFIFLFTVGGLTGIVLSN

SSLDIVLHDTYYVVAHFHYVLSMGAVFAIMAGFVHWFPLFSGFTLDDTWAKAHFAIMFVG

VNMTFFPQHFLGLSGMPRRYSDYPDAYTTWNTVSSMGSFISLTAVLIMIFMIWEAFASKR

EVMSVSYASTNLEWLHGC-PPPYHTFEE—PTYVKVK

COX2

>S purpuratus

MGTWAQFGLQDASSPLMEELTYFHDYALIVLTLITILVFYGLVSLLVSSNTNRFFFEGQE

LETIWTVIPALILILIALPSLQLLYLMDEVNDPFLTIKAFGHQWYWSYEYTDFNDLEFDS

YMVPTSDVSFGNPRLLEVDNRLVLPMQNPIRVLVSSADVLHSWAVPSLGTKMDAVPGRLN

QTTFFAARTGVFYGQCSEICGANHSFMPIVIESVPFNTFENWVTQYLEE

>L variegatus

-MATAQFGLQDASSPLMEELTYFHDYALIVLTLITILVFYGLASLLVSSSTNRFFLEGQE

LETI-TVIPALILIFIALPSLQLLYLMDEVKDPFLTIKAIGHQ--YSYEYTDYKDLEFDS

YMVPTSDISLRDARLLEVDNRLILPMQNPIRVLVSSADVLHS-AVPSLGVKMDAVPGRLN

QTTFFAARTGLFYGQCSEICGANHRFMPIVIESVPFNTFEN-VAQYLEE

>S franciscanus

-MATAQFGLQDASSPLMEELTYFHDYALIVLTLITILVFYGLVSLLVSSNTNRFFLEGQE

LETI-TVIPALILILIALPSLQLLYLMDEVKDPFLTIKAIGHQ--YRYEYTDYKDLEFDS

YMVPTSDVSLGNPRLLEVDNRLILPMQNPIRVLVSSADVLHS-AVPSLGVKMDAVPGRLN

QTTFFAARTGLFYGQCSEICGANHSFMPIVIESVPFNTFEN-VAQYLEE

>H sapiens

MAHAAQVGLQDATSPIMEELITFHDHALMIIFLICFLVLYALFLTLTTKLTNTNISDAQE

METVWTILPAIILVLIALPSLRILYMTDEVNDPSLTIKSIGHQWYWTYEYTDYGGLIFNS

YMLPPLFLEPGDLRLLDVDNRVVLPIEAPIRMMITSQDVLHSWAVPTLGLKTDAIPGRLN

QTTFTATRPGVYYGQCSEICGANHSFMPIVLELIPLKIFEMGPVFTL--

>H glaber

MPYPHQLGFQDATSPIMEELLYFHDHTLMIVFLISSLVLYTISTMLSTKLTHTNTMNAQE

IEMVWTILPAMILILIALPSLRILYMIDEINDPSLTVKTLGHQWYWSYEYTDYEELCFDS

YMVPSTDLKPGELRLLEVDNRVVLPMKTPVRMLISSEDVLHSWAVPSLGLKTDAVPGRLN

QTTLMSSRPGLFYGQCSEICGSNHSFMPIVIEMVPLKTFESWSSLMT--

>233142508__M_musculus 121 origGI 422034515

MAYPFQLGLQDATSPIMEELMNFHDHTLMIVFLISSLVLYIISLMLTTKLTHTSTMDAQE

IETIWTILPAVILIMIALPSLRILYMMDEINNPVLTVKTMGHQWYWSYEYTDYEDLCFDS

YMIPTNDLKPGELRLLEVDNRVVLPMELPIRMLISSEDVLHSWAVPSLGLKTDAIPGRLN

QATVTSNRPGLFYGQCSEICGSNHSFMPIVLEMVPLKYFENWSASMI—

COX3

>H sapiens

MTHQSHAYHMVKPSPWPLTGALSALLMTSGLAMWFHFHSMTLLMLGLLTNTLTMYQWWRD

VTRESTYQGHHTPPVQKGLRYGMILFITSEVFFFAGFFWAFYHSSLAPTPQLGGHWPPTG

ITPLNPLEVPLLNTSVLLASGVSITWAHHSLMENNRNQMIQALLITILLGLYFTLLQASE

YFESPFTISDGIYGSTFFVATGFHGLHVIIGSTFLTICFIRQLMFHFTSKHHFGFEAAAW

YWHFVDVVWLFLYVSIXWWGS

>H glaber

MNHQTHAFHMVNPSPWPLTGALSALLITSGLTMWFHFNSMTLMLLGLLACIMTMYQWWRD

IVREGTYQGHHTPTVQKGLRYGMVLFIISEVFFFAGFFWAFYHSSLAPTPELGGHWPPAG

INPLNPTEVPLLNTAILLASGVSITWAHHSLMEGHRKHVIQSLLTTIILGIYFTLLQASE

YFEAPFTISDGIYGSTFFVATGFHGLHVIIGSTFLTICLIRQLLFHFTSKHHFGFEAAAW

YWHFVDVVWLFLYVSIYWWGS

>S franciscanus

MAHQ-HPYHLVDQSPCPLTGAISGLMMTSGLVL-FHTQRLILLSVGLLLLITTMINWWRD

IIREATFQGRHTAIVEKGLRYGMILFITSEVCFFFA---AFFHRRLAPSVEIGVA-PPTG

ITPLNPFLVPLLKTAVLLSSGVTIT-SHHRILAGNRTESIQALFLTVALGMYFTILQA-E

YIDAPFTIADSVYGSTFFVATGFHGLHVIIGTTFLMVCLFRLAGHHFSTHHHFGFEAAA-

YWHFVDVV-LFLYVCI-YWGS

>S purpuratus

MAHQ-HPYHLVDQSPWPLTGAFSGLMMTSGNVLWFHTQKTNLTLVGFLLLITNMVNWWRD

IIRKANFQGSHTAIVNKGMRYGMILFITSEVCFFFAFFWAFFHSSLAPSVEIGVAWPPSG

ITPLNPFLVPLLNTGVLLSSGVTLSWSHHSILAGNRTESIQALFLTVALGSYFTALQAWE

YIDAPFTIADSVYGSTFFVATGFHGLQVIIGTTFLMVCLFRTAGRHFSTHHHFGFEAAAW

YWHFVDVVWFVLYWLIYWWGA

>M musculus

MTHQTHAYHMVNPSPWPLTGAFSALLLTSGLVMWFHYNSITLLTLGLLTNILTMYQWWRD

VIREGTYQGHHTPIVQKGLRYGMILFIVSEVFFFAGFFWAFYHSSLVPTHDLGGCWPPTG

ISPLNPLEVPLLNTSVLLASGVSITWAHHSLMEGKRNHMNQALLITIMLGLYFTILQASE

YFETSFSISDGIYGSTFFMATGFHGLHVIIGSTFLIVCLLRQLKFHFTSKHHFGFEAAAW

YWHFVDVVWLFLYVSIYWWGS

>L variegatus

MAHQ-HPYHLVDQSP-PLTGAIRGLMMTSGLVL-FHTQRIVLLTVGFLLLVTTMIN--RD

IVREATFQGRHTAIVEKGLRYGMILFITSEVCFFFAFF-AFFHRRLAPSTEIGLT-PPTG

ITPLNPFLVPLLKTAVLLSSGVTIT-SHHRILAGNRTEAIQALFLTVTLGIYFTVLQA-E

YIDSPFTIADRVYGSTFFVATGFHGLHVIIGTTFLIVCLFRLAGHHFSTHHHFGFEAAA-

YWHFVDVV-LFLYVCI-YWGS

COX4

>H sapiens

MLATRVFSLVGKRAISTSVCVRAHESVVKSEDFSLPAYMDRRDHPLPEVAHVKH--LSAS

QKALKEKEKASWSSLSMDEKVELYRIKFKESFAEMNRGSNEWKTVVGGAMFFIGFTALVI

MWQKHYVYGPLPQSFDKEWVAKQTKRMLDMKVNPIQGLASKWDYEKNEWKK

>H_glaber

MLATRVFSLISKRAISTSVCARAHGSVVKSEDYALPSYVDRRDYPLPDVAHVQQ--LSAS

QKALKEKEKASWSSLTRDEKVELYRIRFNESFAEMNRSTNEWKTVVGAAMFFIGFTGLIL

IWEKRHVYGPIPHTFDEEWVAMQTKRMLDMKASPIQGFSAKWDYDKNEWKK

>M brandtii

MLTTRVFSLIGKRAISTSVCARAHGSVVKSEDFSLPAYVDRRDYPLPDVAHVKS--LSAS

QKALKEKEKNPWNSLSIDEKVELYRIKFNESFAEMNRSTNEWKTVVGAAMFFIGFTALVL

IWEKRFVYGPIPHTFEEEWVAKQTKRMLDMKVSPIQGFSAKWDYDKNEWKK

>S francifcanus

--MASLMRLSLRRVASNV-------SRTPKRNFHDKLYADRLDYPAPNCRFLQETELSAE

MQALKVKERGDWKALTQADKKEVYRMSFNKTYSEMRAPTGEWKYVVGGVCIAIAISGVIY

LFQPAYSSPPSPHHMSDEWQEAQLRYQIAIRNGPVTGISSQWDYEKNEWKK

>S purpuratus

--MASLMRLSVRRVASSV-------SRTPKRNFHDKLYADRLDYPAPNCRFVQEAELSAE

MQALKVKERGDWKALTQADKKELYRMSFNKTYSEMRAPTGEWKYVIGGVCIAIAVSGVIY

GFQKAYISPPLPHTMSDEWQEAQLRYQIAIRNGPVTGIASQWDYEKNEWKK

>L variegatus

--MASLMRLSVRRAASNV-------SKTPKRNFHDKLYADRLDYPAPNCRFMKEADLSSD

MQALKVKERGDWKALSQAEKKEVYRMSFNKTYSEMRAPTGEWKYIIGGVSIALAFSAVIY

GFQKAYVSPPLPHTMSPEWQEAQLRREIAIRNGPVTGTASKWDYEKNEWKK

>M musculus

MLASRALSLIGKRAISTSVCLRAHGSVVKSEDYAFPTYADRRDYPLPDVAHVTM--LSAS

QKALKEKEKADWSSLSRDEKVQLYRIQFNESFAEMNRGTNEWKTVVGMAMFFIGFTALVL

IWEKSYVYGPIPHTFDRDWVAMQTKRMLDMKANPIQGFSAKWDYDKNEWKK

COX5b

>H sapiens

MASRLLRGAGTLAAQALRARGPSGAAAMRSMASGGGVPTDEEQATGLEREIMLAAKKG--

LDPYNVLAPKGASGTREDPNLVPSISNKRIVGCI---CEEDNTSVVWFWLHKGEAQRCPR

CGAHYKLVPQQ----LAH

>M brandtii

MASRLLRGTGALVAQALRARGPNAAAAVRSMASAGGVPTDDEQATGLEREVMLAARKG--

LDPYNMLAPKPASGTKEDPNLVPSITNKRIVGCI---CEEDNSAVIWFWVHKGETQRCPS

CGTHYKLVPHQ----LSH

>S franciscanus

MATITLRKCALTAARRCLFRQSS-----RAMAAGGGNFLIFTVIGKMCTSMLVL------

YCSLGLETPVRTRVIAPIPLFISSLS--LFVSHV---GEEEQTHINWMWLYTGEPQRC-E

CGHWFKLSEVEGIAAAAH

>S purpuratus

MATITLRKCALTAARRCLYSQSS-----RAMAAGGGIPDNFEHATGLEKKELDRIKAGD-

SDPFQLNVRRVAAGTKTKPVEVTSAFQKRIVGCI---CEEEQTHINWMWLYTGEPQRC-E

CGHWFKLQEVEGIAAAAH

>L variegatus

MATITLRKCALTAARRCLFSQSS-----RAMAAGGGIPDNFEHATGLEKKELERIKAGDA

AAPLSF-----SASISIAPIPFIYIHEPCRPSCIPYALEEEQTHINWMWLYTGEPQRC-E

CGHWFKLTEVEGIAAAAA

>M musculus

MASRLLRGVGALAAQALRAHGPRGAAVTRSMASGGGVPTDEEQATGLEREIMIAAQKG--

LDPYNMLPPKAASGTKEDPNLVPSISNKRIVGCI---CEEDNCTVIWFWLHKGESQRCPN

CGTHYKLVPHQ----MAH

COX6a

>H sapiens

---MAVVGVSSVSRLLGRSRPQLGRPMSSGAHGEEGSARMWKTLTFFVALPGVAVSMLNV

YL----KSHHGEHERPEFIAYPHLRIRTKPFPWGDGNHTLFHNPHVNPLPTGYEDE

>H glaber

----AAIAASRVSRLLGRSRPQVGRIMSSGAHGEQGSARMWKALTYFVALPGVGVSMLNV

FL----KSRHGKEERPEFIAYPHLRIRTKPFPWGDGNHTLFHNPHVNPLPTGYEDE

>M brandtii

---MATAAASRVAGLLDRLRPQLARPMSSGAHSQEGSARLWKSLTYFVALPGVAVSMLNV

FL----KSRHGEHERPEFVAYPHLRIRTKPFPWGDGNHTLFHNPHLNPLPTGYEDE

>S franciscanus

------------------------------------------------------SSLTSI

FI---------------FFFFFHL-----SFPWGDGNHSLFHNAEANALPEGYEEE

>S purpuratus

----------MLELWLWSDHEMVIVFL---DFLFLGSAKTWKYMSFLVAIPGVIFCMVNA

YKGEMEHKAHFAAHKPEFHAYTHLRIRTKSFPWGDGNHSLFHNVESNALPEGYEEE

>L variegatus

-----------LGEYSGLSKRDSGIFF---CFVLSGTAKTWKYMSFLVALPGVMFCMVNA

YKGEMEHKAHLAHHKPEFVAYSHISIRTQAFPWGDGNHSLFHNAESNALPEGYEE-

>M musculus

MMASAVLSASRVSRPLGRALPGLRRPMSSGAHGEEGSARMWKALTYFVALPGVGVSMLNV

FL----KSRHEEHERPPFVAYPHLRIRTKPFPWGDGNHTLFHNPHVNPLPTGYEDE

COX6b

>H sapiens

MAEDMETKIKNYKTAPFDSRFPNQNQTRNCWQNYLDFHRCQKAMTAKGGDISVCEWYQRV

YQSLCPTSWVTDWDEQRAEGTFPGKI

>H glaber

MAEDIKTKIKNYKTAPFDSRFPNQNQTRNCWQNYLDYHRCQKAMNAKGGDVSVCEWYHRV

YKSLCPISWVSTWDERRVEGTFPGKI

>M brandtii

MAEDIKTKIKNYRTAPFDSHFPNQNQTRNCWQNYLDFHRCEKAMTAKGGDVSVCEWYRRV

YKSLCPISWVSAWDDRRAEGTFPGKI

>S franciscanus

-MADIKEVLSNYKTAPFDARFPNTNQTKNCWQNYVDFHRCQK---MKGEDFEPCEYFRRV

YKSLCPIAWVS------MTHTY----

>S purpuratus

-MADIKEVLENYKTAPFDARFPNTNQTKNCWQNYVDFHRCQK---MKGEDYEPCEYFKRV

YKSLCPIAWAENWDSQREEGTFASKI

>L variegatus

-MSEFKEILENYRTAPFDARFPNTNQTKNCWQNYVDFHRCQK---KKGEDYEPCEYFRRV

YKSLCPLAWVSINYLNDIYYQYCAYD

>M musculus

MAEDIKTKIKNYKTAPFDSRFPNQNQTKNCWQNYLDFHRCEKAMTAKGGDVSVCEWYRRV

YKSLCPVSWVSAWDDRIAEGTFPGKI

COX6c

>H sapiens

------------------------------------------------------------

------------------------------------------------------------

------------------------------------------------------------

------------------------------------------------------------

------------------------------------------------------------

------------------------------------------------------------

------------------------------------------------------------

------------------------------------------------------------

------------------------------------------------------------

------------------------------------------------------------

------------------------------------------------------------

------------------------------------------------------------

------------------------------------------------------------

------------------------------------------------------------

------------------------------------------------------------

------------------------------------------------------------

------------------------------------------------------------

------------------------------------------------------------

------------------------------------------------------------

------------------------------------------------------------

------------------------------------------------------------

------------------------------------------------------------

------------------------------------------------------------

------------------------------------------------------------

------------------------------------------------------------

------------------------------------------------------------

------------------------------------------------------------

------------MAPEV-LPKPRMRGLLARRLRNHMAVAFVLSLGVAALYKFRVADQRKK

AYADFYRNYDVMKDFEEMRKAGIFQSVK-------

>H glaber

------------------------------------------------------------

------------------------------------------------------------

------------------------------------------------------------

------------------------------------------------------------

------------------------------------------------------------

------------------------------------------------------------

------------------------------------------------------------

------------------------------------------------------------

------------------------------------------------------------

------------------------------------------------------------

------------------------------------------------------------

------------------------------------------------------------

------------------------------------------------------------

------------------------------------------------------------

------------------------------------------------------------

------------------------------------------------------------

------------------------------------------------------------

------------------------------------------------------------

------------------------------------------------------------

------------------------------------------------------------

------------------------------------------------------------

------------------------------------------------------------

------------------------------------------------------------

------------------------------------------------------------

------------------------------------------------------------

------------------------------------------------------------

------------------------------------------------------------

------------MSSSA-LTKPQMRGLLARRLRIHMVGAFIVSLGVATLYKFGVAEPRKK

AYADFYRNYDSMKDFEEMRKAGIFQSAK-------

>M brandtii

MSERHSGQVGTGVGNEMDSPTVTIKYTGFKDFSSTSSFQDSGYSELLKSYSVDNTLFLVK

QEDDPTINYEFFEPVSPGLTDPLETPTKRKRFIIFRKKSAIPPDFCETPKLGGKRYLRRR

RRLDVSFSPLKRNYESENSSIESSISEVLNFEEDIPSSASGSPRQNNFSALVTSTLKTEE

ATLSPQKLRLNFSQQKTSTVDDSKDDCSLFEDECISPIQGNNFKDSITHDFSDSSPCVND

ENTFPKLLGSSSSGTTCETDEDIFVTPISNLVANIKFKAGQRFFPQVRGNISTPEDSGFN

SLCLDKSEDSLSDQESSFQELLQKHKGTLKVEDTIRRSRRLGRLRRLSTLREQGSQSETE

EESQIIHSESEITPAASSDILESQPSSDSQSEELILGFNNLSNTPALQLVHELFMKNKKR

RFQQSGTREFLEDRDEAKIAVLQCVLAGLIGKKMGIEKLDILTELKYRNLKHILAVVLDS

LSAESLCSVWSVSRNWREIIVQNKKANQRRRFYLTQLKTDAKQAVLNVQDAATRFQLSNR

SALRSVQAQARTPSSQKEQVSTFSPWGEVLTPIASSSIIDSHSKQEEYVREDSLATDNFL

VDYFNEFLSLPTFSEAIRFNVNYGVFEVVNNAPQLLEKQLKKILQSQQPRNPIYDVVRKA

KNDIKPAQMSDPYKAETININYSVMCLNREQGIQWIKKERLPAFLESDCYFEYRHLEKMK

KNYLVSSGDRHLSTEILTKLKLLDGSQWNKEHLINIQTEVVKPLLLYWAPRFCVTHSSAI

RKASAELKLWHRRQEKPREDIDPFPQIATLLPLRPKSCIPQISDTEKEESSLVPPPTSPK

KPSTASQKPRKGETQRSISPKDSVTEKGSKQMSERYNVIHLTSYTDISECLKPELERRFT

YSEEPLVKTMADGALGGSDMENLLQSLYVENRAGFFFTKFCEHSGNELWKNSVYFWFDLQ

AYHQLFYQETLHPFKVCKQAQLLFATYVAPSATLDIGLQQQKKKEIYMKIQPPFEDLFDT

AEEYILLLLLEPWTRMVQLDQLAYKQVKLVEETRQLDSVNFRKLQALYKETVFKKAEVKL

VEETRQLDSVNFRKLQALYKETVFKKAEDATSEIGTGIPPHPAATKSTEYWNIVPAEYKN

FNFNNLLNNKLEFEHFRQFLEAYSASIDLMCWADIEQFRSIPYKDRKLREAKSIYIKNKY

LNKTYFLGPTSPASPYQQDQIMSLCGGWGKTLHEQLDPPVFVEVQKHVLKRIENVWLPLF

LASEQFAARQKIKLQLKEATDELLLQKHEEKIGIWKPVESKWISSSSEIIAFRKALLNPV

TANQFQRFVALKGDLLENGVLFWQEVQKYKDLCHSHCDESIIHRKVMTIINCFINSSIPP

ALQIDIPVEQAQKIIEHRKELGPYVFREAQMTIFGVLFKFWPQFCEFRKNLTDEKIMSAL

ERRKEYHRQKKRSTVVEEERFQKTPYPDIENIKLAVPSELSPASTVFGWNRPRNAPSSNL

RPPSNLSLGFYFDCGDVALEGSGFFFCDLAEKRCQGAEHLKEAKPCSLKRCQGAEHLKDA

KLCSLPGRAEASQGRWAKLRAVEAALALERSLTQALWELQAQVLPKQTLSSLASWRTIFW

NVLRPPKETITAMSAGT-LPKPQMRGLLAKRLRVHIVGAIIVSLGVAASYKFGVAEPRKK

AYADFYRNYDSMKDFEEMRKAGIFQSTK-------

>S franciscanus

------------------------------------------------------------

------------------------------------------------------------

------------------------------------------------------------

------------------------------------------------------------

------------------------------------------------------------

------------------------------------------------------------

------------------------------------------------------------

------------------------------------------------------------

------------------------------------------------------------

------------------------------------------------------------

------------------------------------------------------------

------------------------------------------------------------

------------------------------------------------------------

------------------------------------------------------------

------------------------------------------------------------

------------------------------------------------------------

------------------------------------------------------------

------------------------------------------------------------

------------------------------------------------------------

------------------------------------------------------------

------------------------------------------------------------

------------------------------------------------------------

------------------------------------------------------------

------------------------------------------------------------

------------------------------------------------------------

------------------------------------------------------------

------------------------------------------------------------

------------MAA---LPRPQMRGLLSSFLTRHFIIGSVLSLAGAGLVKVFVYDARKK

LYTEFYKTYDAQADFERMRELGVFQSARPLSEER-

>S purpuratus

------------------------------------------------------------

------------------------------------------------------------

------------------------------------------------------------

------------------------------------------------------------

------------------------------------------------------------

------------------------------------------------------------

------------------------------------------------------------

------------------------------------------------------------

------------------------------------------------------------

------------------------------------------------------------

------------------------------------------------------------

------------------------------------------------------------

------------------------------------------------------------

------------------------------------------------------------

------------------------------------------------------------

------------------------------------------------------------

------------------------------------------------------------

------------------------------------------------------------

------------------------------------------------------------

------------------------------------------------------------

------------------------------------------------------------

------------------------------------------------------------

------------------------------------------------------------

------------------------------------------------------------

------------------------------------------------------------

------------------------------------------------------------

------------------------------------------------------------

------------MAA---LPRPKMRGLLSSFLTRHFIIGSVLSLAGAGLVKVFLYDARKK

LYTEFYKTYDAQADFERMRELGVFQSARPLSEEAE

>M musculus

------------------------------------------------------------

------------------------------------------------------------

------------------------------------------------------------

------------------------------------------------------------

------------------------------------------------------------

------------------------------------------------------------

------------------------------------------------------------

------------------------------------------------------------

------------------------------------------------------------

------------------------------------------------------------

------------------------------------------------------------

------------------------------------------------------------

------------------------------------------------------------

------------------------------------------------------------

------------------------------------------------------------

------------------------------------------------------------

------------------------------------------------------------

------------------------------------------------------------

------------------------------------------------------------

------------------------------------------------------------

------------------------------------------------------------

------------------------------------------------------------

------------------------------------------------------------

------------------------------------------------------------

------------------------------------------------------------

------------------------------------------------------------

------------------------------------------------------------

------------MSSGALLPKPQMRGLLAKRLRVHIAGAFIVALGVAAAYKFGVAEPRKK

AYAEFYRNYDSMKDFEEMRKAGIFQSAK-------

>L variegatus

------------------------------------------------------------

------------------------------------------------------------

------------------------------------------------------------

------------------------------------------------------------

------------------------------------------------------------

------------------------------------------------------------

------------------------------------------------------------

------------------------------------------------------------

------------------------------------------------------------

------------------------------------------------------------

------------------------------------------------------------

------------------------------------------------------------

------------------------------------------------------------

------------------------------------------------------------

------------------------------------------------------------

------------------------------------------------------------

------------------------------------------------------------

------------------------------------------------------------

------------------------------------------------------------

------------------------------------------------------------

------------------------------------------------------------

------------------------------------------------------------

------------------------------------------------------------

------------------------------------------------------------

------------------------------------------------------------

------------------------------------------------------------

------------------------------------------------------------

------------MSA---LPRPQMRGLLKSFLTRHFIIGATLSVTGAALVKVFCKLHFSP

YWVQSYKTYDAQADFERMRELGVFQSARPLSEQE-

COX7c

>H sapiens

MLGQSI-----RRFTTSVVRRSHYEEGPGKNLPFSVENKWSLLAKMCLYFGSAFATPFLV

VRHQLLKT

>H glaber

MLGQSI-----RRFTASAVRRSHYEEGPGKNLPFSVENKWRLLVMMTLYFGSGFAAPFFI

VRHQLLKK

>S franciscanus

MLLSRLAVVSRRSFGTSVIRQAGYGEGPGTNMPFPTRNKWLLLGIMAAFFGSGFAAPFIL

VRHQLLKK

>S purpuratus

MVFSRLAVISRRSFGTSVIRQAGYGEGPGTNMPFPTRNKWLLLGIMAAFFGSGFAAPFVL

VRHQLLKK

>L variegatus

MVFSRLAVVSRRSFGTSVIRQGGYGEGPGTNMPFPTKNKWALLGIMAAFFGSGFAAPFIL

VRHQLLKK

>M musculus

MLGQSI-----RRFTTSVVRRSHYEEGPGKNLPFSVENKWQLLAMMTVYFGSGFAAPFFI

VRHQLLKK

CuZnSOD

>H sapiens

MATKAVCVLKGDGPVQGIINFEQK---ESNGPVKVWGSIKGLTEGLHGFHVHEFGDNTAG

CTSAGPHFNPLSRKHGG--PKDEERHVGDLGNVTADKDGVADVSIEDSVISLSGDHCIIG

RTLVVHEKADDLGKGGNEESTKTGNAGSRLACGVIGIAQ

>H glaber

MATKAVCVLKGDGPVHGTIHFEQQ---EGNGPVVVKGRIAGLNEGQHGFHVHEFGDNTKG

CTSAGPHFNPLSKKHGG--PKDEERHVGDLGNVTAGTDGVAEVSIEDSLISLFGPNSIIG

RTMVVHEKEDDLGKGGNEESTKTGNAGSRLACGVIGIAQ

>M brandtii

-LLWGRADSMSP-TWSQTCASLWI---QGNGKVKVSGSIRELTEGEHGFHVHQFGDNTQG

CTSAGPHFNPLSKTHGG--PQDEERHVGDLGNVTAGPDGVANVLIEDSVIALSGDHSIIG

RTLVVHEKRDDLGKGGNDESKKTGNAGSRLACGVIGITQ

>S franciscanus

KKQYSISHSISS-P---SCLFQFVINSHQSNSVSVKGKLTLAAPSQHGFHVHQYGDYSGS

CSTTGGHYNPDGVDHAS--PTSAYRHVGDLGNVQADASGRIEVSLSDKYASLSGAQAIIG

RAFVVRCYVLSIIR--QSEKLKYCKKYHSECCRILERLS

>S purpuratus

MSVKAVCMLVGE-AVKGRIEFEQG---EGSNSVSVKGEVTGLAPGQHGFHIHQFGDYTNG

CVSAGGHFNPFGKEHGA--PEDEMRHVGDLGNIIADASGKVDVNLSDKLLSLSGPQSIIG

RAVVVHADVDDLGKGGHATSKTTGNAGGRLACGVIGIQA

>L variegatus

PKQFYFCEKLYE-VFSVICSYYL-----AVGSVNVSGTVTGLAPGQHGFHIHQFGDNTNG

IVKSPLNILSCGMYYSIIFP---YRHVGDLGNIVADDSGTANVNITDRQLSLSGPQSIIG

RSVVVHADIDDLGKGGHELSKTTGNAGGRLACGVIGIRK

>M musculus

-AMKAVCVLKGDGPVQGTIHFEQK---ASGEPVVLSGQITGLTEGQHGFHVHQYGDNTQG

CTSAGPHFNPHSKKHGG--PADEERHVGDLGNVTADKDGVADVSIEDSVISLSGDHCIIG

RTLVVHEKADDLGKGGNEESTKTGNAGSRLACGVIGIAQ

CYTB

>H sapiens

-MTPMRKTNPLMKLINHSFIDLPTPFNISAWWNFGSLLGACLILQITTGLFLAMHYSPDA

STAFSSIAHITRDVNYGWIIRYLHANGASMFFICLFLHIGRGLYYGSFLYSETWNIGIIL

LLATMATAFMGYVLPWGQMSFWGATVITNLLSAIPYIGTDLVQWIWGGYSVDSPTLTRFF

TFHFILPFIIAALATLHLLFLHETGSNNPLGITSHSDKITFHPYYTIKDALGLLLFLLSL

MTLTLFSPDLLGDPDNYTLANPLNTPPHIKPEWYFLFAYTILRSVPNKLGGVLALLLSIL

ILAMIPILHMSKQQSMMFRPLSQSLYWLLAADLLILTWIGGQPVSYPFTIIGQVASVLYF

TTILILMPTISLIENKMLKWA

>H glaber

-MTNIRKSHPLIKIINHSFIDLPTPSSISYWWNFGSLLGACLILQIITGLFLSMHYTADT

ATAFSSVAHICRDVNYGWLIRYLHANGASMFFICLYLHVGRGMYYGSYMFMETWNIGIIL

LLSVMATAFMGYVLPWGQMSFWGATVITNLFSAIPYIGPTLVEWIWGGFAVDKATLTRFF

AFHFILPFIITALTMVHLLFLHETGSNNPSGINSDSDKIPFHPYYSFKDFMGLQIMLLIL

LTLTLFHPDLLGDPDNYTPANPMSTPPHIKPEWYFLFAYAILRSIPNKLGGVLALVMSIL

ILVALPLLHTSKQRSMMFRPISQCLFWTFISTLLTLTWIGSQPVEYPYIIIGQLASILYF

LIILVLMPLAGLVENKMMK--

>M brandtii

-MTNIRKSHPLMKIINSSFIDLPAPSNISSWWNFGSLLGICLALQILTGLFLAMHYTSDT

TTAFNSVTHICRDVNYGWVLRYLHANGASMFFICLYLHVGRGLYYGSYMYTETWNIGVIL

LFAVMATAFMGYVLPWGQMSFWGATVITNLLSAIPYIGTDLVEWIWGGFSVDKATLTRFF

AFHFLLPFIIAAMVMVHLLFLHETGSNNPTGIPSNADMIPFHPYYTIKDILGLLLMITVL

LMLVLFSPDLLGDPDNYTPANPLNTPPHIKPEWYFLFAYAILRSIPNKLGGVLALVLSIL

ILIIIPLLHTSKQRSMTFRPLSQCLFWLLTADLFTLTWIGGQPVEHPYVIIGQLASILYF

SIIIILMPLISLMENHLLKW-

>S franciscanus

MAAPLRKEHPIFRILKSTFVDLPLPSNLSI-WNFGSLLGLCLIVQILTGIFLAMHYTADI

TLAFSSVRHICRDVKYGWLLRKVHAKGASLFFICMYCHIGRGLYYGSYKKIET-KVGVIL

FLVTILTAFMGYVLV-GQMSF-AATVITNLVSAIPYMGTTIVQ-----------------

-LHFLFPFIIAALAIIHLVFLHNRGANNPVGLKSNYDKAPFHIYYTTKDTVGFILLIAAL

FSLALLFPGALKDPENFIPANPLVTPPHIQPE-YFLFAYAILRSIPNKLGGVIALVAAIL

VLFLMPLLNTSKKESNSFRPLSQAAF-LLVATFLILT-IGRQPVEYPYVLLGQVASVIYF

SLFIFGFPLVSSIENKIIFS-

>S purpuratus

MAAPLRKEHPIFRILNSTFVDLPLPSNLSIWWNSGSLLGLCLVVQILTGIFLAMHYTADI

TLAFSSVMHILRDVNYGWFLRYVHANGVSLFFICMYCHIGRGLYYGSYNKIETWNVGVIL

FLVTILTAFMGYVLVWGQMSFWAATVITNLVSAIPYIGTIIVQWLWGGFSVDNATLTRFF

PFHFLFPFIIAALAVIHLVFLHNSGANNPFAFNSNYDKAPFHIYFTTKDTVGFILLVAAL

FSLALLFPGALNDPENFIPANPLVTPPHIQPEWYFLFAYAILRSIPNKLGGVIALVAAIL

VLFLMPLLNTSKNESNSFRPLSQAAFWLLVAHLFILTWIGSQPVEYPYVLLGQVASVLYF

SLFIFGFPIVSSIENKIIFS-

>L variegatus

MAAPIRKEHPIFRILKGTFVDLPLPSNLSI-WKFGSLLGLCLIVQILTGLFLAMHYTADI

TLAFSSVRHICRDVNYG-LLRKVHAKGASLFFICMYCHIGRGLYYGSYKKVET-NIGVIL

FLVTILTAFMGYVLV-GQMSFWAATVITNLVSAIPYIGTTIVQ--LGGFSVDKATLTRFF

AFHFLFPFIIAALAVIHLVFLHKRGANNPVGLKRNYDKAPFHIYYTTKDTVGFILLITAL

FSLALLFPGALKDPENFIPANPLVTPPHIQPE-YFLFAYAILRSIPNKLGGVIALVAAIL

VLFLMPLLNTSKKESNSFRPLSQAAF-LLVATFFILT-IGAQPVEYPYVFLGQVASVLYF

SLFIFGFPLVSSLEKKIIFS-

>M musculus

-MTNMRKTHPLFKIINHSFIDLPTPSNISSWWNFGSLLGICLMVQIITGLFLAMHYTSDT

MTAFSSVTHICRDVNYGWLIRYMHANGASMFFICLFLHVGRGLYYGSYTFMETWNIGVLL

LFAVMATAFMGYVLPWGQMSFWGATVITNLLSAIPYIGTTLVEWIWGGFSVDKATLTRFF

AFHFILPFIIAALAIVHLLFLHETGSNNPTGLNSNADKIPFHPYYTIKDILGILIMFLIL

MTLVLFFPDMLGDPDNYMPANPLNTPPHIKPEWYFLFAYAILRSIPNKLGGVLALVLSIL

ILALMPFLHTSKQRSLMFRPITQILYWILVANLLILTWIGGQPVEHPFIIIGQLASISYF

SIILILMPISGIIEDKMLKLY

CYTC

>H sapiens

-----------MGDVEKGKKIFIMKCSQCHTVEKGGKHKTGPNLHGLFGRKTGQAPGYSY

TAANKNKGIIWGEDTLMEYLENPKKYIPGTKMIFVGIKKKEERADLIAYLKKAT------

NE

>H glaber

-----------MGDVEKGKKIFVQKCAQCHTVEKGGKHKTGPNLHGLFGRKTGQAPGFSY

TDANKNKGITWGEDTLMEYLENPKKYIPGTKMIFTGIKKKGERADLIAYLKKAT------

NE

>M brandtii

-----------MGDVEKGKKIFVQKCAQCHTVEKGGNHKTGPNLHGLFGRKTGQAPGFSY

TDANKNKGITWGEATLMEYLENPKKYIPGTKMIFAGIKKSAERADLIAYLKKAT------

NE

>S franciscanus

LDMAANDDVIPPGDPEKGAEIFREHCAGCHDIDKSGEHRAGPNLMGLWGRKTGSAEGFKY

TEANKNKGITWSMDTLWVYLENPKAYIPGTKMVFAGLKKTKERADLIAYLKEKTTGNYHK

DD

>S purpuratus

MGKEPKATTPSVGDPDKGAKIFKVKCTQCHVIDNSGKHKQGPNLMGLWGRKTGSAEGFKY

TEANKNKGITWGLDTLWVYLENPKAYIPGTKMVFAGLKKTKERADLIAYLKEKTTGNYTK

DD

>L variegatus

SAEGNIDWSPVMGDPEKGAIIYAEICAQCHTIENSRKHKQGPNLSGIWGRKTGQAPGFKY

TDANKNKGITWGLDTLWIYLENPKAYIPGTKMVFAGLKKTKERADLIAYLKEKTTGNYHK

DD

>M musculus

-----------MGDVEKGKKIFVQKCAQCHTVEKGGKHKTGPNLHGLFGRKTGQAAGFSY

TDANKNKGITWGEDTLMEYLENPKKYIPGTKMIFAGIKKKGERADLIAYLKKAT------

NE

DAF9

>H sapiens

---------------------------------------------------------MAL

LLLLFLGLLGLWG----------------------LLCACAQDPSPAARWPP-GPRPLPL

VGNLHLLR-LSQQDRSLMELSERYGPVFTVHLGRQKTVVLTGFEAVKEALAGPGQELADR

PPIAIFQLIQ-----RGGGIFFSSGARWRAARQFTVRALHSLGVGREPVADKILQELKCL

SGQLDG--YRGRPFP-LALLGWAPSNITFALLFGRRFDYRDPVFVSLLGLIDEVMVLLGS

PGLQLFNVYPWLGALLQLHRPVLRKIEEVRAILRTLLEARRPHVCPGDP---------VC

SYVDALIQQGQGDDPEGLFAEANAVACTLDMVMAGTETTSATLQWAALLMGRHPDVQGRV

QEELDRVLGPGRTPRLEDQQALPYTSAVLHEVQRFITLLPH-VPRCTAADTQLGGFLLPK

GTPVIPLLTSVLLDETQWQTPGQFNPGHFLDANG-HFVKREAFLPFSAGR-RVCVGERLA

RTELFLLFAGLLQRYRLLPPPGVS-----PASLDTTPARAFTMRPRAQALCAVPRP----

------

>H glaber

-----------------------------------------------------MLASGLP

LVALLVFLSIVI-----------------------LMSVW-QHRRLHGKLPP-GPTPLPF

IGNYLQLNTEQM-YNSIMKIKEQYGPVFTIHLGPRRVVVLCGYDAVKEALIDQAEEFSGR

GKQATFDWIF----KGYGVAFSSWERAR-HLRRFSISTLRDFGMGKRGIEERIQEEIGFL

IESFRE--TQGAFIDPTFYLSRAVSNIISSIVFGDRFDYEDKEFLSLLRMMLGSFQFTAT

STGQLYEMFYS--VMKYLPG-PQQQAFKELQGLENFIVKKVEQNQRTLDPS--SPRNFID

SFLIRIQKNSNAE-----FHMKNLVMTTLSLFFAGTETVSTTLRYGFLLLMKHPDVEAKI

HEEIDRVIGRNRHPMFEDKAKMPYTDAVIHEIQRFGDMIPMNLAHSVTKDTKFRNFLLPK

GTEVFPVLGSVLRDPKFFSNPQDFHPQHFLDEKGQ-FKKNDAFVPFSLGK-RYCLGEGLA

RMELFLFLTSILQNFRFK-SPQARQDIDVSPKLVGFATI---PQNY--TMSFLPR-----

------

>M brandtii

---------------------------------------------------------MDL

FTVLAICLSCLI-----------------------LLFLW-NRSYAQGKLPP-GPTPLPV

VGNILQINMKNF-NESLRKLAEVYGPVFTVYVGTQPTVVLHGYAAVKEALIDRGEEFAGR

GSFPVIDYLS----RGLGVVFSNGDIWK-HTRRFSLTVLRNMGMGKKTIEDRIQEEALCL

VEALKK--TNASPCNPSLLLGCAPCNVICSVIFQSRFEYSDEKFLTLLHYFNENLRIIST

TWIQLYNAFPY--LMHYLPG-SHNEFFNNFTEQKKFILEQIKEHQESLDLN--NPRDFID

YFLIKMEKEKHNE--QSVFTMDNLITTIWDMFSAGTETTATTLKYGLLLLLKHPEVTAKV

QEEIDRVVGRERSPCMQDKSHMPYTDAVIHEIQRYIDLVPNNLPHLVNQDIKFRGYFIPK

GTAIVTSLTSLLHDDKEFPNPDQFDPGHFLDESGN-FKKSNNFMPFSAGRKRVCVGEGLA

RMELFLLLTHILQHFTLK-PLVDPKDIDTSPVVNGFGSV---PPSY--ELCFVPV-----

------

>S franciscanus

---------------------------------------------------------MDF

GISLTTFLLGMVTFL------------------VTFIMVRSGKSKKYKNLPPRGPREWPV

LGSLPSLAGKDMPHEAIADLAKKYGPIFSMRRGSFYFVVLNDRESIKQALVKSGEFFSDR

PKIAMIEHIA----EGKGKVAIYTSPAQIEQRKFTLSVFRSLGVGKKSYEDTIAAEMAQL

GKAIEE--KKGKPFDPNVLIGQAVANVICSIIFGTQYKYNDTDFQHVLAASYRIFEI--A

EIAGLFNFLPF---LRFLPGSGVKRMVALQAIFDKYMKPLVWKVNEEQEKSRRSPTCYVE

MYTEQMIEAERQTPGPHSFSKLNLKSAIADLFIAGTETTTTTLKWAILYMMAYPEVQSRV

QAEIDHVVGRERLPTLNDRKNLPYTEATLMEVLRYRPVAPLGVPHATTQDTVLGGYTIPK

GTIIIPNLWAVHHDPKEWDDPDKFNPDRFLSEDGKTVVKNEALIPFSIGR-RKCLGEQLA

KMELFLFFTHLLQRFEFKLPPGAP-----PPSMRGRNGITMNPEPY--KICAIPRRIKSN

IKN---

>S_purpuratus

---------------------------------------------------------MDF

GISLTTFLLGMVTFL------------------VTLMIVWSRTGRTYANLPPKGPPEWPI

IGSLLSLAGEHPPHQILANMAKKYGPIFSMQMGSFYAVVLSDYSLIKQAFAKSGDDFSDR

PKIAMIEHLA----EGKGLISGYNSPAQIEQRRFTFSALRSLGMGKFRMEETISEEIQHL

IKSFTE--RNTQPFIPFHDITVSVSNVICWLNFGKRFEYNDDDFKGVLEALFGIVEI--T

EIGGVFNFLPF---LRFLPGSGLKKCFKHKAIFDKHMYPLIRKIKQSYDDKFESAECYIH

MYADKIAEAAKQNIVPNLFNDGNMVLAVADLFVAGTETTATTLKWALLYMVVNQDVQKRV

QEELDRVVGVDRLPSLLDKPNLPYTEATLLEIQRKASIVPLGVPHAPVQDTILYGYDIPK

GTVVLPNLWAIHHDPNLWKNPDEFNPDRFLKPDTKHVVQREELIPFSIGR-RRCIGEELA

KVELYLFFTHLLCRFHFILPPGAP-----QPTMEGRSLATFVPHDY--SLCAIPRCRDKD

FEES--

>L variegatus

---------------------------------------------------------LRR

GSLIFKIMLGLVTFL------------------VTLMLIRSRTRRTQANFPPKGPPEWPI

IGSLLSLAGEHPPHETYANMAKKYGPIFSMQMGSFYAVVLSDYSLIRQAFAKSGEEFSDR

PKITMIENLV----QGKGLIVSYSSPVQTEQRRFTLSALRSLGMGKKSYEETIAEEARQL

AEAIEE--KRGKPFDPFHLIVVAVSNIICWIVFGRRYKYDDAEFKRILESVFESFEI--A

EMAGLFNFLPF---LRFLPGSGVKRMLELRAIFDKYMKPMVWKISEEQEKSRRSPTCYVE

MYTEQMIEAEQETPGPHTFDDTNLLQSIADLFFAGTETTATTLKWAILLMAAHPEVQSRV

QAEIDDVVGRDRLPTLSDRKNLPYTEATLMEVMRFRPVLPLGVPHMTSADVKLGGYDIPK

GTIVLPNLWAVHHDPKLWKDPEEFNPDRFLSEDGKTVVKNEALMPFGIGR-RDCLGEQLA

KMELFLFFTNLLQRFEFRLPPGAP-----PPSMFGHNGATLNPKPY--KICAIPR--STS

LAD---

>C elegans

---MHLENRVLSSVLDYASKFYKRMSSLVFFSANSIQVQMNISQSSWIKCRDWMAFALSH

HIIMGIYLLILRNFLPQVVPDFEWQHYFMRVFIVHIIYIIISYFIRITRYPP-GPPPMAV

FGNSPFVNILTP-EQTFLEYREIYGPIFTLHL-SQPTIILAEYKTIQEALVKNGQQTSGR

SSAESFVLFTGDRLNGDGVILAMRQKWK--DMRHEISRFMNKWYGA-PMDELVLHHTRCL

EQELAKIAETKSLIDLRDPLAGAIANVIQQITIGRNYMYQDQEFQTQLRDINAVVKEIMT

AEVFFVNCYPW---LRYLPE-GILRKWTNYKRSGFRLQQWFRTILEEHHVN-RHQGDFM-

SHMIDLQESKQEQ-----FRDLSIILTCGDMWTGGMETTVTTLRWGIIYLLNNPEVQAKC

QMEILDVFGNDI-PDMGKMNQTPYVRATLSEIQRLANVLPWAIPHKTIEECNIGGYDIPV

NTEIIPALGAVLFDPNVFESPKQFKPERFLDEEGK-YRVMEEFRPFGLGP-RVCLGERIA

RTELYLIFASLLQNFRFYLNRGDP--IPVAERVIG--GITAPPKPYATRVEYLGNRLIN-

------

>M musculus

---------------------------------------------------------MDP

ILVLVLTLSCLF-----------------------LLSLW-RQSSERGKLPP-GPTPLPI

IGNILQIDVKDI-CQSFTNLSKVYGPVYTLYLGRKPTVVLHGYEAVKEALVDHGEEFAGR

GRFPVFDKAT----NGMGLAFSKGNVWK-NTRRFSLMTLRNLGMGKRSIEDRVQEEARCL

VEELRK--TNGSPCDPTFILGCAPCNVICSIIFQDRFDYKDRDFLNLMEKLNEITKILSS

PWLQICNTYPA--LLDYCPG-SHKQFFKNYASIKNFLLEKIKEHEESLDVT--IPRDFID

YFLINGGQEDGNQ--PLQNRLEHLAITVTDLFSAGTETTSTTLRYAILLLLKYPHVTAKV

QEEIEHVIGKHRSPCMQDRSRMPYTDAMIHEVQRFIDLIPNSLPHEVTSDIKFRNYFIPK

GTTVITSLSSVLHDSTEFPNPEKFDPGHFLDENGK-FKKSDYFIPFSTGK-RICAGEGLA

RMELFLFLTSILQNFNLK-PLVHPKDIDVTPMLIGLASV---PPAF--QLCFIPS-----

------

>SIG

~~~---------------------------------------------------------

------------------------------------------------------------

----------------+-------------------------------------------

------------------------------------------------------------

------------------------------------------------------------

------------------------------------------------------------

------------------------------------------------------------

------------------------------------------------------------

------------------------------------------------------------

------------------------------------------------------------

--

EXO1

>H sapiens

MGIQGLLQFIKEASEPIHVRKYKGQVVAVDTYCWLHKGAIACAEKLAKGEPTDRYVGFCM

KFVNMLLSHGIKPILVFDGCTLPSKKEVERSRRERRQANLLKGKQLLREGKVSEARECFT

RSINITHAMAHKVIKAARSQGVDCLVA---PYEADAQLAYLNKAGIVQAIITEDSDLLAF

GCKKVILKMDQFGNGLEIDQARLGMCRQLGDVFTEEKFRYMCILSGCDYLSSLRGIGLAK

ACKVLRLANNPDIVKVIKKIGHYLKM-NITVPEDYINGFIRANNTFLYQLVFDPIKRKLI

PLNAYEDDVDPETLSYAGQ---YVDDSIALQIALGNKDINTFEQIDDYNPDTAMPAHSRS

HSWDDKTCQKSANVSSIWHRNYSPRPESGTVSDAPQLKENPSTVGVERVISTKGLNLPRK

SSIVKRPRSAELSEDDLLSQYSLSFTKKTKKNSSEGNKSLSFSEVFVPDLVNGPTNKKSV

STPPRTRNKFATFLQRKNE----ESGAVVVP-----------------------------

------------------------------------------------------------

---GTRSRFFCSS-DSTDCVSNKVSIQPLDETAVTDKENN--LHESEYGD----------

---------------------------------------------------------QEG

KRLVDTDVARNSSDDIPNNHIPGD-----------------------------------H

IPDKATVFTDEESYSFESSKFTRTISPPTLGTL------RSCF-----------------

-SWSGGLGDFSRT--PSPSPS---------------------------------------

----------TALQQFRRKSDSPTSLPENN--MSDVSQLKSEESSDDESHPLREEACSSQ

SQESGEFSLQSSNASKLSQCS----------SKDSDSEE------------SDCNIKLLD

-------SQSDQT---SKLRLSHFSKKDTPLRNKVPGLY--KS----SSADSLSTTKIKP

LGPARASGLSKKPASIQKRKHHNAENKPGLQIKLNELWKNFGFKKDSEKLPPCKK--PLS

PVRDNI--QLTPEAEEDIFNKPE-CG----------------------------------

----------------------------------------RVQR-----AIFQ-------

------------------------------------------------------------

--------

>H glaber

MGIQGLLQFIKEASEPIHVRKYKGQVVAVDTYCWLHKGAIACAEKLAKGEPTDKYVGFCM

KFVNMLLSHGIKPILIFDGCTLPSKKEVERSRRERRQSNLLKGKQLLREGKVSEARECFT

RSINITHAMAHKVIKAARTLGVDCLVA---PYEADAQLAYLNKAGIVQAIITEDSDLLAF

GCKKVILKMDQFGNGLEIDQSRLGMCRQLGDVFTEEKFRYMCILSGCDYLSSLRGIGLAK

ACKVLRLANNPDIEKVIKKIGHYLKM-NITVPEDYIKGFIRANNTFLYQLVFDPIKRKLV

PLNAYEDDIDPETLTYAGQ---YIDDSIALQIALGNKDINTFEQIDDYNPDTAMPSLLRS

HSWDDKACQKSPAVNSIWHRNYRPTLEMNIVSDVTRLEEKPSTVGMKQVISTKGLNLPRK

SSLVKRPRSEELSEDDLLNQY-FSFAKKTKKNGCENNKSLNSSEMFVPDLRNGTIHTESL

NTPPRTRNKFATFLQRKNE----ESGAVVVP-----------------------------

------------------------------------------------------------

---GTRSRFFSSSLDSADCVSKQTSSQPLSETAVREKVTD--LSESDCPD----------

---------------------------------------------------------LEG

KKLVDTQ----------AAHNSSG-----------------------------------E

SPDDVTVFAGEEPQFVRTGTFTRTISPPTLGTL------RSCF-----------------

-SWSGSLREFSNSLRTSPKPS---------------------------------------

----------TMSQQFQRKSGSPSSLPEDTN-MSDMSQSKSDESG-DEAQPLREMDLSSQ

SQKSMEFSPHSLNASELSQPS----------SKDSDSEE------------SDCNNKSLH

-------NQGNQN---SKQHLSHFSRKETLLRNKVPGLS--KS----SSVDCLSATKIKA

LVPARVSGLSRKPASIQRRNHRNAENKPGLQIKINELWKNFGFKKDSEKLVSYKK--PLS

PVKDNI--QLTPEAEEDIFNKPE-CA----------------------------------

----------------------------------------HVQR-----AIFQ-------

------------------------------------------------------------

--------

>M brandtii

MGIQGLLQFIKDASEPIHVRKYKGQVVAVDTYCWLHKGAIACAEKLAKGEPTDK------

----------------------------------RRQANLLKGKQLLREGKVSEARECFT

RCVNITHAMAHNVIKAARAQGVDCLVA---PYEADAQLAYLNKAGIVQAIVTEDSDLLAF

GCKKVILKMDQFGNGLEIDQARLGMCRQLGDVFTEEKFRYMCILSGCDYLSSLRGIGLAK

ACKVLRLANNPDIVKVIKKLGHYLKM-NITVPEDYIKGFIRANNTFLYQLVFDPIRRKLV

PLNDYGDDIDPAALSYAGR---YIDDSIALQIALGNKDINTFEQIDDYNPDTAMPAQSRS

RSWDDNTSQKPSGVHSIWHRNYCPRPELAPVPGATRLKENPSTAGREQ------------

----------ELSEDDLLSQYSLSFTKKTKKDGCEGSTSLNSSKMLMPDPVDGTTIKKTL

STPPTTRNKFAAFLQRKNQ----ESGAVVVP-----------------------------

------------------------------------------------------------

---GTRSRFFCNSLDSMDCVSKKASPQPLDETADTDKKTN--LNESDCLDDKKVVDLNES

DCLDDKVVDLKESDCLESKMLGELNEPDCLESKKVVDLNESDCLESKKVVDLNESDCLES

KKVVDLNQSDCLESKKLVDLNQSDCLESKKLVERNGAHTP-----------------SAQ

TPDGATVIADEESPSFKTIRFTKTISPPTLGTL------RSCF-----------------

-SWSGSLGDFSRT--PSPSPS---------------------------------------

----------TALHQFRRKGDSPSSLPGST--VSDVSQVKGDASG-DEALPVHELSWSSQ

SQESAELSPHSLSASTLPQPS----------RKDCSPEVPGLYRSSSLDSLSTTKIKALV

PARVSGLSKRPAR---AQKRKHHNAENKPGLQTKINELW--KNFGFKNSLDSLSTTKIKA

LVPARVSGLSKRPARAQKRKHHNAENKPGLQTKINELWKNFGFKKDSEKLPSCKKPDPLS

PVKDNI--QLTPETEEDIYHKPESCGAGGTRLVQKLEEVEKNQERTAVALEAARAETEML

EQQEGQYHKDLGELQWQQQELQDELLSLEDRLLYPQTQLARLKRTNAFKATFEICCDGPL

AIINSFRLGCVPTVPVSWWEINAAWGHTALLLMALSNTVGLEFQRYQLIPPADHSYLKSL

TEDPVELP

>S franciscanus

--------FF-----------------------------LLCS-----------YVRYCL

KYVNMLRALDIKPVMVFDGRNLPAKAGVEDSRREVNIIVMFY---------------HIF

QSLSKQFFIFSPMKQAVRSLGVDCIVA---PYEADAQLAYLEKNGFVQAVITEDSDLIAF

GCKQVIVKLDLAGNCMDVDSSRLNVAMKIGEKFTPEKFRYMCIVAGCDYLASLPGIGIGK

ARKLFQLTANPDITQVIKRMATTLKMKSTTVTPEYQEGFQQANNTFLYQLAFDPQKKKLQ

PLTPYSPEVTPSTLTYAGKVTRYCHEPQSKDKQLMIIETCIAKLI--FQP----------

--YDKKT---PRHLLSIWHKDFQPGPGLGCLKQDHI--EREGTKGKEITMYFMLFTNLLM

FCV-------EEEEVGAPSDYQLSSMYTTK--GQLDGNQWMESQRVIPETPDKDVVE---

------------------------------------------------------------

------------------------------------------------------------

------------------------------------------------------------

------------------------------------------------------------

------------------------------------------------------------

------------------------------------------------------------

------------------------------------------------------------

--------------------QSPSKIPSDVS-SPDTDDVSLNSSTASSPSPPPASSSSSS

LQTTKPFGVATASQSNNNAHS--------LWVGSKGATSGYFGGGEREMSQGVVRQKGMM

QEEEEWENTPPGRGGVSPYNRRRNRNLSGKRHNQFIPAC-TTSIHSKCAIFPNALEQSAF

LGKCKASGLSKKRRSSGKTKPDAT---PKGQTSLLAF---MAKPKLRDPLISQKGVSPLS

PNRANLCTDLTPETEGSLLSRQGRGF----------------------------------

----------------------------------------TTKR-----HIFE-------

------------------------------------------------------------

--------

>S purpuratus

MGIQGLLPFLKEASEPIHIRKYKGYTVGIDSYCWIHRGAVACATQLAKGEPADHYVRYCL

KYVNMLRALDIKPVMVFDGRNLPAKAGVEDSRRERRETYSKKGRQFLREGKASEARDCFV

KCINVTPEMALNVMKAVRSLGVDCIVA---PYEADAQLAYLEKNGFVQAVITEDSDLIAF

GCKKVIVKLDLAGNCMDVDSSRLNVAMKIGEKFTPEKFRYMCIVAGCDYLASLPGIGIGK

ARKLFQLTANPDITQVIKRMATTLKMKSTTVTPEYQEGFVQANNTFLYQLAFDPQKKKLQ

PLTPYSPEVTPATLTYAGK---YVDEVKAYQMALGNINFDTMERIADFNPMVNKP-----

--YDKKT---PRHLLSIWHKDFQPGPGLGCLNQDHI--EREVTKGKEITVEVK------Q

RLIQKKRQRDEEEEVGAPSDVQLSSMYTTK--GQPDGNQWMESQRVIPDTPDKDVVEE--

EMKPSKRNRFATKLTSRNRANIVNSGEVVTSRFFQPKKLDVSSEKQQQIPDNNKLKIEEK

ENQRVSMNTSGLLEGIVDIEMEQEKQFKMEDDDEDDIEDVIEIKLKETVSGSQSRQTHVT

KSIDALQRFQRSSSASVLVKTERNLTSSSLSNSVKPRTALSSTHQSEDIPDSQKSVIEIE

SSQDSVCSTRSVDTCSQSSSLSSSQRQGTPRPRSAFSWSRKKELQGKDKGSKQPTQGTLS

GFVFKPTHRSKAMPRHTAVGVQDGNSQGNSLSRRDSYEMPCSPVSQNSSSQLLRLSLTSS

IPGSMDMLRLGEDVTFMDSQSSNLTESQNTSSITLTSDQTSQL-----------------

-IDNTDLEDVSPPIPPLPQLP--------------------LQIHLSNGEEEEEEKGEMM

EGIRRTGLSRKRKKTFSSEEQSPGKIPSDVS-SPDTDDVSLNSSTASSPSPAPA-LSSSS

LQTTKHFGMATASQSNKDADS--------LRVGGRGATSCYFGGGEREMSQGVVRKKGMM

QEEEEWENTPPGRGGVSPYKGRRNRNLSGKRHNQGPKAAFMNDENQQNSSNSEPKKQSAF

LGQCKASGLSKKRRSSGKAKPDAT---PKGQTSLLAF---MAKPKTKDPLISQKGVSPLS

PNRANLCTDLTPETEGSLLSRQGRGF----------------------------------

----------------------------------------TTKR-----HIFE-------

------------------------------------------------------------

--------

>L variegatus

MGIQGLLPFLKEASEPIHIRKYKGYTLGIDTYCWIHRGAVACATQLAKGDPADQYVRYCL

KYVNMLRALDIKPVMVFDGRNLPAKAAVEDSRRERRETYSRKGRQFLREGKASEARDCFV

KCINVTPEMALDVMKVILHGKGSCNVEVSDSYQFSCLFVHFSQCR--QSI----SSVISL

SILQVIVKLDLAGNCMEVDSSRLNIAMKIGEKFTPEKFRYMCIIAGCDYLASLPGIGIGK

ARKLFQLTVNPDIKQVIKRMATTLKMKSTTVTPEYMEGFQQANNTFLYQLAYDPNKKKLQ

PLTPYPPEVGPSMLTYAGK------VEISFSHSCHSSNYCIFIIEFYIKHICHLPIY---

--HDKKT---PRYLLSIWHKDYQPGPGLGCLDQDHI--EREGTKGKEITVEVK------Q

RLIQKKRQREPIEPYYIYQKY-IMQRYANKKNFAINADIKNLCIYIIQHPMFASSHH---

CLKPAKRNRFATKLTSKKRANIVNSGEVVTSRLYHAKN----------------------

-----------------PITLESRIISGIHSAIYLLCHMVVSEKAKLLQEYCSKMKCMKI

WSLASKPRYIQIALHSMFASSPHYMIRIHMVTVSTPCISSLIRQEDNNMPLFTYLMTQVH

RQCNIAIIISKCNGYFLPTLFFILDFSANLILRIQWKISRETLHVKQMLVFVH----TEQ

RPVVRNALNKEMKGTQIHNTGLSDHRQTY----------------------------MSI

IPSISNIVIFVITKKIVSVVFTKVMLKSSLPTYFDARINNRIFPLHVIIWMAFFIDQYKS

SNWSASSVVVFSN--PTPNMPLSMKTWVSKLYFFLYLFDYTRNQRRHCYLFILLKKSRFF

SLVVTDLFFFNIFQKIPSFDQSPTKVRSEVS-SPDTDDVSLPSSTTSSPSPPPA---SAS

LNTTKVIAMETASQSKDSNHNDAVSGGLGLGTGDGPRSSGHSRGGETEASSGVVGKKGVV

MQEEEWENTPPGRGGVSPYKRHRNRSLSGRRHQVVTRAS-SISYTFSVHHQSYFTQQSAF

LGKCKASGLSKKRRLSGKGKPEAT---PKGQTSLLAF---MAKPKTRNPLISQKGVSPLS

PNRANIHADLTPETDGSLLSRQGRAL----------------------------------

----------------------------------------ATKR-----HIFE-------

------------------------------------------------------------

--------

>M musculus

MGIQGLLQFIQEASEPVNVKKYKGQAVAVDTYCWLHKGAIACAEKLAKGEPTDRYVGFCM

KFVNMLLSYGVKPILIFDGCTLPSKKEVERSRRERRQSNLLKGKQLLREGKVSEARDCFA

RSINITHAMAHKVIKAARALGVDCLVA---PYEADAQLAYLNKAGIVQAVITEDSDLLAF

GCKKVILKMDQFGNGLEVDQARLGMCKQLGDVFTEEKFRYMCILSGCDYLASLRGIGLAK

ACKVLRLANNPDIVKVIKKIGHYLRM-NITVPEDYITGFIRANNTFLYQLVFDPIQRKLV

PLNAYGDDVNPETLTYAGQ---YVGDSVALQIALGNRDVNTFEQIDDYSPDT-MPAHSRS

HSWNEKAGQKPPGTNSIWHKNYCPRLEVNSVSHAPQLKEKPSTLGLKQVISTKGLNLPRK

SCVLKRPRNEALAEDDLLSQYS-SVSKKIKENGCGDGTSPNSSKMSKSCPDSGTAHKTDA

HTPSKMRNKFATFLQRRNE----ESGAVVVP-----------------------------

------------------------------------------------------------

---GTRSRFFCSSQDFDNFIPKKESGQPLNETVATGKATTSLLGALDCPD----------

---------------------------------------------------------TEG

HKPVDAN----------GTHNLSS-----------------------------------Q

IPGNAAVSPEDEAQSSETSKLLGAMSPPSLGTL------RSCF-----------------

-SWSGTLREFSRT--PSPSAS---------------------------------------

----------TTLQQFRRKSDPPACLPEASAVVTDRCDSKSEMLG-ETSQPLHELGCSSR

SQESMD-SSCGLNTSSLSQPS----------SRDSGSEE------------SDCNNKSLD

-------NQGEQN---SKQHLPHFSKKDGLRRNKVPGLC--RS----SSMDSFSTTKIKP

LVPARVSGLSKKSGSMQTRKHHDVENKPGLQTKISELWKNFGFKKDSEKLPSCKK--PLS

PVKDNI--QLTPETEDEIFNKPE-CV----------------------------------

----------------------------------------RAQR-----AIFH-------

------------------------------------------------------------

--------

>SIG

------------------------------------------------------------

------------------------------------------------------------

------------------------------------------------------------

------------------------------------------------------------

------------------------------------------------------------

------------------------------------------------------------

------------------------------------------------------------

----------+-------------------------------------------------

------------------------------------------------------------

------------------------------------------------------------

------------------------------------------------------------

------------------------------------------------------------

------------------------------------------------------------

------------------------------------------------------------

------------------------------------------------------------

------------------------------------------------------------

------------------------------------------------------------

------------------------------------------------------------

------------------------------------------------------------

------------------------------------------------------------

------------------------------------------------------------

------------------------------------------------------------

FOXO1

>H sapiens

MAEAPQVVEIDPDFEPLPRPRSCTWPLPRPEFSQSNSATSSPAPSGSAAANPDAAAGLPS

ASAAAVSADFMSNLSLLEESEDFPQAPGSVAAAVAAAAAAAATGGLCGDFQGPEAGCLHP

APPQPPPPGPLSQHPPVPPAAA---GPLAGQPRKSSSSRRNAWGN----LSYADLITKAI

ESSAEKRLTLSQIYEWMVKSVPYFKDKGDSNSSAGWKNSIRHNLSLHSKFIRV--QNEGT

GKSSWWMLNPEGGKSGKSP--RRRAASMD-NNSKFAKSRSRAAKKKASLQSGQEGAGDSP

GSQF-SKWPASPGSHSNDDFDNWSTFRPRTSSNASTISGRLSPIMTEQDDLGEGDVHSMV

YPPSAAKMASTLPSLSEISNPEN----MENLLDNLNLLSSPTSLTVSTQSSPGTM-MQQT

PCYSFAPPNTSLNSPSPNYQKYTY----GQSSMSPLPQM-PIQTLQDNKSSYGGMSQYNC

APGLLKELLTSDSP-PHNDIMTPVDPGVAQPNSRVLGQNVMMGPNSVMSTYGSQASHNKM

MNPSSHTHPGHAQQTSAVNGRPLPHTVSTMPHTSGMNRLTQVKTPVQVPLPHPMQMSALG

GYSSVSSCNGYGRMGLLHQEKLPSDLDGMFIERL----DCDMESIIRNDLMDGDTLDFNF

DNVLPNQSFPHSVKTTTHSWVSG

>H glaber

MAEAPQVVEIDPDFEPLPRPRSCTWPLPRPEFNQSSSATSSPAPSGGTTGNPDAAAGLAP

APAAAVSADFMSSLSLLEESEDFPRAPGSVAAVVAAAAG----GGLCGDFQGPEAGCLHP

APPL-PPPGPLPQHPPVPAAAAAAAGALAGQPRKSSSSRRNAWGN----LSYADLITKAI

ESSAEKRLTLSQIYEWMVKSVPYFKDKGDSNSSAGWKNSIRHNLSLHSKFIRV--QNEGT

GKSSWWMLNPEGGKSGKSP--RRRAASMD-NNSKFAKSRGRAAKKKASLQSGQEGAGDSP

GSQF-SKWPASPGSHSNDDFDNWSTFRPRTSSNASTISGRLSPIMTEQDDLGDGDVHSLV

YPPSAAKMASTLPSLSEISNPEN----MENLLDNLNLLSSPTPLTVSTQSSPGSM-MQQT

TCYSFAPPNTSLNSPSPNYQKYTY----GQSSMSPLPQM-PMQTLQDNKSSYGGLNQYNC

APGLLKELLTSDSP-PHNDIMSPVDPGVAQPNSRVLGQNVIMGPNSVMPTYGNQASHNKM

MNPTSHTHPGHAQQTSAVNGRALPHPVNTMPHTSGMSRLTPVKTPLQVPLSHPMQMSSL-

GYSSVSSCNGYGRMGILHQEKLPSDLDGMFIERL----DCDMESIIRNDLMDGDTLDFNF

DNVLPNQSFSHSVKTTTHSWVSG

>M brandtii

------------------------------------------------------------

------------------------------------------------------------

------------------------------------------------------------

------------------------------------------------------------

------MLNPEGGKSGKSP--RRRAASMD-NNSKFSKSRGRAAKKKASLQSGPEGAGDSP

GSQF-SKWPASPGSHSNDDFDNWNTFRPRTSSNASTISGRLSPIMTEQDDLGDGDVHSMV

YPPSAAKMASTLPSLSEISNPEN----MENLLDNLNLLSSPPSLTVSTQSSPGAM-MQQT

PCYSFAPPNTSLNSPSPNYQKYTY----GQSSMSPLPQM-SMQTLQDNKSSYGGMNQYNC

APGLLKELLTSDSP-PHNDIMAPVDPGVAQPNSRVLGQNVLMGPNPVMPSYGSQASHNKM

MNPSSHSHPGHAQPTSVVNGRALPHAVNTMPHTSGMNRLAPVKTALQVPLPHPMQMNALG

GYSSMSSCNGYGRMGILHQEKLPSDLDGMFIERL----DCDMESIIRNDLMDGDTLDFNF

DNVLPNQSFPHSVKTTTHSWVSG

>S franciscanus

WARPNKTL----------RDRVCRGQRKWEEVCQ-------PYPRRGPRIRFCHHKLLII

LTPHSVPPS---------------------------------------------------

-----STEETRDTKPILPLEGGENRELSTPSSQR-NGPRRNAWGN----LS-ADLITKAI

QSAPDQRLTLSQIYDWMVKNVPIFKDKGDSNKT--------------RPTCLA--TDWSK

EGIGFWLFQKHCSCTSIKPFCCKPGWKITLVGSRFTSDMRRSRARPWLLSMPWTRPATLS

LDALTSSWRTT-NPSSRPLVTAPTTSRTLAFATKRSRFGTASGLFTFQASAMLLPMPSPV

IPLVSLNSFLSLMMSRTFGTPSTPFHIHPCIIDSL-LLSAPLLTTFRRCAQQMPHHQPKS

SGLSLRMTSVWPQQATPPCPGCPSSRRASPTNATPSRQTSASTTSFES---TSLASMVLC

IV-------------IAWSSRPPFEAACCRPYTLHTRASRKCAPEQNLPSFGQAPQLSSR

CGP-------DALPATACRPLSLPH-----PHPTGLPGLPIPVHCCLLSFPGPL------

----------LGHCGQVQQ--LANSRGGCCWGQWTHCCPAPYLCHVRHQAVIWGTIHVAP

DILPPLGCTPSCFFSCL------

>S_purpuratus

------MVDNDPDFEPQARPRSCTWPLRRPDFLDSK-----PGQPGNAAGAPPVDHAHSA

LSPAVLA-----------------------------------------------------

-------EEPLDIKPVLPLEGGENRELSTPSSQRRNGSRRNAWGN----LSYADLITKAI

QSAPDQRLTLSQIYDWMVKNVPFFKDKGDSNSSAGWKNSIRHNLSLHSRFVRV--QNEGT

GKSSWXMINPDA-KPGKSS--RRRASSMDTTNSKFERKRGRVKKKVLEERAKWGNTSPTP

------KLEGEEGASPLPFNLATTDFRSRASSNASS-CGRLSPIMTTHP---EIDMHDNE

VPPMSPIPFQDLPPSQAYDSPDP-----YQSTDQLAKLAKAMTLDSSLSVEPAIRHTHNN

GGYLFSPQSYSGSDMSPVHSNSPYYSQQGTPAVSPLGQCSPMQELPPSQ--YGMQRSFTN

LIHENESIIPQDPMFAQSAVLRQQQSPRPMPSCREESMNQHTSPHRLMPSSMNQGSNLAM

L-----LNNGHNTHTTSHHHHPQPYPNGGTPHHVHPHHIHP-----HIHHHHP-------

--------------GMVHVDRFPSDLESVQIDPLKGWSDLDVETILRNEQDLTEGPDASF

DNIGTIGTTATTM--AAPSWVH-

>L variegatus

--NTFKMI----------------------------------------------------

------------------------------------------------------------

-------------------------------------------------YCRDEYISCII

RSTIN--LQIEDQFSFLLYKC--------------------------AQLCCS-------

------------------------------------------------------------

----------------------------------------------------QIDLHVLV

------RLSSSQCTISILKRNTD---------------------------------LNTY

TCFIF----------------------------------------------YLLLHLICC

A--------------------------------------------KFLHNY---------

-----------------------------------LSKISISSSSLAFSMQNS-------

---------------------------------------------IRHNL----SLHSRF

VRVQNEGTGKSS-----------

>M musculus

MAEAPQVVETDPDFEPLPRQRSCTWPLPRPEFNQSNSTTSSPAPSGGAAANPDAAASL--

ASASAVSTDFMSNLSLLEESEDFARAPGCVAVAAAAAAS----RGLCGDFQGPEAGCVHP

APPQPPPTGPLSQPPPVPPSAAAAAGPLAGQPRKTSSSRRNAWGN----LSYADLITKAI

ESSAEKRLTLSQIYEWMVKSVPYFKDKGDSNSSAGWKNSIRHNLSLHSKFIRV--QNEGT

GKSSWWMLNPEGGKSGKSP--RRRAASMD-NNSKFAKSRGRAAKKKASLQSGQEGPGDSP

GSQF-SKWPASPGSHSNDDFDNWSTFRPRTSSNASTISGRLSPIMTEQDDLGDGDVHSLV

YPPSAAKMASTLPSLSEISNPEN----MENLLDNLNLLSSPTSLTVSTQSSPGSM-MQQT

PCYSFAPPNTSLNSPSPNYSKYTY----GQSSMSPLPQM-PMQTLQDSKSSYGGLNQYNC

APGLLKELLTSDSP-PHNDIMSPVDPGVAQPNSRVLGQNVMMGPNSVMPAYGSQASHNKM

MNPSSHTHPGHAQQTASVNGRTLPHVVNTMPHTSAMNRLTPVKTPLQVPLSHPMQMSALG

SYSSVSSCNGYGRMGVLHQEKLPSDLDGMFIERL----DCDMESIIRNDLMDGDTLDFNF

DNVLPNQSFPHSVKTTTHSWVSG

FOXO3

>H sapiens

MAEAPASPAPLSPLEVELDPEFEPQSRPRSCTWPLQRPELQASPAKPSGETAADSMIPEE

EDDEDDEDGGGRAG-SAMAIGGGGGSGTLGSGLLLEDSARVLAPGGQDPGSGPATAAGGL

SGGTQA---LLQPQQPLPPPQPGAAGGSGQPRKCSSRRNAWGN----LSYADLITRAIES

SPDKRLTLSQIYEWMVRCVPYFKDK------GDSNSSAGWKNSIRHNLSLHSRFMRVQN-

-EGTGKSSWWIINPDGGKSGKAP--RRRAVSMDNSN-KYTKSRGRAAKKKAALQT-----

--------APESADDSPSQLSKWPGSPTSRSSDELDAWTDFRSRTNSNASTVSGRLSPIM

ASTELDEVQDDD-APLSPMLYSSS-ASLSPSVSKPCTVE-----LPRLTDMAGTMNLNDG

LTENLMDDLLDNITLPPSQPSPTGGLMQRSSSFPYTTKGSGLGSPTSSFNSTVFGPSSLN

SLRQSPMQTIQENKPATFSSMSHYGNQTLQDLLTSDSLSHSDVMMTQSDP--LMSQASTA

VSAQNSRRNVMLRNDPMMSFAAQPNQGSLVNQNLLHHQHQTQGALGGSRALSNSVSNMGL

SESSSLGSAKHQQQSPVSQSMQTLSDSLSGSSLYSTSANLPVMGHEKFPSDLDLDMFNGS

LECDMESIIRSELMDADGLDFNFDSLISTQNVVGLNVGNFTGAKQASSQSWVPG

>H glaber

MAETSASPIPLSPPEVEVDPEFEPQSRPRSCTWPLQRPELQASPAKPSGETAADSMIPEE

EDDEDDED-SGRTS-SAMAIGGG-GSGTLGSGLLLEDSARLLASGGQDFGSGPAPAVGAL

SGGTQT---PLQPQQPLPAPQPGAAGSSGQPRKCSSRRNAWGN----LSYADLITRAIES

SPDKRLTLSQIYEWMVRCVPYFKDK------GDSNSSAGWKNSIRHNLSLHSRFMRVQN-

-EGTGKSSWWIINPDGGKSGKAP--RRRAVSMDNSN-KYTKSRGRAAKKKAALQT-----

--------APESADDSPSQLSKWPGSPTSRSSDELDAWTDFRSRTNSNASTVSGRLSPIL

ATTELDDVQDDD-APLSPMLYSSS-ASLSPSVSKPCTVE-----LPRLTDMAGTMNLNDG

LADNLMDDLLDNITLQSSQPSPTGALMQRSSSFPYTTKGSGLGSPTSSFNSTVFGPSSLN

SLRQSPMQTIQENKPATFSSMSHYGNQTLQDLLTSDSLSHSDVMMTQSDP--LMSQASTA

VSAQNSRRNVMLRNDPMMSFAAQPNQGSLVNQNLLHHQHQTQGALGGSRALSNSVSNMGL

SDSSSLGSAKHQQQSLVSQSMQTLSDSLSGSSLYSASANLPVMGHEKFPSDLDLDMFNGS

LECDMESIIRSELMDADGLDFNFDSLISTQNVVGLNVGNFTGAKQASSQSWVPG

>M brandtii

------------------------------------------------------------

------------------------------------------------------------

------------------------------------------------------------

--------------MVRCVPYFNDK------GDSNSSAGWKNSIRHNLSLHSRFMRVQN-

-EGTGKSSWWIINPDGGKSGKAP--RRRAVSMDNSN-KYTKSRSRAAKKKAALQT-----

--------APESADDSPSQLSKWPGSPTSRSSDELDAWTDFRSRTNSNASTVSGRLSPIL

AGTELDDVQDDD-APLSPMLYSSS-ASLSPSVSKPCTVE-----LPRLTDMAGTMNLNDG

LADNLMDDLLDNIPLPPSQPSPAAGLMQRSSSFPYTTKGSGLGSPTSSFNSTVFGPSSLN

SLRQSPMQTIQENKPATFSSMPHYGTQTLQDLLTSDSLSHSDVMMTQSDP--LMSQASTA

VSAQNSRRNVMLRNDPMMSFAAQPNQGSLVNQNLLHHQHQTQGALSGSRALSNSVSNMGL

SDSSSLGSAKHQQQSLVSQSMQTLSDSLSGSSLYSTSANLSVMGHEKFPSDLDLDMFNGS

LECDMESIIRSDLMDADGLDFNFDSLISTQNVVGLNVGNFTGAKQASSQSWVPG

>S franciscanus

WARPNKT---LRDRVCRGQRKWEEVCQP----YPRRGPRIRFCHHKLLIILTPHSVPPSS

TEE---------------------------------------------------------

----TR---DTKPILPLEGGENRELSTPSSQR-NGPRRNAWGN----LS-ADLITKAIQS

APDQRLTLSQIYDWMVKNVPIFKDK------GDSNKT--------RPTCLATDWSK----

-EGIG---FWLFQKHCSCTSIKPFCCKPGWKITLVGSRFTSDMRRSRARPWLLSMPWTRP

ATLSLDALTSSWRTTNPSSRPLVTAPTTSRTLAFATKRSRFGTASGLFTFQASAMLLPM-

-PSPVIPLVSLN-SFLSLMMSRTFGTPSTPFHIHPCIIDSLLLSAPLLTTFRRCAQ----

-------------QMPHHQPKSSGLSLRMTSVWPQQATPPCPGCPSSRRAS----PTNAT

PSRQTS------------ASTTSFESTSLASMVLC-------------------------

---------------IVIAWSSRPPFEAACCRPYTLHTRASRKCAP--------------

------------EQNLPSFGQAPQLSSRCGPDALPATACRPLSLPHPHPTGLP------G

LPIPVHCCLLSF------------------------------------------

>S purpuratus

--------------MVDNDPDFEPQARPRSCTWPLRRPDFLDSKPGQPGNAAGAPPVDHA

HSA-------------------------LSPAVLAEE-----------------------

----PL---DIKPVLPLEGGENRELSTPSSQRRNGSRRNAWGN----LSYADLITKAIQS

APDQRLTLSQIYDWMVKNVPFFKDK------GDSNSSAGWKNSIRHNLSLHSRFVRVQN-

-EGTGKSSWWMINPD-AKPGKSS--RRRASSMDTTNSKFERKRGRV--KKKVLEE-----

--------RAKWGNTSPT--PKLEGEEGASPLPFNLATTDFRSRASSNASSC-GRLSPIM

TTHPEIDMHDNEVPPMSPIPFQDL-PPSQAYDSPDPYQS-----TDQLAKLAKAMTLDSS

LSVE--------------------------PAIRHTHNNGGY----------LFSPQSYS

GSDMSPVH----------SNSPYYSQQGTPAVSP----------LGQCSP--MQELPPSQ

YGMQRSFTNLIHENESIIP------QDPMFAQSAVLRQQQSPRPMPSCRE----------

-------ESMNQHTSPHRLMPSSMNQGSNLAMLLNNGHNTHTTSHHHHPQPYP----NGG

TPHXVHPQC---------------------------------------------

>L variegatus

-----------------------------------NTFKMIYCRDEYISCIIRSTINLQI

EDQ---------------------------FSFLLYKCAQLCCS----------------

----QI---DLHVLVRLSSSQ------------CT---------------ISILKRNTDL

NTYTCFIFYLLLH-LICCAKFLHNYLSKISISSSSLAFSMQNSIRHNLSLHSRFVRVQN-

-EGTGKSS----------------------------------------------------

------------------------------------------------------------

------------------------------------------------------------

------------------------------------------------------------

------------------------------------------------------------

------------------------------------------------------------

------------------------------------------------------------

------------------------------------------------------

>M musculus

MAEAPASPVPLSPLEVELDPEFEPQSRPRSCTWPLQRPELQASPAKPSGETAADSMIPEE

DDDEDDEDGGGRAS-SAMVIGGG-VSSTLGSGLLLEDSAMLLAPGGQDLGSGPASAAGAL

SGGTPT---QLQPQQPLPQPQPGAAGGSGQPRKCSSRRNAWGN----LSYADLITRAIES

SPDKRLTLSQIYEWMVRCVPYFKDK------GDSNSSAGWKNSIRHNLSLHSRFMRVQN-

-EGTGKSSWWIINPDGGKSGKAP--RRRAVSMDNSN-KYTKSRGRAAKKKAALQA-----

--------APESADDSPSQLSKWPGSPTSRSSDELDAWTDFRSRTNSNASTVSGRLSPIL

ASTELDDVQDDD-GPLSPMLYSSS-ASLSPSVSKPCTVE-----LPRLTDMAGTMNLNDG

LAENLMDDLLDNIALPPSQPSPPGGLMQRGSSFPYTAKSSGLGSPTGSFNSTVFGPSSLN

SLRQSPMQTIQENRPATFSSVSHYGNQTLQDLLASDSLSHSDVMMTQSDP--LMSQASTA

VSAQNARRNVMLRNDPMMSFAAQPTQGSLVNQNLLHHQHQTQGALGGSRALSNSVSNMGL

SDSSSLGSAKHQQQSPASQSMQTLSDSLSGSSLYSASANLPVMGHDKFPSDLDLDMFNGS

LECDMESIIRSELMDADGLDFNFDSLISTQNVVGLNVGNFTGAKQASSQSWVPG

FOXO4

>H sapiens

MDPGNENSATEAAAIIDLDPDFEPQSRPRS--------CTWPLPRPE-IANQPSEP----

--PEVEPDLGEK--------VHTEGRSEP-----------------------------IL

LPSRLPEPAGGPQPGILGAVTGPRKGGSRRNAWGN----QSYAELISQAIESAPEKRLTL

AQIYEWMVRTVPYFKDKGDSNSSAGWKNSIRHNLSLHSKFIKVHN--EATGKSSWWMLNP

EGGKSGKAP--RRRAASMDSSSKLLRGRSKAPKKKPSVLPAPPEGATPTSPVGHFAKW-S

GSPCSRNREEADMWTTFRPRSSSNASSVSTRLSPL---RPESEVLAEE-IPASVSSYAGG

VPPTLNEGLELLDGLNLTSSHSLLS-RSGLSGFSLQHPGVTGPLHTYSSSLFSPAEGPLS

AGEGCFSSSQALEALLTSDTPPPPADVLMTQVDPI------LSQAPTLLLLGGLPSSSKL

ATGVGLCPKPLEAPG-----------PSSLVP--TLSMIAPPPVMASAPIPKALGTPVLT

PPTEAASQDR-MPQDLDLDMYMENLECDMDNIISDLMDE------GEGLDFNFEPDP---

----------------------------

>H glaber

MDPGNKNSATEAAAIVDLDPDFEPHSRPRS--------CTWPLPRPE-LATEPSEP----

--PEVEPGLGQK--------VHPEGRSEP-----------------------------IL

LPSQLPEPAGGPQPGILGAVTGPRKGGSRRNAWGN----QSYAELISQAIESAPEKRLTL

SQIYEWMVRTVPYFKDKGDSNSSAGWKNSIRHNLSLHSKFIKVHN--EATGKSSWWMLNP

EGGKSGKAP--RRRAASMDSSSKLLRGRSKAPKKKPSVLPAPPEGATPTSPISHFAKW-S

GSPCSRSREEADMWTTFRPRSSSNASTVSTRLSPM---RPESEVLAEEEMPASVSSYAGG

VP-TLNEDLELLDGLNLTSPHSLLS-RSSLSGFSLQHAGVTGPLHTYSTSLFSPAEGPLS

PE-GCFSSSQSLEALLTSDKPPPPADVLMTQVDPI------LSQAPTLLLLGGIPSSSKL

STGVGLCPKPLEAPG-----------PSSLVP--SLPIMVPPPVMAGAPIPKALGAPVLT

PSTEAAGQDR-MPQDLDLDMYMENLECDMDNIISDLMDG------GEGLDFNFEPDP---

----------------------------

>M brandtii

MDPENENSATEAAAIIDLDPDFEPQSRPRS--------CTWPLPRPE-LATQPSKP----

--PEVDPGLGEK--------VHTEGRTEP-----------------------------IL

LPSRLPEPAGGPQSGILGAVTGPRKGGSRRNAWGN----QSYAELISQAIESAPEKRLTL

AQIYEWMVRTVPYFKDKGDSNSSAGWKNSIRHNLSLHSKFIKVHN--EATGKSSWWMLNP

EGGKSGKAP--RRRAASMDSSSKLLRGRSKAPKKKPVVLPAPPEGATPKSPVGHFAKW-S

GSPCSRNREEADVWTTFRPRSSSNASTVSTRLSPL---RPESEVLAGEEMPASVSSYAGG

VPPTLKEDLELLDGLNITSSHSLLS-RSSLSGFSLQHPGVTGPLHTYSTSLFSPAEGPLS

AGEGCFSSSQSLEALLTSDTPPPPANVLMTQVDPI------LSQAPTLLLLGGIPSSSKV

ATGVGLCPKPPEAPG-----------PSSLIA--PLPMIAPPPVMVGAPIPKTLGTPMLT

PSTEATSQAR-MPQDLDLDMYLENLECDMDNIISDLMDG------EEGLDFNFEPGFVIV

TCCKHYRHYWGKHGEGVHQQESSSGKQY

>S franciscanus

--------------WARPN----KTLRDRV--------CRGQRKWEEVCQPYPRRG----

--PRIRFCHHKLLIILTPHSVPPSSTEET-----------------------------RD

TKPILPLEGGENRE--LSTPSSQR-NGPRRNAWGN----LS-ADLITKAIQSAPDQRLTL

SQIYDWMVKNVPIFKDKGDSNKT--------RPTCLATDWSK-----EGIG---FWLFQK

HCSCTSIKPFCCKPGWKITLVGSRFTSDMRRSRARPWLLSMPWTRPATLSLDALTSSWRT

TNPSSRPLVTAP--TTSRTLAFATKRSRFGTASGLFTFQASAMLLP---MPSPVIPLV--

---SLNSFLSLMMSRTFGTPSTPFHIHPCIIDSLLLSAPLLTTFRRCAQQM--PHHQPKS

SG----LSLRMTSVWPQQATPPCPGCPSSRRASPTNATPSRQTSASTTSFESTSLASMVL

CIVIAWSSRPPFEAACCRPYTLHTRASRKCAPEQNLPSFGQAPQLSSRCGPDALPATACR

PLS--------LPHPHPTGL--------------------------PGLPIPVHCCLLSF

----------------------------

>S purpuratus

--------------MVDNDPDFEPQARPRS--------CTWPLRRPDFLDSKPGQPGNAA

GAPPVDHAHSAL--------SPAVLAEEP-----------------------------LD

IKPVLPLEGGENRE--LSTPSSQRRNGSRRNAWGN----LSYADLITKAIQSAPDQRLTL

SQIYDWMVKNVPFFKDKGDSNSSAGWKNSIRHNLSLHSRFVRVQN--EGTGKSSWWMINP

D-AKPGKSS--RRRASSMDTTNSKFERKRGRVKKKVLEERAKWGNTSPTPKLE--GEE-G

ASPLPFNLATTD----FRSRASSNASSCG-RLSPIMTTHPEIDMHDNEVPPMSPIPFQ-D

LPPS--QAYDSPDPYQSTDQLAKLA-KAMTLDSSLSVEPAIRHTHNNGGYLFSP------

------------QSYSGSDMSPVHSN------SPY------YSQQ-------GTPAVSPL

GQCSPMQELPPSQYGMQRSFTNLIHENESIIP--QDPMFAQSAVLRQQQSPRPMPSCREE

SMNQHTSPHRLMPSSMNQGSNLAMLLNNGHNTHTTSHHHHPQPYPNGGTPHXVHPQC---

----------------------------

>L variegatus

-----------------------NTFKMIY--------C-----RDEYISCIIRSTINLQ

IEDQFSFLLYKC----------AQLCCSQ-----------------------------ID

LHVLVRLSSS------QCTISILKRNTDLNT-YTC----FIFY-LLLHLICCAKFLHNYL

SKI------------SISSSSLAFSMQNSIRHNLSLHSRFVRVQN--EGTGKSS------

------------------------------------------------------------

------------------------------------------------------------

------------------------------------------------------------

------------------------------------------------------------

------------------------------------------------------------

------------------------------------------------------------

----------------------------

>M musculus

MDPENKKSATGAAAILDLDPDFEPQSRPRS--------CTWPLPRPD-LATEPHEP----

--SEVEPSLGQK--------VPTEGHSEP-----------------------------IL

LPSRLPEPAGGPQPGILGAVTGPRKGGSRRNAWGN----QSYAELISQAIESAPEKRLTL

AQIYEWMVRTVPYFKDKGDSNSSAGWKNSIRHNLSLHSKFIKVHN--EATGKSSWWMLNP

DGGKGGKAP--RRRAASMDSSSKLLRGRSKGPKKKPSVLPAPPEGATPRSPLGHFAKW-S

SSPCPRNREEADVWTTFRPRSSSNASTVSTRLSPM---RPESEVLAEEEMPASASSYAGG

VPPTLSEDLELLDGLNLASPHSLLS-RSGLSGFSLQHPGLAGPLHSYGASLFGPIDGSLS

AGEGCFSSSQSLEALLTSDTPPPPADVLMTQVDPI------LSQAPTLLLLGGMPSSSKL

GTGVSLCPTPLEGPG-----------PSNLVP--NLSVMAPPPVMAGAPIPKVLGTPVLA

SPTEDSSHDR-MPQDLDLDMYMENLECDMDNIISDLMDG-------EGLDFNFEPDP---

GPx

>H sapiens

----------------------------------------------------MARLLQAS

CLLSLLLAGFVSQSRGQEKSKMDCHGGISGTIYEYGALTIDGEEYIPFKQYAGKYVLFVN

VASYXGLTGQYIELNALQEELAPFGLVILGFPCNQFGKQEPGENSEILPTLKYVRPGGGF

VPNFQLFEKG-DVNGEKEQKFYTFLK---NSCPPTSELLGTSDRLFWEPMKVHDIRWNFE

KFLVGPDGIPIMRWHHRTTVSNVKMDILSYMR---RQAALGVKRK------

>H glaber

MLLAPEIPASLRLAGGPCLAMDWPSLRPEKWLQGSDAQTDGQPGCQTVLNAAMARLLRAS

CLLSLLLAGSVPWSLGQEKSKTECHGGVSGTIYEYGALTIDGEEYIPFKKYSGKYILFVN

VASYXGLTDQYLELNALQEELEPFGLVVLGFPCNQFGKQEPGENSEILPSLKYVRPGRGF

VPNFQLFEKG-DVNGEKEQKFYTFLK---NSCPPTAELLGSPSRLFWEPMKIHDIRWNFE

KFLVGPDGKPVMRWYHRTTVGSVKVDILAHMR---RQAALLAPGK------

>M brandtii

------------------------------------------------------------

---------------------MDCYKDVKGTIYEYDALTLDGKEHISFKQYVGKHVLFVN

VATYCGLTAQYPELNTLQEELKPFGLVVLGFPCNQFGKQEPGENLEILPGLKYVRPGGGY

VPNFQLFEKG-DVNGEKEQKVFTFLK---HSCPHPSELLGSFKHISWEPIKVQDIRWNFE

KFLVGPDGVPVMRWFHRAPVSAVKSDILLYLK--------QFKTK------

>S franciscanus

----------------------------------------------------FKMATYKQ

IVFAFFALAVCSRLSEATTLDAVCYDDAK-SLSD-----VAITERLSLDGYRGKVVLVVN

TASFCTYTHQYPYFNKLQNHFGD-QLVILGFPCNQFSLQEPGMGQEIPNTLRYVRPGGGY

KPNFYLNKEKIDVNGPKAHPLFQKMK---NSCPPVKIEIGDPSDLYWSPMTIGDITWNFN

KFLLDKEGVPFKRYDSAVEPLQLVHDIQLILDGAYKMVKRPSRPKYAREAL

>S purpuratus

--------------------------------------------MRGCSFSFFKMATYEK

IILAFFALVVCSRLSGATMLDAVCYDDAE-SLSD-----VAMTKSLSLDDYRGKVVLVVN

TASFCTYTYQYPYFNELKNEFGD-QLAILGFPCNQFWLQEPGVGQEIPNTLRYVRPGGGY

EPNFYLNEEKIDVNGPKAHPLFKKLK---NSCPPVKMEIGDPSNLYWSPMTIGDVTWNFN

KFLLDKEGVPFKRYDSVVEPLQLVSDIQLVVDGDYKTVKRPSRPKYATEEL

>L variegatus

---------------------------------------------NINPVDIFKMALNDE

VILIVLALALFPYVSKATMIDSVCYDDAE-SLHE-----VEITESLSLGDYRGKVVLVVN

TASFCTYTHQYPLFNKLQDQFDD-ELAILGFPCNQFGLQEPGLGEEIPNTLRYVRPGGGY

QPNFYLNEKKIDVNGPNAHPLFTKLKVYIHKKTPIIFYIGP--DWPYQERGGGRAIW--K

QTLTNNGGDEGRRKTGRRKIKSIRLNLFHINIKRFQICIMITRGKCIEPT-

>M musculus

----------------------------------------------------MARILRAS

CLLSLLLAGFVPPGRGQEKSKTDCHGGMSGTIYEYGALTIDGEEYIPFKQYAGKYILFVN

VASYXGLTDQYLELNALQEELGPFGLVILGFPSNQFGKQEPGENSEILPSLKYVRPGGGF

VPNFQLFEKG-DVNGEKEQKFYTFLK---NSCPPTAELLGSPGRLFWEPMKIHDIRWNFE

KFLVGPDGIPVMRWYHRTTVSNVKMDILSYMR---RQAALSARGK------

IGF1R

>H sapiens

------------------------------------------------------------

--------------------------------MKSGSGGGS---PTSLWG---LLFLS--

------AALSLWP----------------------TSGEICGPGIDIRNDYQQLK-----

-----RLENCTVIEGYLHILLI--SKAEDYRSYR--------------FPKLTVITEYLL

LFRVAGLESLGDLFPNLTVIRGWKLFYNYALVIFEMTNLKDIGLYNLRNITRGAIRIEKN

ADLCYLSTVDWSLI-LDAVSNNYIV-----GNKPP-KECGDLCPGTMEEKPMCEKTTINN

-----EYNYRCWTTNRCQKMC------PSTCGKRACTENNECCHPECLGSCSAPDNDTAC

VACRHYYYAGVCVPACPPNTYRFEGWRCVDRDFCANILSAESSD-SEG---FVIHDGECM

QECPSGFIRNGSQSMYCIPCEGPCPKVC-EEEKKTKTIDSVTSAQMLQGCTIFKGNLLIN

IRRG--NNIASELENFMGLIEVVTGYVKIRHSHALVSLSFLKNLRLILGEE--QLEGNYS

FYVLDNQNLQQLWDW-DHRNLTIKAGKMYFAFNPKLCVSEIYRMEEVTGTKGRQSKGDIN

TRNNGERASCESDVLHFTSTTTSKNR--IIITWH--RYR-PPDYRDLISFTVYYKEAP--

FKNVTEYDGQDACGSNSWNMVDVDLP-----PNKDVEPGILLHG-LKPWTQYAVYVKAVT

LTMVENDHIRGAKSEILYIRTNASVPSIPLDVLSASNSSSQLIVKWNPPSLPNGNLSYYI

VRWQRQPQDGYLYRH-----NY-----------------CSKD-KIPIRKYADGTIDIEE

VTENPKTEVCGGEKGPCCACPKTEAEKQAEKEEAEYRKVFENFLHNSIFV--PRPERKRR

DVMQVANTTMS----------------------------SRSRNTTAADTYNITDPEELE

TEYPFF------------------ESRVDNKERTVISNLRP-----FTLYRIDIHSCNHE

AE-KLGCSASNFVFARTMPA--EGADDIPGPVTWEPRPENS--IFLKWPEPENPNGLILM

YEIKYG------SQVEDQR-----------------------------ECVSRQEY-RKY

GGAKLNRLNPGNYTARIQATSLSGNGSWTDPVFFYVQAKTGYENFIHLIIALP-------

---VAVLL----IVGGLVIM----LYVFHRKRNNSRLGNGVLYASVNPEYFSAAD-----

VYVPDEWEVAREKITMSRELGQGSFGMVYEGVAKGVVKDEPE--TRVAIKTVNEAASMRE

RIEFLNEASVMKEFNCHHVVRLLGVVSQGQPTLVIMELMTRGDLKSYLRSLRPEME-NNP

VL---APPSLSKMIQMAGEIADGMAYLNANKFVHRDLAARNCMVAEDFTVKIGDFGMTRD

IYETDYYRKGGKGLLPVRWMSPESLKDGVFTTYSDVWSFGVVLWEIATLAEQPYQGLSNE

QVLRFVMEG-GLLDKPDNCPDMLFELMRMCWQYNP-KMRPSFLEIISSIKE---------

------------------------------------------------------------

---------------------------E--MEPGFREV-----------------SFYYS

EENK-----LP----EPEELDL----------EPENMESVPLDPS---------------

---------------------------------------ASSSSLPLPDRHSGHKA----

------ENGPGPGVLV--------------------------------------------

----------------LRASFDERQPYAHMNGGRKNERALPLP-------------QSST

C--------------------------------------------

>H glaber

------------------------------------------------------------

--------------------------------MKSGSGGGP---PTSLWG---LLFLS--

------AALSLWP----------------------TSGEICGPGIDIRNDYQQLK-----

-----RLENCTVIEGYLHILLI--SKAEDYRSYR--------------FPKLTVITEYLL

LFRVAGLESLGDLFPNLTVIRGWKLFYNYALVIFEMTNLKDIGLYNLRNITRGAIRIEKN

ADLCYLSTVDWSLI-LDAVSNNYIV-----GNKPP-KECGDLCPGTMEEKPLCEKTTINN

-----EYNYRCWTTNRCQKMC------PSACGKRACTESNECCHPECLGSCRAPDDDTAC

VACRHFYYAGVCVPVCPPGTYRFEGWRCVDRDFCANIPNAESSDSSEG---FVIHDGECM

QECPSGFIRNGSQSMYCIPCEGPCPKVC-EEEKKTKTIDSVTSAQMLQGCTIFKGNLLIN

IRRG--NNIASELENFMGLIEVVTGYVKIRHSHALVSLSFLKNLRQILGEE--QLEGNYS

FYVLDNQNLQQLWDW-DHRNLTIKSGKMYFAFNPKLCVSEIYRMEEVTGTKGRQSKGDIN

TRNNGERASCESDVLHFTSTTTWKNR--IIITWR--RYR-PPDYRDLISFTVYYKEAP--

FKNVTEYDGQDACGSNSWNMVDVDLP-----PNKDVEPGILLHG-LKPWTQYAVYVKAVT

LTMVENDHIRGAKSEILYIRTNASVPSIPLDVLSASNSSSQLIVKWNPPSLPNGNLSYYI

VRWQRQPQDGYLYRH-----NY-----------------CSKD-KIPIRKYADGTIDVEE

VTENPKTEVCGGEKGPCCACPKTEAEKQAEKEEAEYRKVFENFLHNSIFV--PRPERKRR

DVMQVANTTMS----------------------------SRSRNTTVADTYNVTDPEELE

TEYPFF------------------ESRVDNKERTVISNLRP-----FTLYRIDIHSCNHE

AE-KLGCSASNFVFARTMPA--EGADDIPGPVTWESRPENS--IFLKWPEPENPNGLILM

YEIKYG------SQTEDQR-----------------------------ECVSRQEY-RKY

GGAKLNRLNPGNYTARIQATSLSGNGSWTDPVFFYVPAKTTYENFIHLIIALP-------

---VAILL----IVGGLVIM----LYVFHRKRNNSRLGNGVLYASVNPEYFSAAD-----

VYVPDEWEVAREKIAMSRELGQGSFGMVYEGVAKGVVKDEPE--TRVAIKTVNEAASMRE

RIEFLNEASVMKEFNCHHVVRLLGVVSQGQPTLVIMELMTRGDLKSYLRSLRPEME-NNP

VL---APPSLSKMIQMAGEIADGMAYLNANKFVHRDLAARNCMVAEDFTVKIGDFGMTRD

IYETDYYRKGGKGLLPVRWMSPESLKDGVFTTHSDVWSFGVVLWEIATLAEQPYQGLSNE

QVLRFVMEG-GLLDKPDNCPDMLFELMRMCWQYNP-KMRPSFLEIISSIKD---------

------------------------------------------------------------

---------------------------E--MEPGFREV-----------------SFYYS

EENK-----PP----EPEELDL----------EPENMESVPLDPS---------------

---------------------------------------ASSSSLPLPDRHSGHKA----

------ENGPGPGVLV--------------------------------------------

----------------LRTSFDERQPYAHMNGGRTNERALPLP-------------QSST

C--------------------------------------------

>M brandtii

------------------------------------------------------------

------------------------------------------------------------

--------------------------------------------MDIRNNFTRLY-----

-----ELANCSIIEGHLQILLMFSSRPEDFRDLS--------------FPKLIMITDYLL

LFRVYGLESLKDLFPNLTVIRGSRLFFNYALVIFEMVHLKELGLYSLMNITRGSVRIEKN

NDLCYLATIDWSRI-LDSVEDNYIV-----LNKDDNEECGDVCPGTAKGKTSCPATVING

-----QFVERCWTHSHCQKVC------PTSCKSHGCTAEGLCCHPECLGSCSEPDDPTKC

VACRHFYLDGRCVETCPAPYYHFRDWRCVNFSFCLDLHNKCKNSRRQGCHQYVIHNNKCI

PECPSGYTMNSSNLM-CTPCLGPCPKVC-HVLEGQKIIDSVTAAQELRGCTVINGSLTIN

IRGG--NNLAAELEANLGLIEEISGYLKIRRSYALVSLSFFRKLRLIQGDT--LEVGNYS

FYALDNQNLRQLWDWSKH-NLTITRGKLFFHYNPKLCLSEIHKMEEISGTKGRQERTDIA

LKTNGDQASCENELLKFSYIRTSSDK--IMLKWE--PYW-PPDFRDLLGFMLFYKEAP--

YRNVTEFDGQDACGSNSWTVVDIDPPPRSTDPKSQAHPGWLMRS-LKPWTQYAIFVKTL-

VTFSDERRTYGAKSDIIYIQTNATNPSVPLDPISVSNSSSQIILKWKPPSDPNGNITHYL

VFWERQEEDSELYEL-----DY-----------------CLKGLKLPSRTWSPPS-ELEG

SQKHNQSE-YDEAAGECCSCPKTDSQILKELEESSFRKTFENYLHNVVFV--PRSSRKRR

ALGDEGNVT-T----------------------------AEPTALGFPNTSTIV-PTRSE

EPRPF--------------------EKVVNKESLVISGLRH-----FTGYRIELQACNQD

FP-EERCSVAAYISARTMPE--AKADDIVGPVTHEIFENNI--VHLMWQEPKHPNGLIVL

YEVSY-------RRYGDEE---------------------------LHLCVSRRHF-ALE

RGCRLRGLSPGNYSVRVRATSLAGNGSWTEATYFYVTNYLDVPSNIAKIIIGP-------

------LI--FVFLFSVVIG---SIYLFLRKRQSDG-PVGPLYASSNPEYLSASDVFPCS

VYVPDEWEVPREKITLLRELGQGSFGMVYEGNAKDIIKGEAE--TRVAVKTVNESASMRE

RIEFLNEASVMKGFACHHVVRLLGVVSKGQPTLVVMELMTHGDLKSHLRSLRPDAE-NNP

---GRPPPTLQEMFQMAAEIADGMAYLNAKKFVHRDLAARNCMVAHDFTVKIGDFGMTRD

IYETDYYRKGGKGLLPVRWMAPESLKDGVFTASSDMWSFGVVLWEITSLAEQPYQGLSNE

QVLKFVMDG-GYLDIPDNCPERLSELMSMCWHFNP-KMRPTFLEVVDLLKD---------

------------------------------------------------------------

---------------------------N--LHPSFPEV-----------------SFFYS

EENK-----AP----ENDELEM----------EFEDMESVPLDRS---------------

-------------------------------------------------SHCQRE-----

------EDGGCPLA----------------------------------------------

----------------LKRNYEDHMSYSHMNGGKKNGRVLTLP-------------RSSP

S--------------------------------------------

>S franciscanus

------------------------------------------------------------

---------------------------------------NE-------------LFV---

FIYLF---------------------------------TVC-QSMDIRNSAEKFS-----

-----KLENCTVIEGYLQIVLIDHATPSDYAGLS--------------FPKLREITEYFV

MFRVKNLQTLRHIFPNLAVIRGDSLFFNYALIIFEMFELQEIGLPSLQAVMRGSVRIEKN

INLCYLSTVDWSLVQFQGMENNFIK-----GNKDQ-EECFNFCPED-DGKRLCRKLTASG

-----DVAELCWTNEHCQKGK------PVSC-PRSCNAAGRCCHEQCIGGCSGS-TPSDC

TACRHFLHLGVCVGECPNGTFQVSQ----SFQSC-----------SKK---LFLGQIRIL

IKFLARIILPSRQCKLILSLSLFLLLVC-----DGKTIQSVEDAADMRGCTYVHGSLTIS

VHGR--GTVA-----------------------PWIGCKIYRSYEE-QGKK---------

---------------------YTGTGGEFAAEMPKRSPQNLDRVQ--CKTNQRLSMMCLF

LAS---HSVGNTHRLNLEF-NFFGTF--LMVNWS--PPKGKGDVREIVGYILSYREAP--

QRNVTELDGEDACGTSLWEVQYI--P-----PT---DTSTFVSN-LKPWTQYAFMVRTYT

IAGAQ----FSARYMIIYSLYPEAEASPPSSLKVISNSSSELIITWKPPEHPNGNLTHYI

ISWEPQ----YLPIHQFEDRDF-----------------CRES-CIPRTIIISSLIHIIA

P--------YYGQS--SCISDTQCIRLT----------------HNSLIM----------

NKTFLVHYP-------------------------------AHYDLTTTSFIQPI-PITPE

SEHNLNGSLYPSSPATGVNASTTPPSRVEERVIGLVESFRVTGLEHFTEYFITLQACN--

---SKDCSRAAAMYGRSLPK--ESADQIESAVMINHTSTDR--VHLTWAEPSNPNGIIVI

HEIRFEMMDVEVNDVGDERDAYTVVESRSTPKPVT-------QSTENTECVSAEGY-RRV

LGAELLGLKVGNYTAMVRAVSLAGEGPWTKPVMFSVPDITNSSYAKAALILCA-----KP

VRSCYLLKKPIAEILSYI-----HYPRHIKQS-HSHMKQ----ISISKYYIMIIY-----

VYVADEWEFPRDKLEIIRELGKGSFGMVYEGLAKGILPEEEEI-SRVAIKSVQANASMRD

RIEFLNEASYMKLIDAHNVVRLLGVVSKGQPTYVIMEFMAQGDLKNWLRARRPENQQDLP

-VELKNVYSVRALIGMIAQIASGMAYLSSMNFVHRDLAARNVLVGEGNVCKVADFGLARD

IYQSDYYRKESGGKLPIRWMAPEALLYGIFTIKSDVWSFGILLWEIVTYGKIPYPGLDNE

EVIEYIRQG-YRLPPPEGCPEEMYALMTKCWSYIP-AKRPPFKDISTRLKA---------

------------------------------------------------------------

---------------------------Y----PILKEV---------------ALCILLN

KLSG-----PIPQYGAASPMFL----ECSCRQSITQVISCPAHPQICR------------

-----------------------------------------PLASKASLIYHQI------

------TIKPCRDHTI--------------------------------------------

------------------TSYQSQILF-HPSMTMQQDQLCYLP-------------SIYI

P--------------------------------------------

>S purpuratus

------------------------------------------------------------

--------------------------------MMDGLARPARMGRTSVRSTTLLFFIIFI

VCSLFCISNVAG-------A---------------DADGVC-KSMDIRNSAEKFA-----

-----KLENCTVIEGYLQIVLIDHATPSDYAGLS--------------FPKLREITEYFV

MFRVKNLQTLRHIFPNLAVIRGDSLFFNYALIIFEMFELQEIGLPSLQAVMRGSVRIEKN

INLCYLSTVDWSLVQFQGMENNFIK-----GNKDQ-EECFNFCPED-DGKRLCRKLTSSG

-----DVAELCWTNEHCQKVC------PASC-PRSCNAAGRCCHEQCIGGCSGS-TASDC

TACRHFLHLGVCVGQCPNGTFQFKNRHCITGERC-----------PSG---WRLFNEKCT

EECPGGYMPDFNDSRHCLPCTGTCPKVC-----KGKTIQSVEDAADMRGCTYVHGTLTIS

VHGR--GNVLKELERNLGMIEVISDALIVRRANSLVSLNFLRNLRKIEGRDGGLEMDQYS

LYVLANNNLQQLFDFSQHPNITIVNGKFFFHYNPKLCYSEIEELHNLTGIKEPLSNVDVS

LSSNGDQVACNTHRLNLQF-NFFGTF--LMVNWS--PPKGKGDVREIVGYILSYREAP--

QRNVTELDGEDACGTSLWEVQYI--P-----PT---ETSTFVSN-LKPWTQYAFMVRTYT

IAGAQ----FSARSNIEYRLTPEAKASPPSSLKVISNSSSELIVTWKPPEHPNGNLTHYI

ITWEPQ----YLPIHQFDDRDF-----------------CREK--LPNLRKSESSTEMET

SEDEDTNTTMVGAG--CCMCPIDPDELARREEDARFQKDFENQLHNTIYK--RRPDTRRR

KKRDVPGSAIQ----------------------------DAGTTPTTTSFITPI-PTTPT

SGFNLNGSLYPSSPATGVNASTTSPSRVEERVIGLVESFHVTGLEHFTEYFITLQACN--

---SKDCSRAAAMYGRSLPK--ESADQIESAVMINHTSTDR--VHLTWAEPSNPNGIIVI

HEIRFEMMDVEVDDVGDERDAYTVVESHSTPKPVT-------QSTENTECVSAEGY-KRV

LGAELLGLKVGNYTAMVRAVSLAGEGPWTKPVMFSVPDITNNPTYEPDKALPP---MMGV

TIAVSIAAVII--IIVFIIIF---LRWHYRK---DQMPDGVLYASVNPEYMSTSD-----

MYVADEWEFPRDKLEIIRELGKGSFGMVYEGLAKGILPEEEEI-SRVAIKSVQANASMRD

RIEFLNEASVMKLIDAHNVVRLLGVVSKGQPTYVIMEFMAQGDLKNWLRARRPENQQDLP

LIDRKSVPTLEQLLNMAAEIADGMSFLAARKYVHRDLSARNCLVSGEGTCKVADFGLARD

IYQSDYYRKERGGMLPIRWMAPESVKDGVFQASSDVWSFGILLWEMATLGELPYQGLSNE

EAGEYIKGG-NVLRPPENCPEKMQEIMMACWQYQE-KLRPLFGEIILNLQD---------

------------------------------------------------------------

---------------------------YGLLRDSFAQN-----------------CFFLN

EQLN-----A-----SAAPADM----------SSKELETVSILDQ---------------

------------------------------------------NAKNNENLYTSMSNNK--

-------PEGASNGG---------------------------------------------

----------------NRAGGSVEVPIVTGPSSSPKDRNGSLTG------------NGSI

PHGVGVG-KCTEC--------------------------------

>L variegatus

------------------------------------------------------------

--------------------------------REDNLSINSLKLEVMLRG---FLFILNL

VLKLL-INIILFT----------------------LFVTVC-KSMDIRNSAEYFS-----

-----KLENCTVIEGYLQIVLIDHAKPSDYAGLS--------------FPKLREITEYFV

MFRVKNLQTLRHIFPNLAVIRGDSLFFNYALIIFEMFELQEIGLPSLQAVMRGWVRIEKN

INLCYLSTVDWSLVQVQGMENNFIK-----GNKDQ-EECFNFCPED-DGKRLCRKLTATG

-----DVAELCWTGQDCQKVC------PVSC-PRSCNEAGRCCHEQCIGGCRGS-TASDC

TACRHFLHLGVCVGQCPNRTFQVKNRHCITAEQC-----------PAG---WKLFNEKCT

QDCPGGYMPDFNDSRHCLPCTGTCPKVC-----DGKTIQSVEDAAAMKGCTYVHGSLTIS

VHGR--GTVVKELEQNLGMIEVVSDALIVRRANSLVSLNFLRNLRRIEGRVGGLEMSEYS

LYVLANNNLQQLFDFSQHPNISIGNGKFFFHYNPKLCYSEIEKLHNLTGIKAPLSNIDVS

LSSNGDQFAGNTHRLNLEF-AFFQSV--LSVRWS--PPKGKGDVREIVGYILSYREAP--

QRNVTELDGEDACGTSLWEVQYI--P-----PT---ETSTFVAN-LKPWTQYAFMVRTYT

IAGAQ----FSARSNIQYLLTPESEASPPSSLNVISNSSSELVITWKPPEHPNGNLTHYI

ITWEPR----YLFKQQFEDRDF-----------------CRQE--LPTLRKSESSTEMET

SEDEDTNTTMVGAG--CCTCPIDPEELRRREEDARFQKDFENQLHNNIYMAYATSSATRS

GASQYIHYAID----------------------------DIEVSKLQYELHTPL-SKQYK

SCYFIYGMILCYIRYAL-------PALIDQHIHSFISRY-------IAHYYRDSQRHHLP

YR-SWSASKIISICPMPIARIYESADQIESTVMINHTSTDR--VHLTWAAPTNPNGIVVI

HEVRFEMVDPKVDDVGDERDAYTVVESLSTPKPVT-------QSTENTECVSAEGY-RRV

LGAELLGLKVGNYTAMVRAVSLAGEGPWTKPVTFSVPDITNSCYIIAAAVAKSQFPSLSI

VQYCHIMH--YSYIYSII------CYIIYRK---DQMPDGVLYASVNPEYIIISP-----

VYVADEWEFPHDKLEIIKKLGKGSFGMVYEGLAKGILPDEEDI-SRVAIKSVQANASMRD

RIEFLNEASYSK-IEAHHVVRLLGVVSKGQPTYVIMEFMAQGDLKNWLRARRPENQQDLP

LIDRKNVPSLRQLIYMAAQIADGMAYLSSQRFVHRDLAARNILVGENYTCKIADFGLARD

IYQSDYYRKEGGAKFPIRWMAPESLLYGKFTIKSDVWSFGVLLWEIVTLGRIPYPGLSNA

EVPKYIKGG-NSLRPPENCSPQM-AILFQSWYYEKICLRSSALKNLARQGA---------

------------------------------------------------------------

---------------------------R----CIYAKE---------------KICFSKP

EIYH-----KI----HCSDFYK----------LPLDLELIPYICM---------------

--------------------------------------------SVLILIHCLQY-----

------DKTLCQKVSL--------------------------------------------

----------------FQSASACCLQGVWCVVGDHSKRSIAVP-------------SELK

CRGPQVR-GIWTC--------------------------------

>M musculus

------------------------------------------------------------

--------------------------------MKSGSGGGS---PTSLWG---LVFLS--

------AALSLWP----------------------TSGEICGPGIDIRNDYQQLK-----

-----RLENCTVIEGFLHILLI--SKAEDYRSYR--------------FPKLTVITEYLL

LFRVAGLESLGDLFPNLTVIRGWKLFYNYALVIFEMTNLKDIGLYNLRNITRGAIRIEKN

ADLCYLSTIDWSLI-LDAVSNNYIV-----GNKPP-KECGDLCPGTLEEKPMCEKTTINN

-----EYNYRCWTTNRCQKMC------PSVCGKRACTENNECCHPECLGSCHTPDDNTTC

VACRHYYYKGVCVPACPPGTYRFEGWRCVDRDFCANIPNAESSD-SDG---FVIHDDECM

QECPSGFIRNSTQSMYCIPCEGPCPKVCGDEEKKTKTIDSVTSAQMLQGCTILKGNLLIN

IRRG--NNIASELENFMGLIEVVTGYVKIRHSHALVSLSFLKNLRLILGEE--QLEGNYS

FYVLDNQNLQQLWDW-NHRNLTVRSGKMYFAFNPKLCVSEIYRMEEVTGTKGRQSKGDIN

TRNNGERASCESDVLRFTSTTTWKNR--IIITWH--RYR-PPDYRDLISFTVYYKEAP--

FKNVTEYDGQDACGSNSWNMVDVDLP-----PNKEGEPGILLHG-LKPWTQYAVYVKAVT

LTMVENDHIRGAKSEILYIRTNASVPSIPLDVLSASNSSSQLIVKWNPPTLPNGNLSYYI

VRWQRQPQDGYLYRH-----NY-----------------CSKD-KIPIRKYADGTIDVEE

VTENPKTEVCGGDKGPCCACPKTEAEKQAEKEEAEYRKVFENFLHNSIFV--PRPERRRR

DVMQVANTTMS----------------------------SRSRNTTVADTYNITDPEEFE

TEYPFF------------------ESRVDNKERTVISNLRP-----FTLYRIDIHSCNHE

AE-KLGCSASNFVFARTMPA--EGADDIPGPVTWEPRPENS--IFLKWPEPENPNGLILM

YEIKYG------SQVEDQR-----------------------------ECVSRQEY-RKY

GGAKLNRLNPGNYTARIQATSLSGNGSWTDPVFFYVPAKTTYENFMHLIIALP-------

---VAILL----IVGGLVIM----LYVFHRKRNNSRLGNGVLYASVNPEYFSAAD-----

VYVPDEWEVAREKITMNRELGQGSFGMVYEGVAKGVVKDEPE--TRVAIKTVNEAASMRE

RIEFLNEASVMKEFNCHHVVRLLGVVSQGQPTLVIMELMTRGDLKSYLRSLRPEVEQNNL

VL---IPPSLSKMIQMAGEIADGMAYLNANKFVHRDLAARNCMVAEDFTVKIGDFGMTRD

IYETDYYRKGGKGLLPVRWMSPESLKDGVFTTHSDVWSFGVVLWEIATLAEQPYQGLSNE

QVLRFVMEG-GLLDKPDNCPDMLFELMRMCWQYNP-KMRPSFLEIIGSIKD---------

------------------------------------------------------------

---------------------------E--MEPSFQEV-----------------SFYYS

EENK-----PP----EPEELEM----------EPENMESVPLDPS---------------

---------------------------------------ASSASLPLPERHSGHKA----

------ENGPGPGVLV--------------------------------------------

----------------LRASFDERQPYAHMNGGRANERALPLP-------------QSST

C--------------------------------------------

>C elegans

MTRMNIVRCRRRHKILENLEEENLGPSCSSTTSTTAATEALGTTTEDMRLKQQRSSSRAT

EHDIVDGNHHDDEHITMRRLRLVKNSRTRRRTTPDSSMDCYEENPPSQKTSINYSWISKK

SSMTSLMLLLLFAFVQPCAS---------IVEKRCGPIDIRNRPWDIKPQWSKLGDPNEK

DLAGQRMVNCTVVEGSLTISFVLKHKTKAQEEMHRSLQPRYSQDEFITFPHLREITGTLL

VFETEGLVDLRKIFPNLRVIGGRSLIQHYALIIYRNPDL-EIGLDKLSVIRNGGVRIIDN

RKLCYTKTIDWKHLITSSINDVVVDNAAEYAVTETGLMCPRGACEEDKGESKCHYLEEKN

QEQGVERVQSCWSNTTCQKSCAYDRLLPTKEIGPGCDANGDRCHDQCVGGCERVNDATAC

HACKNVYHKGKCIEKCDAHLYLLLQRRCVTREQCLQLNPVLSN----KTVPIKATAGLCS

DKCPDGYQINPDDHRECRKCVGKCEIVCEIN----HVIDTFPKAQAIRLCNIIDGNLTIE

IRGKQDSGMASELKDIFANIHTITGYLLVRQSSPFISLNMFRNLRRIEAKS--LFRNLYA

ITVFENPNLKKLFDSTTD--LTLDRGTVSIANNKMLCFKYIKQLMSKLNIP--LDPIDQS

EGTNGEKAICEDMAINVSITAVNADS--VFFSWPSFNIT-DIDQRKFLGYELFFKEVPRI

DENMTIEEDRSACVDSWQSVFKQYYETSNGEPTPDIFMDIGPRERIRPNTLYAYYVAT--

-QMVLHAGAKNGVSKIGFVRTSYYTPDPP-TLALAQVDSDAIHITWEAPLQPNGDLTHYT

IMWR-ENEVSPYEEAEKFCTDASTPANRQHTKDPKETIVADKPVDIPSSRTVAPTLLTMM

GHEDQQKTCAATPGCCSCSAIEESSEQNKKKRPDPMSAIESSAFENKLLDEVLMPRDTMR

VRRSIEDANRVSEELEKAENLGKAPKTLGGKKPLIHISKKKPSSSSTTSTPAPTIASMYA

LTRKPTTVPGTRIRLYEIYEPLPGSWAINVSALALDNSYVIRNLKHYTLYAISLSACQNM

TVPGASCSISHRAGALKRTKHITDIDKVLNETIEWRFMNNSQQVNVTWDPPTEVNGGIFG

YVVKLK------SKVDG-------------------------SIVMTRCVGAKRGYSTRN

QGVLFQNLADGRYFVSVTATSVHGAG--PEAESSDPIVVMTPGFFTVEIILG--------

---MLLVFLILMSIAGCIIYYY------IQVRYGKKVKALSDFMQLNPEYCVDNK-----

-YNADDWELRQDDVVLGQQCGEGSFGKVYLGTGNNVVSLMGDRFGPCAIKINVDDPASTE

NLNYLMEANIMKNFKTNFIVKLYGVISTVQPAMVVMEMMDLGNLRDYLRSKREDEVFNET

DCNFFDIIPRDKFHEWAAQICDGMAYLESLKFCHRDLAARNCMINRDETVKIGDFGMARD

LFYHDYYKPSGKRMMPVRWMSPESLKDGKFDSKSDVWSFGVVLYEMVTLGAQPYIGLSND

EVLNYIGMARKVIKKPECCENYWYKVMKMCWRYSP-RDRPTFLQLVHLLAAEASPEFRDL

SFVLTDN-----------------------------------------------------

-----------QMILDDSEALDLDDIDDTDMNDQVVEVAPDVEN----------VEVQSD

SERRNTDSIPLKQFKTIPPINATTS------HSTISIDETPMKAKQ----------REGS

LDEEYALMNHSGGPSDAEVRTYAGDGDYVERDVRENDVPTRRNTGASTSSYTGGGPYCLT

NRGGSNERGAGFGEAVRLTDGVGSGHLNDDDYVEKEISSMDTRRSTGASSSSYGVPQTNW

SGNRGATYYTSKAQQAATAAAAAAAALQQQQNGGRGDRLTQLPGTGHLQSTRGGQDGDYI

ETEPKNYRNNGSPSRNGNSRDIFNGRSAFGENEHLIEDNEHHPLV

INDY

>H sapiens

--------------------------MAALAAAAKKVWSARRLLVLLFTPLALLPVVFAL

PPKEGRCLFVILLMAVYWCTEALPLSVTALLPIVLFPFMGILPSNKVCPQYFLDTNFLFL

SGLIMASAIEEWNLHRRIALKILMLVGVQPARLILGMMVTTSFLSMWLSNTASTAMMLPI

ANAILKSLFGQKEVRKDPSQESEE----------------------NTAAVRRNGLHTVP

TEMQFLASTEAKDHPGETEVPLDLPADSRKEDEYRRNIWKGFLISIPYSASIGGTATLTG

TAPNLILLGQLKSFF--PQCDVVNFGSWFIFAFPLMLLFLLAGWLWISFLYGGLSFRG-W

RKNKSEIRTNAEDRARAVIREEYQNLGPIKFAEQAVFILFCMFAILLFTRDPKFIPGWAS

LFNPG-----FLSDAVTGVAIVTILFFFPSQRPSLK-----WWFDFK--APNTETEP--L

LTWKKAQETVPWNIILLLGGGFAMAKGCEES-GLSVW-IGGQLHPLENVPPALAVLLITV

VIAFF-TEFASNTATIIIFLPVLAELAIRLRVHPL-------------------------

------------------------------------------------------------

---------------YLMIPGTVGCSFAFMLPVSTPPNSIAFASGHLLVKDMVRTGLLMN

LMGVLLLSLAMNTWAQTIF-QLGTFPDWADMYSVNVTALPPTLANDTFRTL

>H_glaber

-----------------------------MTTCWQGLWAYRSYLIVLLLPIILLPLPILI

PTKEAYCAYAIILMALFWCTEALPLAVTAFFPLFLFPLMGIMEASEVSTEYLKDTNILFI

GGLIVAIAVEHWNLHKRIALRALLIIGVRPALLILGFMMVTAFLSMWISNTATTAMMVPI

AHAVLEELQ---NTRMDSQGGSD-----------------------NPTFELQD--PSPQ

KEVTKLENGQITPA-------HSEPRAMKSKEEVR--FSKGMSLCVAYSASIGGIATLTG

TTPNLVMQGQMQSIF-PENPNIINFASWFGFAFPTMVILLLLAWLWLQFLFLGFNFKKNF

GIGDQGKEKK--QAALQVIKIQHKLLGPMNFAEKAVTVLFVILVVLWFTREPGFFPGWGN

LAFANSKGESMPSDGTVAMFISVILFIVPSKIPGL-------TQDPE--NPGKLQAPPAL

LTWKTVNEKMPWNIMLLLGAGFALAKGSEKS-GLSEW-VGDKLSPLQHMPPPATALIICL

LIATF-TECISNVATTTLFLPILASMAQAICLHPL-------------------------

------------------------------------------------------------

---------------YIMLPCTLASSLAFMLPVATPPNAIVFSFGGLKVTDMARAGFLLN

IIGVLVIMLAINTWSFHIF-NMHTFPSWAQSNATQCFPSQPDHTTASP---

>M_brandtii

-----------------------------MATCWQGLWAYRFYLFVYLLPIILLPLPLLI

PTKQAYCAYAIILMALFWCVEALPLAVTALFPLILFPMMGIMDAAEVCIEYFNDTNILFI

GGLLVAIAVEHWNLHKRIALRVLLIIGVRPALLMLGFMMVTAFLSMWISNTATTAMMVPI

AQAVLEQLHKA-PEDKDVEKGSD-----------------------NPTFELQE--TSPQ

KEVTTLDNGQVHPV----PPGPLEPKAQRTQEQLR--FSQGMSLCVCYSASIGGIATLTG

TTPNLVLQGQINSIF-PDNGNVVNFASWFGFAFPAMIILLVFAWVWLQLLFLGFNFRKNF

GMGEEARGQQ--QAAVRVIQTEHRRLGPMTFAEMAVTFLFVLLVLLWFTREPGFFPGWGT

LAFSNSKGESLVSDGTVAIFISIIMFIVPSEIPGL-------TQNPE--KPGKLKAPPAL

LNWKTVNEKMPWNIVFLLGGGFALAKGSEES-RLSAW-LGDKLTPLQSVSPPAIAFILSI

LVATF-TECTSNVATTTLFLPILASMAKAICLHPLYVMLPCTLAASLAFMLPVATPPNAI

VFSFGGIKVSDMESRLSAWLGDKLTPLQSVSPPAIAFILSILVATFTECTSNVATTTLFL

PILASMAKAICLHPLYVMLPCTLAASLAFMLPVATPPNAIVFSFGGIKVSDMVRAGFLLN

ILGVLVIMLAINTWSTPIF-SLNTFPPWAFANATHCMANATA---PGP---

>S franciscanus

---------------------------KQCLVVWVYSYQHLEFVQLNEHKNNYALICPKY

SKKIGYMAFTIIIMATYWVTEVIPIAATALIPMVLLPLFGIQDSGDVIQQYLKSTTFLFF

GGLVLAAAIEHQNLHRRFALRVLLLFGTKPKWLMFGFMSATAFLSMWMSNTATTAMMIPI

AQAVLDELK---SQRIDEEMGGDVDKNDGNGSPLYKVT---TSSC-QGYIAICQFIGG-H

QSIVEEPGSLPVEIKN---APLLIEEESPAERGYR-LLCKGITLSIPYAANIGGTATLTG

TGPSIAISTQFCSLYGPEAG--LTFASWMMYAIPGMIICLLICWIWLQIIYIDF-PCLQR

SDKRVGIAQE--ERAKMVIRRSYTALGKMSFAEWAILGHFVILISLWLTRDPKFVSGW-S

VGFLT--G--YVTDATSVIFISVLLFSFPSRPPSFLCRRKQDDDGSEDKKPAKI---PAL

LTWPVVHEKMAWSVIILLGGGSALADGCKVNYHVCAYHIAHRCAPQIYLQYGLIPYLKPK

YMYYYYLLISYIVKSFTYFY-----CAVATRINPL-------------------------

------------------------------------------------------------

---------------YLMLPATISASFAFMLPVATPPNAIAFASKELSVIDMVGSGVVMN

IACILVSNLSLNTLGVWVF-DVKTFPDWANRKAR-----------------

>S purpuratus

-------------------------MGKERGLEFQEVLKNWQYLVIVLVPIIFGWVPIAF

NNSIGHMAYCIIIMALYWMTECLPLAATALLPILLFPLFGIQKSSDVCSNYLTDTNTVFF

GGLMVAAAVEHWNLHKRIALRVLMLVGAKPRWLMFGFMSTTAFLSMWMSNTATTAMMIPI

SQAVLDELK---PSENDEETGGDVDKNDENGSPMHKGSEEKEEHCSENPIELDEIAAG--

-HIVEEPGSFPVEIKN---APLLIEEESKADRGHR-LLCKGITLSIPYAANIGGTATLTG

TPPNLVISAQVKKLFGPEVG--VNFGTWMMYAFPGMVICLLICWVWLQIMYLDF-FCLQR

SDKRVGLAQE--ERAKMVIRRSYAALGKISFAEWAILGHFVVLVILWLTRDPKFVNGW-S

VNFLK--G--YVTDATSVIFISLLLFAFPSRLPSFFCRKKQDDDGSEDKKPAKI---PTL

LTWPVIHEKMAWSVIILLGGGSALADGCKVS-GLSAW-LGGQFKEFSALSPAALAIIIST

VVATF-TEVTSNTATATIFLPIFAELSISIGLNPL-------------------------

------------------------------------------------------------

---------------YLMLPAVIACSFAFMLPVATPPNAIAFAHGNLTVLDMAKAGIVMN

IACIIVSNLSLNTLGVWVF-DVKTFPAWANRTVT-----------------

>D melanogaster

MATETTKMIYTPPPLDIKMEIEIGEQPQPPVKCSNFFANHWKGLVVFLVPLLCLPVMLLN

EGAEFRCMYLLLVMAIFWVTEALPLYVTSMIPIVAFPIMGIMSSDQTCRLYFKDTLVMFM

GGIMVALAVEYCNLHKRLALRVIQIVGCSPRRLHFGLIMVTMFLSMWISNAACTAMMCPI

IQAVLEELQ---AQ------GVC-----------------------KINHEPQYQIVGGN

KKNNEDEPPYPT------------------------KITLCYYLGIAYASSLGGCGTIIG

TATNLTFKGIYEARF-KNSTEQMDFPTFMFYSVPSMLVYTLLTFVFLQWHFMGLWRPKSK

EAQEVQRGREGADVAKKVIDQRYKDLGPMSIHEIQVMILFIFMVVMYFTRKPGIFLGW--

ADLLNSKD---IRNSMPTIFVVVMCFMLPANYAFLRYCTRRGGPVPT--GPT-----PSL

ITWKFIQTKVPWGLVFLLGGGFALAEGSKQS-GMAKL-IGNALIGLKVL-PNSVLLLVVI

LVAVFLTAFSSNVAIANIIIPVLAEMSLAIEIHPL-------------------------

------------------------------------------------------------

---------------YLILPAGLACSMAFHLPVSTPPNALVAGYANIRTKDMAIAGIGPT

IITIITLFVFCQTWGLVVYPNLNSFPEWAQIYAAAALGNKTH---------

>L variegatus

-------------------------EIRNMGFAFQEALRNWHYLVIVLVPIIFCPVPIVF

NNSIGYMAYCIIIMALYWMTECIPLAATALLPILLFPLFGIQKSKDVCSNYLTDTNTVFF

GGLMLAAAVEHWNLHKRIALRVLMLVGAKSRWLMLGFMSGTAFLSSWMSNTATTAMMIPI

AQAVLNELK---SQKHDGEMGNDVGKVEENSNIITAFA-----YC-KSILLVDYFYVTLY

IRIIKVLDSVPRCMST---KENLSTRENRSERRHR-RLCKAITLSIPYAAGIGGIASLTA

TPANVVCVAQVERLFGSEAG--LNFGTWMMYAFPGMIVCLLICWIWLQIMYLDFLLINKI

MINNIIQGDS--------VCYGHLELTHIVFAEWAILGHFVVLVALWLTRDPKFVSGW-S

VGFLN--G--YVTDATSVIFISLLLFAFPSRLPTFLCRRNRGDDDPEDKKPAQI---PAL

LTWPVVHEKMAWNVIILLGGGFALADGCKVS-GLSAW-LGAEFSVFSSLSPSAMAIIIST

IVAIF-TEISSNTATATIFLPIFAELAVAMRINPL-------------------------

------------------------------------------------------------

---------------YLMLTTTICASFAFMLPVATPPNAIAFAYKEIKVIDMVRAGIVMK

IACILVSSLSINTLGVWIF-DVNTFPAWANVTAA-----------------

>M musculus

-----------------------------MATCWQALWAYRSYLIVLCLPIFLLPLPLIV

QTKEAYCAYSIILMALLWCTEALPLAVTALFPIILFPLMGIMEASKVCLEYFKDTNILFV

GGLMVAIAVEHWNLHKRIALGVLLIIGVRPALLLLGFMLVTAFLSMWISNTATTAMMLPI

GYAVLEQLQ---GSQKDVEEGNS-----------------------NPSFELQE--ASPQ

KEETKLDNGQAVSV-------SSEPRAQKTKEHHR--FSQGLSLCICYSASIGGIATLTG

TTPNLVLQGQVNSIF-PENSNVVNFASWFGFAFPTMVILLLLAWLWLQVLFLGVNFRKNF

GFGEGEEERK--QAAFQVIKTQHRLLGPMSFAEKAVTFLFVLLVVLWFTREPGFFPGWGD

TAFANKKGQSMVSDGTVAIFISLIMFIIPSKIPGL-------TEDPK--KPGKLKAPPAI

LTWKTVNDKMPWNILILLGGGFALAKGSEES-GLSKW-LGDKLTPLQHVPPSATVLILSL

LVAIF-TECTSNVATTTLFLPILASMAQAICLHPL-------------------------

------------------------------------------------------------

---------------YVMLPCTLAASLAFMLPVATPPNAIVFSFGGLKVSDMARAGFLLN

IIGVLTITLSINSWSIPIF-KLDTFPTWAYSNTSQCLLNPPNSTVPGH---

>SIG

------------------------------------------------------------

+-----------------------------------------------------------

------------------------------------------------------------

------------~--------+--------------------------------------

------------------------------------------------------------

------------------------------------------------------------

------------------------------------------------------------

------------------------------------------------------------

------------------------------------------------------------

------------------------------------------------------------

------------------------------------------------------------

------------------------------------------------------------

INSR

>H sapiens

------------------------------------------------------------

------------------------------------------MATGGRRGAAAAPLLVAV

AALLLGAAGHLY------------------PGEVCPGMDIRN-----------------N

LTRLHELENCSVIEGHLQILLMFKTRPEDFRDLS--------------FPKLIMITDYLL

LFRVYGLESLKDLFPNLTVIRGSRLFFNYALVIFEMVHLKELGLYNLMNITRGSVRIEKN

NELCYLATIDWSRI-LDSVEDNYIV-LNKDDNEECGDICPG----TAKGKTNCPATVING

-----QFVERCWTHSHCQKVC------PTICKSHGCTAEGLCCHSECLGNCSQPDDPTKC

VACRNFYLDGRCVETCPPPYYHFQDWRCVNFSFCQDLHHKCKNSRRQGCHQYVIHNNKCI

PECPSGYTMNSSNL-LCTPCLGPCPKVCHLLEGEKTIDSVTSAQELRGCTVINGSLIINI

RGG--NNLAAELEANLGLIEEISGYLKIRRSYALVSLSFFRKLRLIRGET--LEIGNYSF

YALDNQNLRQLWDWSKH-NLTITQGKLFFHYNPKLCLSEIHKMEEVSGTKGRQERNDIAL

KTNGDQASCENEL--LKFSYIRTSFDKILLRWEPYWPP---DFRDLLGFMLFYKEAPYQN

VTEFDGQD-ACGSNSWTVVDIDPPLRSNDPKSQ--NHPGWLMRGLKPWTQYAIFVKTLVT

FSDERRTYGAKSDIIYVQTDATNPSVPLDPISVSNSSSQIILKWKPPSDPNGNITHYLVF

WERQAEDSELFE-LDYCLKGLKLPSRTWS---------------------PPFESEDSQK

HNQSEYEDSAGECCSCPKTDSQILKELE------ESSFRKTFEDYLHNVVFV--PRKTSS

GTGAEDPRPSRKRRSLGDVGNVTVAVPTVAA----------------FPNTSSTSVPTSP

EEHR--------------------PFEKVVNK--ESLVISGLRHFTGYRIELQACNQDTP

E-ERCSVAAYVSARTMPE--AKADDIVGPVTHEIFENN--VVHLMWQEPKEPNGLIVLYE

VSYRRY-------GDE--------------------ELHLCVSRKH--FALERGCRLRGL

SPGNYSVRIRATSLAGNGSWTEPTYFYVTDYLDVPSNIAKIIIG---PLIFVFLFSVVIG

SIYLF-------LRKRQPDGPLGPLYASSNPEYLSASDVFPCSVYVPDEWEVSREKITLL

RELGQGSFGMVYEGNARDIIKGEAE--TRVAVKTVNESASLRERIEFLNEASVMKGFTCH

HVVRLLGVVSKGQPTLVVMELMAHGDLKSYLRSLRPEAENN-PG---RPPPTLQEMIQMA

AEIADGMAYLNAKKFVHRDLAARNCMVAHDFTVKIGDFGMTRDIYETDYYRKGGKGLLPV

RWMAPESLKDGVFTTSSDMWSFGVVLWEITSLAEQPYQGLSNEQVLKFVMDG-GYLDQPD

NCPERVTDLMRMCWQFNP-KMRPTFLEIVNLLKDDLHPS------FPEV-----------

--------SFFHSEEN---KAPESEELEMEFEDMENV--PLDRSSHCQREEAGGRDGGSS

LGFKRS-----YEEH-------------IPYTHMNGGKKNGRILTLP-------------

------RSNPS-------------------------------------------------

------------------------------------------------------------

------------------------------------------------------------

-----------

>H glaber

------------------------------------------------------------

------------------------------------------MGAWGARGAATAPLLVAA

AALLVGVTGHLY------------------PGEVCPGMDIRN-----------------N

LTRLHRLENCSIIEGHLQILLMFKTKPEDFRDLS--------------FPKLVMITDYLL

LFRVYGLESLKDLFPNLTVIRGSRLFFNYALVIFEMVHLKELGLYSLMNITRGSVRIEKN

NELCYLATIDWSRI-LDSVEDNYIV-LNKDDNEECGDICPG----TTKGKTNCPATVING

-----QFIERCWTHSHCQKVC------PTMCKSHGCTTEGLCCHSECLGNCSEPDDPTKC

VACRNFYLDGRCVASCPPPYYHFQDWRCVNFSFCQELHNKCKNSRRQGCHQYVIHNDRCI

PECPSGYTMNSSNL-MCTPCLGPCPKVCHLLEGEKTIDSVTSAQELRGCTVINGSLIINI

RGG--NNLAAELEANLGLIEEISGYLKIRRSYALVSLSFFRKLRLIRGET--LEIGNYSF

YALDNQNLRQLWDWSKH-NLTITQGKLFFHYNPKLCLSEIHKMEEVSGTKGRQERNDIAL

KTNGDQASCENEL--LKFSSIRTSSDKILLRWEPYWPP---DFRDLLGFMLFYKEAPYQN

VTEFDGQD-ACGSNSWTVVDIDPPLRSNDPKLQ--NHPGWLMRGLKPWTQYAIFVKTLVT

FSDERRTYGAKSDILYVQTDATNPSVPLDPISVSNSSSQIILKWKPPSDPNGNITHYLVY

WQRQEEDSELYE-LDYCLKGLKLPSRAWS---------------------PPFESEDAQK

HNQSEYEESAGECCSCPKTDSQILKELE------ESSFRKTFEDYLHNVVFV--PRNPSA

GSGAQDPRPSRKRRSLGE-VNVTTAVPLVPG----------------FPTLSSVSVPTNA

EEHR--------------------PFEKVVNK--ESLVISGLRHFTGYRIELQACNQDAP

E-ERCSVAAYVSARTMPE--AKADDIVGPVTHEIFDNN--AVHLMWQEPKEPNGLIVLYE

VSYRRY-------GEE--------------------ELHLCVSRKH--YALEKGCRLRGL

QPGNYSVRIRATSLAGNGSWTEATYFYVTNYSDVSSNIAKMIIG---PLIFVFLFSIVIG

SIYLF-------LRKRQPDGPMGPLYASSNPEYLSASDVFPCSVYVPDEWEVPREKITLL

RELGQGSFGMVYEGNAKDIVKGEAE--TRVAVKTVNEAASLRERIEFLNEASVMKGFTCH

HVVRLLGVVSKGQPTLVVMELMAHGDLKSYLRSLRPESENN-PG---RPPPTLQEMIQMA

AEIADGMAYLNAKKFVHRDLAARNCMVAHDFTVKIGDFGMTRDIYETDYYRKGGKGLLPV

RWMAPESLKDGVFTTSSDMWSFGVVLWEITSLAEQPYQGLSNEQVLKFVMDG-GYLDQPD

NCPERVTDLMRMCWQFNP-KMRPTFLEIVSLLKDDLHPS------FPEV-----------

--------SFFHSEEN---KAPESEELEMEFEDMENV--PLDRSSHCQREEAGGREGGSS

LGIKRN-----YEEH-------------IPYTHMNGGKKNGRILPLP-------------

------RSSPS-------------------------------------------------

------------------------------------------------------------

------------------------------------------------------------

-----------

>M brandtii

------------------------------------------------------------

------------------------------------------------------------

-------------------------------------MDIRN-----------------N

FTRLYELANCSIIEGHLQILLMFSSRPEDFRDLS--------------FPKLIMITDYLL

LFRVYGLESLKDLFPNLTVIRGSRLFFNYALVIFEMVHLKELGLYSLMNITRGSVRIEKN

NDLCYLATIDWSRI-LDSVEDNYIV-LNKDDNEECGDVCPG----TAKGKTSCPATVING

-----QFVERCWTHSHCQKVC------PTSCKSHGCTAEGLCCHPECLGSCSEPDDPTKC

VACRHFYLDGRCVETCPAPYYHFRDWRCVNFSFCLDLHNKCKNSRRQGCHQYVIHNNKCI

PECPSGYTMNSSNL-MCTPCLGPCPKVCHVLEGQKIIDSVTAAQELRGCTVINGSLTINI

RGG--NNLAAELEANLGLIEEISGYLKIRRSYALVSLSFFRKLRLIQGDT--LEVGNYSF

YALDNQNLRQLWDWSKH-NLTITRGKLFFHYNPKLCLSEIHKMEEISGTKGRQERTDIAL

KTNGDQASCENEL--LKFSYIRTSSDKIMLKWEPYWPP---DFRDLLGFMLFYKEAPYRN

VTEFDGQD-ACGSNSWTVVDIDPPPRSTDPKSQ--AHPGWLMRSLKPWTQYAIFVKTLVT

FSDERRTYGAKSDIIYIQTNATNPSVPLDPISVSNSSSQIILKWKPPSDPNGNITHYLVF

WERQEEDSELYE-LDYCLKGLKLPSRTWS---------------------PPSELEGSQK

HNQSEYDEAAGECCSCPKTDSQILKELE------ESSFRKTFENYLHNVVFV--PRS---

---------SRKRRALGDEGNVTTAEPTALG----------------FPN-TSTIVPTRS

EEPR--------------------PFEKVVNK--ESLVISGLRHFTGYRIELQACNQDFP

E-ERCSVAAYISARTMPE--AKADDIVGPVTHEIFENN--IVHLMWQEPKHPNGLIVLYE

VSYRRY-------GDE--------------------ELHLCVSRRH--FALERGCRLRGL

SPGNYSVRVRATSLAGNGSWTEATYFYVTNYLDVPSNIAKIIIG---PLIFVFLFSVVIG

SIYLF-------LRKRQSDGPVGPLYASSNPEYLSASDVFPCSVYVPDEWEVPREKITLL

RELGQGSFGMVYEGNAKDIIKGEAE--TRVAVKTVNESASMRERIEFLNEASVMKGFACH

HVVRLLGVVSKGQPTLVVMELMTHGDLKSHLRSLRPDAENN-PG---RPPPTLQEMFQMA

AEIADGMAYLNAKKFVHRDLAARNCMVAHDFTVKIGDFGMTRDIYETDYYRKGGKGLLPV

RWMAPESLKDGVFTASSDMWSFGVVLWEITSLAEQPYQGLSNEQVLKFVMDG-GYLDIPD

NCPERLSELMSMCWHFNP-KMRPTFLEVVDLLKDNLHPS------FPEV-----------

--------SFFYSEEN---KAPENDELEMEFEDMESV--PLDRSSHCQREE----DGGCP

LALKRN-----YEDH-------------MSYSHMNGGKKNGRVLTLP-------------

------RSSPS-------------------------------------------------

------------------------------------------------------------

------------------------------------------------------------

-----------

>S franciscanus

------------------------------------------------------------

------------------------------------------------------------

--NELFVFIYLF--------------------TVCQSMDIRN-----------------S

AEKFSKLENCTVIEGYLQIVLIDHATPSDYAGLS--------------FPKLREITEYFV

MFRVKNLQTLRHIFPNLAVIRGDSLFFNYALIIFEMFELQEIGLPSLQAVMRGSVRIEKN

INLCYLSTVDWSLVQFQGMENNFIK-GNKDQ-EECFNFCP-----EDDGKRLCRKLTASG

-----DVAELCWTNEHCQKGK------PVSC-PRSCNAAGRCCHEQCIGGCSGS-TPSDC

TACRHFLHLGVCVGECPNGTFQVSQ----SFQSCSK--------------KLFLGQIRIL

IKFLARIILPSRQCKLILSLSLFLLLVC----DGKTIQSVEDAADMRGCTYVHGSLTISV

HGR--GTVA-----------------------PWIGCKIYRS------------------

-------------YEEQGKKYTGTGGEFAAEMPKRSPQNLDRVQCKTNQR--LSMMCLFL

ASHS---VGNTHRLNLEFNFFGTF---LMVNWSP--PKGKGDVREIVGYILSYREAPQRN

VTELDGED-ACGTSLWEVQYIPPTDTST------------FVSNLKPWTQYAFMVR---T

YTIAGAQFSARYMIIYSLYPEAEASPPSSLKVISNSSSELIITWKPPEHPNGNLTHYIIS

WEPQYLPIHQFEDRDFCRESCIPRTIIIS---------------------SLIHIIAP--

------YYGQS-SCIS--DTQCIRLTHN------SLIMNKTFLVHYPAHYDL--------

-----------------TTTSFIQPIPITPESEHN-------LNGSLYPSSPATGVNAST

TPPS--------------------RVEERVIGLVESFRVTGLEHFTEYFITLQACNSKD-

----CSRAAAMYGRSLPK--ESADQIESAVMINHTSTD--RVHLTWAEPSNPNGIIVIHE

IRFEMMDVEVNDVGDERDAYTVVESRSTPKPVTQSTENTECVSAEG--YRRVLGAELLGL

KVGNYTAMVRAVSLAGEGPWTKPVMFSVPDITNSSYAKAALILCAK-PVRSCYLLKKPIA

EILSY-----IHYPRHIKQSHSHMKQISISKYYIMIIY-----VYVADEWEFPRDKLEII

RELGKGSFGMVYEGLAKGILPEEEEI-SRVAIKSVQANASMRDRIEFLNEASYMKLIDAH

NVVRLLGVVSKGQPTYVIMEFMAQGDLKNWLRARRPENQQDLPV-ELKNVYSVRALIGMI

AQIASGMAYLSSMNFVHRDLAARNVLVGEGNVCKVADFGLARDIYQSDYYRKESGGKLPI

RWMAPEALLYGIFTIKSDVWSFGILLWEIVTYGKIPYPGLDNEEVIEYIRQG-YRLPPPE

GCPEEMYALMTKCWSYIP-AKRPPFKDISTRLKAYPILKEVALCILLNK-----------

---------LSGPIPQYGAASPMFLECSCRQSITQVISCPAHPQICRPLASK--ASLIYH

QITIKP-----CRDHT------------ITSYQSQILFHPSMTMQQDQL-----------

-CYL-----PSIYIP---------------------------------------------

------------------------------------------------------------

------------------------------------------------------------

-----------

>S purpuratus

------------------------------------------------------------

------------------------------------MMDGLARPARMGRTSVRSTTLL--

-FFIIFIVCSLFCISNVAG---------ADADGVCKSMDIRN-----------------S

AEKFAKLENCTVIEGYLQIVLIDHATPSDYAGLS--------------FPKLREITEYFV

MFRVKNLQTLRHIFPNLAVIRGDSLFFNYALIIFEMFELQEIGLPSLQAVMRGSVRIEKN

INLCYLSTVDWSLVQFQGMENNFIK-GNKDQ-EECFNFCP-----EDDGKRLCRKLTSSG

-----DVAELCWTNEHCQKVC------PASC-PRSCNAAGRCCHEQCIGGCSGS-TASDC

TACRHFLHLGVCVGQCPNGTFQFKNRHCITGERCPS--------------GWRLFNEKCT

EECPGGYMPDFNDSRHCLPCTGTCPKVC----KGKTIQSVEDAADMRGCTYVHGTLTISV

HGR--GNVLKELERNLGMIEVISDALIVRRANSLVSLNFLRNLRKIEGRDGGLEMDQYSL

YVLANNNLQQLFDFSQHPNITIVNGKFFFHYNPKLCYSEIEELHNLTGIKEPLSNVDVSL

SSNGDQVACNTHRLNLQFNFFGTF---LMVNWSP--PKGKGDVREIVGYILSYREAPQRN

VTELDGED-ACGTSLWEVQYIPPTETST------------FVSNLKPWTQYAFMVR---T

YTIAGAQFSARSNIEYRLTPEAKASPPSSLKVISNSSSELIVTWKPPEHPNGNLTHYIIT

WEPQYLPIHQFDDRDFCREKL-PNLRKSE---------------------SSTEMETSED

EDTNTTMVGAG-CCMCPIDPDELARREE------DARFQKDFENQLHNTIYKRRPDTRRR

KKRDVPGSAIQDAGTTPTTTSFITPIPTTPTSGFN-------LNGSLYPSSPATGVNAST

TSPS--------------------RVEERVIGLVESFHVTGLEHFTEYFITLQACNSKD-

----CSRAAAMYGRSLPK--ESADQIESAVMINHTSTD--RVHLTWAEPSNPNGIIVIHE

IRFEMMDVEVDDVGDERDAYTVVESHSTPKPVTQSTENTECVSAEG--YKRVLGAELLGL

KVGNYTAMVRAVSLAGEGPWTKPVMFSVPDITNNPTYEPDKALPPMMGVTIAVSIAAVII

IIVFIIIFLRWHYRK--DQMPDGVLYASVNPEYMSTSD-----MYVADEWEFPRDKLEII

RELGKGSFGMVYEGLAKGILPEEEEI-SRVAIKSVQANASMRDRIEFLNEASVMKLIDAH

NVVRLLGVVSKGQPTYVIMEFMAQGDLKNWLRARRPENQQDLPLIDRKSVPTLEQLLNMA

AEIADGMSFLAARKYVHRDLSARNCLVSGEGTCKVADFGLARDIYQSDYYRKERGGMLPI

RWMAPESVKDGVFQASSDVWSFGILLWEMATLGELPYQGLSNEEAGEYIKGG-NVLRPPE

NCPEKMQEIMMACWQYQE-KLRPLFGEIILNLQDYGLLRDS----FAQN-----------

------CFFLNEQL---NASAAPA-DMSSKELETV----SILDQNAKNN-----ENLYTS

MSNNKP-----EGAS-------NGGNRAGGSVEVPIVTGPSSSPKDRNGS----------

-LTGNGSIPHGVGVGKCTEC----------------------------------------

------------------------------------------------------------

------------------------------------------------------------

-----------

>L variegatus

------------------------------------------------------------

------------------------------------------REDNLSINSLKLEVMLRG

FLFILNLVLKLL-INIILFT---------LFVTVCKSMDIRN-----------------S

AEYFSKLENCTVIEGYLQIVLIDHAKPSDYAGLS--------------FPKLREITEYFV

MFRVKNLQTLRHIFPNLAVIRGDSLFFNYALIIFEMFELQEIGLPSLQAVMRGWVRIEKN

INLCYLSTVDWSLVQVQGMENNFIK-GNKDQ-EECFNFCP-----EDDGKRLCRKLTATG

-----DVAELCWTGQDCQKVC------PVSC-PRSCNEAGRCCHEQCIGGCRGS-TASDC

TACRHFLHLGVCVGQCPNRTFQVKNRHCITAEQCPA--------------GWKLFNEKCT

QDCPGGYMPDFNDSRHCLPCTGTCPKVC----DGKTIQSVEDAAAMKGCTYVHGSLTISV

HGR--GTVVKELEQNLGMIEVVSDALIVRRANSLVSLNFLRNLRRIEGRVGGLEMSEYSL

YVLANNNLQQLFDFSQHPNISIGNGKFFFHYNPKLCYSEIEKLHNLTGIKAPLSNIDVSL

SSNGDQFAGNTHRLNLEFAFFQSV---LSVRWSP--PKGKGDVREIVGYILSYREAPQRN

VTELDGED-ACGTSLWEVQYIPPTETST------------FVANLKPWTQYAFMVR---T

YTIAGAQFSARSNIQYLLTPESEASPPSSLNVISNSSSELVITWKPPEHPNGNLTHYIIT

WEPRYLFKQQFEDRDFCRQEL-PTLRKSE---------------------SSTEMETSED

EDTNTTMVGAG-CCTCPIDPEELRRREE------DARFQKDFENQLHNNIYMAYATSSAT

RSG---ASQYIHYAIDDIEVSKLQYELHTPLSKQ---------YKSCYFIYGMILCYIRY

ALPA--------------------LIDQHIHSFI---SRYIAHYYRDSQRHHLPYRSWS-

----ASKIISICPMPIARIYESADQIESTVMINHTSTD--RVHLTWAAPTNPNGIVVIHE

VRFEMVDPKVDDVGDERDAYTVVESLSTPKPVTQSTENTECVSAEG--YRRVLGAELLGL

KVGNYTAMVRAVSLAGEGPWTKPVTFSVPDITNSCYIIAAAVAKSQFPSLSIVQYCHIMH

YSYIYSIICYIIYRK--DQMPDGVLYASVNPEYIIISP-----VYVADEWEFPHDKLEII

KKLGKGSFGMVYEGLAKGILPDEEDI-SRVAIKSVQANASMRDRIEFLNEASYSK-IEAH

HVVRLLGVVSKGQPTYVIMEFMAQGDLKNWLRARRPENQQDLPLIDRKNVPSLRQLIYMA

AQIADGMAYLSSQRFVHRDLAARNILVGENYTCKIADFGLARDIYQSDYYRKEGGAKFPI

RWMAPESLLYGKFTIKSDVWSFGVLLWEIVTLGRIPYPGLSNAEVPKYIKGG-NSLRPPE

NCSPQM-AILFQSWYYEKICLRSSALKNLARQGARCIYA------KEKI-----------

--------CFSKPEIYHKIHCSDFYKLPLDLELIPYICMSVLILIHCLQYD---KTLCQK

VSLFQS-----ASACC------------LQGVWCVVGDHSKRSIAVP-------------

-SELKCRGPQVRGIWTC-------------------------------------------

------------------------------------------------------------

------------------------------------------------------------

-----------

>M musculus

------------------------------------------------------------

------------------------------------------MGFGRGCETTAVPLLVAV

AALLVGTAGHLY------------------PGEVCPGMDIRN-----------------N

LTRLHELENCSVIEGHLQILLMFKTRPEDFRDLS--------------FPKLIMITDYLL

LFRVYGLESLKDLFPNLTVIRGSRLFFNYALVIFEMVHLKELGLYNLMNITRGSVRIEKN

NELCYLATIDWSRI-LDSVEDNYIV-LNKDDNEECGDVCPG----TAKGKTNCPATVING

-----QFVERCWTHSHCQKVC------PTICKSHGCTAEGLCCHKECLGNCSEPDDPTKC

VACRNFYLDGQCVETCPPPYYHFQDWRCVNFSFCQDLHFKCRNSRKPGCHQYVIHNNKCI

PECPSGYTMNSSNL-MCTPCLGPCPKVCQILEGEKTIDSVTSAQELRGCTVINGSLIINI

RGG--NNLAAELEANLGLIEEISGFLKIRRSYALVSLSFFRKLHLIRGET--LEIGNYSF

YALDNQNLRQLWDWSKH-NLTITQGKLFFHYNPKLCLSEIHKMEEVSGTKGRQERNDIAL

KTNGDQASCENEL--LKFSFIRTSFDKILLRWEPYWPP---DFRDLLGFMLFYKEAPYQN

VTEFDGQD-ACGSNSWTVVDIDPPQRSNDPKSQTPSHPGWLMRGLKPWTQYAIFVKTLVT

FSDERRTYGAKSDIIYVQTDATNPSVPLDPISVSNSSSQIILKWKPPSDPNGNITHYLVY

WERQAEDSELFE-LDYCLKGLKLPSRTWS---------------------PPFESDDSQK

HNQSEYDDSASECCSCPKTDSQILKELE------ESSFRKTFEDYLHNVVFV--PRKTSS

GNGAEDSRPSRKRRSLEEVGNVTATTLTLPD----------------FPNVSSTIVPTSQ

EEHR--------------------PFEKVVNK--ESLVISGLRHFTGYRIELQACNQDSP

D-ERCSVAAYVSARTMPE--AKADDIVGPVTHEIFENN--VVHLMWQEPKEPNGLIVLYE

VSYRRY-------GDE--------------------ELHLCVSRKH--FALERGCRLRGL

SPGNYSVRVRATSLAGNGSWTEPTYFYVTDYLDVPSNIAKIIIG---PLIFVFLFSVVIG

SIYLF-------LRKRQPDGPMGPLYASSNPEYLSASDVFPSSVYVPDEWEVPREKITLL

RELGQGSFGMVYEGNAKDIIKGEAE--TRVAVKTVNESASLRERIEFLNEASVMKGFTCH

HVVRLLGVVSKGQPTLVVMELMAHGDLKSHLRSLRPDAENN-PG---RPPPTLQEMIQMT

AEIADGMAYLNAKKFVHRDLAARNCMVAHDFTVKIGDFGMTRDIYETDYYRKGGKGLLPV

RWMSPESLKDGVFTASSDMWSFGVVLWEITSLAEQPYQGLSNEQVLKFVMDG-GYLDPPD

NCPERLTDLMRMCWQFNP-KMRPTFLEIVNLLKDDLHPS------FPEV-----------

--------SFFYSEEN---KAPESEELEMEFEDMENV--PLDRSSHCQREEAGGREGGSS

LSIKRT-----YDEH-------------IPYTHMNGGKKNGRVLTLP-------------

------RSNPS-------------------------------------------------

------------------------------------------------------------

------------------------------------------------------------

-----------

>C elegans

MTRMNIVRCRRRHKILENLEEENLGPSCSSTTSTTAATEALGTTTEDMRLKQQRSSSRAT

EHDIVDGNHHDDEHITMRRLRLVKNSRTRRRTTPDSSMDCYEENPPSQKTSINYSWISKK

SSMTSLMLLLLFAFVQPCAS---------IVEKRCGPIDIRNRPWDIKPQWSKLGDPNEK

DLAGQRMVNCTVVEGSLTISFVLKHKTKAQEEMHRSLQPRYSQDEFITFPHLREITGTLL

VFETEGLVDLRKIFPNLRVIGGRSLIQHYALIIYRNPDL-EIGLDKLSVIRNGGVRIIDN

RKLCYTKTIDWKHLITSSINDVVVDNAAEYAVTETGLMCPRGACEEDKGESKCHYLEEKN

QEQGVERVQSCWSNTTCQKSCAYDRLLPTKEIGPGCDANGDRCHDQCVGGCERVNDATAC

HACKNVYHKGKCIEKCDAHLYLLLQRRCVTREQCLQLNPVLSN----KTVPIKATAGLCS

DKCPDGYQINPDDHRECRKCVGKCEIVCEIN---HVIDTFPKAQAIRLCNIIDGNLTIEI

RGKQDSGMASELKDIFANIHTITGYLLVRQSSPFISLNMFRNLRRIEAKS--LFRNLYAI

TVFENPNLKKLFDSTTD--LTLDRGTVSIANNKMLCFKYIKQLMSKLNIP--LDPIDQSE

GTNGEKAICEDMAINVSITAVNADS--VFFSWPSFNIT-DIDQRKFLGYELFFKEVPRID

ENMTIEEDRSACVDSWQSVFKQYYETSNGEPTPDIFMDIGPRERIRPNTLYAYYVAT--Q

MVLHAGAKNGVSKIGFVRTSYYTPDPP-TLALAQVDSDAIHITWEAPLQPNGDLTHYTIM

WR-ENEVSPYEEAEKFCTDASTPANRQHTKDPKETIVADKPVDIPSSRTVAPTLLTMMGH

EDQQKTCAATPGCCSCSAIEESSEQNKKKRPDPMSAIESSAFENKLLDEVLMPRDTMRVR

RSIEDANRVSEELEKAENLGKAPKTLGGKKPLIHISKKKPSSSSTTSTPAPTIASMYALT

RKPTTVPGTRIRLYEIYEPLPGSWAINVSALALDNSYVIRNLKHYTLYAISLSACQNMTV

PGASCSISHRAGALKRTKHITDIDKVLNETIEWRFMNNSQQVNVTWDPPTEVNGGIFGYV

VKLKSKVDG-------------------------SIVMTRCVGAKRGYSTRNQGVLFQNL

ADGRYFVSVTATSVHGAG--PEAESSDPIVVMTPGFFTVEIILG--MLLVFLILMSIAGC

IIYYY------IQVRYGKKVKALSDFMQLNPEYCVDNK------YNADDWELRQDDVVLG

QQCGEGSFGKVYLGTGNNVVSLMGDRFGPCAIKINVDDPASTENLNYLMEANIMKNFKTN

FIVKLYGVISTVQPAMVVMEMMDLGNLRDYLRSKREDEVFNETDCNFFDIIPRDKFHEWA

AQICDGMAYLESLKFCHRDLAARNCMINRDETVKIGDFGMARDLFYHDYYKPSGKRMMPV

RWMSPESLKDGKFDSKSDVWSFGVVLYEMVTLGAQPYIGLSNDEVLNYIGMARKVIKKPE

CCENYWYKVMKMCWRYSP-RDRPTFLQLVHLLAAEASPEFRDLSFVLTDN---QMILDDS

EALDLDDIDDTDMNDQVVEVAPDVENVEVQSDSERRNTDSIPLKQFKTIPPINATTSHST

ISIDETPMKAKQ----------REGSLDEEYALMNHSGGPSDAEVRTYAGDGDYVERDVR

ENDVPTRRNTGASTSSYTGGGPYCLTNRGGSNERGAGFGEAVRLTDGVGSGHLNDDDYVE

KEISSMDTRRSTGASSSSYGVPQTNWSGNRGATYYTSKAQQAATAAAAAAAALQQQQNGG

RGDRLTQLPGTGHLQSTRGGQDGDYIETEPKNYRNNGSPSRNGNSRDIFNGRSAFGENEH

LIEDNEHHPLV

IRS

>H sapiens

MASPPESD--------GFSDVRKVGYLRKPKSMHKRFFVLRAASEAGGPARLEYYENEKK

WRHKSSAPKRSIPLESCFNINKRADSKNKHLVALYTRDEHFAIAADSEAEQDSWYQALLQ

LHNRAKGHHDGAAALGAGGGGGSCSGSSGLGEAGEDLSYGDVPPGPAFK-EVWQVILKPK

GLGQTKNLIGIYRLCLTSKTISFVKLNSEAAAVVLQLMNIRRCGHSENFFFIEVGRSAVT

GPGEFWMQVDDSVVAQNMHETILEAMRAM--SDEFRPRSKSQSSSNCSNPISVPLRRHHL

NNPPPSQVGLTRRSRTESITATSPASMVGGKPGSFRVRASSDGEGTMSRPASVDGSPVSP

STNRTHAHRHRGSARLHPPLNHSRSIPMPASRCSPSATSPVSLSSSSTSGHGSTSDCLFP

RRSSASVSGSPSDGGFISSDEYGSSPCDFRSSFRSVTPDSLGHTPPARGEEELSNYICMG

GKGPSTLTAPNGHYILSRGGNGHRCTPGTGLGTSPALAGDEAASAADLDNRFRKRTHSAG

TSPTITHQKTPSQSSVASIEEYTEMMP-AYPPGGGSGGRLPGHRHSAFVPTRSYPEEGLE

MHPLERRGGHHRPDSSTLHTDDGYMPMSPGVAPVPSGRKGSGDYMPMSPKSVSAPQQIIN

PIRRHPQRVDPNGYMMMSPSGGCSPDIGGGPSSSSSSSNAVPSGTSYGKLWTNGVGGHHS

HVLPHPKPPVESSGGKLLPCTGDYMNMSPVGDSNTSSPSDCYYGPEDPQHKPVLSYYSLP

RSFKHTQRPGEPEEGARHQHLRLSTSSGRLLYAATADDSSSSTSSDSLGGGYCGARLEPS

LP-HPHHQVLQPHLPRKVDTAAQTNSRLARPTRLSLGDPKASTLPRAREQQQQQQPLLHP

PEPKSPGEYVNIEFGSDQSGYLSGPVAFHSSPSVRCPSQLQPAPREEETGTEEYMKMDLG

PGRRAAWQESTGVEMGRLGPAPPGAASICRPTRAVP--SSRGDYMTMQMSCPRQSYVDTS

PAAPVSYADMRTGIAAEEVSLPRATMAAASSSSAASASPTGPQGAAELAAHSSLLGGPQG

PGGMSAFTRVNLSPNRNQSAKVIRADPQGCRRRHSSETFSSTPSATRVGNTVPFGAGAAV

GG-GGGSSSSSEDVKRHSSASFENVWLRPGELGGAPKEPAKLCGAAGGLENGLNYIDLDL

VKDFKQCPQECTPEPQPPPPPPPHQPLGSGESSSTRRSSEDLSAYASISFQKQPEDRQ

>H glaber

MASPPDTD--------GFSDVRKVGYLRKPKSMHKRFFVLRAASEAGGPARLEYYENEKK

WRHKSSAPKRSIPLETCFNINKRADSKNKHLVALYTRDEHFAIAADSEAEQDSWYQALLQ

LHNRAKGHHDGAAVPGAGGGGGSCSGSSGVGEAGEDLSYGDVPPGPAFK-EVWQVILKPK

GLGQTKNLIGIYRLCLTSKTISFVKLNSEAAAVVLQLMNIRRCGHSENFFFIEVGRSAVT

GPGEFWMQVDDSVVAQNMHETILEAMRAM--SDEFRPRSKSQSSSNCSNPISVPLRRHHL

NNPPPSQVGLTRRSRTESITATSPASMVGGKPGSFRVRASSDGEGTMSRPASVDGSPVSP

STNRTHAHRHRGSSRLHPPLNHSRSIPMPSSRCSPSATSPVSLSSSSTSGHGSTSDCLFP

RRSSASVSGSPSDGGFISSDEYGSSPCDFRSSFRSVTPDSLGHTPPARGEEELSNYICMG

GKGASTLTAPNGHYILSRGGNGHRYLPGAGLGTSPALAGEEAASAADLENRFRKRTHSAG

TSPTISHQKTPSQSSVASIEEYTEMMP-AYPPGGGSGGRLPSYRHSAFVPTHSYPEEGLE

MHPMEQRGGHPRPDSSGLHTDDGYMPMSPGVAPVPGSRKGSGDYMPMSPKSVSAPQQIIN

PIRRHPQRVDPNGYMMMSPSGSCSPDIGGG-SSSSSSISAAPSGSSYGKLWTNGVGGHHS

HALSHPRPPVESGGSKLLPCTSDYMNMSPVGDSNTSSPSDCYYGSEDPQHKPVLSYYSLP

RSFKHTQRPGEPEEGARHQHLRLSSSSGRLLYAAAAEDSSSSTSSDSLGGGYCGARPEAG

PP-HPHHHVLQPRLPRKVDTAAQSNSRLARPTRLSLGDPKASTLPRARE--QQQPPLVHP

PEPKSPGEYVNIEFGSGQPGYFSGPMASRSFPSVRC--QLQPAPREEETGTEEYMNMDLG

PGRRAAWQESSGAETGRAGPAPPGAASVCRPTRAVP--SSRGDYMTMQMGCPRQNYVDTS

PVTPVSYADMRTGI-AEEVSLPRATVAAPSSSSAAPASPTAPQGAAELAAHSSLLGGPQG

PGGVSAFTRVNLSPNRNQSAKVIRADAPGCRRRHSSETFSSTPAATRAGNTVPFGGGAAV

GG-SGGSSSSSEDVKRHSSASFENVWPRPGEQGGAPKDTAQACAAAGGLENGLHYIDLDL

VKDLKQRPQERPSQLQAPLPPPPHHPLGSSESSSTSRSSEDLSAYASISFQKQPEDRQ

>M brandtii

MASPTETE--------GFSDVRKVGYLRKPKSMHKRFFVLRAASEAGGPARLEYYENEKK

WRHKSSAPKRSIPLESCFNINKRADSKNKHLVALYTRDEHFAIAADSEAEQDSWYQALLQ

LHNRAKSHHDGASAPGTGGGGGSCSGSSGVGEAGEDLSFGDSNPGPAFK-EVWQVVLKPK

GLGQTKNLIGIYRLCLTSKTISFVKLNTEAAAVVLQLMNIRRCGHSENFFFIEVGRSAVT

GPGEFWMQVEDSVVAQSMHETILEAMRAM--SDEFRPRSKSQSSSNCSNPISVPLRRHHL

NNPPPSQVGLTRRSRTESITATSPASLVGGKQGSFRVRASSDGEGTMSRPASVDGSPVSP

STNRTHAHRHRGCSRLHPPLNHSRSIPMPSSRCSPSATSPVSLSSSSTSGHGSTSDCLFP

RRSSASVSGSPSDGGFISSDEYGSSPCDFRSSFRSVTPDSLGHTPPARGEEELSNYICMG

GKGASTLAAPNGHYILPRGGNGHRYLPATGLGTNSALAGDEAASAADLDNRFRKRTHSAG

TSPTISHQKTPSQSSVASIEEYTEMMP-ACPPGGGSGGRLPGYRHSAFLPTQSYPEEGLE

MHPLERRGGHSRPDTSTLHTDDGYMPMTPGVAPVPGSRKGSGDYMPMSPKSVSSPQQIIN

PLRRHPQRVDPNGYMMMSPSGSSSPDTGGGPSSSNS---AAPSRSSYGKLWTNGVGGHHA

HPLPPSKLPSESSGGKLLPCTGDYMNMSPVGDSNTSSPSDCYCGPEDPQHKSVLSYCSLP

RSFKHTQRPGDLEETARHQHLRLSSSSGRLLYAAAAEDSSSSTSNDSLGGGYCGVRPEPS

LPQHLHHQVLQSHLPRKVDSAAQTNSRLARPTRLSLGDPKASTLPRARE--QQQPALLLP

PEPKSPGEYVNIEFGSDQPGYLSGPVASHSSPSVRCPSQLQPAPREEESGTEEYMNMDLG

PGRRATWQESPVVQPSRVGPAPPGTASTCRPTRAVPSTSTRGDYMTMQMGCPHQSYVDTS

PVAPVSYADIRTGI-VEEASLPGAIAAAPSSSSTASASPTAPQGAGELVA-------PQG

SGGTSAFTRVNLSPSGNQSAKVIRADPQGCRRRHSSETFSSMPSTPRAGNMVPSG-GAAV

GG-SSGGSSSIEDVKRHSSASFENVWLRPGELGGAPKEQAQVCGAAGGLENGLNYIDLDL

VKDFKQHPQEHPRQVQHPQPPTLRHPPGSSESSSTSRSREDVSAYASISFQKQPEDLQ

>S farnciscanus

MSSQKENVPIPCGVELSIGDIEKSGYLRKLKTMRKKYFVLRSESNT-GPSRLEYYDNEKK

FRL-GGEAKRTVPLSACFNINKKSDAKHKHAIALYTRDDCFSLVADDEESQQEWLRALLE

----------------------------------QQRAGGDGKPVPAFE-HVWQVTVKPK

GLGSSRQLSGIYRLCLTTSTISLVRMNSENEGLELQLSAIRRCGHSDCFFFMEVGRQSVT

GPGELWMQVEDTVIAQSIHEASIRAMRSVKQQEEMRPRSHS-------------------

------------------------STMSSGNPGS--------------------------

------------------------------------------------------------

DSKSHSASGSRRQSNTL-------------------------------------------

-------------------GPSWR----------------TRCDSMPVPNRAM-------

------------------------------------------------------------

----------------------------------PGSRSG--------------------

------------------------------------------------------------

--------------------------------SRSRGNSEGEDGQSKEEHAIASS--SSP

ISL---------------------------------------------------------

------------------------------------------------------------

-----------LKFGN----------------------KLRPRTSSE-------------

-----------------------------------------GE-----------------

-----------KGM-------------------KAP------------------------

------------------------------------------------------------

------------------------------------------------------------

----------------------------------------------------------

>S purpuratus

MSSQKENVPIPCGVELSIGDIEKSGYLRKLKTMRKKYFVLRSESIT-GPSRLEYYDNEKK

FRL-GGEAKRTVPLSACFNINKKSDAKHKHAIALYTRDDCFSLVADDEESKQEWLRVLLE

----------------------------------QQRAGGDGKPVPAFE-HVWQVTVKPK

GLGSSRQLSGIYRLCLTTSTISLVRMNSENEGLEFSLSAIRRCGHSDCFFFMEVGRQSVT

GPGELWMQVEDTVIAQSIHEASLEAMRSVKQQEEMRPRSHS-------------------

------------------------STMSSGNPGS--------------------------

------------------------------------------------------------

DSKSHSASGSRRQSNTL-------------------------------------------

-------------------GPSWR----------------TRCDSMPVPNRAM-------

------------------------------------------------------------

----------------------------------PGSRSG--------------------

------------------------------------------------------------

--------------------------------SRSRGNSEGEDGQSKEEHAIASS--SSP

VSL---------------------------------------------------------

------------------------------------------------------------

-----------LKFGN----------------------KLRPRTSSE-------------

-----------------------------------------GE-----------------

-----------KGM-------------------KAPDSPTKTRGRFSIPSLTSRSSLSSG

PIFVMVLDLSILRSVGSIFQKLLPTKKDFMKVKV--------------------------

------------------------------------------------------------

----------------------------------------------------------

>L variegatus

MSSQKENVPIPCGVELSIGDIEKSGYLRKLKTMRKKFFVLRSESNT-GPSRLEYYDNEKK

FRL-GGEPKRTVPLSACFNINKKSDAKHKHAIALYTRDDCFSLVADDEESQQEWLRAQFQ

------------------------------------YPYCHAHILYAIKEHVWQVTVKPK

GLGSSRQLSGIYRLCLTTSTISLVKMNSENQGLEFSLSAIRSCAHSDCFFFMEVGRQSVT

GPGELWMQVEDTVIAQSIHEASLEVFHQP-------------------------------

---------------------------SCDKPGGCFIKL---------------------

------------------------------------------------------------

---------------FV-------------------------------------------

-------------YVKSDYRNDWSFLVVNGI-----FIGDIACKK---------------

------------------------------------------------------------

--------------------------MYPTCLIISHFHYC--------------------

------------------------------------------------------------

--------------------------------ANHSKKTNTSCSLYQSHYCILCVPLSFI

ISL---------------------------------------------------------

------------------------------------------------------------

-----------LKFGN----------------------KLRPRTSSE-------------

-----------------------------------------GE-----------------

-----------KGM-------------------KAP------------------------

-------------------------DRYVC------------------------------

------------------------------------------------------------

----------------------------------------------------------

>M musculus

MASPPDTD--------GFSDVRKVGYLRKPKSMHKRFFVLRAASEAGGPARLEYYENEKK

WRHKSSAPKRSIPLESCFNINKRADSKNKHLVALYTRDEHFAIAADSEAEQDSWYQALLQ

LHNRAKAHHDGAG----GGCGGSCSGSSGVGEAGEDLSY-DTGPGPAFK-EVWQVILKPK

GLGQTKNLIGIYRLCLTSKTISFVKLNSEAAAVVLQLMNIRRCGHSENFFFIEVGRSAVT

GPGEFWMQVDDSVVAQNMHETILEAMRAM--SDEFRPRSKSQSSSSCSNPISVPLRRHHL

NNPPPSQVGLTRRSRTESITATSPASMVGGKPGSFRVRASSDGEGTMSRPASVDGSPVSP

STNRTHAHRHRGSSRLHPPLNHSRSIPMPSSRCSPSATSPVSLSSSSTSGHGSTSDCLFP

RRSSASVSGSPSDGGFISSDEYGSSPCDFRSSFRSVTPDSLGHTPPARGEEELSNYICMG

GKGASTLAAPNGHYILSRGGNGHRYIPGANLGTSPALPGDEAAGAADLDNRFRKRTHSAG

TSPTISHQKTPSQSSVASIEEYTEMMPAAYPPGGGSGGRLPGYRHSAFVPTHSYPEEGLE

MHHLERRGGHHRPDTSNLHTDDGYMPMSPGVAPVPSNRKGNGDYMPMSPKSVSAPQQIIN

PIRRHPQRVDPNGYMMMSPSGSCSPDIGGG-SSSSSSISAAPSGSSYGKPWTNGVGGHHT

HALPHAKPPVESGGGKLLPCTGDYMNMSPVGDSNTSSPSECYYGPEDPQHKPVLSYYSLP

RSFKHTQRPGEPEEGARHQHLRLSSSSGRLRYTATAEDSSSSTSSDSLGGGYCGARPESS

LT-HPHHHVLQPHLPRKVDTAAQTNSRLARPTRLSLGDPKASTLPRVREQQQQQQSSLHP

PEPKSPGEYVNIEFGSGQPGYLAGPATSRSSPSVRCPPQLHPAPREE-TGSEEYMNMDLG

PGRRATWQESGGVELGRIGPAPPGSATVCRPTRSVP--NSRGDYMTMQIGCPRQSYVDTS

PVAPVSYADMRTGIAAEKASLPRPTGAAPPPSSTASSSVTPQGATAEQATHSSLLGGPQG

PGGMSAFTRVNLSPNHNQSAKVIRADTQGCRRRHSSETFS---APTRAGNTVPFGAGAAV

GGSGGGGGGGSEDVKRHSSASFENVWLRPGDLGGVSKESAPVCGAAGGLEKSLNYIDLDL

AKE---RSQDCPSQQQSLPPPPPHQPLGSNEGNSPRRSSEDLSNYASISFQKQPEDRQ

LDLR

>H sapiens

------MGPWGWKLRWTVALLLAAAGTAVGDRCERNEFQCQDGKCISYKWVCDGSAECQD

GSDESQ-----------------------------------------------------E

TCLSVTCKSGDFSCGGRVNRCIPQFWRCDGQVDCDNGSDEQGCPPKTCSQDEFRCHDGKC

ISRQFVCDSDRDCLDGSDEASCPVLTCGPASFQC---NSSTCIPQLWACDNDPDCEDGSD

EWPQRCRGLYV--FQGDSSPCSAFEFHCLSGECIHSSWRCDGGPDCKDKSDEENCAVAT-

--CRPDEFQCSDGNCIHGSRQCDREYDCKDMSDEVGCVNVTLCEGPNKFKCHSGECITLD

KVCNMARDCRDWSDEPIKE-CGTNECLDNNGGCSHVCNDLKIGYECLCPDGFQLVAQRR-

---CEDIDECQD-PDTCSQLCVNLEGGYKCQCEEGFQLD-PHTKA-CKA--VGSIAYLFF

TNRHEVRKMTLDRSEYTSLIPNLRNVVALD---TEVASNRIYWSDLSQRMICSTQLDRAH

GVSSYDTVISRDIQAPDGLAVDWIHSNIYWTDSVLGTVSVADTKGVKRKTLFRENGSKPR

AIVVDPVHGFMYWTDWGTPAKIKKGGLNGVDIYSLVTENIQWPNGITLDLLSGRLYWVDS

KLHSISSIDVNGGNRKTILEDEKRLAHPFSLAVFEDKVFWTDIINEAIFSANRLTGSDVN

LLAENLLSPEDMVLFHNLTQPRGVNWCERTT-LSNGGCQYLCLPAPQINPHSPKFTCACP

DGMLLARDMRSCLTEAEAAVATQETSTVRLKVSSTAVR-TQHTTTRPVPDTSRLPGATPG

LTTVEIVT-MSHQALGDVAGRGN-EKKPSSVRALSIVLPIVLLVFLCLGVFLLWKNWRLK

NINSINFDNPVYQKTTEDEVHICHNQDGYSYPSRQMVSLEDDVA

>H glaber

MQCAPRMRTAGPVLRYGALLLCLAAGAA-EDKCARNEFQCWDGKCISYTWVCDGRAECQD

GSDESQ-----------------------------------------------------E

TCMAATCKPGDFSCGGRLNRCIPSSWRCDGHEDCNNGADEQGCPTRTCPEDEFHCQDGRC

ISQQFLCDGDRDCLDGSDEVTCSVTTCGPANFQC---NSSACIPQLWACDGDSDCSDGSD

EWPQRCEGRDTSAAQGANGPCSSLEFHCRSGECIHSSWHCDGAHDCKDKSDEEDCVVAT-

--CRPDEFQCADGTCIHGSRQCDQEHDCRDQSDEVGCINVTLCEGPNKFKCHSGECITLD

KVCNAARDCRDWSDEPLKE-CGTNECLDNNGGCSHICKDLKIGYQCLCPSGFHLADQWQ-

---CKDIDECQE-PDTCSQLCVNLEGSYKCECEDGFQMD-PSTKT-CKA--VGSVAYLFF

TNRHEVRKMTPDRSQYASLLSNLKYAVALD---AEVASSRLYWSDLSHRKIYSTRISAAP

GS-AHDTVIDGDLQAPDGLAVDWVHRNIYWTDSVPGTVSVADTRGVKRRTLFQRKGSKPR

GIVVDPVHGFMYWTDWGTPAKIEKGGLNGVDMYSLVTEDIEWPNGITLDLSSSRLYWVDS

KLHSIFSINVNGRNRKTILEDEKQLAHPFSLAIFEDKIFWTDIINEAIFSVNRLTGLDVH

LVAENLSSPEDIVLFHNVTQPKGVNWCERTA-LPNGGCQYLCLPAPQINSHSPKFTCVCP

DGMVLAKDMRSCLTEAEPSVTTPVHPTARPEASSTAVR-PKHADQWPAPSTSGRPVATSG

LTTAASVT-MSHQVVGNAAGRG---EKPHSVGALTIVLPIGLVALLCLGAFLLWKNWRLK

NINSINFDNPVYQKTTEDEVHICRSQDGYIYPSRQRVSLEDDVV

>M brandtii

------------------------------------------------------------

------------------------------------------------------------

------------------------------------------------------------

----------------MREKRRSAASCTPP-------------------TGDVPAT----

-------------------------------KCIHRSWRCDGSADCKDKSDEENCAVAT-

--CRPDEFQCFDGTCIHGSQQCDREHDCKDMSDELGCINVTLCEGPNKFKCHSGECITMD

KVCNSVRDCRDWSDEPLKE-CETNECLTNKGGCSHLCKDLKIGYECLCPEGFRLADQRR-

---CEDIDECED-PDACSQLCVNSVGSYQCKCEEGFRLD-PFTKA-CKA--VDTIAYLFF

TNRHEVRKMTLDRSEYTSLIPNLKNVVALD---VEVHANRVYWSDLSQRKIYSTQIHRES

NS-SYDTIIDEGLQAPDGLAVDWIHGNLYWTDSMLGTVSVANTKGVKRKTLFKEKGSKPR

AIVVDPRHGFMYWTDWGTPAKIKKGGLNGVDIHSLVTEDIQWPNGITLDLSSDRLYWVDS

KLHSISSIDLTGRHRKTILEDKKRLAHPFSLAIFEDKVFWTDIINEAIFSANRHTGSDIN

LMAENLMSPEDIVLSHRLTQPKGINWCETTF-YSYGGCQYLCLPAPQINSYSPKFTCACP

DGMLLAEDRRSC--RPEPVVTTRG------------------------PNMTRSTVTTPE

LTTAKMVTKAPPQASEDVPIKEDRKERPRGVGWLYIVLPIGLLTLLCFGAFLLWKNWRLK

SINSIHFDNPVYQKTTEDEVHICHSQDGYTYPSRQMVRMEDDMA

>S franciscanus

LLLSLLLSSHCITLILL------FYSGS-AILCGEYQFRCQNGHCIPSYWQCDNYDDCGD

NSDEQSCPKMYMYSLEYSY---------SYIIHCRIRIRMIYYIPCPNPSLRIAT----S

HAETLTCSPSQFSCGPGKN-CIPLTWRCDRDVDCPSGADEHNCDALTCASNQFTCNNKKC

VTTRWVCDGDNDCGDGSDEINCPSLTCAPDEFECTGTNETTCIPNRWKCDGEIDCRDGSD

ESSTHCSTATT--------PCSAGQFQCASGRCIHAAWKCDGERDCGDGSDEESCCLVTP

SPCAASEYRCDDGVCIPGDVECDGTQQCADGSDEANCNAPAECNPITHFRCSNSTCLPMV

RVCNGIPECPNNEDEPGQEICGKNECLTNNGGCSHTCMDLAIGHVCTCNAGYALVNETQ-

---CVDINECKTTLGICSQRCYNLPGSYKCDCDENYTLEHVNDRGHCKANPEFGTPKLLI

ANRHDIRIFDLRTSSEDYIRRSLHSAVAVE---FDSASNTVFWTDVGLEVVAKFGKQILD

FF--EMSYISD-VLLPDGVAVDWITRNLYWTDSGRDTIEVSRLDGSSRTVLIDEDLDEPR

AIALDPEGG-MFWSDWGLGV-IERAGMNGQGREAIVTDEIYWPNGLTLDYTLDMLYWIDA

KESSISSVDFMGGNRRVVLAGQGRLPHPFGIAVFEDYIYWTDWESKSIERANKFTGSNQT

TILSGLSDAMDVIVVNPLRQAPS-----------------------K-------------

------------------------------------------------------------

------------------------------------------------------------

--------------------------------------------

>S_purpuratus

------MGTYTLSLILLGLLACVFSAVN-GNLCGENQFRCDNNKCIPSVWKCDNEDDCGD

NSDETVCGQLTCSSAEFECVGSGAGNGGSTCIPARWRCDGDS--DCPDGSDEIA------

-CQTLTCSPSQFSCGPGKN-CIPLTWRCDRDVDCPSGADEHNCDALTCASNQFTCNNKKC

VTTRWVCDGDNDCGDGSDEINCPSLTCAPDEFECTGHNETTCIPNRWKCDGDIDCRDGSD

ESSTHCSGPTV--------PCSAGHFQCTSGECVHNSWKCDGERDCFDGSDEDSCPLVTP

SPCAANEYQCDNGVCIPGDVECDGTQQCADGSDEANCNAPAECNPTTHFKCSNSTCLPMV

RVCNGIPECPNNEDEPGQEICGRNECLTNNGGCSHTCLDLAIGHVCTCNAGYTLVNETQ-

---CVDINECKTTLGICSQRCYNLPGSYKCDCDENYTLENVNDRGHCKANPEFGAPKLLI

ANRHDIRIFDPSTSSEDYIKRSLHSAVAVE---FDSASNTVFWTDVGLEVVSKTQLQAEN

EV--VPVVVTDKVKTPDGIAVDWINKLLYWTDTGVDNIVVSNFEGTRHTVLISSNLDEPR

AIAVDPESGLMFWSDWGLGV-IERAGMNGQGRTAIVTEGVSWPNGLTLDYTLDMLYWIDA

KESSISCVDFMGGNRRVVVAGQGRISHPFSIAVFEDYIYWTDWTKESIERADKFTGSNQT

TILSGVSDAMDVIVVNPLRQAPY-----------------------Q-------------

------------------------------------------------------------

------------------------------------------------------------

--------------------------------------------

>L variegatus

SVIYIVYCPYRVIQSLM------FSFTL-GNLCGGNQFTCANNKCIPSVWKCDNEDDCGD

NSDELVCGKLEASIFILYIILP------PYVAQTKAHAKA----KCASPHYGYIHSLIIA

HTESLTCAPNQFSCGPGKN-CIPLTWRCDHDVDCQTGADEHNCDALTCSANQFTCENKKC

ITTRWVCDGDNDCGDSSDEVNCPSLTCAPDQFECTGANETTCIPGRWKCDGDIDCRDGSD

ESTTHCSDAAI--------PCPQGHFQCNSGECVRLSWKCDGEQDCFDGSDEESCAVITP

TPCAPDEFQCANGVCIQGDVECNNVEECADGSDEANCNGPVDCNPTTHYRCKNSTCLPWV

RVCNGIPECPDNEDEPGQEICGEQRI--------HYIYQIIYAKPYHSKSKYIWSKYHNI

QPEVIIIQVPKSIASYYHRPCVKI-------------IHYPKQLIYCYYHAVRAIPQCQI

KMYVSNRISHINQVVCFYCFQYLFEAIFIDCLVFESCIRCL--NASGCYLSNSSQLVAEN

GV--EPVMVTNKVKTPDGIAVDWITKNLYWTDAGYDRIEVSNLDGTSRTVLISSDLDEPR

AIAVDPESGLMFWSDWGLGV-IERAGMNGQHRTAIVTEKIQWPNALTVDYTLDRIYWIDA

KESSISSVSWMGTNRRVILSGHGRLPHPFAIAVFEDYIYWTDWHTKSIYRANKFTGQDQT

IIQGGLHDPMDIHVFHPQRQVPG-----------------------K-------------

------------------------------------------------------------

------------------------------------------------------------

--------------------------------------------

>M_musculus

------MSTADLMRRWVIALLLAAAGVA-EDSCSRNEFQCRDGKCIASKWVCDGSPECPD

GSDESP-----------------------------------------------------E

TCMSVTCQSNQFSCGGRVSRCIPDSWRCDGQVDCENDSDEQGCPPKTCSQDDFRCQDGKC

ISPQFVCDGDRDCLDGSDEAHCQATTCGPAHFRC---NSSICIPSLWACDGDVDCVDGSD

EWPQNCQGRDT-ASKGVSSPCSSLEFHCGSSECIHRSWVCDGEADCKDKSDEEHCAVAT-

--CRPDEFQCADGSCIHGSRQCDREHDCKDMSDELGCVNVTQCDGPNKFKCHSGECISLD

KVCDSARDCQDWSDEPIKE-CKTNECLDNNGGCSHICKDLKIGSECLCPSGFRLVDLHR-

---CEDIDECQE-PDTCSQLCVNLEGSYKCECQAGFHMD-PHTRV-CKA--VGSIGYLLF

TNRHEVRKMTLDRSEYTSLLPNLKNVVALD---TEVTNNRIYWSDLSQKKIYSALMDQAP

NL-SYDTIISEDLHAPDGLAVDWIHRNIYWTDSVPGSVSVADTKGVKRRTLFQEAGSRPR

AIVVDPVHGFMYWTDWGTPAKIKKGGLNGVDIHSLVTENIQWPNGITLDLSSGRLYWVDS

KLHSISSIDVNGGNRKTILEDENRLAHPFSLAIYEDKVYWTDVINEAIFSANRLTGSDVN

LVAENLLSPEDIVLFHKVTQPRGVNWCETTALLPNGGCQYLCLPAPQIGPHSPKFTCACP

DGMLLAKDMRSCLTEVDTVLTTQGTSAVRPVVTASATRPPKHSEDLSAPSTPRQPVDTPG

LSTVASVT-VSHQVQGDMAGRGN-EEQPHGMRFLSIFFPIALVALLVLGAVLLWRNWRLK

NINSINFDNPVYQKTTEDELHICRSQDGYTYPSRQMVSLEDDVA

>SIG

------------------------------------------------------------

------------------------------------------------------------

------------------------------------------------------------

------------------------------------------------------------

------------------------------------------------------------

------------------------------------------------------------

------------------------------------------------------------

--------------------------------------------+---------------

------------------------------------------------------------

------------------------------------------------------------

------------------------------------------------------------

------------------------------------------------------------

------------------------------------------------------------

------------------------------------------------------------

------------------------------------------------------------

------------------------------------------------------------

------------------------------------------------------------

------------------------------------------------------------

------------------------------------------------------------

------------------------------------------------------------

------------------------------------------------------------

MnSOD

>H sapiens

MLSRAVCGTSRQLAPALGYLGSRQKHSLPDLPYDYGALEPHINAQIMQLHHSKHHA-AYV

NNLNVTEEKYQEALA---------------------------------------KGELLE

AIKRDFGSFDKFKEKLTAASVGVQGSGWGWLGFNKERGHLQIAACPNQDPLQGTTGLIPL

LGIDVWEHAYYLQYKNVRPDYLKAIWNVINWENVTERYMACKK-

>H glaber

MLPYAACSASRRLAPALGLLGVRQKHSLPDLPYDYGALEPHINAQIMQLHHSKHHA-AYV

NNLNATEEKYQEALVKGDVTTQVALQPALRFNGGGHINHTIFWTNLSPNGGGEPKGELLE

AIKRDFGSFDKFKEKLTAVSVGVQGSGWGWLGFNKEQGRLQIAACSNQDPLQGTTGLIPL

LGIDVWEHAYYLQYKNVRPDYLKAIWNVINWENVTERYMACKK-

>S franciscanus

------------------------------------------------IHHSHSSNPILI

VDILLVTINLYSVPSLGDISTIISLQAALRFNGGGHINHTIFWQNLSPDGGGEPTGILLN

VIKKDFGSFDDMRSKLSAATVAIQGSGWGWLGYNKGTRSLQIATCPNQDPLKATAGLIPL

FGIDVWEHAYYLQYKNVRPDYVKAIFNIANWSDVERRLADAMTT

>S purpuratus

----------------------------------------------MELHHKKHHN-TYV

TNLNVAEEALEHAVEAGDISTIISLQAALRFNGGGHINHTIFWQNLSPDGGGEPTGELLN

VIKKDFGSYDDMRSKLSAATVAIQGSGWGWLGYNKGTRSLQIATCPNQDPLKATTGLIPL

FGIDVWEHAYYLQYKNVRPDYVKAIFNIANWSDVEKRLTDAMTT

>L variegatus

-----------------------------------------------GDDTVPVGNVTFV

EVVLLVNIYIILDIFLGDISGIISLQSALRFNGGGHINHTIFWQNLSPDGGGEPTGELLS

AIKKDFGSYDDMRAKLTAATVAIQGSGWGWLGYNKGTGSIQIATCANQDPLQATTGLVPL

FGIDVWEHAYYLQYKNVRPDYVKAIFNIANWVDVERRLRDAMTS

>M musculus

MLCRAACSTGRRLGPVAGAAGSRHKHSLPDLPYDYGALEPHINAQIMQLHHSKHHA-AYV

NNLNATEEKYHEALAKGDVTTQVALQPALKFNGGGHINHTIFWTNLSPKGGGEPKGELLE

AIKRDFGSFEKFKEKLTAVSVGVQGSGWGWLGFNKEQGRLQIAACSNQDPLQGTTGLIPL

LGIDVWEHAYYLQYKNVRPDYLKAIWNVINWENVTERYTACKK-

MTH

>S franciscanus

------------------------------------------------IYQDISFPP---

-------------ESYVRYLNG-------SVRLC--------------------------

-----------------------WTGPDPPEPV------------------SMFQY----

--SKGQNILNLITI----------------------------TLSSICLLATLVTYCIFK

PLRNLQGISIMSFISALLVAQVMLQYIAPKMVSLPTV-CTIAAIITHYLLLVVFTWTNIL

AWDLVRHFASSSFLPKRHGEKRRLIGYLTIGWCLPLII----VVPCLIVQVQNPSLFRYG

KIGTSCWIY-QARAIVLAFLVPVAVSFLVNIILFSFTAYGVHKSKRD-SSVLHEGAQERR

KELFHE---LVVHFQISTLLGFTWLFGFVAAFAKVTALW-YIFIILNSL---QGVFIFIA

FGANKRVRKLWRKKMRESKSVTTSTSSSGSSGSSSDSTSDG

>S purpuratus

------------------------------------------------IYQDISFPP---

-------------ESYVRYLNG-------SVRLC--------------------------

-----------------------WTGPDPPEPV------------------SMFQY----

--SKGQYILNLILL----------------------------VLSSICLLATLITYCIFK

PLRNLQGLSIMSFVSALLVAQLMLQFIAPNMVSLPTV-CTIAAIITHYFLLAIFAWTNIL

AYDLFRCFASSSFLPKRHSQKTRLICYFTFGWCLPLVI----VVPCLITQVQNPSLFRYG

KIGTSCWIY-QARAIVLAFLVPVAASFLVNFVFFLLTAYGVHKSKRD-SSVLHTGVQERR

KEIFTE---LAVHFKISCLLGFGWSFGFIGAFAQSAAVW-TIFIITSSL---QGIFVFIF

FGANQRVRGLWRKKIYGSKARGSTTTGTSSTSVGLRDMSQK

>L variegatus

------------------------------------------------IYQEISFPP---

-------------ESYVRYRNG-------SVRLC--------------------------

-----------------------WTGPDPPEPV------------------SMFAY----

--SRGQYILNAILF----------------------------TLSSICLLATLITYCIFK

PLRNLQGISTMNFATALFIAQVMLQFIAPNMSSIPIA-CTIAAAITHYSLLAAFTWTNIL

AWDLVRHFASSSFLPKRHKETRRLIVYHTIGWCVPLII----IVPCLIVQSQNPSLYRYG

KIGSSCWIY-QAMGIVLAFLVPVAVSFLINIVLFLLTAYGVHKSKRD-SSILHKSAKEQR

KQLFQE---LLVHFQISCLLGFTWSFGFIAAFANVAALW-YIFIILNSL---QGVFIFIA

FGANKRVRDLWRKKVCGSAAQTSSSSSSKTSSTSPGSTTFS

>D melanogaster

MKTLLVLRISTVILVVLVIQKSYADILECDYFDTVDISAAQKLQNGSYLFEGLLVPAILT

GEYDFRILPDDSKQKVARHIRGCVCKLKPCVRFCCPHDHIMDNGVCYDNMSDEELAELDP

FLNVTLDDGSVSRRHFKNELIVQWDLPMPCDGMFYLDNREEQDKYTLFENGTFFRHFDRV

TLRKREYCLQHLTFADGNATSIRIAPHNCLIVPSITGQTVVMISSLICMVLTIAVYLFVK

KLQNLHGKCFICYMVCLFMGYL---FLLLDLWQISISFCKPAGFLGYFFVMAAFFWLSVI

SLHLWNTFRGSSHKANRFLFEHRFLAYNTYAWGMAVVLTGITVLADNIVENQDWNP-RVG

HEG-HCWIYTQAWSAMLYFYGPMVFLIAFNITMFILTAKRILGVKKDIQNFAHR--QERK

QKLNSDKQTYTFFLRLFIIMGLSWSLEIGSYFSQSNQTWANVFLVADYLNWSQGIIIFIL

FVLKRSTWRLLQESIRGEGEEVNNSEEEISLENTTTRNVLL

MTOR

>H sapiens

MLGTGPAAATTAATTSSNVSVLQQFASGLKSRNEETRAKAAKELQHYVTMELREMSQEES

TRFYDQLNHHIFELVSSSDANERKGGILAIASLIGVEGGNATRIGRFANYLRNLLPSNDP

VVMEMASKAIGRLAMAGDTFTAEYVEFEVKRALEWLGADRNEGRRHAAVLVLRELAISVP

TFFFQQVQPFFDNIFVAVWDPKQAIREGAVAALRACLILTTQREPKEMQKPQWY-RHTFE

EAEKGFDETLAKEKGMNRDDRIHGALLILNELVRISSMEGERLREEMEEITQQQLVHDKY

CK------DLMGFGTKPRHITPFTSFQAVQPQQSNALVGLLGYSSHQGLMGFGTSPSPAK

STLVESRCCRDLMEEKFDQVCQ-WVLKCRNSKNSLIQMTILNLLPR------LAAFRPS-

AFTDTQYLQDTMNHVLSCVKKEKERTAAFQALGLLSVAVRSEFKVYLPRVLDIIRAALPP

KDFAHKRQKAMQVDATVFTCISMLARAMGPGIQQDIKELLEPMLAVGLSPALTAVLYDLS

RQIPQLKKDIQDGLLKMLSLVLMHKPLRHPGMPKGLAHQLASPGLT-TLPEASDVGSITL

ALRTLGSFEFEGHSLTQFVRHCADHFLNSEHKEIRMEAARTCSRLLTPSIHLISGHAHVV

SQTAVQVVADVLSKLLVVGITDPDPDIRYCVLASLDERFDAHLAQAENLQALFVALNDQV

FEIRELAICTVGRLSSMNPAFVMPFLRKMLIQILTELEHSGIGRIKEQSARMLGHLVSNA

PRLIRPYMEPILKALILKLKDPDPDPNPGVINNVLATIGELAQVSGLEMRKWVDELFIII

MDMLQDSSLLAKRQVALWTLGQLVASTGYVVEPYRKYPTLLEVLLNFLKTEQNQGTRREA

IRVLGLLGALDPYKHKVNIGMIDQSRDASAVSLSESKSSQDSSDYSTSEMLVNMGNLPLD

EFYPAVSMVALMRIFRDQSLSHHHTMVVQAITFIFKSLGLKCVQFLPQVMPTFLNVIRVC

DGAIREFLFQQLGMLVSFVKSHIRPYMDEIVTLMREFWVMNTSIQSTIILLIEQIVVALG

GEFKLYLPQLIPHMLRVFMHDNSPGRIVSIKLLAAIQLFGANLDDYLHLLLPPIVKLFDA

PEAPLPSRKAALETVDRLTESLDFTDYASRIIHPIVRTLDQSPELRSTAMDTLSSLVFQL

GKKYQIFIPMVNKVLVRHRINHQRYDVLICRIVKGYTLADEEEDPLIYQHRMLRSGQGDA

LASGPVETGPMKKLHVSTINLQK-------------AWGAARRVSKDDWLEWLRRLSLEL

LKDSSSPSLRSCWALAQAYNPMARDLFNAAFVSCWSELNEDQQDELIRSIELALTSQDIA

EVTQTLLNLAEFMEHSDKGPLPLRDDNGIVLLGERAAKCRAYAKALHYKELEFQKGPTPA

ILESLISINNKLQQPEAAAGVLEYAMKHFGELEIQATWYEKLHEWEDALVAYDKKMDTNK

DDPELMLGRMRCLEALGEWGQLHQQCCEKWTLVNDETQAKMARMAAAAAWGLGQWDSMEE

YTCMIPRDTHDGAFYRAVLALHQDLFSLAQQCIDKARDLLDAELTAMAGESYSRAYGAMV

SCHMLSELEEVIQYKLVPERREIIRQIWWERLQGCQRIVEDWQKILMVRSLVVSPHEDMR

TWLKYASLCGKSGRLALAHKTLVLLLGVDPSRQLDHPLPTVHPQVTYAYMKNMWKSARKI

DAFQHMQHFVQTMQQQAQHAIATEDQQHKQELHKLMARCFLKLGEWQLNLQGINESTIPK

VLQYYSAATEHDRSWYKAWHAWAVMNFEAVLHYKHQNQARDEKKKLRHASGANITNATTA

ATTAATATT--TASTEGSNSESEAESTENSPTPSPLQKKVTEDLSKTLLMYTVPAVQGFF

RSISLSRGNNLQDTLRVLTLWFDYGHWPDVNEALVEGVKAIQIDTWLQVIPQLIARIDTP

RPLVGRLIHQLLTDIGRYHPQALIYPLTVASKSTTTARHNAANKILKNMCEHSNTLVQQA

MMVSEELIRVAILWHEMWHEGLEEASRLYFGERNVKGMFEVLEPLHAMMERGPQTLKETS

FNQAYGRDLMEAQEWCRKYMKSGNVKDLTQAWDLYYHVFRRISKQLPQLTSLELQYVSPK

LLMCRDLELAVPGTYDPNQPIIRIQSIAPSLQVITSKQRPRKLTLMGSNGHEFVFLLKGH

EDLRQDERVMQLFGLVNTLLANDPTSLRKNLSIQRYAVIPLSTNSGLIGWVPHCDTLHAL

IRDYREKKKILLNIEHRIMLRMAPDYDHLTLMQKVEVFEHAVNNTAGDDLAKLLWLKSPS

SEVWFDRRTNYTRSLAVMSMVGYILGLGDRHPSNLMLDRLSGKILHIDFGDCFEVAMTRE

KFPEKIPFRLTRMLTNAMEVTGLDGNYRITCHTVMEVLREHKDSVMAVLEAFVYDPLLNW

RLMDTNTKGNKRSRTRTDSYSAGQSVEILDGVELGEPAHKKTGTTVPESIHSFIGDGLVK

PEALNKKAIQIINRVRDKLTGRDFSHDDTLDVPTQVELLIKQATSHENLCQCYIGW----

------------------C-----------------------PFW---------------

------------

>H glaber

MLGTGPAAATTAASTSSNVSVLQQFASGLKSRSEETRAKAAKELQHYVTMELREMSQEES

TRFYDQLNHHIFELVSSSDANERKGGILAIASLIGVEGGNATRIGRFANYLRNLLPSSDP

VVMEMASKAIGRLAMAGDTFTAEYVEFEVKRALEWLGADRNEGRRHAAVLVLRELAISVP

TFFFQQVQPFFDNIFVAVWDPKQAIREGAVAALRACLILTTQREPKEMQKPQWY-RHTFE

EAEKGFDETLAKEKGMNRDDRIHGALLILNELVRISSMEGERLREEMEEITQQQLVHDKY

CK------DLMGFGTKPRHITPFTSFQAVQPQQSNALVGLLGYSSHQGLMGFGASPSPAK

PTLVESRCCRDLMEEKFDQVCQ-WVLKCRNSKNSLIQMTILNLLPR------LAAFRPS-

AFTDTQYLQDTMNHVLSCVKKEKERTAAFQALGLLSVAVRSEFKVYLPRVLDIIRAALPP

KDFAHKRQKAMQVDATVFTCISMLARAMGPGIQQDIKELLEPMLAVGLSPALTAVLYDLS

RQIPQLKKDIQDGLLKMLSLVLMHKPLRHPGMPKGLAHQLASPGLA-TLPEASDVGSITL

ALRTLGSFEFEGHSLTQFVRHCADHFLNSEHKEIRMEAARTCSRLLTPSIHLISGHAHVV

SQTAVQVVADVLSKLLVVGITDPDPDIRYCVLASLDERFDAHLAQAENLQALFVALNDQV

FEIRELAICTVGRLSSMNPAFVMPFLRKMLIQILTELEHSGIGRIKEQSARMLGHLVSNA

PRLIRPYMEPILKALILKLKDPDPDPNPGVINNVLATIGELAQVSGLEMRKWVDELFIII

MDMLQDSSLLAKRQVALWTLGQLVASTGYVVEPYRKYPTLLEVLLNFLKTEQNQGTRREA

IRVLGLLGALDPYKHKVNIGMIDQSRDASAVSLSESKSSQDSSDYSTSEMLVNMGNLPLD

EFYPAVSMVALMRIFRDQSLSHHHTMVVQAITFIFKSLGLKCVQFLPQVMPTFLNVIRVC

DGAIREFLFQQLGMLVSFVKSHIRPYMDEIVTLMREFWVMNTSIQSTIILLIEQIVVALG

GEFKLYLPQLIPHMLRVFMHDNSPGRSVSIKLLAAIQLFGANLDDYLHLLLPPIVKLFDA

PEVPLPSRKAALETVDRLTESLDFTDYASRIIHPIVRTLDQSPELRSTAMDTLSSLVFQL

GKKYQIFIPMVNKVLVRHRINHQRYDVLICRIVKGYTLADEEEDPLIYQHRMQRSGQGDA

LASGPVETGPMKKLHVSTINLQK-------------AWGAARRVSKDDWLEWLRRLSLEL

LKDSSSPSLRSCWALAQAYNPMARDLFNAAFVSCWSELNEDQQDELIRSIELALTSQDIA

EVTQTLLNLAEFMEHSDKGPLPLRDDNGIVLLGERAAKCRAYAKALHYKELEFQKGPTPA

ILESLISINNKLQQPEAAAGVLEYAMKHFGELEIQATWYEKLHEWEDALVAYDKKMDTNK

DDPELVLGRMRCLEALGEWGQLHQQCCEKWTLVNDETQAKMARMAAAAAWGLGQWDSMEE

YTCMIPRDTHDGAFYRAVLALHQDLFSLAQQCIDKARDLLDAELTAMAGESYSRAYGAMV

SCHMLSELEEVIQYKLVPERREIIRQIWWERLQGCQRIVEDWQKILMVRSLVVNPHEDMR

TWLKYASLCGKSGRLALAHKTLVLLLGVDPSRQLDHPLPTVHPQVTYAYMKNMWKSARKI

DAFQHMQHFVQTVQQQAQHAVATEDQQHKQELHKLMARCFLKLGEWQLNLQGINESTIPK

VLQYYSAATEHDRSWYKAWHAWAVMNFEAVLHYKHQNQARDEKKKLRHASGANITNATTA

ATTAATATTTATASTEGSNSESEAESTEHSPTPSPVQKKVTEDLSKTLLMYTVPAVQGFF

RSISLSRGNNLQDTLRVLTLWFDYGHWPDVNEALVEGVKAIQIDTWLQVIPQLIARIDTP

RPLVGRLIHQLLTDIGRYHPQALIYPLTVASKSTTTARHNAANKILKNMCEHSNTLVQQA

MMVSEELIRVAILWHEMWHEGLEEASRLYFGERNVKGMFEVLEPLHAMMERGPQTLKETS

FNQAYGRDLMEAQEWCRKYMKSGNVKDLTQAWDLYYHVFRRISKQLPQLTSLELQYVSPK

LLMCRDLELAVPGTYDPNQPIIRIQSIAPSLQVITSKQRPRKLTLMGSNGHEFVFLLKGH

EDLRQDERVMQLFGLVNTLLANDPTSLRKNLSIQRYAVIPLSTNSGLIGWVPHCDTLHAL

IRDYREKKKILLNIEHRIMLRMAPDYDHLTLMQKVEVFEHAVNNTAGDDLAKLLWLKSPS

SEVWFDRRTNYTRSLAVMSMVGYILGLGDRHPSNLMLDRLSGKILHIDFGDCFEVAMTRE

KFPEKIPFRLTRMLTNAMEVTGLDGNYRITCHTVMEVLREHKDSVMAVLEAFVYDPLLNW

RLMDTNTKGNKRSRTRTDSYSAGQSVEILDGVELGEPAHKKAGTTVPESIHSFIGDGLVK

PEALNKKAIQIINRVRDKLTGRDFSHDDTLDVPTQVELLIKQATSHENLCQCYIGW----

------------------C-----------------------PFW---------------

------------

>M_brandtii

MLGTGPAAAAAAAMASSNVSILQQFASGLKSRNEETRAKAAKELQHYVTMELREMSQEES

TRFYDQLNHHIFELVSSSDANERKGGILAIASLIGVEGGNATRIGRFANYLRNLLPSNDP

VVMEMASKAIGRLAMAGDTFTAEYVEFEVKRALEWLGADRNEGRRHAAVLVLRELAISVP

TFFFQQVQPFFDNIFVAVWDPKQAIREGAVAALRACLILTTQREPKEMQKPQWY-RHTFE

EAEKGFDETLAKEKGMNRDDRIHGALLILNELVRISSMEGERLREEMEEITQQQLVHDKY

CK------DLMGFGTKPRHITPFTSFQAVQPQPSNALVGLLGYSSHQSLMGFGASPSPAK

STLVESRCCRDLMEEKFDQVCQ-WVLKCRNSKNSLIQMTILNLLPR------LAAFRPS-

AFTDTQYLQDTMNHVLSCVKKEKERTAAFQALGLLSVAVRSEFKIYLPRVLDIIRAALPP

KDFAHKRQKAMQVDATVFTCISMLARAMGPGIQQDIKELLEPMLAVGLSPALTAVLYDLS

RQIPQLKKDIQDGLLKMLSLVLMHKPLRHPGMPKGLAHQLASPGLT-TLPEASDVGSITL

ALRTLGSFEFEGHSLTQFVRHCADHFLNSEHKEIRMEAARTCSRLLTPSVHLISGHAHVV

SQTAVQVVADVLSKLLVVGITDPDPDIRYCVLASLDERFDAHLAQAENLQALFVALNDQV

FEIRELAICTVGRLSSMNPAFVMPFLRKMLIQILTELEHSGIGRIKEQSARMLGHLVSNA

PRLIRPYMEPILKALILKLKDPDPDPNPGVINNVLATIGELAQVSGLEMRKWVDELFIII

MDMLQDSSLLAKRQVALWTLGQLVASTGYVVEPYRKYPTLLEVLLNFLKTEQNQGTRREA

IRVLGLLGALDPYKHKVNIGMIDQSRDASAVSLSESKSSQDSSDYSTSEMLVNMGNLPLD

EFYPAVSMVALMRIFRDQSLSHHHTMVVQAITFIFKSLGLKCVQFLPQVMPTYLNVIRVC

DGAIREFLFQQLGMLVSFVKSHIRPYMDEIVTLMREFWVMNTSIQSTIILLIEQIVVALG

GEFKLYLPQLIPHMLRVFMHDNSAGRTVSIKLLAAIQLFGANLDDYLHLLLPPIVKLFDA

PEVPLPSRKAALETVDRLTESLDFTDYASRIIHPIVRTLDQCPELRPTAMDTLSSLVFQL

GKKYQIFIPMVNKVLVRHRINHQRYDVLICRIVKGYTLADEEEDPLIYQHRMLRSGQGDA

LASGPVETGPMKKLHVSTINLQKAADGFPLSPVELQAWGAARRVSKDDWLEWLRRLSLEL

LKDSSSPSLRSCWALAQAYNPMARDLFNAAFVSCWSELNEDQQDELIRSIELALTSQDIA

EVTQTLLNLAEFMEHSDKGPLPLRDDNGIVLLGERAAKCRAYAKALHYKELEFQKGPTPA

ILESLISINNKLQQPEAAAGVLEYAMKHFGELEIQATWYEKLHEWEDALVAYDKKMDTNK

DDPELMLGRMRCLEALGEWGQLHQQCCEKWTLVNDETQAKMARMAAAAAWGLGQWDSMEE

YTCMIPRDTHDGAFYRAVLALHQDLFSLAQQCIDKARDLLDAELTAMAGESYSRAYGAMV

SCHMLSELEEVIQYKLVPERREIIRQIWWERLQGCQRIVEDWQKILMVRSLVVNPHEDMR

TWLKYASLCGKSGRLALAHKTLVLLLGVDPSRQLDHPLPTVHPQVTYAYMKNMWKSARKI

DAFQHMQHFVQTMQQQAQHAIATEDQQHKQELHKLMARCFLKLGEWQLNLQGINESTIPK

VLQYYSAATEHDRSWYKAWHAWAVMNFEAVLHYKHQNQAREEKKKLRHASGANITNTTTA

SSTAAATTT--ATSTEGSNSESEAESSENSPTPSPLQKKVAEDLSKTLLMYTVPAVQGFF

RSISLSRGNNLQDTLRVLTLWFDYGHWPDVNEALVEGVKAIQIDTWLQVIPQLIARIDTP

RPLVGRLIHQLLTDIGRYHPQALIYPLTVASKSTTTARHNAANKILKNMCEHSNTLVQQA

MMVSEELIRVAILWHEMWHEGLEEASRLYFGERNVKGMFEVLEPLHAMMERGPQTLKETS

FNQAYGRDLMEAQEWCRKYMKSGNVKDLTQAWDLYYHVFRRISKQLPQLTSLELQYVSPK

LLMCRDLELAVPGTYDPNQPIIRIQSIAPSLQVITSKQRPRKLTLMGSNGHEFVFLLKGH

EDLRQDERVMQLFGLVNTLLANDPTSLRKNLSIQRYAVIPLSTNSGLIGWVPHCDTLHAL

IRDYREKKKILLNIEHRIMLRMAPDYDHLTLMQKVEVFEHAVNNTAGDDLAKLLWLKSPS

SEVWFDRRTNYTRSLAVMSMVGYILGLGDRHPSNLMLDRLSGKILHIDFGDCFEVAMTRE

KFPEKIPFRLTRMLTNAMEVTGLDGNYRITCHTVMEVLREHKDSVMAVLEAFVYDPLLNW

RLMDTNTKGNKRSRTRTDSYSAGQSVEILNGVELGEPAHKKTGTTVPESIHSFIGDGLVK

PEALNKKAIEIINRVRDKLTGRDFSHDDTLDVPTQVELLIKQATSHENLCQCYIGWHTAN

SAQSTNLGSWRTRPRILDCKEAGGAHFLTHPSHYLTGPATAFARWAKMGGKGLSSASPVM

LRCTTSVQDPDH

>S franciscanus

------------------------------------------------------------

------------------------------------------------------------

------------------------------------------------------------

------------------------------------------------------------

-----FEETGKGEKGLNRDDRAHGALLIINELIRTSSREGE--------------VSLSW

SV------DLGSSSNKAKSPT--------GPQLGHNQYVQRALASPLSLVSTSIQPKP--

-RITESRYCLELMQENFDIVSQ----KCFRGIFCLYIHRGIKIYQKFKE---LLSIQFQ-

NFPKL-CIDNLCMFCF-----ERERSNAFQAVGLLAVAVKGNITEHLGKILEIVRSSLPH

KDIPHRKHKSIIVDPAVFTCISMLARAVGPVIARDVKELLEPMLAVGLRLVFDVPEFAAP

VRLTRPYSCIFHSYISYNSMSEFAARYKHTSL-----NSLDSPGTALSSIDSGDLTSTVL

ALRTLGNFDFEGHSLTQFVRHCADHFLSSEHKEIRIEAARTCSSLLQPAVN---------

------------------------------------------------------------

------------------------------------------------------------

------------------------------------------------------------

------------------------------------------------------------

------------------------------------------------------------

------------------------------------------------------------

---------------VSF------------------------------------------

------------------------------------------------------------

------------------------------------------------------------

------------------------------------------------------------

------------------------------------------------------------

------------------------------------------------------------

------------------------------------------------------------

------------------------------------------------------------

------------------------------------------------------------

-------------------------------------------------------HRVIV

HC----------------------------------------------------------

------------------------------------------------------------

------------------------------------------------------------

------------------------------------------------------------

------------------------------------------------------------

------------------------------------------------------------

------------------------------------------------------------

------------------------------------------------------------

------------------------------------------------------------

------------------------------------------------------------

------------------------------------------------------------

------------------------------------------------------------

--VLF-------------------------------------------------------

------------------------------------------------------------

------------------------------------------------------------

------------------------------------------------------------

------------------------------------------------------------

------------

>S purpuratus

------------------------------------------------------------

------------------------------------------------------------

------------------------------------------------------------

-----------------------------------------------MQKPQWY-KQTYE

EAEKGFEETGKGEKGLNRDDRAHGALLIINELIRTSSREGERLRQEMEEVSQQQTQHEHS

FR------DLGSSSNKAKSPT--------GPQLGHNQYVQRALASPLSLVATGIQPKP--

-RITESRYCLELMQENFDIVCQ-QVLKFCGSRNPLIQQILLNLLPR------LAAFEPK-

MFVTS-YLEDTVKYMLISLRRERERSNAFQAVGLLAVAVKGNITEHLGKILEIVRSSLPH

KDIPHRKHKSIIVDPAVFTCISMLARAVGPVIARDVKELLEPMLAVGLSPALTAALHDLA

KQIPQLKKDIQDGLLKMLSLVLMHKPLRHPGTPRALLSPTGQTGTALSSIDSGDLTSTVL

ALRTLGNFDFEGHSLTQFVRHCADHFLSSEHKEIRIEAARTCSSLLQPAVN---------

------------------------------------------------------------

------------------------------------------------------------

------------------------------------------------------------

------------------------------------------------------------

------------------------------------------------------------

------------------------------------------------------------

---------------VSF------------------------------------------

------------------------------------------------------------

------------------------------------------------------------

------------------------------------------------------------

------------------------------------------------------------

------------------------------------------------------------

------------------------------------------------------------

------------------------------------------------------------

------------------------------------------------------------

-------------------------------------------------------HGVIV

HY----------------------------------------------------------

------------------------------------------------------------

------------------------------------------------------------

------------------------------------------------------------

------------------------------------------------------------

------------------------------------------------------------

------------------------------------------------------------

------------------------------------------------------------

------------------------------------------------------------

------------------------------------------------------------

------------------------------------------------------------

------------------------------------------------------------

--VVF-------------------------------------------------------

------------------------------------------------------------

------------------------------------------------------------

------------------------------------------------------------

------------------------------------------------------------

------------

>L variegatus

------------------------------------------------------------

------------------------------------------------------------

---------------------------------EFIG-----------------------

----------------------------------------------------WVDDQTYE

EAEKGFEEIGKGEKGLNRDDRAHGSLLIINELIRNSSREGEH-------SSQSKLFYNPT

FSDPCLGMDLGSSSNKAKSPT--------GPQLGYNQYVQRALASPLSLGSTSIQPKP--

-RITESRYCLELMQEKFNPVSSVVVLHIRTPK--VARPPIHAYTPRKKSISVFIAISPAI

NFLLSKYPEQTIIYFIEYLQIQQERYAIHGTLYHLIIKIH----EYYPSSL---------

--LAFRKHKSIIVDPAVFTSISMLARAVGPVIARDVKELLEPMLAVGLSPALTAALHDLA

KQIPQLKKDIQDGLLKMLSLVLMHKPLRHPGTPRALLSPASQTGATLSSVDSGDITSTVL

ALRTLGNFDFEGHSLTQFVRHCADHFLSSEHKEIRIEAARTCSSLLQPAVN---------

------------------------------------------------------------

------------------------------------------------------------

------------------------------------------------------------

------------------------------------------------------------

------------------------------------------------------------

------------------------------------------------------------

---------------VSY------------------------------------------

------------------------------------------------------------

------------------------------------------------------------

------------------------------------------------------------

------------------------------------------------------------

------------------------------------------------------------

------------------------------------------------------------

------------------------------------------------------------

------------------------------------------------------------

-------------------------------------------------------YWNSI

SA----------------------------------------------------------

------------------------------------------------------------

------------------------------------------------------------

------------------------------------------------------------

------------------------------------------------------------

------------------------------------------------------------

------------------------------------------------------------

------------------------------------------------------------

------------------------------------------------------------

------------------------------------------------------------

------------------------------------------------------------

------------------------------------------------------------

--KWK-------------------------------------------------------

------------------------------------------------------------

------------------------------------------------------------

------------------------------------------------------------

------------------------------------------------------------

------------

>M musculus

MLGTGPAVATASAATSSNVSVLQQFASGLKSRNEETRAKAAKELQHYVTMELREMSQEES

TRFYDQLNHHIFELVSSSDANERKGGILAIASLIGVEGGNSTRIGRFANYLRNLLPSSDP

VVMEMASKAIGRLAMAGDTFTAEYVEFEVKRALEWLGADRNEGRRHAAVLVLRELAISVP

TFFFQQVQPFFDNIFVAVWDPKQAIREGAVAALRACLILTTQREPKEMQKPQWY-RHTFE

EAEKGFDETLAKEKGMNRDDRIHGALLILNELVRISSMEGERLREEMEEITQQQLVHDKY

CK------DLMGFGTKPRHITPFTSFQAVQPQQPNALVGLLGYSSPQGLMGFGTSPSPAK

STLVESRCCRDLMEEKFDQVCQ-WVLKCRSSKNSLIQMTILNLLPR------LAAFRPS-

AFTDTQYLQDTMNHVLSCVKKEKERTAAFQALGLLSVAVRSEFKVYLPRVLDIIRAALPP

KDFAHKRQKTVQVDATVFTCISMLARAMGPGIQQDIKELLEPMLAVGLSPALTAVLYDLS

RQIPQLKKDIQDGLLKMLSLVLMHKPLRHPGMPKGLAHQLASPGLT-TLPEASDVASITL

ALRTLGSFEFEGHSLTQFVRHCADHFLNSEHKEIRMEAARTCSRLLTPSIHLISGHAHVV

SQTAVQVVADVLSKLLVVGITDPDPDIRYCVLASLDERFDAHLAQAENLQALFVALNDQV

FEIRELAICTVGRLSSMNPAFVMPFLRKMLIQILTELEHSGIGRIKEQSARMLGHLVSNA

PRLIRPYMEPILKALILKLKDPDPDPNPGVINNVLATIGELAQVSGLEMRKWVDELFIII

MDMLQDSSLLAKRQVALWTLGQLVASTGYVVEPYRKYPTLLEVLLNFLKTEQNQGTRREA

IRVLGLLGALDPYKHKVNIGMIDQSRDASAVSLSESKSSQDSSDYSTSEMLVNMGNLPLD

EFYPAVSMVALMRIFRDQSLSHHHTMVVQAITFIFKSLGLKCVQFLPQVMPTFLNVIRVC

DGAIREFLFQQLGMLVSFVKSHIRPYMDEIVTLMREFWVMNTSIQSTIILLIEQIVVALG

GEFKLYLPQLIPHMLRVFMHDNSQGRIVSIKLLAAIQLFGANLDDYLHLLLPPIVKLFDA

PEVPLPSRKAALETVDRLTESLDFTDYASRIIHPIVRTLDQSPELRSTAMDTLSSLVFQL

GKKYQIFIPMVNKVLVRHRINHQRYDVLICRIVKGYTLADEEEDPLIYQHRMLRSSQGDA

LASGPVETGPMKKLHVSTINLQK-------------AWGAARRVSKDDWLEWLRRLSLEL

LKDSSSPSLRSCWALAQAYNPMARDLFNAAFVSCWSELNEDQQDELIRSIELALTSQDIA

EVTQTLLNLAEFMEHSDKGPLPLRDDNGIVLLGERAAKCRAYAKALHYKELEFQKGPTPA

ILESLISINNKLQQPEAASGVLEYAMKHFGELEIQATWYEKLHEWEDALVAYDKKMDTNK

EDPELMLGRMRCLEALGEWGQLHQQCCEKWTLVNDETQAKMARMAAAAAWGLGQWDSMEE

YTCMIPRDTHDGAFYRAVLALHQDLFSLAQQCIDKARDLLDAELTAMAGESYSRAYGAMV

SCHMLSELEEVIQYKLVPERREIIRQIWWERLQGCQRIVEDWQKILMVRSLVVSPHEDMR

TWLKYASLCGKSGRLALAHKTLVLLLGVDPSRQLDHPLPTAHPQVTYAYMKNMWKSARKI

DAFQHMQHFVQTMQQQAQHAIATEDQQHKQELHKLMARCFLKLGEWQLNLQGINESTIPK

VLQYYSAATEHDRSWYKAWHAWAVMNFEAVLHYKHQNQARDEKKKLRHASGANITNATTA

ATTAASAAA--ATSTEGSNSESEAESNENSPTPSPLQKKVTEDLSKTLLLYTVPAVQGFF

RSISLSRGNNLQDTLRVLTLWFDYGHWPDVNEALVEGVKAIQIDTWLQVIPQLIARIDTP

RPLVGRLIHQLLTDIGRYHPQALIYPLTVASKSTTTARHNAANKILKNMCEHSNTLVQQA

MMVSEELIRVAILWHEMWHEGLEEASRLYFGERNVKGMFEVLEPLHAMMERGPQTLKETS

FNQAYGRDLMEAQEWCRKYMKSGNVKDLTQAWDLYYHVFRRISKQLPQLTSLELQYVSPK

LLMCRDLELAVPGTYDPNQPIIRIQSIAPSLQVITSKQRPRKLTLMGSNGHEFVFLLKGH

EDLRQDERVMQLFGLVNTLLANDPTSLRKNLSIQRYAVIPLSTNSGLIGWVPHCDTLHAL

IRDYREKKKILLNIEHRIMLRMAPDYDHLTLMQKVEVFEHAVNNTAGDDLAKLLWLKSPS

SEVWFDRRTNYTRSLAVMSMVGYILGLGDRHPSNLMLDRLSGKILHIDFGDCFEVAMTRE

KFPEKIPFRLTRMLTNAMEVTGLDGNYRTTCHTVMEVLREHKDSVMAVLEAFVYDPLLNW

RLMDTNTKGNKRSRTRTDSYSAGQSVEILDGVELGEPAHKKAGTTVPESIHSFIGDGLVK

PEALNKKAIQIINRVRDKLTGRDFSHDDTLDVPTQVELLIKQATSHENLCQCYIGW----

------------------C-----------------------PFW---------------

ND1

>H sapiens

----MPMANLLLLIVPILIAMAFLMLTERKILGYMQLRKGPNVVGPYGLLQPFADAMKLF

TKEPLKPATSTITLYITAPTLALTIALLLWTPLPMPNPLVNLNLGLLFILATSSLAVYSI

LWSGWASNSNYALIGALRAVAQTISYEITLAIILLSTLLMSGSFNLSTLITTQEHLWLLL

PSWPLAMMWFISTLAETNRTPFDLAEGESELVSGFNIEYAAGPFALFFMAEYTNIIMMNT

LTTTIFLGTTYDALSPELYTTYFVTKTLLLTSL-F--LWIRTAYPRFRYDQLMHLLWKNF

LPLTLALLMWYVSMPITISSIPPQT-

>M_brandtii

----MYFINLLAMIIPVLLAVAFLTLLERKVLGYMQLRKGPNIVGPYGLLQPIADAVKLF

TKEPLQPLTSSLVLFIIAPTLALTLALMMWVPLPMPHPLINMNLSMLFMLALSSLAVYAI

LWSGWASNSKYALIGALRAVAQTISYEVTLAIIILSILPMNGSFTLTTLITTQEYMWLMI

PSWPLAMMWFISTLAETNRAPFDLTEGESELVSGFNVEYAGGPFALFFLAEYANIIMMNA

LTIILFLGAYHSPMFSELYTINFATKALLFTMF-F--LWIRASYPRFRYDQLMHLLWKNF

LPLTLVMCMWHVTLPIILASIPPQT-

>H glaber

----MHIINTLLLLVPILLAMAYLTLMERKLLGYMQLRKGPNIVGPYGILQPIADALKLF

IKEPLKPSASSISLFIIAPMLALTLALSMWIPLPMPHPLINMNLGILFILATSSLAVYSI

LWSGWASNSKYALLGALRAVAQTISYEVSLAIILLSVLLLSGSFTLGTLMETQKYTWLIL

PTWPLAMMWFISTLAETNRAPFDLAEGESELVSGFNVEYAAGPFALFFMAEYINIIMMNA

LTTIIFINTSPMSTNPENFTMYFTIKTLMLTSL-F--LWIRASYPRFRYDQLMHLLWKNF

LPMSLAMCMWHISFPIGLFGTPPQTT

>S franciscanus MVYIFRILELVSFLVPVLLAVAFLTLVERKVLGYMQFRKGPKVVGPYGLLQPFADGLKLF

IKETLKPSTASPYLFFLSPLLFLALA----KFMPVHTPTLDLQLSLLLVLGLSRLSVYAI

LGSG-ASKSKYSLLGAIRAVAQTISYEIRLALILLSLILFSRRFNLTYIMNVQEFSFSL-

PCLPLFYI-FVSTLAETNRAPFDLTEGESEIVSGYNVEYAGGPFALFFIAEYAKIILMNL

FSVVLFLGGP--SPFKKLFPIRVIVIGIKTTCLVL--LLVRAAYPRFRYDQLMFLT-KRY

LPLSIGALCAILAL-VTLLGIS----

>S purpuratus

MVYVFSILELISFLIPILLSVAFLTLVERKVLGYMQFRNGPNVVGPFGLLQPFADGMKVF

IKEELKPVNSSPYLFFFSPLLFLALALLLWNFMPVHTPTLDLQLSLLLVLGLSSLSVYAI

LGSGWASNSKYSLLGAIRAVAQTISYEISLALILLSLIIFSSSFNLTYIMNTQEFSWFSL

SCLPLFYIWFVSTLAETNRAPFDLTEGESEIVSGYNVEYAGGPFVLFFIAEYANIILMNY

FSVVLFLGGP--SPLNNLFPISIIIVGIKTTFL-FSVLWVRAAYPRFRYDQLMFLTWKSY

LPLSIGALCAILAL-VALLGISLPLF

>L variegatus

MVYIFRLLELISFLVPVLLAVAFLTLVERKVLGYMQFRKGPKVVGPYGLLQPFADGLKLF

IKETLKPSTASPYLFFFSPLLFL-------------------QLSLLLVLGLSRLSVYAI

LGSGRASKSKYSLLGAIRAVAQTISYEI-----------FTRRFNLTYIMNVQEFSFSL-

PCLPLFYI-FVSTLAETNRAPFDLTEGESEIVSGYKVEYAGGPFALFFIAEYAKIILMNL

FSVVLFLGGP--SPFKSLFPIKVIVIGAKTTLLVF--LFVRAAYPRFRYDQLMFLT-KSY

LPLAIGALCVTIRL-VVVFGIYL---

>M musculus

----MFFINILTLLVPILIAMAFLTLVERKILGYMQLRKGPNVVGPYGILQPFADAMKLF

MKEPMRPLTTSMSLFIIAPTLSLTLALSLWVPLPMPHPLINLNLGILFILATSSLSVYSI

LWSGWASNSKYSLFGALRAVAQTISYEVTMAIILLSVLLMSGSYSLQTLITTQEHMWLLL

PAWPMAMMWFISTLAETNRAPFDLTEGESELVSGFNVEYAAGPFALFFMAEYTNIILMNA

LTTIIFLGPLYYINLPELYSTNFMMETLLLSST-F--LWIRASYPRFRYDQLMHLLWKNF

LPLTLALCMWHISLPIFTAGVPPYM-

ND2

>L variegatus

---------------GTVIVISTEK-FII-VGLELRTLALVQVLCSNFSPRKVEATIKYF

LVQA------LKGALIQAWLTGS-SVLEPLKKVSAICLRVALAFKLGLAPCHFWFPDVLQ

GLPFFQGLIIAT-QKIAPLLILFFFGQLVISYLLIVARLISILVGG-GGLNQTQVRKILA

FSSIGNMGWLIITSAYSVKAAILMLIIYLVIKTAIFLLFDFLKISTLGHLKTASQLSPTR

IALVILLMLSLGGLPPL-------------ISNGFIAFSSIMIVGSLLSLFFYLRISFKT

GLILFP-QHIIRITS-RKNAILSPLTTKV------------------------

>M musculus

MNPITLAIIYFTIFLGPMITMSSTNLMLMWVGLEFSLLAIIPMLINKKNPRSTEAATKYF

VTQATASMIILLAIVLNYKQLGTWMFQQQTNSLILNMTLMALSMKLGLAPFHFWLPEVTQ

GIPLHMGLILLTWQKIAPLSILIQIYPLLNSTIILMLAITSIFMGAWGGLNQTQMRKIMA

YSSIAHMGWMLAILPYNPSLTLLNLMIYIILTVPMFMALMLNSSMTINSISLLWNKTPAM

LTMISLMLLSLGGLPPLTGFLPKWIIITELMKNNCLIMATLMAMMALLNLFFYTRLIYST

SLTMFPTNNNSKMMTHQTKTKPNPMFSTL------AIMSTMTLPLAPQLII--

>S purpuratus

MRQIVSTFLFVTVVSGTIIVVSSENWFIIWVGLELSTLALVPILCSGFSPRNVEADNKYF

LVQASSAALLLNGALGQAWLTGSWSILDPVNEVTSICLSIALAFKIGLAPVHFWFPDVLQ

GLPFFQGLIIATWQKIAPLILMFYFSQLGFSYLLITPSLISVLIGGWGGLNQTQVRKILA

FSSIGNMGWLVITSAYSFNAAIIMLVIYLIINTSLFLLFDHLKVSTLGHLNTISQLSPIS

VALVLLVMLSLGGLPPLTGFILKFTSLYFLVANNFIILSSIMIIGNLQDYFFYLRISFNT

SLFLFP-QHIISSASWRNSTIISPLAPKAWLSSVSTVLSTLAIPLTLPLYIIT

>S franciscanus

MHRIVSTFLFITVVSGTIIVISSKKLFVI-VGLELSTLALVPILCSRFSPRKVEATIKYF

------------------ALTGSWSILTPVKKVTSICLSIALAFKIGLAPCHF-FPDVLQ

GLPFFQGLIIAT-QKIAPLVLLFYFRQLVFSYLLIAARLLSILIGG-GGLNQTQVRKILA

FSSIGNMG-LVVTSAYSLNAAIVMLVIYLTINTSLFLLFDHLGVSTLGHLKTTSQLSPIR

VALVLLVMLSLGGLPPLTGFILKFTSLYFLIANNFIILASIMIVGRLLRLFFYLRISFKT

RLILFP-QHIISIAS-RNRAIVSPLTPKT-LISVSAVLSTLAIPLTLPLYIIT

>H glaber

MNLISNSVIYLTLISGTLITLTSSHWLLMWAGLEMSMFAMIPILTHKTSPRSTEAATKYF

LVQATASMMFMMAIIYSVALSGQWAIMHSQNQTISSILMLSLMMKLGLAPFHLWVPEVTQ

GANLWSGMILLTWQKIAPLSIMYQTYSSANQTMIISFSLLSILLGGWGGLNQTQLRKILA

YSSISHMGWMTTVMCFNPSIMHLNLLIYIMLTLSMFSMLSINTNTTTLSLSHLWNISPIL

ANTILIILLSLGGLPPLTGFMPKWMIITEMTKNQSIILPTLMAMMALLNLFFYMRLAYST

SLTLFPSTNNLKMKWYTQNTKMPYLLPPL------IIVSTLMLPLSPMMTTLE

>H sapiens

MNPLAQPVIYSTIFAGTLITALSSHWFFTWVGLEMNMLAFIPVLTKKMNPRSTEAAIKYF

LTQATASMILLMAILFNNMLSGQWTMTNTTNQYSSLMIMMAMAMKLGMAPFHFWVPEVTQ

GTPLTSGLLLLTWQKLAPISIMYQISPSLNVSLLLTLSILSIMAGSWGGLNQTQLRKILA

YSSITHMGWMMAVLPYNPNMTILNLTIYIILTTTAFLLLNLNSSTTTLLLSRTWNKLTWL

TPLIPSTLLSLGGLPPLTGFLPKWAIIEEFTKNNSLIIPTIMATITLLNLYFYLRLIYST

SITLLPMSNNVKMKWQFEHTKPTPFLPTL------IALTTLLLPISPFMLMIL

>SIG

------------------------------------------------------------

------------------------------------------------------------

-----------------------------------+------------------------

----------------------------------------------------------+-

------------------------------------------------------------

ND3

>H sapiens

-MNFALILMINTLLALLLMIITFWLPQLNGYMEKSTPYECGFDPMSPARVPFSMKFFLVA

ITFLLFDLEIALLLPLPWALQTTNLPLMVMSSLLLIIILALSLAYEWLQKGLDWTE

>H_glaber

-MNMTISLLMNFSLASILMLIAFWLPQMDAYAEKLSPYECGFDPMGSARLPFSMKFFLIA

ITFLLFDLEIALLLPLPWASQTNNLTLMLTVSLMLISVLALGLFYEWSQKGLEWDK

>S franciscanus

MTTIIFLFGITIAVATVFGLAAHALPRRAGDKEKNSPYECGFDPLNSARLPFSFRFFLVA

ILFLLFDLEIALLFPLPAAELITNPSTLIPISLIFMIILALGLVFE-VKGGLE-AE

>S_purpuratus

MTTIIFLFSITIAVAVVLGLAAHALPNRTSDSEKSSPYECGFDPLNSARLPFSFRFFLVA

ILFLLFDLEIALLFPLPAASLITPPSTLIPISMVFMVILTLGLVFEWINGGLEWAE

>L variegatus

MTPIIFIFLITIVVAIVFAAAAHILPKRSKDREKTSPYECGFDPLNSARLPFSFRFFLVA

ILFLLFDLEIALLFPLPAAKSSTPPSILIPISLIFMIILALGLVFEWIKGGLE---

>M musculus

-MNLYTVIFINILLSLTLILVAFWLPQMNLYSEKANPYECGFDPTSSARLPFSMKFFLVA

ITFLLFDLEIALLLPLPWAIQTIKTSTMMIMAFILVTILSLGLAYEWTQKGLEWTE

>SIG

------------+--+--------------------------------------------

ND4

>S_purpuratus

MITLILFTVGMATTTLLIPSNKLWAGAIFQSALLSLLSLIVLNNHWTASWHNLSSILASD

TISAPLIILSCWLAPIALIASKGQLNNSSDLGSRVFIIMIIVITGALIITFSSLELILFY

IVVETTLIPTLILITRWGAQMERCQAGLYFMFYTLFGSLPLLIAIIAIYISSSSLSIPNV

NLLWANDGSIESLTMWWALSINCFFNNLPVYGFHLWLPKAHVEAPVAGSMILAAILLKIG

GYGLMRLIALFSTISMNALSLALIVFCTWGALITSVICVRQTDLKALIAYSSVGHMSIVA

AAIFSETSWGMNGALMLMVAHGLVSSALFSLANTVYERSGTRTLAITRGLKLLLPLSTLW

WLLMCAANLGLPPSPNLIGEILILSSLISWSVWLFPIVGFAQVFGAIYSLMIFQLSQQGT

PFTSIINVFSSFSREHLFAALHILPLILIMINPFSALIAWLK

>L variegatus

MITLILFTTGIAITNFLVPRNKVWVSSIAQRAALSLLSLIVLTNHWT-SSHKLSSTLASD

TISAPLIILRCWLCPIALIARKGHLKKARSLNQRMFISLVIIITGALIITFSSLELLLFY

VAFETTLIPTLILITRWGAQMERFQAGLYFMFYTLFGSLPL------------------V

ELIWT-EKSINSLTM--ALSIIAFLIKMPIYGFHL-LPKAHVEAPVAGSMILAAILLKLG

GYGLTRLISLFSTISLKYLSLVLIVFCS-GALVTRIICIRQTDLKALIAYSSVGHMRVVA

AAIFSGTKWGLKGALMLMIAHGLVSSALFSMAKTVYERRGTRTLAITRGLKMLLPLRTLW

WLLMCAAKLGLPPSPNLIGEILILSSLIAWSVWLFPILGFATVFGAIYSLMIFQMSNQGT

PSRKVAKIAPSFSREHLLARLHILPLVLIIINPMSTLISLK-

>S franciscanus

----ILFTVGIATTTFLVPGNKLWARAIFQTALLSLLSLIVLKNHWT-SSHKLSTILASD

TISAPLIILRC-LAPIALIARKGHLKTARDLNQRVFITMVIIITGALIVTFSSLELLLFY

IAFETTL-----------------------------------------------------

-----TERSVESLTI--SLSIIAFLIKMPVYGFHLWLPKAHVEAPVAGSMILAAILLKLG

GYGLMRLISLFSTISMKALSLALIVFCS-GALVTSIICVRQTDLKALIAYSSVGHMRIVA

AAIFSETKWGMKGALMLMVAHGLVSSALFSLAKTVYERRGTRTLAITRGLKLLLPLRTFW

-----------------------------ISSILFPIVGFATVFGAVYSLMIFQLSQQGT

PSTGIMKIPPSFSREHLLAALHILPLILI-------------

>M musculus

MLKIILPSLMLLPLTWLSSPKKTWTNVTSYSFLISLTSLTLLWQTDE-NYKNFSNMFSSD

PLSTPLIILTAWLLPLMLMASQNHLKKDNNVLQKLYISMLISLQILLIMTFSATELIMFY

ILFEATLIPTLIIITRWGNQTERLNAGIYFLFYTLIGSIPLLIALILIQKHVGTLNLMIL

SFTTHTLDTSWSNNLLWLACMMAFLIKMPLYGVHLWLPKAHVEAPIAGSMILAAILLKLG

SYGMIRISIILDPLT-KYMAYPFILLSLWGMIMTSSICLRQTDLKSLIAYSSVSHMALVI

ASIMIQTPWSFMGATMLMIAHGLTSSLLFCLANSNYERIHSRTMIMARGLQMVFPLMATW

WLMASLANLALPPSINLMGELFITMSLFSWSNFTIILMGLNIIITGMYSMYMIITTQRGK

LTNHMINLQPSHTRELTLMALHMIPLILLTASP-KLITGLTM

>H sapiens

MLKLIVPTIMLLPLTWLSKKHMIWINTTTHSLIISIIPLLFFNQINN-NLFSCSPTFSSD

PLTTPLLMLTTWLLPLTIMASQRHLSSEPLSRKKLYLSMLISLQISLIMTFTATELIMFY

IFFETTLIPTLAIITRWGNQPERLNAGTYFLFYTLVGSLPLLIALIYTHNTLGSLNILLL

TLTAQELSNSWANNLMWLAYTMAFMVKMPLYGLHLWLPKAHVEAPIAGSMVLAAVLLKLG

GYGMMRLTLILNPLT-KHMAYPFLVLSLWGMIMTSSICLRQTDLKSLIAYSSISHMALVV

TAILIQTPWSFTGAVILMIAHGLTSSLLFCLANSNYERTHSRIMILSQGLQTLLPLMAFW

WLLASLANLALPPTINLLGELSVLVTTFSWSNITLLLTGLNMLVTALYSLYMFTTTQWGS

LTHHINNMKPSFTRENTLMFMHLSPILLLSLNP-DIITGFSS

>H_glaber

MLKIILPTIMLIPVTWLSKNSLLWINTSCHSLLIALISLPTLNKLDI-NSHNFSTNFFSD

SLSSPLVNLTVWLLPLMIIASQHHMKNEPAVRKKLYLSLLITLQALLIMTFTTSEMMMFY

ILFEATLIPTLIIITRWGNQAERMKAGLYFLFYTLTGSLPLLVALTYLKNYLGSLNFMLF

SYKASPLLPTWSNSMLWLACIMAFMVKFPLYGLHLWLPKAHVEAPIAGSMVLAAILLKLG

GYGMMRISQLLEPLT-TSMSYPFILLSLWGMVMTSSICLRQTDLKSLIAYSSISHMALVI

TAILIQTPWSYMGATTLMIAHGLTSSLLFCLANSNYERTHSRTMILARGLQTILPLMALW

WLLASFTNLALPPSINLVGELLIITSTFSWSNFTIILTGMNVLITALYTLYMLISTQRGT

PPHHIINLPPSFTRENTLMTLHLLPLLLMTLNP-KIIMGNLF

ND4L

>H sapiens

-MTPMRKTNPLMKLINHSFIDLPTPFNISAWWNFGSLLGACLILQITTGLFLAMHYSPDA

STAFSSIAHITRDVNYGWIIRYLHANGASMFFICLFLHIGRGLYYGSFLYSETWNIGIIL

LLATMATAFMGYVLPWGQMSFWGATVITNLLSAIPYIGTDLVQWIWGGYSVDSPTLTRFF

TFHFILPFIIAALATLHLLFLHETGSNNPLGITSHSDKITFHPYYTIKDALGLLLFLLSL

MTLTLFSPDLLGDPDNYTLANPLNTPPHIKPEWYFLFAYTILRSVPNKLGGVLALLLSIL

ILAMIPILHMSKQQSMMFRPLSQSLYWLLAADLLILTWIGGQPVSYPFTIIGQVASVLYF

TTILILMPTISLIENKMLKWA

>M_brandtii

-MTNIRKSHPLMKIINSSFIDLPAPSNISSWWNFGSLLGICLALQILTGLFLAMHYTSDT

TTAFNSVTHICRDVNYGWVLRYLHANGASMFFICLYLHVGRGLYYGSYMYTETWNIGVIL

LFAVMATAFMGYVLPWGQMSFWGATVITNLLSAIPYIGTDLVEWIWGGFSVDKATLTRFF

AFHFLLPFIIAAMVMVHLLFLHETGSNNPTGIPSNADMIPFHPYYTIKDILGLLLMITVL

LMLVLFSPDLLGDPDNYTPANPLNTPPHIKPEWYFLFAYAILRSIPNKLGGVLALVLSIL

ILIIIPLLHTSKQRSMTFRPLSQCLFWLLTADLFTLTWIGGQPVEHPYVIIGQLASILYF

SIIIILMPLISLMENHLLKW-

>H_glaber

-MTNIRKSHPLIKIINHSFIDLPTPSSISYWWNFGSLLGACLILQIITGLFLSMHYTADT

ATAFSSVAHICRDVNYGWLIRYLHANGASMFFICLYLHVGRGMYYGSYMFMETWNIGIIL

LLSVMATAFMGYVLPWGQMSFWGATVITNLFSAIPYIGPTLVEWIWGGFAVDKATLTRFF

AFHFILPFIITALTMVHLLFLHETGSNNPSGINSDSDKIPFHPYYSFKDFMGLQIMLLIL

LTLTLFHPDLLGDPDNYTPANPMSTPPHIKPEWYFLFAYAILRSIPNKLGGVLALVMSIL

ILVALPLLHTSKQRSMMFRPISQCLFWTFISTLLTLTWIGSQPVEYPYIIIGQLASILYF

LIILVLMPLAGLVENKMMK--

>S franciscanus

MAAPLRKEHPIFRILKSTFVDLPLPSNLSI-WNFGSLLGLCLIVQILTGIFLAMHYTADI

TLAFSSVRHICRDVKYGWLLRKVHAKGASLFFICMYCHIGRGLYYGSYKKIET-KVGVIL

FLVTILTAFMGYVLV-GQMSF-AATVITNLVSAIPYMGTTIVQ-----------------

-LHFLFPFIIAALAIIHLVFLHNRGANNPVGLKSNYDKAPFHIYYTTKDTVGFILLIAAL

FSLALLFPGALKDPENFIPANPLVTPPHIQPE-YFLFAYAILRSIPNKLGGVIALVAAIL

VLFLMPLLNTSKKESNSFRPLSQAAF-LLVATFLILT-IGRQPVEYPYVLLGQVASVIYF

SLFIFGFPLVSSIENKIIFS-

>S_purpuratus

MAAPLRKEHPIFRILNSTFVDLPLPSNLSIWWNSGSLLGLCLVVQILTGIFLAMHYTADI

TLAFSSVMHILRDVNYGWFLRYVHANGVSLFFICMYCHIGRGLYYGSYNKIETWNVGVIL

FLVTILTAFMGYVLVWGQMSFWAATVITNLVSAIPYIGTIIVQWLWGGFSVDNATLTRFF

PFHFLFPFIIAALAVIHLVFLHNSGANNPFAFNSNYDKAPFHIYFTTKDTVGFILLVAAL

FSLALLFPGALNDPENFIPANPLVTPPHIQPEWYFLFAYAILRSIPNKLGGVIALVAAIL

VLFLMPLLNTSKNESNSFRPLSQAAFWLLVAHLFILTWIGSQPVEYPYVLLGQVASVLYF

SLFIFGFPIVSSIENKIIFS-

>L variegatus

MAAPIRKEHPIFRILKGTFVDLPLPSNLSI-WKFGSLLGLCLIVQILTGLFLAMHYTADI

TLAFSSVRHICRDVNYG-LLRKVHAKGASLFFICMYCHIGRGLYYGSYKKVET-NIGVIL

FLVTILTAFMGYVLV-GQMSFWAATVITNLVSAIPYIGTTIVQ--LGGFSVDKATLTRFF

AFHFLFPFIIAALAVIHLVFLHKRGANNPVGLKRNYDKAPFHIYYTTKDTVGFILLITAL

FSLALLFPGALKDPENFIPANPLVTPPHIQPE-YFLFAYAILRSIPNKLGGVIALVAAIL

VLFLMPLLNTSKKESNSFRPLSQAAF-LLVATFFILT-IGAQPVEYPYVFLGQVASVLYF

SLFIFGFPLVSSLEKKIIFS-

>M musculus

-MTNMRKTHPLFKIINHSFIDLPTPSNISSWWNFGSLLGICLMVQIITGLFLAMHYTSDT

MTAFSSVTHICRDVNYGWLIRYMHANGASMFFICLFLHVGRGLYYGSYTFMETWNIGVLL

LFAVMATAFMGYVLPWGQMSFWGATVITNLLSAIPYIGTTLVEWIWGGFSVDKATLTRFF

AFHFILPFIIAALAIVHLLFLHETGSNNPTGLNSNADKIPFHPYYTIKDILGILIMFLIL

MTLVLFFPDMLGDPDNYMPANPLNTPPHIKPEWYFLFAYAILRSIPNKLGGVLALVLSIL

ILALMPFLHTSKQRSLMFRPITQILYWILVANLLILTWIGGQPVEHPFIIIGQLASISYF

SIILILMPISGIIEDKMLKLY

ND5

>H sapiens

MTMHTTMTTLTLTSLIPPILTTLVNP--------------------------------NK

KNSYPHYVKSIVASTFIISLFPTTMFMCLDQEVIISNWHWATTQTTQLSLSFKLDYFSMM

FIPVALFVTWSIMEFSLWYMNSDPNINQFFKYLLIFLITMLILVTANNLFQLFIGWEGVG

IMSFLLISWWYARADANTAAIQAILYNRIGDIGFILALAWFILHSNSWDPQQMALLNANP

-SLTP--LLGLLLAAAGKSAQLGLHPWLPSAMEGPTPVSALLHSSTMVVAGIFLLIRFHP

LAENSPLIQTLTLCLGAITTLFAAVCALTQNDIKKIVAFSTSSQLGLMMVTIGINQPHLA

FLHICTHAFFKAMLFMCSGSIIHNLNNEQDIRKMGGLLKTMPLTSTSLTIGSLALAGMPF

LTGFYSKDHIIETANMSYTNAWALSITLIATSLTSAYSTRMILLTLTGQPRFPTLTNINE

NNPTLLNPIKRLAAGSLFAGFLITNNISPASPFQTTIPLYLKLTALAVTFLGLLTALDLN

YLTNKLKMKSPLCTFYFSNMLGFYPSITHRTIPYLGLLTSQNLPLLLLDLTWLEKLLPKT

ISQHQISTSIIT-STQKGMIKLYFLSFFFPLVLTLLLIT----

>H_glaber

MNLYISSFIMSLLLLTTPIPLSSINF--------------------------------PK

NTSYPSYVKSIISLSFFISLIPTTMLLLSNQEAVISNWNWMHIQTVSINMSFKLDYFSVM

FMSVALFVTWSIMEFSMWYMHSDPDMNKFIKYLLLFLITMMILVTANNAMQLFIGWEGVG

IMSFLLIGWWLGRSDANTAALQAILYNRIGDIGFIISMVWFMTNLSSWEMQQLLIMQDEM

-NYIP--LLGLLLAATGKSAQFGLHPWLPSAMEGPTPVSALLHSSTMVVAGIFLMIRFYP

LIQHNQTILTMSMCLGATTTLFTALCALTQNDIKKIIAFSTSSQLGLMMVTIGINQPHLA

FLHICMHAFFKAMLFMCAGSIIHNLNNEQDIRKMGGLLKMLPFTSSSLIIGSLALTGMPF

LTGFYSKDLIIESANSSYINAWALLITLISTSLTAVYSSRIVFYALLDHPRSTTTLNINE

NNPLLINPIKRLAMGSILAGFIISNNIPPTSIPQMTMPTHIKLSTLMVTALGFMTAMELI

IMSHYLKNKYPNKLFNFSNMLGYFTPTAHRFMPYKKLMMSQNLASSSMDAIWLEKSAPKN

VSDIQYTASMFT-SNQTGLIKLYFLSFLLSFMVALILST----

>S franciscanus

------------------------------------------------------------

-------------------------INTEFSDINITLSLWLNNTPTNISLNFIYDQYFLV

FLSIALIVTWSIMEFSFYYMAEDPNSSAFFRLLTIFLLNMLILTCSNSLFLVFLGWEGVG

FLSFLLISWWTTRNDASSSALEAVIYNRIGDIGLITFMALSALSFNSWNLTEILSSSEDL

TPFLPFLLFGLVLAAAGKSAQFGLHPWLPAAMEGPTPVSALLHS----------------

------------------------------------------------------------

------------------------------------------------------------

------------------------------------------------------------

------------------------------------------------------------

------------------------------------------------------------

-------------------------------------------

>S_purpuratus

MVISPSLIISSLNLGILTILLGSIFFFSKSYFSKGNVNFPLIKTTSACLSVNNDKEETIE

YNSGPFAMAILKVLAFLSVLSLLVTCNNSIQSINITLSLWLSNTPLNISLNFIYDQYFLV

FLSVALIVTWSIMEFSFYYMTEDPNSSAFFRLLTIFLLNMLILTCSNSLFLIFLGWEGGG

FLSFLLISWWTTRNDASSSALEAVITNRIGNIGLITFMALSALNFNSSNLTNILSSNENL

TPLLPFLLFGLILAAAGKSAQFGLHPWLPALLEGPTPVSALLHSSTMVVAGVFLLVRTSE

LFSSPLITHSLVLILGGTTALFAASTAIAQHDIKKIIAYSTTSQLGLMVTAIGIGQPALA

FFHICTHAFFKAMLFLCSGSVIHSLSDEQDLRKMGGLSKLLPVTSSCLILGSLALMA-PL

LAGFYSKDLILEATSASVLNLLGIVLSIVATMLTAVYSFRIIFFCFSLSPSCSSPFSHSE

ENFNLNNALLRLATGTIASGWFFSNLL--FAPPSFNVTSLAKGTPLIVPIIG--VAALFM

SLISSTSNSIGSNAHSATTSQWFFVDAVHLSIITMSLALS-FFSSRTLDRGWQENIGPQG

IAPTSTALSKISQAGQIGLIKRYILSSMASVLVILALSLLILS

>S franciscanus

------------------------------------------------------------

------------------------------------------------------------

--------------------------------------------------------EGVG

FLSFLLIS-WTTRKDARSSELEAVIYKRIGDIGLITFMALSA------------------

-------LFGLVLAAAGKSAQFGLHPWLPAAMEGPTPVSALLHRSTMVVAGVFLLVRTRD

LFSLSPNAQSIVLILGGTTALFAASTAIAQHDIKKIIAYSTTRQLGLMVTAIGIGQPILA

FFHICTHAFFKAMLFLCSGRVIHRLRDEQDLRKMGGLRKLLPVTSACLILGRLALMGTP-

------------------------------------------------------------

------------------------------------------------------------

------------------------------------------------------------

-------------------------------------------

>L variegatus

------------------------------------------------------------

---------------------------------------LLAKTPTKISLNFIYDQYFLL

FLTVALIVT-SIMEFSFYYMADDPKGTSFFRLLTIFLLKMLLLTCSKRLFLIFLG-EGVG

FLSFLLIR-WTTRKDARRSALEAVIYNRIGDIGLITFMALSILTLNS-NLTEI-------

-SFLPFLLFGLILAAAGKSAQFGLHP-LPAAMEGPTPVSALLHRSTMVVAGVFLLVRTRD

LFVLSPRTQSVVLILGGITALFAASTAIAQHDIKKIIAYSTTRQLGLMVSSIGIGQPILA

FFHICTHAFFKAMLFLCSGRIIHRLSDEQDLRKMGGLRKLLPVTSACLALGRLAL---PF

LAGFYSKDLILEAARASFLNTRGLSLSIVATMLTAVYRFRIVYFCT--------------

------------------------------------------------------------

-----------------------------------SFISSLFLSSRTLDRG-QEEIGPQG

IAQSSTFLSKANQRRQIGLIKRYILSSIISI------------

>M musculus

MNIFTTSILLIFVLLLSPILISMSNL--------------------------------IK

YINFPLYTTTSIKFSFIISLLPLLMFFYNNMEYMITTWHWVTMNSMELKMSFKTDFFSIL

FTSVALFVTWSIMQFSSWYMHSDPNINRFIKYLTLFLITMLILTSANNMFQLFIGWEGVG

IMSFLLIGWWYGRTDANTAALQAILYNRIGDIGFILAMVWFSLNMNSWELQQIMFSNNND

-NLIP--LMGLLIAATGKSAQFGLHPWLPSAMEGPTPVSALLHSSTMVVAGIFLLVRFHP

LTTNNNLILTTMLCLGALTTLFAAICALTQNDIKKIIAFSTSSQLGLMMVTLGMNQPHLA

FLHICTHAFFKAMLFMCSGSIIHSLADEQDIRKMGNITKTMPFTSSCLVIGSLALTGMPF

LTGFYSKDLIIEAINTCNTNAWALLITLIATSMTAMYSMRIIYFVTMTKPRFPPLISINE

NDPDLMNPIKRLAFGSIFAGFVISYNIPPTNIPILTMPWFLKTTALIISVLGFLIALELN

NLTMKLSMSKPNPYSSFSTLLGFFPSIIHRITPMKSLNLSLKTSLTLLDLIWLEKTIPKS

TSTLHTNMTTLT-TNQKGLIKLYFMSFLINIILIIILYSINLE

>SIG

------------------------------------------------------------

------------------------------------------------------------

------------------------------------------------------------

------------------------------------------------------------

------------------------------------------------------------

-----------------+------------------------------------------

------------------------------------------------------------

------------------------------------------------------------

------------------------------------------------------------

------------------------------------------------------------

p66Shc

>H sapiens

MDLLPPKPKYNPLRNESLSSLEEGASGSTPPEELPSPSASSLGPILPPLPGDDSPTTLCS

FFPRMSNLRLANPAGGRPGSKGEPGRAADDGEGIDGAAMPESGPLPLLQDMNKLSGGGGR

RTRVEGGQLGGEEWTRHGSFVNKPTRGWLHPNDKVMG-PGVSYLVRYMGCVEVLQSMRAL

DFNTRTQVTREAISLVCEAVPGAKGATRRRKPCSRPLSSILGRS-NLKFAGMPITLTVST

SSLNLMAADCKQIIANHHMQSISFASGGDPDTAEYVAYVAKDPVNQRACHILECPEGLAQ

DVISTIGQAFELRFKQYLRNPPKLVTPHDRMAGFDGSAWDEEEEEPPDHQYYNDFPGKEP

PLGGVVDMRLREGAAPGAARPTAPNAQTPSHLGATLPVGQPVGGDPEVRKQMPPPPPCPG

RELFDDPSYVNVQNLDKARQAVGGAGPPNPAINGSAPRDLFDMKPFEDALRVPPPPQSVS

MAEQLRGEPWFHGKLSRREAEALLQLNGDFLVRESTTTPGQYVLTGLQSGQPKHLLLVDP

EGVVRTKDHRFESVSHLISYHMDNHLPIISAGSELCLQQPVERKL

>H glaber

MDLLPPKPKYNPLRNESLSSLEEGASGSSPPEELPSPSASSLGPILPPLPSDDSPTTLCS

FFPRMSNLKLANPAGGRLGPKGEPGRAAEDGEGITGTAVLDSGPLPLLQDMNKLSGGSGR

RTRVEGGQLGGEEWTRHGSFVNKPTRGWLHPNDKVMG-PGVSYLVRYMGCVEVLQSMRAL

DFNTRTQVTREAISLVCEAVPGAKGATRRRKPCSRPLSSVLGRS-NLKFAGMPITLTVST

SSLNLMAADCKQIIANHHMQSISFASGGDPDTAEYVAYVAKDPVNQRACHILECPEGLAQ

DVISTIGQAFELRFKQYLRNPPKLVTPHDRMAGFDGSAWDEEEEEPSDHQYYNDFPGKEP

PLGGVVDMRLREGATPGAARPTPPSAQTPSHLGATLPVGQPIAGDPEVRKQMPPPPPCPG

RELFDDPSYVNVQNLDKARQAGGGAGPPNPAVNGSAPRDLFDMKPFEDALRVPPPPQLMS

MAEQLRGEPWFHGKQSRREAEALLQLNGDFLVRESTTTPGQYVLTGLQSGQPKHLLLVDP

EGVVRTKDHRFESVSHLISYHMDNHLPIISAGSELCLQQPVERKL

>M brandtii

MDLLPPKPKYNPLRNESLSSLEEGASGSSPPEELPSPSASSLGPILPPQAGDDSPTTLCS

FFPRMSNLKLANPAGARPGPKGEPGRVAEDGEGTAGAAVPDSGPLPLLQDMNKLSGGGGR

RTRVEGGQLGGEEWTRHGSFVNKPTRGWLHPNDKVMG-PGVSYLVRYMGCVEVLQSMRAL

DFNTRTQVTREAISLVCEAVPGAKGASRRRKPCSRPLSSILGRS-NLKFAGMPITLTVST

SSLNLMAADCKQIIANHHMQSISFASGGDPDTAEYVAYVAKDPVNQRACHILECPEGLAQ

DVISTIGQAFELRFKQYLRNPPKLVTPHDRMAGFDGSAWDEEEEEPPDHQYYNDFPGKEP

PLGGVVDMRLREGAPQGPARPTLPSAQSPSHLGATLPVGQPAGGDPEVHKQMPPLPHCPG

RELFDDPSYVNIQNLDKARQAGGGAGPPNLAVNGSAPRDLFDMKPFEDALRVPPPPQSVA

MAEQLRGEPWFHGKLNRREAEALLQLNGDFLVRESTTTPGQYVLTGLQSGQPKHLLLVDP

EGVVRTKDHRFESVSHLISYHMDNHLPIISAGSELCLQQPVERKL

>S franciscanus

------------------------------------------------------------

------------------------------------------------------------

-------------------FCLL----FLS------------------------------

------------------------------------------------------------

-----------------------------------------------ACHVLECLAGLAQ

DVITTVGQAFEIRFKDYLRNPPQVFSTPDR------------------------------

------------------------------------------------------------

------------------------------------------------------------

------------------------------------------------------------

---------------------------------------------

>S purpuratus

------------------------------------------------------------

------------------------------MAGVMSHFKKNKGP----------------

--NMVGQRSNEELWTRTGTFINKPTRGWLHIEEELKDHGGVCYGVRYVGCVEVKQSMRTL

PFEMRGQVTKEAIFRICDA---AGFKYTRKKKASKTICKIIGENPNILFGGANVNLTIST

ASINIRIKETGKPIANHQMQGISFASGGDAN----------------ACHVLECLAGLAQ

DVITTVGQAFEIRFKDYLRNPPQVFSTPDR------------------------------

------------------------------------------------------------

------------------------------------------------------------

------------------------------------------------------------

---------------------------------------------

>L variegatus

LQMMHNTSLFNEIM----------------------------------------------

------------------------------------------------------------

--RIHASAYYINHWSFEKAVCNE----FLPCLELLYGYSFIDIWIYLHSYLTLTPIFYIV

QQRIRCKHPH-------------KYGIKLHEQASKTICKIVGENPNILFGGANVNLTIST

ASINIRIKETGKV-----KPSIIYFCCNSSSRIDC------------ACHVLECLAGLAQ

DVITTVGQAFEIRFKDYLRNPPQVFSTPDR------------------------------

------------------------------------------------------------

------------------------------------------------------------

------------------------------------------------------------

---------------------------------------------

>M musculus

MDLLPPKPKYNPLRNESLSSLEEGASGSTPPEELPSPSASSLGPILPPLPGDDSPTTLCS

FFPRMSNLKLANPAGGRLGPKGEPGKAAEDGEGSAGAALRDSGLLPLLQDMNKLSGGGGR

RTRVEGGQLGGEEWTRHGSFVNKPTRGWLHPNDKVMG-PGVSYLVRYMGCVEVLQSMRAL

DFNTRTQVTREAISLVCEAVPGAKGATRRRKPCSRPLSSILGRS-NLKFAGMPITLTVST

SSLNLMAADCKQIIANHHMQSISFASGGDPDTAEYVAYVAKDPVNQRACHILECPEGLAQ

DVISTIGQAFELRFKQYLRNPPKLVTPHDRMAGFDGSAWDEEEEEPPDHQYYNDFPGKEP

PLGGVVDMRLREGA----ARPTLPSAQMSSHLGATLPIGQHAAGDHEVRKQMLPPPPCPG

RELFDDPSYVNIQNLDKARQAGGGAGPPNPSLNGSAPRDLFDMKPFEDALRVPPPPQSMS

MAEQLQGEPWFHGKLSRREAEALLQLNGDFLVRESTTTPGQYVLTGLQSGQPKHLLLVDP

EGVVRTKDHRFESVSHLISYHMDNHLPIISAGSELCLQQPVDRKV

PDK1

>H sapiens

------------------------------------------------------------

MARTTSQLYDAVPIQ-------SSVVLCSCPSPSMVRT-QTESSTPPGIP-GGSRQGPAM

DGTAAEPRPGAGSL-QH-AQPPPQPRKKRPEDFKFGKILGEGSFSTVVLARELATSREYA

IKILEKRHIIKENKVPYVTRERDVMSRLDHPFFVKLYFTFQDDEKLYFGLSYAKNGELLK

YIRKIGSFDETCTRFYTAEIVSALEYLHGKGIIHRDLKPENILLNEDMHIQITDFGTAKV

LSPESK---QARANSFVGTAQYVSPELLTEKSACKSSDLWALGCIIYQLVAGLPPFRAGN

EYLIFQKIIKLEYD---FPEKFFPKARDLVEKLLV-----LDATKRLGCEEMEGYGPLKA

HPFFESVTWENLHQQTPPKLTAYLPAMSEDDEDCYGNYDNLLSQFGCMQVSSSSSSHSLS

ASDTGLPQRSGSNIEQYIHDLDSNSFELDLQFSEDEKRLLLEKQAGGNPWHQFVENNLIL

KMGPVDKRKGLFARRRQLLLTEGPHLYYVDPVNKVLKGEIPWSQELRPEAKN--------

------FKTF--FVHTPNRTYYLMDPSGNAHKWCRKIQEVWRQRYQSHPDAAVQ

>H_glaber

------------------------------------------------------------

MARTTSQLYDTVPIQ-------PSVVLCACPSPSMVRT-QTESGTAPSVPSGGSRQGPTM

DGTTAESRPSANPL-QHAAQPPPQPRKKRPEDFKFGKILGEGSFSTVVLARELATSREYA

IKILEKRHIIKENKVPYVTRERDVMSRLDHPFFIKLYFTFQDDEKLYFGLSYAKNGELLK

YIRKIGSFDETCTRFYTAEIVSALEYLHGKGIIHRDLKPENILLNEDMHIQITDFGTAKV

LSPESK---QARANSFVGTAQYVSPELLTEKSACKSSDLWALGCIIYQLVAGLPPFRAGN

EYLIFQKIIKLEYD---FPEKFFPKARDLVEKLLV-----LDATKRLGCEEMKGYEPLKA

HPFFESITWKNLHQQTPPKLTAYLPAMSEDDEDCYGNYDNLLSQFGCMQVSSSSSSHSLS

VVDASLPHRSDSNIEQYIHDLDTNSFELDLQFSEDEKRLLLEKQAGGNPWHQFVENNLIL

KMGPVDKRKGLFARRRQLLLTEGPHLYYVDPVNKVLKGEIPWSQELRPEAKN--------

------FKTF--FVHTPNRTYYLMDPSGNAHKWCRKIQEVWRQRYQSHPDAAVQ

>M_brandtii

MRFYPSMNPANHRSTPGAEGKQSTAGPLQMVEGGLVGLCGAAAEWSQKNLDPECTWHGGQ

KAEAPRRQYDTIPIQ-------SSVVLCSCPSPSMVRT-QTDSGSAP-VPSGGSRQGPTM

DSTAAESRPSANSL-QHAAQPPPQPRKKRPEDFKFGKILGEGSFSTVVLARELATSREYA

IKILEKRHIIKENKVPYVTRERDVMSRLDHPFFVKLYFTFQDDEKLYFGLSYAKNGELLK

YIRKIGSFDETCTRFYTAEMVSALEYLHGRGIIHRDLKPENILLNEDMHIQITDFGTAKV

LSPETK---QARANSFVGTAQYVSPELLTEKSACKSSDLWALGCIIYQLVAGLPPFRAGN

EYLIFQKIIKLEYD---FPEKFFPKARDLVEKLLV-----LDATKRLGCEEMEGYGPLKA

HSFFDSITWENLHHQTPPKLTAYLPAMSEDDEDCYGNYDNLLSQFGCMQVSSSSSSHSLS

ASDTGLPQRAGSNIEQYIHDLDTNSFELDLQFSEDEKRLLLEKQASGNPWHQFVENNLIL

KMGPVDKRKGLFARRRQLLLTEGPHLYYVDPVNKVLKGEIPWSQELRPEAKN--------

------FKTF--FVHTPNRTYYLMDPSGNAHKWCKKIQEVWRHRYQSHPDTTVQ

>S franciscanus

----------------------------------------------------------RH

KERYEPRVKTYNPQAYKFYMEQHVENILKSHRQRITRRMQLEQEMQRVDLTSGDRDQMRR

MLFQKESNYLRLKR-----------AKMNKSMFKKIKTLGIGAFGQVAIAQKRDTKAMYA

MKYMRKAMCIQKDQARHVKAEQEILAAAEHPWLVKLYYSFQDSDKLCFVMEYVNGGDLFY

HLSREGVFDEERTRFYTAEIVEALAYLHTHGIVYRDLKPENILLDNKGHVKIADFGRAKE

FDSLNDIL-IVNIIEFQGTPLYMAPELVEEKPYDHTADLWSLGCILYELFVGTPPFYTNS

IFQLVSLIIKVRNTVICFHLVF------MIAFLLV-----IDPSKRLGCDECGGYPALKA

HPFLEGVDWENLHEQKAP---VLKPCTVVDGEEIHSEYY--VDDFD-LELNPTSLSEYLK

RIETHDRPHSPTRDNKYVVD-----------LTPAERKQRLERQQKENKWHRFVEGNLIL

KNGWVDKRK------------PG----YIAPESYPRPGEIGGDDKDPGSHLQDIVAMSMP

LDDSHYISCAKRLTVQPNRTYYLIDPESDAVEWCQAIKEMYRHTYQKSI-----

>S_purpuratus

-----------------------------------------------------------M

ASVNSAEFSECKPII-------HSPVSRSCHQEAMSKHKSTDSQPISSSGNSGSSSKASN

NQFASSPRSATSPS---PSPSSTSILKKKRADFRFGTILGEGSYSEVVLATEIATGNQYA

IKVLEKRHIIREKKEKYVLREKEVLSRLDHPFFVRLYFTFQDNYKLYFGLSLAKRGELLP

YIKKLGSFDESSSRFYTSEIILALEYLHSMGIIHRDLKPENILLNEKMHIQITDFGTAKI

IGADDKKEGQPRAKSFVGTAQYVSPELLTEKSATKSSDLWALGCIVYQLQAGLPPFRASN

EYMIFQKIIKLEYD---FPDGFSQTARDLVSKLLV-----IDPSKRLGCDECGGYPALKA

HPFLEGVDWENLHEQKAP---VLKPCTVVDGEEIHSEYY--VDDFD-LELNPTSLSEYLK

RIETHDRPHSPTRDNKYVVD-----------LTPAERKQRLERQGKENKWHRFVEGNLIL

KNGWVDKRKGLFARRRMLLLTEGPHLYYVDPENMVLKGEIPWSPQLKVEAKN--------

------FKTY--FVHTPNRTYYLIDPESDAVEWCQAIKEMYRHTYQKSI-----

>L variegatus

----------------------------------------------------------KH

KMRYEPRYRWYRPPAYIFYMEQHYPIIKS--HRQIARQLQNKQMYQRYDLNCGLCMCKAI

MLFFEELFCSGEND-----------LKMNKSMFKKIKTLGIGAFGQVAIAQKRDTKAMYA

VKYLRKAHIIRKDQAKYVLAEQEILAALDHPFLVKLYFTFQDKEKLYMVLDYAPGGDLHY

YLIQLGSFDEETSRFYAAEIILALEYLHALGIVYRDLKPENILLDEKGHVKISDFGRAKI

IGTDDKKDGITQLMS-SGTPLYMAPELVEEKPYDHSADLWALGCILYQLQAGLPPFYASN

IYQLVQKIIKLEYD---FPDGFSQTARDLVSKLLVSAILPLDENKR--NQVCILFQIIMA

FFNF----FFPIHCQ------IYYLIHIISYKYYYYYYYSTVDDFD-LELNPTSLSEYLK

RIETHDRAHSPTRDNKYVVD-----------FTPGERKQRLERQRKENKWHRFVEGNLIL

KNGWVDKRKGLFARRRMLLLTEGPHLYYVDPENMVLKGEIPWDKLSRAAYIS--------

------VSCC----FQPNRTYYLIDPESDAVEWCSAIKEMYRHTYQKSI-----

>M musculus

------------------------------------------------------------

MARTTSQLYDAVPIQ-------SSVVLCSCPSPSMVRS-QTEPGSSPGIPSGVSRQGSTM

DGTTAEARPSTNPLQQHPAQLPPQPRKKRPEDFKFGKILGEGSFSTVVLARELATSREYA

IKILEKRHIIKENKVPYVTRERDVMSRLDHPFFVKLYFTFQDDEKLYFGLSYAKNGELLK

YIRKIGSFDETCTRFYTAEIVSALEYLHGKGIIHRDLKPENILLNEDMHIQITDFGTAKV

LSPESK---QARANSFVGTAQYVSPELLTEKSACKSSDLWALGCIIYQLVAGLPPFRAGN

EYLIFQKIIKLEYH---FPEKFFPKARDLVEKLLV-----LDATKRLGCEEMEGYGPLKA

HPFFETITWENLHQQTPPKLTAYLPAMSEDDEDCYGNYDNLLSQFGFMQVSSSSSSHSLS

TVETSLPQRSGSNIEQYIHDLDTNSFELDLQFSEDEKRLLLEKQAGGNPWHQFVENNLIL

KMGPVDKRKGLFARRRQLLLTEGPHLYYVDPVNKVLKGEIPWSQELRPEAKN--------

------FKTF—FVHTPNRTYYLMDPSGNAHKWCRKIQEVWRQQYQSNPDAAVQ

PI3K

>H sapiens (alpha)

----------------------MPPRPSS-GELWGIHLMPPRILVECLLPNGMIVT----

---------LECLREATLITIKHELFKEA----------------------RKYPLHQLL

QDESSYIFVSV----------TQEAERE--EFFDETRRLCDLRLFQPFLKVIEPVGNRE-

-EKILNREIGFAIGMPVCE---FDMVKDPEVQDFRRNILNVCKEAVDLRDLNSPHSRAMY

VYPPNVESSP--ELPKHIYNKLDKGQIIVVIWVIVS--------------PNNDKQKYTL

KINHDCVPEQVIAEAIRKKTRSMLLSSEQLKLCVLEYQGKYILKVCGCDEYFLE-KYPLS

QYKYIRSCIMLGRMPNLMLMAKES----LYS------QLPMDCFTMPSYSRRISTATPYM

NGETSTKSLWVINSALRIKILCA----TYVNVNIRDIDKIYVRTGIYHGGEPLCDNVNTQ

RVPCSNPRWNEWLNYDIYIPDLPRAARLCLSICSVKG------------------RKGAK

EEHCPLAWGNINLFDYTDTLVSGKMALNLWPVPHG----LEDLLNPIGVTGSNPNKE-TP

CLELEFDWFSS-VVKFPDMSVIEEHANWSVSREAGFSYSHAG-LSNRLARDNELRENDKE

QLKAISTRDPLSEITEQEKD-FLWSHRHYCVT-IPEILPKLLL-SVKWNSRDEVAQMYCL

VKDWPPIKPEQAMELLDCNYPDPMVRG----FAVRCLEKYLTDDKLSQYLIQLVQVLKYE

QYLDNLLVRFLLKKALTNQRIGHFFFWHLKSEMHNK------TVS-QRFGLLLESYCRAC

G-MYLKHLNRQVEAMEKLINLTDILKQEKKDETQ-KVQMKFLVEQMRRPD-FMDALQGFL

SPLNPAHQLGNLRLEECRIMSSAKRPLWLNWENPDIMSELLFQNNEIIFKNGDDLRQDML

TLQIIRIMENIWQNQGLDLRMLPYGCLSIGDCVGLIEVVRNSHTIMQIQCKG-GLKGAL-

-QFNSHTLHQWLKDKN-----------------KGEIYD---------AAIDLFTRSCAG

YCVATFILGIGDRHNSNIMVKDDGQLFHIDFGHFLDHKKKKFGYKRERVPFVLTQDFLIV

ISKGAQECTKTREFERFQEMCYKAYLAIRQHANLFINLFSMMLGSGMPELQSFDDIAYIR

KTLALD-KTEQEALEYFMKQMNDAHHGGWTTKMDWIFHTIK------QHALN

>H sapiens (gamma)

-----------MELENYKQPVVLREDNCRRRRRMKPRSAAASLSSMELIPIEFVLPTSQR

KCKSPETALLHVAGHGNVEQMKAQVWLRAL---------------------ETSVAADFY

HRLGPHHFLLL-----------YQKKGQWYEIYDKYQVVQTLDCLRYWKATHRSPGQIHL

VQRHPPSEESQAFQRQLTALIGYDVTDVSNVHDDELEFTRRGLVTPRMAEVASRDPKLYA

MHPWVTSKP--------LPEYLWKKIANNCIF----------------IVIHRSTTSQTI

KVSPDDTPGAILQSFFTKMAKKKSLMDIP----ESQSEQDFVLRVCGRDEYLVG-ETPIK

NFQWVRHCLKNGEEIHVVLDTPP--DPALDEVRKEEWPLVDDCTGVTGYHEQLTIHGKDH

ESVFTVS-LWDCDRKFRVKIRGIDIPVLPRNTDLT----VFVEANIQHGQQVLCQRRTSP

KPFTEEVLWNVWLEFSIKIKDLPKGALLNLQIYC-GKAPALSSKASAESPSSESKGKVQ-

----LLYYVNLLLIDHRFLLRRGEYVLHMWQISGKGEDQGSFNADKL-TSATNPDKENSM

SISILLDNYCH-PIALPKHQPTPDPE-------GDRVRA---------EMPN----QLRK

QLEAIIATDPLNPLTAEDKE-LLWHFRYESLKH-PKAYPKLFS-SVKWGQQEIVAKTYQL

LARREVWDQSALDVGLTMQLLDCNFSDENVRAIAVQKLESLEDDDVLHYLLQLVQAVKFE

PYHDSALARFLLKRGLRNKRIGHFLFWFLRSEIAQS------RHYQQRFAVILEAYLRGC

GTAMLHDFTQQVQVIEMLQKVTLDIKSLSAEKYDVSSQVISQLKQKLENLQNSQLPESFR

VPYDPGLKAGALAIEKCKVMASKKKPLWLEFKCADPTA-LSNETIGIIFKHGDDLRQDML

ILQILRIMESIWETESLDLCLLPYGCISTGDKIGMIEIVKDATTIAKIQQSTVGN----T

GAFKDEVLNHWLKEKS----------------PTEEKFQ---------AAVERFVYSCAG

YCVATFVLGIGDRHNDNIMITETGNLFHIDFGHILGNYKSFLGINKERVPFVLTPDFLFV

MGTS--GKKTSPHFQKFQDICVKAYLALRHHTNLLIILFSMMLMTGMPQLTSKEDIEYIR

DALTVG-KNEEDAKKYFLDQIEVCRDKGWTVQFNWFLHLVLGIKQGEKHSA-

>H glaber

-----------MELENSEPPVILREDNLRRRRRMKPRSAASSLSSTELVPIEFVLPTSQR

HSKTPETVLLHVAGHGNVEQMKAQVWFRAL---------------------ETSAAADFY

HRLGPDHFLLL-----------YQKKGQWYEIYDKYQVVQTLDCLQYWKVLHQSPGQIHL

VQRHAPSEETLAFQRQLTALIGYDVTDVSNVHDDELESARRRLVTPRMAEVASRDARLYS

MHPWVTSKP--------LPEYLSKKITNNCIF----------------IVIHRSTTSQTI

KVSADDTPASILQSFFAKMARKKSLMDIP----ESQSEQDFVLRVCGRDEYLVG-DTPIK

NFQWLRHCLKNGEEIHLVLDTPP--DPALDEVRKEEWPLVDDCTGVTGYHEQLTIHGKDH

ESVFTVS-LWDCDRNFRVKIRGIDIPVLPRNADLT----VFVEANIQHGQQVLCQRRTSA

KPFTEEVLWNVWLEFSIKIKDLPKGALLNLEIYC-GKAPGQSGKSPAETPGSESRGKAQ-

----LLYYVNLLLIDHRFLLRHGEYVLHMWQIPGKGEDQGSFNADKL-TSATNPDKENSM

SISILLDNYCH-PIALPKHQPTPDPE-------GDRVRA---------EMPN----QLRK

QLEEIIATDPLNPLTAEDKE-LLWHFRYESLKH-PKAYPKLFS-SVKWGEQETVAKTYQL

LARREIWDQSALDVGLTMQLLDCNFSDENVRAIAVQKLESLEDDDVLHYLLQLVQAVKFE

PYHDSALARFLLKRGLRNKRIGHFLFWFLRSEIAQS------RHYQQRFAVILEAYLRGC

GTAMLQDLAQQVHVIEMLQRVTIDIKSLSAEKYDVSAQVISQLKQKLENLQNSNLPKSFR

VPYDPGLKAGTLVIEKCKVMASKKKPLWLEFKCADPTA-LSNETIGIIFKHGDDLRQDML

ILQILRIMESIWETESLDLCLLPYGCISTGDKIGMIEIVKDATTIAKIQQSTVGN----T

GAFKDEVLNHWLKEKC----------------PIEEKFQ---------AAVERFVYSCAG

YCVATFVLGIGDRHNDNIMISETGNLFHIDFGHILGNYKSFLGINKERVPFVLTPDFLFV

MGTS--GKKTSLHFQKFQDVCVKAYLALRHHTNLLIILFSMMLMTGMPQLTSKEDIEYIR

DALTVG-KSEEDSKKYFLDQIEVCRDKGWTVQFNWFLHLVLGIKQGEKHSA-

>M_brandtii

---------------------------------MKPRGSAASLSSMELIPIEFVLPTSQR

TSKTPETALLHVAGHGNVEQMKAQVWLRAL---------------------ETSSAADFY

QRLSPDHFLLL-----------YQKKGQWYEIYDKYQVVQTLDCLRYWKVLHRSPGQIHV

VQRRGPSEETLAFQRQLTALMGYDL-----------EFTRRRLVTPRMAEVAGRDPQLYA

MHPWVTSKP--------LPEYLLKKIAHNCIF----------------IVIHRSTTSQTI

KVSADDTPAAILQSFFTKMAKKKSLMDIP----ESQSEQDFVLRVCGRDEYLVG-ETPLK

NFQWVRHCLKNGEEIHLVLDAPP--DPAQDEVRKEEWPLVDDCTGVTGYHEQLTIHGKDH

DSVFTVS-LWDCDRKFRVKIRGIDIPVLPRNADLT----VFVEANIQHGQQVLCQRRTSP

KPFTEEVLWNVWLEFSIRIKDLPKGALLNLQIYC-GKAPALSGKASAETTSPEARGKAQ-

----LLYYVNLLLIDHRFLLRHGEYVLHMWQISGKGEDQGSFNADKL-TSATNPDKDSSM

SICILLDNYCH-PIALPKHRPTPDPE-------GDRVRA---------EMPN----QLRK

QLEAIIATDPLNPLTAEDKE-LLWHFRYESLKH-PKAYPKLFS-SVKWGQQEMVAKTYQL

LARREVWDQSALDVGLTMQLLDCNFSDENVRAIAVQKLESLEDDDVLNYLLQLVQAVKFE

PYHDSALARFLLKRGLRNKRIGHFLFWFLRSEIAQS------RHYQQRFAVILEAYLRGC

GTAMLRDFTQQVQVIEMLQKVTIDIKSLSAEKYDVSSQVISQLKQKLENLQNSNLPKSFR

VPYDPGLKAGALVVEKCKVMASKKKPLWLEFKCADPTA-LSNETIGIIFKHGDDLRQDML

ILQILRIMESIWEIESLDLCLLPYGCISTGDKIGMIQIVKDATTIAKIQQSTVGN----T

GAFKDEVLNHWLKEKC----------------PIEEK-----------AAVERFVYSCAG

YCVATFVLGIGDRHNDNIMITETGNLFHIDFGHILGNYKSFLGINKERVPFVLTPDFLFV

MGTS--GKKTSLHFQKFQEVCTKAYLALRHHTNLLIILFSMMLMTGMPQLTSKEDIEYIR

DALTVG-KNEEDAKKYFLDQIEVCRDKGWTVQFNWFLHLVLGIKQGEKHSA-

>S franciscanus

----------------------MAPGSVPNLDQWSSHDMHSKIRLECLLPNGIYIP----

---------MQCSREATLEKIKAELWNKA----------------------KSEPLFNLL

LDLNFYIFQCI----------NYSAEQE--ELLDEKRRLCDIKPFQPWLKVVEPAGNRE-

-EKMLNYEIGQYCDYILHE---YDLMKNQEVHDFRSNMKRMCMAVQKAHMKWSWDRKAMY

LFPPEVDLS--------APNNLPESILINMRF------------------QH--SVSSKL

QKPSMEKAEDLVE----KAQKKMSMYQ------QNTQPEEYVLKIRSYENFLLG-GHPLS

KYQYIRKAVREGQKPELILIHKS--ELKVED------RFKDPMFNVLAQKEEQAPPLPSK

AG-RYVS--WDIEEKVRVTFH------LAKNVNVVEKSGVYLKAGIYHGEEPLCPIVSSK

QVPWPDPKWQETYEADIKVSDIPRSAKLCISIYSSEKKKIVKTTSGAQLDQGKTKRRNPR

QDLVPIAWVNLTFFDYRSQLRSGPLKLFMWPASED------IDLNPLGHCESNPSRDDTT

SIEMSFEQYRQGSVSYPTFDKVIERA-------ADIARSD----DNPFQTPS---EQVLE

ELRQIVEKDPLEPLSEQERE-DLWLARQDCRDQLPNALPRLLM-CVHWNHRDEVAQVSQG

EKR---------------------------------------GEGKKKAYEVVIKPPHYQ

SQYTIALAIMYYQCAPT-----HFIN-NSRSEMHKP------SVS-RRFGLMLEAYCRSL

G-PYLKLLVRQVEALDKLSKLTESLNERKAKQQD-KGEIRDMMKKVLQQDSYADTLSNFV

SPLSHAIISKFHIIIKVKFMSSKMRPLWLVFENDDPI----GDDVFVLFKNGDDLRQDML

TLQVMQIMDNIWQEEGLDLRIIPYKTLATGPKVGLIEVVTNSDTIANIQKQH-GSVNVLT

AAFQKAALFEWIKKHS----------------PTDEMLD---------AAIETFTKSCAG

YCVATFILGIGDRHSSNIMVIQSLQIFHIDFGHFLGNYKKKYGIKRERVPFVLTHDFLHV

ITKGK-DASKSIEFDSFRRCCEEAFMILRRRGNLLINLFAMMLSSGIPEL-SDETIKYLR

DVLVLD-RNEDDALRFFQGKFNEALKNSWKTSLDWMMHHI-------AHRD-

>S_purpuratus

----------------------MAPGSVPNLDQWSEGDLHANVELMCLLPNGIYIP----

---------LQCSREATLTNIKRELWTKA----------------------KSEPLFNLL

LDLNFYIFQCI----------NYSAEQE--ELLDEKRRLCDIKPFWSTLRVVRKAGDVE-

-DRVLNSQIGLCIGKSLHE---YDMMKNQEVHDFRSNMKRMCMSVQKAHMKWPWDKKAMY

LFPPEVDLA--------APNNLPENLLINMRF------------------SSSLSTTSVL

QVKSMEKAEDLVE----KAQKKMSMYQ------QNTQPEEYVLKIRSYEDFLLG-GHPLS

KYKYIRKAVKKGQKPELILIHKS--ELKVED------RFKDPMFNVLAQKEEQAPPLPSK

AG-RYVS--WDIEEKVRVTFH------LAKNVNVIEKSGVRLKAGVFHGDEELCPFFTSK

EVEGPDPKWQETYEADINVSNLPRMAKLCIMVYTLGNKKIVKTTSGAQLDQGKAKKKFPR

KDLVPIAWVNLTFFDYRSQLRSGPHKLFMWPASED------IDLNPLGHCESNPSKDDTT

CIEVSFEQYRQGSVSYPTFDKVIERA-------ADIARSD----DNPFETPS---EQALE

ELRQIVEKDPLEPLSEQERE-DLWLARQDCRDQLPHALPRLLK-CVHWNHRDEVAQV---

------------------------------------------NDQLSQYLLQLVQALKYE

THLDCAIGKFLLDRALSNQRIGHFLFWHLRAEMHQS------AVC-TRFGLMLEAYCRGS

M-AHIKTLSKQVESLNKMKNLNELIKSKQAKQQD-KGEIRDMMQKVLQQDSYADTLSNFV

SPLAPSNKLKCLRLNKCKFMSSKMRPLWLVFENDDPI----GDDVYILFKNGDDLRQDML

TLQVMQIMDNIWQEEGLDLRIIPYKTLATGPKVGLIEVVTNSDTIANIQKQH-GSVNVLT

AAFQKAALFEWLKKHS----------------PTEEMLN---------KAIEDFTFSCAG

YCVATYVLGIGDRHNDNIMVKESGQLFHIDFGHFLGNYKRKFGLKRERVPFVLTHDFVHV

ITRGK-GASTTTEFDSFRRCCEEAFMILRRRGNLLINLFAMMLSSGIPEL-SDETIKYLR

DVLVLD-RSEDDALRFFQGKFNEALKNSWKTSLDWMMHHI-------AHRD-

>L variegatus

----------------------MPPVSVPNLDQWSSHEMPSKIRLMCLLPNGIYIP----

---------MQCSREATLEKIKQMLWRKA----------------------KSYPLAHLL

LEASYYIFQCI----------TYSAEQE--ELYDEKRRLCDIKPFQPTLKVVRPAGDRE-

-EKMLNYQIG-CIGKSLHE---YDLMKNQEVHDFRSNMKRMCMSVQKAHMKWSWDRKATY

LFPPQVDLS--------APNDLPESILIDMRF--------------------FSSWVSLS

RGSGFAVAVVLLWLRSPKTGKCAAFAK-------NAHIPQYPIIKQKKGATERS-FHPRK

----IAQAKKKAPHPFIRMRARP---IKVPQKE--CPQIIPPIICIAGHNQVIATYIAAQ

----IIS--CHSCSKCRSLIQ------IISSMQIQ----IYLKAGIYHGDEELCPFFTSK

EVEGPDPRWGETYEAEIKVSILPRMAKLCVMVYTLGNKKIVKTTSGAQLDQGKQRRRNPR

KDLVPIAWVNLTFFDYRSQLRTGPVKLFMWPASDD------IDLNPLGPCESNPSRDDTT

CIELSFEQYRQGSVSYPTFDKVIERA-------ADIARSD----DNPFETPQ---EQALD

ELRQIVEKDPLEPLSEQERE-DLWLARQDCRDQLPHALPRLLR-CVHWNHRDEVAQV---

------------------------------------------RRGFICSCSQIIQVLKYE

SYLDNPLARFLLKRSLYNQRIGHFSFPILRAEMHQS------AVC-TRFGLMLEAYCRGS

M-AHIKTLSKQVSSLNKMKNLNELIKSKQAKQQD-KGEIRDMMKKVLQQDSYADTLSNFV

SPLSPAFFI-FLRLNKCKFMSSKMRPLWLVFENDDPI----GDDVFLLFKNGDDLRQDML

TLQVMQIMDNIWQEEGLSLRIIPYRTLATGPKVGLIEVVTNADTIANIQKQH-GSVNVLT

AAFQKTALFEWIKKHS----------------PTEERLS---------QAIEDFTLSCAG

YCVATYVLGIGDRHNDNIMVTKSGQLFHIDFGHFLGNYKRKFGVKRERVPFVLTHDFVHV

ITRGK-GASTTTEFDQVP-CCEEAFMILRRRGNLLINLFAMMLSSGIPEL-SDETIKYLR

DVLVLD-RSEDDALRFFQGKFNEALKNSWKTSFDWMMHHI-------ANRD-

>M musculus

-----------MELENYEQPVVLREDNLRRRRRMKPRSAAGSLSSMELIPIEFVLPTSQR

ISKTPETALLHVAGHGNVEQMKAQVWLRAL---------------------ETSVAAEFY

HRLGPDQFLLL-----------YQKKGQWYEIYDRYQVVQTLDCLHYWKLMHKSPGQIHV

VQRHVPSEETLAFQKQLTSLIGYDVTDISNVHDDELEFTRRRLVTPRMAEVAGRDAKLYA

MHPWVTSKP--------LPDYLSKKIANNCIF----------------IVIHRGTTSQTI

KVSADDTPGTILQSFFTKMAKKKSLMNIP----ESQSEQDFVLRVCGRDEYLVG-ETPLK

NFQWVRQCLKNGDEIHLVLDTPP--DPALDEVRKEEWPLVDDCTGVTGYHEQLTIHGKDH

ESVFTVS-LWDCDRKFRVKIRGIDIPVLPRNTDLT----VFVEANIQHGQQVLCQRRTSP

KPFAEEVLWNVWLEFGIKIKDLPKGALLNLQIYC-CKTPSLSSKASAETPGSESKGKAQ-

----LLYYVNLLLIDHRFLLRHGDYVLHMWQISGKAEEQGSFNADKL-TSATNPDKENSM

SISILLDNYCH-PIALPKHRPTPDPE-------GDRVRA---------EMPN----QLRK

QLEAIIATDPLNPLTAEDKE-LLWHFRYESLKH-PKAYPKLFS-SVKWGQQEIVAKTYQL

LARREIWDQSALDVGLTMQLLDCNFSDENVRAIAVQKLESLEDDDVLHYLLQLVQAVKFE

PYHDSALARFLLKRGLRNKRIGHFLFWFLRSEIAQS------RHYQQRFAVILEAYLRGC

GTAMLQDFTQQVHVIEMLQKVTIDIKSLSAEKYDVSSQVISQLKQKLESLQNSNLPESFR

VPYDPGLKAGTLVIEKCKVMASKKKPLWLEFKCADPTV-LSNETIGIIFKHGDDLRQDML

ILQILRIMESIWETESLDLCLLPYGCISTGDKIGMIEIVKDATTIAQIQQSTVGN----T

GAFKDEVLNHWLKEKC----------------PIEEKFQ---------AAVERFVYSCAG

YCVATFVLGIGDRHNDNIMISETGNLFHIDFGHILGNYKSFLGINKERVPFVLTPDFLFV

MGSS--GKKTSPHFQKFQDVCVRAYLALRHHTNLLIILFSMMLMTGMPQLTSKEDIEYIR

DALTVG-KSEEDAKKYFLDQIEVCRDKGWTVQFNWFLHLVLGIKQGEKHSA-

>C elegans

MSMGRSPSTTFRSRTGSHGARDLIAGHGRNSRRISQMHVNILHPQLQTMVEQWQMRERPS

LETENGKGSLLLENEGVADIITMCPFGEVISVVFPWFLANVRTSLEIKLSDFKHQLFELI

APMKWGTYSVKPQ---DYVFRQLNNFGEIEVIFNDDQPLSKLELHGTFPMLFLYQPDGIN

RDKELMSDISHCLGYSLDK------LEESLDEELRQFRASLWARTKKTCLTRGLEGTSHY

AFPEEQYLCVGESCPKDLESKVKAAKLSYQMFWRKRKAEIN--------GVCEKMMKIQI

EFNPNETPKSLLHTFLYEMRKLDVYDT------DDPADEGWFLQLAGRTTFVTNPDVKLT

SYDGVRSELESYRCPGFVVRRQS---LVLKDYCRPKPLYEPHYVRAHERKLALDVLSVSI

DSTPKQS---KNSDMVMTDFRPTASLKQVSLWDLDAN--LMIRPVNISGFDFPADVDMYV

RIEFSVYVGTLTLASKSTTKVNAQFAKWNKEMYTFDLYMKDMPPSAVLSIRVLYGKVKLK

SEEFEVGWVNMSLTDWRDELRQGQFLFHLWAPEPT------ANRSRIGENGARIGTNAAV

TIEISSYGGRVRMPSQGQYTYLVKHR-------STWTETLN---IMGDDYESCIRDPGYK

KLQMLVKKHESGIVLEEDEQRHVWMWRRYIQKQEPDLLIVLSELAFVWTDRENFSELYVM

LEKWKPPSVAAALTLLGKRCTDRVIRKFAVEKLN----EQLSPVTFHLFILPLIQALKYE

PRAQSEVGMMLLTRALCDYRIGHRLFWLLRAEIARLRDCDLKSEEYRRISLLMEAYLRGN

E-EHIKIITRQVDMVDELTRISTLVKG--------MPKDVATMKLRDELRSISHKMENMD

SPLDPVYKLGEMIIDKAIVLGSAKRPLMLHWKNKNPKSDLHLPFCAMIFKNGDDLRQDML

VLQVLEVMDNIWKAANIDCCLNPYAVLPMGEMIGIIEVVPNCKTIFEIQVGT-GFMNTAV

RSIDPSFMNKWIRKQCGIEDEKKKSKKDSTKNPIEKKIDNTQAMKKYFESVDRFLYSCVG

YSVATYIMGIKDRHSDNLMLTEDGKYFHIDFGHILGHGKTKLGIQRDRQPFILTEHFMTV

IRSGKSVDGNSHELQKFKTLCVEAYEVMWNNRDLFVSLFTLMLGMELPELSTKADLDHLK

KTLFCNGESKEEARKFFAGIYEEAFNGSWSTKTNWLFHAVKHY---------

>SIG

------------------------------------------------------------

------------------------------------------------------------

------------------------------------------------------------

------------------------------------------------------------

---------------------------------------------------~~~-~~~~~

~~~~~~~~~~~~~~~~~~~~~~~~~~~~~~~~~~~~~~~~~~~~~~~~~~~~~~~~~~-~

------+-----------------------------------------------------

------------------------------------------------------------

------------------------------------------------------------

------------------------------------------------------------

------------------------------------------------------------

------------------------------------------------------------

------------------------------------------------------------

------------------------------------------------------------

------------------------------------------------------------

------------------------------------------------------------

------------------------------------------------------------

------------------------------------------------------------

-------------------~~~~~~~~~~~~~~~~~~~~~~~~~~~~~~~~~~~~~~~~~

~~~~~~~~~~~-------------------------------------------------

POT1

>H sapiens

------------------------------------------------------------

-----------------------------------M------------------------

------------------------------------------------------------

SLVPATN--------YIYTPLNQLKGGTIVNVYGVVKFFKPPYLSKGT-------DYCSV

VTIVD----QTNVKLTCLLFSGNYEALPIIYKNGDIVRFHRLKIQVYKKETQGITSSGFA

SLTFEGTLGAPIIPRTSSKYFNFTTEDHKMVE-------ALRVWASTHMSP-----SWTL

LKLCDV--QPMQYFDLTCQLLGKAEV---DGASFLLKVWDGTRTPFPSWRVL---IQDLV

LEGDLSHIHRLQNLTIDILVYDNHVHVARSLKVGSFLRIYSLHTKLQSMNSENQTMLS--

--------LEFHLHGGTS---------YGRGIRVLPESNSDVDQLK--------------

----------KDLESANLTA-----NQHSDVIC----------------------QSEPD

DSFP----SSGSVSLYEVERCQQLSATILTDHQYLERTPLCAILKQKAPQQYRIRAKLRS

YKPRRLFQSVKLHCPKCHLLQEVPHEGDLDIIFQDGATKTPDVKLQNTSLYDSKIWTTKN

QKGRKVAVHFVKNNGILPLSNECLLLIEGGTLSEICKLSNKFNSVIPVRSGHEDLELLDL

SAPFLIQGTIHHYGCKQCSSLRSIQNLNSLVDKTSWIPSSVAEALGIVPLQYVFVMTFTL

DDGTGVLEAYLMDSDKFFQIPASEVLMDDDLQKSVDMIMDMFCPPGIKIDAYPWLECFIK

SYNVTNGTDNQICYQIFDTTVAEDVI

>H_glaber

------------------------------------------------------------

-----------------------------------M------------------------

------------------------------------------------------------

SLTPVTN--------YIYTPLNQLKGGTIVNVYGVVKFFKPPYLSKGT-------DYCSV

VTIVD----QTNVKLTCLLFSGNYEALPRIYKIGDIVRFHRLKIQVYKNEPQGITSSGFA

SLTFEGTLGAPVIPRTSSKCFHFTAADHKMVE-------ALRIWSSTHISP-----SSTL

VQLCDV--QPMQYFDLTCQLLGKAQV---DGASFLLKVWDGTRTPFPSWRVS---IEDHV

LEGDLNHIRRLQNLTIDILVYDNHVQVAKSLKIGSFLRIYSLHTKLQSVTSESQAPLS--

--------LEFHLHGGTS---------YGRGVTVLPESNSDVDQLK--------------

----------KALETADLTA-----AQHSDGLY----------------------QSDHE

DSY-LTLSSSGSVSLYEVERCQQLSATILTDHQYLEKTPLCAILKQKAPQQYRIRAKLRS

YKPKRLFQSVKLYCPKCHSLQEVPHEDNLDAVLRDGATKTPDTKLQNTPLYDSQIWTTED

QGGRKVVIHFVKYNGILPLSNECLILIEGGTVGEICKLSSEFHSIIPVKSGPKDLELLDL

SVPFLVQGRLYHYGCKQCSSLKPVQNLNSLVGETSWIPSSVAEVLGIVPLQHVFVMTFTL

DDGTGVLEAYLMDSDKFFQIPASEVLRDDDLQRNMDLIMDMFCPPGTKIDAYPWMECVIK

SYNVTNGMEQQICYQIFDTTVADDVI

>M_brandtii

--------------------MGCYMIYGCTVDTLLEEVVGSVLYSDTIAVDGFIVLSSPT

ASKDGGHCLFLKALEDFKSTVYVYVKSIIDFSTESM------------------------

------------------------------------------------------------

SLVPATR--------YTYTPLNELKGGMIVNVYGVVKFFKPPYLSKGT-------DYCSV

VTIVD----QTNVKLTCLLFSGNYESLPIIYKNGDIVRFHRLKIQVYKNETQGITSSGFA

SLTFEGTLGAPVIPRTSSKYFNFTTEDHKMVE-------TLRAWASTHISP-----SSTL

VKLCDV--QSMQYFDLTCQLLGKAEV---DGASFLLKVWDGTRTPFPSWRVC---VQDLV

FHGDLSHIRRLQNLAVDILVYDNHVQVAKSLKVGSFLRIYSLHTKLQSVNSENQATLLA-

--------LEFHLHGGTS---------YGRGIRVLPESNSDVDQLK--------------

----------KALECASLTA-----NQCSDGVC----------------------QSECE

DSFP---TSSGSVSLYEVERCQQLSATILTDHQYLENTPLCAILKQKAPQRYRIRAKLRS

YKPRKLFQSIKLYCPNCQSMQEVLHEGDLNRILQENATKTPNTKLQNTSLYDSKVWSTKD

QGERQVAVHFVKNNGILPLSNDCLILIEGGTLSEIYKLADKFHSVIPVKSGHEDLELLDL

SVPFLIQGKIHHYGCKKCSSLRPIQNLNNLDDKTSWIPSSVAEVLGIVPLQHVFVMTFTL

DDGTGVLEAYLMDSDKFFHIPASEILTNDYLQESMDMIMNMLCPPGKKIDTYPWLECFIK

SYNVTSGTEQQICYQIFDTTVADDVI

>S franciscanus

------------------------------------------------------------

---------RISFFGGLLMGHLAYKCANLNFSSYRI------------------------

------------------------------------------------------------

ARARYRQK-------Y------------------------PP------GMFLSSLDYCMS

LTIVDPSLPLENSGLKCVIFHQDIKKLPQVHSIGDIVRLHRLKIQQYNNQFQGAKGDGFS

SLVFDGRSSAPVEPRSSSVNYTLSDQTY-MVQ-------ELRLWATKQTSVPLAAKSWTL

SQIVSR-----EYFDLICQVVAVVII--PAESCVLIKVWDGTKY---KWYVRCLLVSKVQ

VKWDTELACRAEGLILDVALYDNHMKAGRELQPGDFVRLYNLHAASYVPPNTAPAAASAN

QDDSNTDMIELVLHRGTA---------YGRGLVVMKEYEEDVRVMKRVDIQIFHLSLALI

LDLT------TILQPTPYIFDINLMNECRQSKCW-VAIS--DFNLLKE-GMTDGERSTPG

QKNPENASWPSSFNSEQELQCMQQPIS-----GKVRVSKI-KRMMDHPPLLFRLKAAVVD

YYPRTIQDFVHLFCSKCRMRYCL---------------------LVHSASISGKMHVYAN

VIHRHCSFFRVYR-------QRC-------------------------CASAVSLRAQIM

AARFYRESRDHLTR----------------------------------------------

------------------------------------------------------------

--------------------------

>S_purpuratus

MAALKEMSESDLAPELAGLQKISLSELHPSSTLLNRYVMGKMVDRSPLITKGKLPVLRLV

LKESSSSHINVYTFGDFATGIDAYKNGDSVFISNAVIERSHTFTKDGHHKCQLMVHKGRS

HALVWVCPQQEQGGSNTASTSAATATTTTPTTQKGPTTTTDHAGSEQGTPSKATHGRMVT

ITAESSP--IKSKRTYEYKCLNDVKEARSVDVYGVVKFLQPPYKTKGPGMFLSSIDYCMS

LTIVDPSLPQDNFGLKCVIFHQDIKKLPQVHSIGDIVRLHRLKIQQYNNQLQGAKGDGFS

SLVFDGRPNAPVEPRSSSVNYTLSDQDKEKVQ-------ELRLWATQQTSVPLAGKSWTL

SQIVSR-----EYFDLICQVVAVVII--PAESCVLIKVWDGTKY---KWPVRCLDVSKVQ

VKWDTALACRAEGLILDVALYDNHMKAGRELKPGDYVRLYNLHAASYVPPNTAPAAASTN

QDNGDTDMIELVLHRGTA---------YGRGLVVMKEYEEDVRVMKR---RMEELSTRKN

QPSSNGQGSLNIGQGSLITGQVSHRSGQRSGSND-IELDNLDWEVLDSLGMTDGERSTQG

QKNPENASWPSSLNSEQELQCMQQPISVITSYHDSRVSKI-KRMMDHPPLLFRLKAAVVD

YYPRTIQDFVHLFCSKCRMSCRIP----------------TAKSAATNPPQDEQSRSDKD

RKRKAEQ------------GQRC-------------------------CAPPVSLLAQIM

AARFYRESRDHLTRSRGYNVAQPAVRMFFDAPPEAPTPSASASASELPGIG---------

--------------------------------------------DACNVYLWPW-EGFWQ

TKRVCDQTP-----------------

>L variegatus

MAALKDMSESDVAPELSGLQMLPLVELYPSSSLLNRYVMVSVLYRYHLHIFCTASM--VY

NDRKSIQHLPELHLYCPINDEGNTSLADTIFISNVMIERSHTFSKDNHHKCHLMVHKARS

HARIWVSP--------SCYYMRIRCWIAISLSLVQPKNICSRIIKIHYSIFKLHSKFISQ

FILQISKQISSSNRTYKYTCLNDIKETGSVDVYGVVKFFKPPYKTKGPGPSQPR-SYCMS

LTIVDPSLPEEDSGLKCVLFQQDIKKLPEVLSKGDIVRLHRLRIQKYNSDFQGAKGNGFS

SLVFDGRPNTPLEPRSSSVNYTLTDQDKEKVRIEAICLVHYKILMSIVLSSCKAAKARIA

QIIIAQYSQQNQYAAYAYPSIGIYYIIIQIAGMIYIASICNEKYKNQKYAIQKSPSCIIC

YHISYIYHAIIQQQISPVAYHYQH--------PPTY---CNLHFQIAIISAHEPEKSSSY

Q-------YVKIIHKCQQVHIIQPIIDYYYAAYLICQIIAKIQTIN----PLAHQSTMMY

QIPSYCHLMCQYFADAIIPAESCILIKCDDGSKYKLYMYIWIWSLM---------RIHCK

IKYSHHYCY-----------CLFIFLSVITQYQDCQVSKI-KQMKDHPPHLFRLKATVVD

YYPRTIQDFVHLFCSQCRMIYCYQ----------------PECS--AYPSYLYQNFHSPE

RSYLTISISKLERLIRLHIIKKC-------------------AFIKPAKSGHP--IKFTF

HYPLLEEIILQAIKSNGKSTSKVIYRFLS----PSIRPSACAR-----------------

-------------------------------------------------------PCQIH

SSKPAEYK------------------

>M_musculus

------------------------------------------------------------

-----------------------------------M------------------------

------------------------------------------------------------

SLVSTAP--------YTYTPLNLLKEGTIANVYGVVKFFKPPYVSKGT-------DYCSV

VTIVD----QTNVKLTCMLFSGNYEALPIIYKVGDIVRFHRLKIQVYKNELQGINCSGFA

SLTFEGTVGMPVTARTSSKVFSFTPQDQKMVE-------ALRVWASKHISA-----SSTL

VQLCDA--QPMQYYDLTCQLLGKAQV---DSTAFLLKVWDGTQTVLPSWRVS---TQDLT

FEGDLSHIERLQSLVVDILVYDNHVQVARSIEVGCFLRLYSLHTKLQPGNSETSSSESLR

--------LEFHLHGGTS---------YGRGIRVLPDTSPCVDQLK--------------

----------KALEGANLPV-----TETSTGIC----------------------QSENG

DSSALSNSGSGAVSPYEEERCQQVSATILTNHQHLEKTPLCAILTQKAPQQYRVRAKLRS

YLPRRLSQSVKLLCPKCHSVQEVPHGDSLDKILQDAATEAPDIKLKATSLYYSKVWTTED

QGGRQVAVHFVKNNGILPASSECLILIEGGRLCEVSKLSSKFHSVMPVRSGPESLELLTL

SAPFLIQGKVHHYGCKQCSSLKPIQNLNSRFHKGPWTPSSVAEALGVVPLQYVFVMVFTL

DDGTGVLEAYLKDSEHFFKIPASEVLTDDDLQRSLETIMDMICPPGIKVDAYPWLECLLK

SYNVTIGTERRICYQIFDTTVAENVV

>SIG

------------------------------------------------------------

------------------------------------------------------------

------------------------------------------------------------

------------------------------------------------------------

------------------------------------------------------------

------------------------------------------------------------

-------------------------------------------------------+----

------------------------------------------------------------

------------------------------------------------------------

------------------------------------------------------------

------------------------------------------------------------

------------------------------------------------------------

------------------------------------------------------------

------------------------------------------------------------

------------------------------------------------------------

PRDX1

>H sapiens

--------------------------------------------MSSGNAKIGHPAPNFK

ATAVMPDGQFKDISLSDYKGKYVVFFFYPLDFTFVCPTEIIAFSDRAEEFKKLNCQVIGA

SVDSHFCHLAWVNTPKKQGGLGPMNIPLVSDPKRTIAQDYGVLKADEGISFRGLFIIDDK

GILRQITVNDLPVGRSVDETLRLVQAFQFTDKHGEVCPAGWKPGSDTIKPDVQKSKEYFS

KQK

>H glaber

--------------------------------------------MSSGNAKIGHPAPSFQ

ATAVMPDGQFKDISLSDYKGKYIVFFFYPLDFTFVCPTEIIAFSDRAEEFKKLNCQVIGA

SVDSHFCHLAWINTPRKQGGLGPMNIPLVSDPKRTIAQDYGVLKADEGISFRGLFIIDDK

GILRQITVNDLPVGRSVDETLRLIQAFQFTDKHGEVCPAGWKPGSDTIKPDVQKSKEYFS

KQK

>M brandtii

MWCSSETLYPGLGFELPDSGASGVDPECVCASLRPRPRVAADSKMSSGNAKIGHPAPNFK

ATAVMPDGQFKDISLSDYKGKYVVFFFYPLDFTFVCPTEIIAFSDRAEEFKKINCQVIGA

SVDSHFCHLAWINTPKKQGGLGPMNIPLVSDPKRTIAQDYGVLKADEGISFRGLFIIDDK

GILRQITVNDLPVGRSVDETLRLVQAFQFTDKHGEVCPAGWKPGSDTIKPDVQKSKEYFS

KQK

>S franciscanus

---------------------------------------------MAGKARVQEPAPYFE

GTAVSTTGEFVDIKLSDYKGKKFILFFFSLSSTFVCPTEIIAFSDRADEFRAIDCEVLAA

SIDSHFCHLAWINTPRKSGGLGQMKIPLLSDMKKQIAEDYGVLLKDAGIALRGLFLIDPE

GVVRHMSINDLPVGRSVDETLRLVKAFQFVAEHGEVCPAGWTPDSETVSTNLHKA--LFV

NI-

>S purpuratus

---------------------------------------------MAAKARVQDPAPYFE

GTAVSTTGEFVDVKLSDYKGKYLVFFFYPLDFTFVCPTEILAFSDRSDEFTKIGCEVLAA

SCDSHFCHLAWTNTTKKLGGVGQLKIPLLSDMSGKIARDYGIMIEKEGISLRGLFIIDDK

GTLRQITINDLPVGRSVDEVLRLVQAFQFTDKFGEVCPAGWKPGDDTIKPGVKESKEFFG

KQ-

>L variegatus

-------------------------------------------HIFYSKQTLGWSL--YI

LKVILRSLTELEFIFNDF---FAHLFFVLFCSTFVCPTEIIAFSDRVEEFRAINTEVVAA

SVDSQFTHLAWINTPKKMGGVGELKIPLLSDMNGAIARDYGIMLEKEGVSLRGLFIIDDK

GILRQITINDLPVGRSVDETLRLVQAFQFTDKHGEVCPAGWKPGSDTVSPDVKESKEFFG

KK-

>M musculus

--------------------------------------------MSSGNAKIGYPAPNFK

ATAVMPDGQFKDISLSEYKGKYVVFFFYPLDFTFVCPTEIIAFSDRADEFKKLNCQVIGA

SVDSHFCHLAWINTPKKQGGLGPMNIPLISDPKRTIAQDYGVLKADEGISFRGLFIIDDK

GILRQITINDLPVGRSVDEIIRLVQAFQFTDKHGEVCPAGWKPGSDTIKPDVNKSKEYFS

KQK

PSEN1

>H sapiens

-MTELPAPLS-----YFQNAQ--MSEDNHLS-----------NTVRSQNDNRERQEH-ND

RRSLGHPEPLSNGRPQGNSRQVVEQDEEEDEELTLKYGAKHVIMLFVPVTLCMVVVVATI

KSVSFYTRK-DGQLIYTPFTEDTETVGQRALHSILNAAIMISVIVVMTILLVVLYKYRCY

KVIHAWLIISSLLLLFFFSFIYLGEVFKTYNVAVDYITVALLIWNFGVVGMISIHWKGPL

RLQQAYLIMISALMALVFIKYLPEWTAWLILAVISVYDLVAVLCPKGPLRMLVETAQERN

ETLFPALIYSSTMVWLVNMAEGD-----------PEAQRRVSKNSKYNAESTERESQDTV

AENDD------GGFSEEWEAQRDSHLGPHRSTP----ESRAAVQELSSSI--------LA

GEDPEERGVKLGLGDFIFYSVLVGKASATASGDWNTTIACFVAILI--------------

------------------------------------------------------------

-----------------------------------------------------------G

LCLT--------------------------------------------------------

------------------------------------------------------------

-----LLLLAIFKKALPA------LPISIT------------------------------

------------------------------------------------------------

------------------------------------------------------------

------------------------------------------------------------

------------------------------------------------------------

------------------------------------------------------------

------------------------------------------------------------

-FGLVF------------------------------------------YF----------

------------------------------------------------------------

------------------------------------------------------------

------------------------------------------------------------

------------------------------------------------------------

------ATDY-------------LVQP-------------------------FMDQLAFH

QFYI---------------------

>M_brandtii

-MTELPAPLS-----YFQNAQ--MSEDNHLS-----------NIVRSQNDSRERHEHSNE

RRRRGNPEPLSNGGPQGNTLQVVEQDEEEDEELTLKYGAKHVIMLFVPVTLCMVVVVATI

KSVSFYTRK-DGQLIYTPFTEDTETVGQRALHSILNAAIMISVIVVMTILLVALYKYRCY

KVIHAWLIISSLLLLFFFSFIYLGEVFKTYNVAVDYITIALLIWNFGVVGMIAIHWKGPL

RLQQAYLIMISALMALVFIKYLPEWTAWLILAVISVYDLVAVLCPKGPLRMLVETAQERN

ETLFPALIYSSTMVWLVNMAKGD-----------PEAQRKVSKNSNYNEQNTGSESQNPV

TESDD------GGFSEEWEAQRDSRLGPHHSTT----ESRSAVQEISSSI--------LA

SEDPEERGVKLGLGDFIFYSVLVGKASATASGDWNTTIACFVAILIAPKVRRQSDTWGPW

GDWSPCSRTCGGGISFRERFCYSQRRDGGSSCVGPARRHRLCHIESCPPGARDFRAEQCG

EFDSREFQGRQYQWLPYYGAPNKCELACIPKGESFYYRHREAVVDGTPCEPGKPDVCVDG

VCRVVGCDHKLDSSKQEDKCLQCGGDGTTCYPVTGTFDVSDLSRGYNQILIVPTGATSIR

IEEVAASRNFLAVKSIQGEYYLNGHWTIEAARALPVASTILHYERGAEGDLAPERLHARG

PTSEPLVIELISQESSPGVRYEYHLPLSTPRPGFSWSHGSWSDCSAECGGGHQSRPVFCT

IDNEVYPDHMCQHQPRPATRRPCSPHSCPQTQRWKTGPWTPCSASCGGGSQSRSVYCVWS

DGAGGEEAMEEVQCAGLPGKPATTQACNLQRCAAWREEPWGECSVSCGAGIRRRSVTCRG

DEGSLLHASACSFKDQPPLTEPCVHDDCPRLSDQAWHAGAWSPCSKSCSSGTRRRQVACA

IGPPSHCGSLQLWKPAEVEPCNTQPCPLPPGCPRHEELLEEGHRGEHLLGVRMRYSGKCG

LAGVWGWPSGAVERDPVSHDEDPVSRDEDPVSRDEDPVSRDEDPVSRDEDPVSRDEDPVS

RDEDPVSRDEDPVSRDEDPVSRDEDPVSRDEDPVSRDEDPVPQAHQPQAQQSEPTECRGS

QFGCCYDNVASAAGPFGEGCVGQPSYAYPVGCLLPSALGSCTDWAPRWYFIASVGRCNRF

WYGGCHGNANNFASEEECMSSCRGSQHTPRQPEPGAAGQSTHRAGAPLTWAEMLGGQHPP

PTAPPPGEHSLSQGGGEGPGPGAEPLDLKPRGQPALPPHPAWRPRHLGRRRQPGAGGARI

RQRTLPVSPGSVQHQQQPDGSLVISQVAVEDGGFYACVAFNGQDRDQQWVQLRVLGELTI

TGLPSTVTVPEGDTARLLCVVAGESVNIRWSRNGLPIRADGHRVHQSPDGTLLIHNLRAG

DQGSYTCSTYRGSQAVSRSTEVKVVPPALAAQPRDPGRDCMDQPELANCELILQAQLCGN

EYYSSFCCASCSRAQPHAQPVWQQG

>H_glaber

-MTELPAPLS-----YFQNAQ--MSEDNHLS-----------NIARSQNDSRERQDC-SE

RRRPGNPEPVSNGRPQGNSRQVVEQDEEEDEELTLKYGAKHVIMLFVPVTLCMVVVVATI

KSVSFYTRK-DGQLIYTPFTEDTETVGQRALHSVLNAAIMISVIVVMTILLVVLYKYRCY

KVIHAWLIISSLLLLFFFSFIYLGEVFKTYNVAMDYITVALLIWNFGVVGMISIHWKGPL

RLQQAYLIMISALMALVFIKYLPEWTAWLILAVISVYDLVAVLCPKGPLRMLVETAQERN

ETLFPALIYSSTMVWLVNMAEGD-----------PEAQRRVSKNSKYNTQSTEREAQDTG

RENDD------GGFSEEWEAQRDSHLGPHRSTP----ESRAAVQELSSNT--------LT

SEDPEERGVKLGLGDFIFYSVLVGKASATASGDWNTTIACFVAILI--------------

------------------------------------------------------------

-----------------------------------------------------------G

LCLT--------------------------------------------------------

------------------------------------------------------------

-----LLLLAIFKKALPA------LPISIT------------------------------

------------------------------------------------------------

------------------------------------------------------------

------------------------------------------------------------

------------------------------------------------------------

------------------------------------------------------------

------------------------------------------------------------

-FGLVF------------------------------------------YF----------

------------------------------------------------------------

------------------------------------------------------------

------------------------------------------------------------

------------------------------------------------------------

------ATDY-------------LVQP-------------------------FMDQLAFH

QFYI---------------------

>S franciscanus

MSAEILAPPSDGASNARRPIQ--VEGSNNPSYGTSVNAEVRGTSLQKQANAGNRPAN-QE

RRRERDPPPM----------------EEEEEDEMLKYGAKHVIMLFVPVSLCMLVVVATI

STVSFYTESGDVYLIYTPFHEKSDQAGTKAWNALANALIIIGIVLIMTIFLVVLYKYRCY

KVSHYYGY--QIIQTYRHQCILNLRCIVTYNIPMDYFTIAVIMWNFGMVGMVSIHWKGPL

RLQQLYLIVISALMALIFIKYLPEWTLWTILAAIAVYDEFAVLCPKGPLRMLVETAQERD

EQIFPALIYSYEIHQILF----------------PPICMYVIFFIFY-------------

---------------------------------------PILITYFCLYI----------

-----TGGVKLGLGDFIFYSVLVGKAS--ASGDWTTTVACFVAILQ--------------

------------------------------------------------------------

-----------------------------------------------------------G

SQSE--------------------------------------------------------

------------------------------------------------------------

-----LILLAIFKKALPA------LPISIA------------------------------

------------------------------------------------------------

------------------------------------------------------------

------------------------------------------------------------

------------------------------------------------------------

------------------------------------------------------------

------------------------------------------------------------

-FGLVF------------------------------------------YF----------

------------------------------------------------------------

------------------------------------------------------------

------------------------------------------------------------

------------------------------------------------------------

------CTSN-------------LVFP-------------------------FTDELASQ

QVYI---------------------

>S_purpuratus

MSAEILAPTSDGASNARRPIQ--VEGSNNPSYGTSVNAEVRGTSVQRRANAGNRPAN-QE

RRRERDPPPM----------------EEEEEDEMLKYGAKHVIMLFVPVSLCMLVVVATI

STVSFYTESGDVYLIYTPFHEKSDQAGTKAWNALANALIIIGIVLIMTIFLVVLYKYRCY

KVIHGWLVLSSLLLLFFFTFFYLQELLVTYNIPMDYFTIAVIMWNFGMVGMVSIHWKGPL

RLQQLYLIVISALMALIFIKYLPEWTLWTILAAIAVYDLFAVLCPKGPLRMLVETAQERD

EQIFPALIYSSTMVWLVGMADIDSPPPKSKAKQEPPVEEHTAEENATGGQEDQTVSSDQE

VN---------GGFDTVFTERRERDLERSANSSVTSEDRRAAVRALRQNNGPSNRQPPVD

EEEEEERGVKLGLGDFIFYSVLVGKAS--ASGDWTTTIACFVAILI--------------

------------------------------------------------------------

-----------------------------------------------------------G

LCLT--------------------------------------------------------

------------------------------------------------------------

-----LILLAIFKKALPA------LPISIA------------------------------

------------------------------------------------------------

------------------------------------------------------------

------------------------------------------------------------

------------------------------------------------------------

------------------------------------------------------------

------------------------------------------------------------

-FGLVF------------------------------------------YF----------

------------------------------------------------------------

------------------------------------------------------------

------------------------------------------------------------

------------------------------------------------------------

------CTSN-------------LVFP-------------------------FTDELASQ

QVYI---------------------

>L variegatus

---QIEKKTLKVKQVYFTPLHEIVEGSNNPSYGTSVNAEVRGTSVQRQANAGNRQAN-QE

RQRDRNPPPM----------------EEDEEDEMLKYGAKHVIMLFVPVSLCMLVVVATI

STVSFYTESGDLYRIYTPFHEKSEQAGTKAWNALANALIIIGIVLVMTIFLVVLYKYRCY

KTIEGFGHIMTIEQLYLSS---PSEKLVTYNIPMDYFTIAVIMWNFGMVGMVSIHWKGPL

RLQQLYLIVISALMALIFIKYLPEWTLWTILAAIAVYGQFSVLCPKGPLRMLVETAQERD

EQIFPALIYSCKKLFVQPIQRKQCF---------PLFCFIFHYCIICIPLIVKKNNLIPP

THRISIVFRQVGPYCQVCKKKNILIIQYKQKCIVMIIDKPICCILISAIH--------HT

SKISEITGVKLGLGDFIFYSVLVGKAS--ASGDWTTTVACFVAILI--------------

------------------------------------------------------------

-----------------------------------------------------------V

SCLT--------------------------------------------------------

------------------------------------------------------------

-----LILLAIFKKALPA------LPISIA------------------------------

------------------------------------------------------------

------------------------------------------------------------

------------------------------------------------------------

------------------------------------------------------------

------------------------------------------------------------

------------------------------------------------------------

-FGLVF------------------------------------------YF----------

------------------------------------------------------------

------------------------------------------------------------

------------------------------------------------------------

------------------------------------------------------------

------CTSN-------------LVFP-------------------------FTDELASQ

QVYI---------------------

>M_musculus

-MTEIPAPLS-----YFQNAQ--MSEDSHSS-----------SAIRSQNDSQERQQQ-HD

RQRLDNPEPISNGRPQSNSRQVVEQDEEEDEELTLKYGAKHVIMLFVPVTLCMVVVVATI

KSVSFYTRK-DGQLIYTPFTEDTETVGQRALHSILNAAIMISVIVIMTILLVVLYKYRCY

KVIHAWLIISSLLLLFFFSFIYLGEVFKTYNVAVDYVTVALLIWNFGVVGMIAIHWKGPL

RLQQAYLIMISALMALVFIKYLPEWTAWLILAVISVYDLVAVLCPKGPLRMLVETAQERN

ETLFPALIYSSTMVWLVNMAEGD-----------PEAQRRVPKNPKYNTQRAERETQDSG

SGNDD------GGFSEEWEAQRDSHLGPHRSTP----ESRAAVQELSGSI--------LT

SEDPEERGVKLGLGDFIFYSVLVGKASATASGDWNTTIACFVAILI--------------

------------------------------------------------------------

-----------------------------------------------------------G

LCLT--------------------------------------------------------

------------------------------------------------------------

-----LLLLAIFKKALPA------LPISIT------------------------------

------------------------------------------------------------

------------------------------------------------------------

------------------------------------------------------------

------------------------------------------------------------

------------------------------------------------------------

------------------------------------------------------------

-FGLVF------------------------------------------YF----------

------------------------------------------------------------

------------------------------------------------------------

------------------------------------------------------------

------------------------------------------------------------

------ATDY-------------LVQP-------------------------FMDQLAFH

QFYI---------------------

>SIG

------------------------------------------------------------

-+----------------------------------------------------------

------------------------------------------------------------

------------------------------------------------------------

------------------------------------------------------------

------------------------------------------------------------

------------------------------------------------------------

------------------------------------------------------------

------------------------------------------------------------

------------------------------------------------------------

------------------------------------------------------------

------------------------------------------------------------

------------------------------------------------------------

------------------------------------------------------------

------------------------------------------------------------

------------------------------------------------------------

------------------------------------------------------------

------------------------------------------------------------

------------------------------------------------------------

------------------------------------------------------------

------------------------------------------------------------

------------------------------------------------------------

------------------------------------------------------------

------------------------------------------------------------

------------------------------------------------------------

PTEN

>H sapiens

-------------------------------------------MTAIIKEIVSRNKRRYQ

---------------------------------------EDGFDLDLTYIYPNIIAMGFP

AERLEGVYRNNIDDVVRFLDSKHKNHYKIYNLCAERHYDTAKFNCRVAQYPFEDHNPPQL

ELIKPFCEDLDQWLSEDDNHVAAIHCKAGKGRTGVMICAYLLHRGKFLKAQEALDFYGEV

RTRDKKGVTI--PSQRRYVYYYSYLLKNHLDYRPVALLFHKMMFETIPMF-SGGTCNPQF

VVCQLKVKIYSSNSGPTRREDKFMYFEFPQP-LPVCGDIKVEFFHKQNKMLKKDKMFHFW

VNTFFI------------------------------------------------------

------------------------------------------------------------

------------------------------------------------------------

------------------------------------------------------------

------------------------------------------------------------

------------------------------------------------------------

------------------------------------------------------------

------------------------------------------------------------

-------------------------------------PGPEETSEKVENGSLCD------

------------------------------------------------------------

-----------------------------------------------------------Q

EIDSICSIERA-------------------------------------------------

------------------------------------------------------------

------------------------------------------------------------

------------------------------------DNDKEYLVLTL-------------

------------------------------------------------------------

------------------------------------------------------------

------------------------------------------------------------

-------TKNDLDKAN--------------------------------------------

------------------------------------------------------------

-------------KDKANRYFSPNFKVKLYFTKTV-EEP---------------------

------------------------------------------------------------

---------SNPEASSSTSVTPDVSDNE--------------------------------

----------------------------------PDHYRYSDTT--DSDPENEPF-----

---------------------------------DEDQHTQITKV--------

>H glaber

-------------------------------------------MTAIIKEIVSRNKRRYQ

---------------------------------------EDGFDLDLTYIYPNIIAMGFP

AERLEGVYRNNIDDVVRFLDSKHKNHYKIYNLCAERHYDTAKFNCRVAQYPFEDHNPPQL

ELIKPFCEDLDQWLSEDDNHVAAIHCKAGKGRTGVMICAYLLHRGKFLKAQEALDFYGEV

RTRDKKGVTI--PSQRRYVYYYSYLLKNHLDYRPVALLFHKMMFETIPMF-SGGTCNPQF

VVCQLKVKIYSSNSGPTRREDKFMYFEFPQP-LPVCGDIKVEFFHKQNKMLKKDKMFHFW

VNTFFI------------------------------------------------------

------------------------------------------------------------

------------------------------------------------------------

------------------------------------------------------------

------------------------------------------------------------

------------------------------------------------------------

------------------------------------------------------------

------------------------------------------------------------

-------------------------------------PGPEETSEKVENGSLCD------

------------------------------------------------------------

-----------------------------------------------------------Q

EIDSICSIERA-------------------------------------------------

------------------------------------------------------------

------------------------------------------------------------

------------------------------------DNDKEYLVLTL-------------

------------------------------------------------------------

------------------------------------------------------------

------------------------------------------------------------

-------TKNDLDKAN--------------------------------------------

------------------------------------------------------------

-------------KDKANRYFSPNFKVKLYFTKTV-EEP---------------------

------------------------------------------------------------

---------SNPEASSSTSVTPDVSDNE--------------------------------

----------------------------------PDHYRYSDTT--DSDPENEPF-----

---------------------------------DEDQHTQITKV--------

>M_brandtii

MVDTGSTESEQGSEAHAPEQETVQLEVAAPCIPPSNHELVPVNAESAPKNVVDTGEGTFR

GGNTRKSLEEDGSTRVTPSVQPHPQPVSLPRNMSVSQTMEDSCELDLVYVTERIIAVSFP

STANEENFRSNLREVAQMLKSKHGGNYLLFNL-SERRPDITKLHAKVLEFGWPDLHTPAL

EKICSVCKAMDTWLNADPHNVVVLHNKGNRGRIGVVIAAYMHYSNISASADQALDRFAMK

RFYEDKIVPIGQPSQRRYVHYFSGLLSGTIKMNNKPLFLHHVIMHGIPNFESKGGCRPFL

RIYQAMQPVYTSGIYNVQGDSQTSICITIEPGLLLKGDILLKCYHKKFRSPARDVIFRVQ

FHTCAIHDLGVVFGKEDLDDAFKDDRFPEYGKVEFVFSYGPEKIQGMEHLENGPSVSVDY

NTSDPLIRWDSYDNFNGHRDDGMEEVVGHTQGPLDGSLYAKVKKKDSLNGSTGAVNATRP

ALSATPNHVEHTLSVSSDSGNSTASTKTDKTDEPAPGPSSAPVALSPEEKRELDRLLSGF

GLEREKPGNMYHTQHLRSRLAGGPAVPSASRHVVPAQVHVNGGALASERETDILDDELPN

QDGHSVGSMGTLSSLDGITNTSEGGYPEALSPLTNGLDKPYAVEPMVNGGGYPYESASRA

VPAHAGHLAPMRPSYSAQEGLAGYQREGPHPAWSQQVTTSHYGHDPSSMFRSQSFPDTEP

QLPPAPARGGSSREAVQRGLNSWQQQQQQQQQQQQQQQQPHPPPRQQERAHLESVRPSRP

GPQPLAETPTPGLPEFPRAASQQEIEQSIEALNMLMLDLEPSSAAAPLHKSQSVPGAWPG

ASPLSSQPLSGSSYQTHPLTQSRSGYIPSGHSLGTPEPAPRASLESIPQGRSYSPYDYQA

CLAGPNQSYRPKSPASSSSLPAFLPTNHSPPGPQQPPTSLPGLAAQPQLPPKEVTSDPSR

TPEEEPLNLEGLVAHRVAGVQAREKQPAEPPAPLRRRAASDGQYENQSPEPSSPRSPGVR

SPVQCVSPELALTIALNPGGRPKEPHLHSYKEAFEEMEGTAPSSPPPSGVRSPPGLAKTP

LSALGLKPHNPADILLHPKGEEDGGQEVMEPLGEPRSYVESVVRTAVAGPRSQDPEPKSF

SAPAAQAYGHDTPLRNGTLGGSFVSPSPLSTSSPILSADSTSVGSFPSGESSDQGPRTPT

QPLLDSGFRSGSLGQPSPSAQRSYQSSSPLPTVGSSYSSPDYSLQQFSSPESQARPQFNA

AGVHAVPGSPQARHRTVGTNTPSSPGFGRRAVNPGMAAPSSPSLSHRQVMGPPATGFHGN

VVSSPQSSAVTTPGSPSLGRHPGAHQASGLHGSVAVTPGSPSRGQQPGAHQASGLPGNVA

TTPGSPSLGRHPGAHQGNLASNLHGNTISSPGSPILGRHLGASGSVVPGSPSLDRHVAYG

GYSTPEDRRPTLSRQSSASGYQAPSTPSFPVSPAYYPGLSSPATSPSPDSAAFRQGSPTP

ALPEKWRMSMGDRVGSLPNYATINGKVSSSPVASGMSSPSGGSTVSFSHTLPDFSKYSMP

DNSPETRAKVKFVQDTSKYWYKPEISREQAIALLKDQEPGAFIIRDSHSFRGAYGLAMKV

SSPPPTIMQQNKKGDMTHELVRHFLIETGPRGVKLKGCPNEPNFGSLSALVYQHSIIPLA

LPCKLVIPNRDPTDDSKDSSGPANSTTDLLKQGAACNVLFVNSVDMESLTGPQAISKATS

ETLAADPTPAATIVHFKVSAQGITLTDNQRKLFFRRHYPLNTVTFCDLDPQERKWMKTEG

GAPAKLFGFVARKQGSTTDNACHLFAELDPNQPASAIVNFVSKVMLSAGQKR

>S franciscanus

-----------------------------------------------FQ-----------

-------------------------------------------------LYGQQYSFVFP

-----------------------------YVRCSERNYDTSKFYHRVAHYPFDDHNPPRI

ELIKPFCEDLMQWLAADKQNVAAIHCKAGKGRTHTYICAYMLHRRLYQSASEALDFYASK

RTKNRQGVTI--PSQRRYVQYYGELIKRELEYKPETLLLKRIQLYTIPMM-SGGSCAPAF

TIYQQKVKIHSQQAYKVAKKERSVRCEFPLP-VPICGDIKIEFFNKPNK-LKK-------

------------------------------------------------------------

------------------------------------------------------------

------------------------------------------------------------

------------------------------------------------------------

------------------------------------------------------------

------------------------------------------------------------

------------------------------------------------------------

------------------------------------------------------------

-----------------------------------------------VSHVASK------

------------------------------------------------------------

-----------------------------------------------------------S

NLSSC-------------------------------------------------------

------------------------------------------------------------

------------------------------------------------------------

--------------------------------------LLLFQVLSL-------------

------------------------------------------------------------

------------------------------------------------------------

------------------------------------------------------------

------------------------------------------------------------

------------------------------------------------------------

------------------IFF---LQVICYFKRVENEKP---------------------

------------------------------------------------------------

---------RRPSGDSNDRSSGTSSDHE--------------------------------

------------------------------------NSDGGDIS--ETDSESE-W-----

---------------------------------D---VTEITHV--------

>S purpuratus

-------------------------------------------MNVIAD-----------

-------------------------------------------------IYPNIIAMGFP

AESLEGVYRNKLEVVVKFFNEKHGERYKVYNLCSERNYDTTKFYNRVAHYPFDDHNPPRL

ELIKPFCEDLDNWLGADGQNVAAIHCKAGKGRTGVMICAYMLHRRLYQTASEALDFYASK

RTKNRKGVTI--PSQRRYVEYYGNLIVRELEYKPTTLLLTRIDLETVPMF-NGGSCAPAF

VIYQQKVKIFSSQPYEVAKKERSVRCEFPLP-VPICGDIKIEFFNKPNK-LKKDRMFHFW

FNTFFVANS---------------------------------------------------

------------------------------------------------------------

------------------------------------------------------------

------------------------------------------------------------

------------------------------------------------------------

------------------------------------------------------------

------------------------------------------------------------

------------------------------------------------------------

-------------------------------------NLLNSSSQQDLANNGSS------

------------------------------------------------------------

-----------------------------------------------------------D

NSSHCSEDNME-------------------------------------------------

------------------------------------------------------------

------------------------------------------------------------

------------------------------------ALDADCTVLRL-------------

------------------------------------------------------------

------------------------------------------------------------

------------------------------------------------------------

-------QKDQLDRAN--------------------------------------------

------------------------------------------------------------

-------------KDKTNKWYSPNFKVICYFKRVENEKP---------------------

------------------------------------------------------------

---------RRPSGDSNDRSSGTSSDHE--------------------------------

------------------------------------HSDGGDIS--ETDSESE-W-----

---------------------------------D---VTEITHV--------

>L variegatus

-------------------------------------------VDVIAD-----------

-------------------------------------------------IYPNIIASSFP

ASGKQSIYRNPISEVSKFLNTKHPDHYKVFNLCSERNYDTSHFYHRVAHYPFDDHNPPRI

ELIRPFCEDLMQWLAADKQNVAAIHCKAGKVKSYLSSLCETPYISIIIAIPAPI--HHKI

FQLAAFGVTI--PSQRRYVEYYGDLIIRELEYKPTTLLLTRIDLETVPMF-NGGSCAPAF

VIYQQKVKIFSSQPYEVAKKERSVRCEFPLP-VPICGDIKIEFFNKPNK-LKKSIPLHQK

LDIKYYMRL---------------------------------------------------

------------------------------------------------------------

------------------------------------------------------------

------------------------------------------------------------

------------------------------------------------------------

------------------------------------------------------------

------------------------------------------------------------

------------------------------------------------------------

-------------------------------------PKPIIQLYSYPSASAWK------

------------------------------------------------------------

-----------------------------------------------------------S

SPEQCV------------------------------------------------------

------------------------------------------------------------

------------------------------------------------------------

--------------------------------------ESKYVIIGFVRR----------

------------------------------------------------------------

------------------------------------------------------------

------------------------------------------------------------

-------RKDQLDRAN--------------------------------------------

------------------------------------------------------------

-------------KDKTNKWYSPNFQVICYFKRVENEKP---------------------

------------------------------------------------------------

---------RRPSGDSNDHSSGTSSDHE--------------------------------

------------------------------------NSDGGDIS--ETDSESE-W-----

---------------------------------D---VTEITHV--------

>M musculus

-------------------------------------------MTAIIKEIVSRNKRRYQ

---------------------------------------EDGFDLDLTYIYPNIIAMGFP

AERLEGVYRNNIDDVVRFLDSKHKNHYKIYNLCAERHYDTAKFNCRVAQYPFEDHNPPQL

ELIKPFCEDLDQWLSEDDNHVAAIHCKAGKGRTGVMICAYLLHRGKFLKAQEALDFYGEV

RTRDKKGVTI--PSQRRYVYYYSYLLKNHLDYRPVALLFHKMMFETIPMF-SGGTCNPQF

VVCQLKVKIYSSNSGPTRREDKFMYFEFPQP-LPVCGDIKVEFFHKQNKMLKKDKMFHFW

VNTFFI------------------------------------------------------

------------------------------------------------------------

------------------------------------------------------------

------------------------------------------------------------

------------------------------------------------------------

------------------------------------------------------------

------------------------------------------------------------

------------------------------------------------------------

-------------------------------------PGPEETSEKVENGSLCD------

------------------------------------------------------------

-----------------------------------------------------------Q

EIDSICSIERA-------------------------------------------------

------------------------------------------------------------

------------------------------------------------------------

------------------------------------DNDKEYLVLTL-------------

------------------------------------------------------------

------------------------------------------------------------

------------------------------------------------------------

-------TKNDLDKAN--------------------------------------------

------------------------------------------------------------

-------------KDKANRYFSPNFKVKLYFTKTV-EEP---------------------

------------------------------------------------------------

---------SNPEASSSTSVTPDVSDNE--------------------------------

----------------------------------PDHYRYSDTT--DSDPENEPF-----

---------------------------------DEDQHSQITKV--------

SGK1

>H sapiens

MTVKTEAAKGTLTYSRMRGMVAILIAFMKQRRMGLNDFIQ-KIANNSYACKHPEVQSILK

ISQPQEPELMNANPSPPPSPSQQINLGPSSNPHAKPSD---------FHFLKVIGKGSFG

KVLLARHKAEEVFYAVKVLQKKAILKKKEEKHIMSERNVLLKNVKHPFLVGLHFSFQTAD

KLYFVLDYINGGELFYHLQRERCFLEPRARFYAAEIASALGYLHSLNIVYRDLKPENILL

DSQGHIVLTDFGLCKENIEHNSTTSTFCGTPEYLAPEVLHKQPYDRTVDWWCLGAVLYEM

LYGLPPFYSRNTAEMYDNILNKPLQLKPNI-TNSARHLLEGLLQKDRTKRLGAKDD-FME

IKSHVFFSLINWDDLINKKITPPFNPNVSGPNDLRHFDPEFTEEPVPNSIGKSPDSVLVT

ASVKEAAEAFLGFSYAPPTDSFL--

>H glaber

MTVKTEAARGTVTYSRMRGMVAILIAFMKQRRMGLNDFIQ-KIANNSYACKHPEVQSILK

ISQPQEPELMNANPSPPPSPSQQINLGPSSNPHAKPSD---------FHFLKVIGKGSFG

KVLLARHKAEEAFYAVKVLQKKAILKKKEEKHIMSERNVLLKNVKHPFLVGLHFSFQTAD

KLYFVLDYINGGELFYHLQRERCFLEPRARFYAAEIASALGYLHSLNIVYRDLKPENILL

DSQGHIVLTDFGLCKENIEHNGTTSTFCGTPEYLAPEVLHKQPYDRTVDWWCLGAVLYEM

LYGLPPFYSRNTAEMYDNILNKPLQLKPNI-TNSARHLLEGLLQKDRTKRLGAKDD-FME

IKSHVFFSLINWDDLINKKITPPFNPNVSGPSDLRHFDPEFTEEPVPNSIGRSPDSILIT

ASVKEAAEAFLGFSYAPPMDSFL--

>M brandtii

MTVKTEAARGTLTYSRMRGMVAILIAFMKQRRMGLNDFIQ-KIANNSYACKHPEVQSILK

ISQPQEPELMNANPSPPPSPSQQINLGPSSNPHAKPSD---------FHFLKVIGKGSFG

KVLLARHKAEEAFYAVKVLQKKAILKKKEEKHIMSERNVLLKNVKHPFLVGLHFSFQTAD

KLYFVLDYINGGELFYHLQRERCFLEPRARFYAAEIASALGYLHSMNIVYRDLKPENILL

DSQGHIVLTDFGLCKENIEPNGTTSTFCGTPEYLAPEVLHKQPYDRTVDWWCLGAVLYEM

LYGLPPFYSRNTAEMYDNILNKPLQLKPNI-TNSARHLLEGLLQKDRTKRLGAKDD-FME

IKNHVFFSVINWDDLINKKITPPFNPNVSGPSDLRHFDPEFTEEPVPNSIGRSPDSILLT

ASVKEAAEAFLGFSYAPPVDSFL--

>S franciscanus

---------QDARSEIIPYPVIPILFF----SGEVQNFLSLDNPRNNPDNNVTDENNIDF

FSDPNDPN--------------TINLGPSANPSARPSD---------FDFLKVIGKGSFG

KVLLAKHKIDGKVYAIKVLKKAAIIQDHDAKHIMTEKSVLLKASKHPFLVSLHYSFQTAD

KLYFVLEYVNGGELFFHLQREKKFPEERARFYAAEIASALAYLHAHGIIYRDLKPENILL

DADGHVKLADFGLCKEGIAAKKTTSTFCGTPEYIAPEVIEEKGYGHAVDWWALGVVLYEM

LVGTPPFYTDSIEQLFSLILNKPLKMPSSLFSPAAKSLLSELLEKKPEKRI---------

---ISFCIAPPLAKIILLKAIVKYVSLQSDSMDVKNIDPEFVREPVPNSVGKSVDPNTVS

ASVQDADNVFEGFTYVPPTVGLDDP

>S purpuratus

----------MSVLSKGAGTVQKRGKFL---CGEVQNFLSLDNPRNNPDNSVADENNTDF

FSDPNDPN--------------TINLGPSANPSARPSD---------FDFLKVIGKGSFG

KVLLAKHKIDGKVYAVKVLQKAAIVKRNEAKHIMAERSVLLKNLKHPFLVSLHYSFQTAD

KLYFVLDYVNGGELFFHLQREKSFPEVRAKFYAAEIASAIGYLHSLDIIYRDLKPENILL

DHDGHVRLTDFGLCKEGIAAKKTTSTFCGTPEYLAPEVLRKQEYDRSVDWWCLGAVLFEM

MSGLPPFYSRDTAEMYDNILNKPLRMRSSVFSPSAKGLLEGLLHKERAKRLGAGDKGFKA

VKHHEFFSSISWSDLDGKKITPPYNPRVSDSMDVKNIDPEFVREPVPNSVGKSVDPNTVS

ASVQDADNVFEGFTYVPPTVGLDDP

>L variegatus

--------LLKLLTSHIKKLLFPILF-----SGEVQNFLSLDDPRNNSDNSVTDENNTDF

FSDPNDPN--------------TIFLGPSANPSARPSD---------FDFLKVIGKGSFG

KVLLAKHKIDGKVYAIKILQKAAIIQRHQAKHIMAEKHVLETASKHPFLVSLHSCFQTDD

RLYFVMEYVSGGDLFFHIQRQRRFDEETARFYAAEIVSALEYLHSLGIVYRDLKPENILL

DADGHVKLADFGLCKEGIGYGRTTSTFCGTPEYLAPEIILEQGYDRSVDWWSLGILLFEM

LSGLPPFHGETPDQLFRCIIHGKWRMLLKSIDFVCLG-IALLLHKERAKRLGAGEKGFKT

VKHHEFFSSISWSDLDAKKITPPYNPRVSSTMDVKNIDPEFVREPVPNSVGKSVDPNTVS

ASVQDADNVFEGFTYVPPTVGLDDP

>M musculus

MTVKAEAARSTLTYSRMRGMVAILIAFMKQRRMGLNDFIQ-KIASNTYACKHAEVQSILK

MSHPQEPELMNANPSPPPSPSQQINLGPSSNPHAKPSD---------FHFLKVIGKGSFG

KVLLARHKAEEVFYAVKVLQKKAILKKKEEKHIMSERNVLLKNVKHPFLVGLHFSFQTAD

KLYFVLDYINGGELFYHLQRERCFLEPRARFYAAEIASALGYLHSLNIVYRDLKPENILL

DSQGHIVLTDFGLCKENIEHNGTTSTFCGTPEYLAPEVLHKQPYDRTVDWWCLGAVLYEM

LYGLPPFYSRNTAEMYDNILNKPLQLKPNI-TNSARHLLEGLLQKDRTKRLGAKDD-FME

IKSHIFFSLINWDDLINKKITPPFNPNVSGPSDLRHFDPEFTEEPVPSSIGRSPDSILVT

ASVKEAAEAFLGFSYAPPVDSFL—

SIRT1

>H sapiens

MADEAALALQPGGSPSAAGADREAASSPAGEPLRKRPRRDGPGLERSPGEPGGAAPEREV

PAAARGCPGAAA---AALWREAEAEAAAAGGEQEAQATAAAGEGDNGPGLQGPSREPPLA

DNLYDEDDDDEGEEE---EEAAAAAIGYRDNLLFGDEIITNGFHSCESDEEDRASHASSS

DWTPRPRIGPYTFVQQHLMIGTDPRTILKDLLPETIP-PPELDDMTLWQIVINIL---SE

PPKRKKRKDINTIEDAVKLLQECKKIIVLTGAGVSVSCGIPDFRSRDGIYARLAVDFPDL

PDPQAMFDIEYFRKDPRPFFKFAKEIYPGQFQPSLCHKFIALSDKEGKLLRNYTQNIDTL

EQVAGIQRIIQCHGSFATASCLICKYKVDCEAVRGDIFNQVVPRCPRCPADEP-LAIMKP

EIVFFGENLPEQFHRAMKYDKDEVDLLIVIGSSLKVRPVALIPSSIPHEVPQILINREPL

PHLHFDVELLGDCDVIINELCHRLGGEYAKLCCNPVKLSEITE-----KPPRTQKELAYL

SELPPTPLHVSED-----------------------------------------------

-------------------------------------------------------SSSPE

RTSPPDSSVIVTLLDQAAKSN-DDLDVSESKGCMEEKPQEVQTS-RNV-------ESIAE

QMENPD-------------------------------------------LKNVGSSTGEK

NERTSVAGTVRKCWPNRVAKEQISRRLDGNQYLFLPPNRYIFHGAEVYSDSEDDVLSSSS

CGSNSDSGT---CQSPSLEEPMEDESEIEEFYNGLEDEPDVPERAGGAGFGTDGDDQEAI

NEAISVKQE---------------------------------------------------

------------------------------------------------------------

-----VTDMNYPSNKS--------------------------------------------

-----------------------

>H_glaber

MADEAALTLQP-GSPSAAAAEREAASPPAGEPLRKRPRREGSVPGRSPSEPSG-------

-AAAGGCP-AAA---AALWREA---AAVAGGEREAQGTAGVGEGDNGPGLQGLALELPPG

DDFDDEDDDDDDDDDDEGEEEEEAAIGYRDNLLLSDDIITNGFHSCESDDDDRASHASSS

DWTPRPRI--------------------------------------VWQIVINIL---SE

PPKRKKRKDINTIEDAVKLLQECKKIIVLTGAGVSVSCGIPDFRSRDGIYARLAVDFPDL

PDPQAMFDIEYFRKDPRPFFKFAKEIYPGQFQPSLCHQFIALSDKEGKLLRNYTQNIDTL

EQVAGIQRIIQCHGSFATASCLICRYKVDCEAVRGDIFNQVVPRCPRCPPDEP-LAIMKP

EIVFFGENLPEQFHRAMKYDKDEVDLLIVIGSSLKVRPVALIPSSIPHEVPQILINREPL

PHLHFDVELLGDCDVIINELCHRLGGEYAKLCCNPVKLSEITE-----KPPRTQKELVHL

SQLPPTPLHISED-----------------------------------------------

-------------------------------------------------------SSSPE

RTSP-DSSVIVKLLDQATKSSVDDLDGAESKGYVEEKPQEVQASTRNA-------ESI--

NVENPD-------------------------------------------LKD-SSNTAEK

SGRTSVAETVRKCWPNRLAKEQISKRLDGNQYLFLPPNRYIFHGAEVYSDSEDDVLSSSS

CGSNSDSGT---CQSPSLEEPLEDESEIEEFYNGLEEDPDAPEITGGTGFGANGGDQEAV

NEAISTKQE---------------------------------------------------

------------------------------------------------------------

-----VTDRNYPSNKS--------------------------------------------

-----------------------

>M_brandtii

MAN-----LKS-GRVTKLSLTREEMS----RGVVRGQHWEGEVPFGFPSESQG-------

---------FST---KML------------------------------------------

-----------------------------DNLLFGDEIITNGFHSCESDEDDRASHASSS

DWTPRPRIGPYAFVQQHLMIGTDPRTILKDLLPETIP-PPEWDDMTLWQIVINIL---SE

PPKRKKRKDINTIEDAVKLLQECKKIIVLTGAGVSVSCGIPDFRSRDGIYARLAVDFPDL

PDPQAMFDIEYFRKDPRPFFKFAKEIYPGQFQPSLCHKFIALSDKEGKLLRNYTQNIDTL

EQVAGIQRIIQCHGSFATASCLICKYKVDCEAVRGDIFNQVVPRCPRCPADEP-LAIMKP

EIVFFGENLPEQFHRAMKYDKDEVDLLIVIGSSLKVRPVALIPSSIPHEVPQILINREPL

PHLHFDVELLGDCDVIINELCHRLGGEYAKLCCNPIKLSEITE-----KPPRTQKELAHL

SELPPTPLNISEG-----------------------------------------------

-------------------------------------------------------SSSPE

RTSPPDSSVIVTLLDHAMKSNIDDPDVSESKDCMEEKSQAVQTSTRSI-------ESVTE

QLESPD-------------------------------------------LKNISSNTVEK

NERTSVAETVRKYWPAKIAKEQISKRLDGNQYLFLPPSRYIFHGAEVYSDSEDDVLPSSS

CGSNSDSGT---CQSPSLEEPLEDESEIEEFYNGLEDDPDINERAGGTEFGADEGDQEAV

NEAISMKQE---------------------------------------------------

------------------------------------------------------------

-----ATDINYPSNKS--------------------------------------------

-----------------------

>S franciscanus

---------------------------------------FIPICL-YPLQP---------

------------------------------------------------------------

------------------------------------------------------------

--------GPMGWLQKQMMSGTNPRSILMRIIPSGMTIPEEMDEFEMWSIIAEYLRSIDE

PPPRQKLEQYNTFDDAIQLLKSCKNILVIYGAQVSVSIAIPDFRSRDGVYARLAVDFPDL

PDPQAMFEISYFRKDPRPFYKFAQELFPGQFKPSTSHKFISQLEQQQKLLRNYTQNIDTL

EQAAGISGVIQCIGKFATATCTRCGLSVDSDAIRDDVFNQVVPICPQCGPYTPQMAVLKP

DIVLFIETYSYHSYDKLNDDKETADLLIVMGSSLKVRPVATIASLLPKKVPQILINREPL

PHLNFDIELLGDCDVIINEILHRLGDGWNHVCQSQQRLTETTYIPNVSKPKEPQQAQKAV

LDEGETTVDQKPD-----------------------------------------------

-------------------------------------------PAGRIDNEVSASNPVNV

KSEEDSQQSLSSLKEESQVYSGDDKNEGSNAGEGDDRLASSNSQESPILPEGPVKQSCDE

RLQAPENQTSSEHHINGLTNEGKVHVDALPCSQSKTNYSPSSMSDSLQSVDEVENALVSS

EPQTSKTAPSESSQSQTAHDQTVQRNSAAEQYMLFQRTLDLIGNSSTGASSSDVKVGNDS

VATGESESTQTHVDSDCKEPSTDVKTEAEELGSCVNEKDRTKSQDAKDDIDSEVGNVSSS

SGSVVDHREQASPAPDSEAKPSPSTSAAAKTPRATISTHLK-------------------

------------------------------------------------------------

-----GKDQHSFRNSEIVNV----------------------------------------

----TFITYLPIYLTNVWNFNFY

>S purpuratus

---------------------------------------MGLGDIRPDSLA---------

------------------------------------------------------------

------------------------------------------------------------

--------GPMGWLQKQMMSGTNPKSILMRIIPSGMTIPEEMDEFEMWSIIAEYLRSIDE

PPPRQKLEQYNTFDDAIQLLKTCKNILVLTGAGVSVSCGIPDFRSRDGVYARLAVDFPDL

PDPQAMFEISYFRKDPRPFYKFAKELFPGQFKPSTSHKFISQLEEHQKLLRNYTQNIDTL

EQAAGIKGVIQCHGSFATATCTRCGLSVDSDAIRDDVFNQMIPICPQCGPDTPDMAVLKP

DIVFFGEGLPNHFYDKLNDDKETADLLIVMGSSLKVRPVATIPSLLPKKVPQILINREPL

PHLNFDIELLGDCDVIINEILHRLGDGWDHVCQSQQRLTETTHIPNGFKPKESRQARKAV

LEEGGTTVDQRPDPVLDEGETTVDQRPDPVLDEGETTVDQRSNPVLDEGETTVDQKPDPV

LDEGETTVDQKPDPVLDEGETTVDQKPDPVLDEGETTVDQKPDPAGRIDQEESVSNPVNV

KSKEDTQHSLSSLKEEGQVSSGDDKNEGSKAGEGDDRLARSNSQDSPISPEGHVKQSCDE

RLQAPENQTSLEHHINGLTNEGKIHVDALPCSRSETNCSPSSNSDRLQSVDEVENALASS

EPQTSKTIPSESSQSHTAQDQTAQRNSAAEQYMLFQRTLDSIGNSSTGASSLDVMAGNDS

VASGESESTQTHVASDCKEPSTDVKAEAEDLGLCVNETDRTNSQ---DDLNSEVGNVSSS

SGSVLEHREQASPAPDSEAKPSPSTSAAAKTPRATISTQLKESSFLFIPPMRYVFHGAEV

FLSDDDNEIEHGLEGLNKDMDINDLHNELENGDLHNDLGSGDLHNELENGDLHNHLHNGD

DLPDLSEEDHSPAKEKTVSLDDLTPTVPSTDRETSQYGQSASKAQCVPETNGRDGSPFEA

PSGETVYTHIDLPVTP-------

>L variegatus

---------------------------------------HIAIHSTYTTPP---------

------------------------------------------------------------

------------------------------------------------------------

--------GPMGWLQKQMMSGTNPRSILMRIIPSGMTIPDDMDEFEMWSIIAEYLRSIDE

PPPRQKLEQYNTFDDAIQLLNSCKNILVLTGAGVSISCGIPDFRSRDGVYARLAVDFPDL

PDPQAMFEISYFRKDPRPFYKFAKELFPGQFKPSPSHKFISQLEQHDKLLRNYTQNIDTL

EQCAGITRVIQCHGSFATATCTRCGLRVDSDAIREDVF-KVVPVCPQCGPDTPEMAVLKP

DIVFFGEGLPNHFYDKLNDDKEAADLLIVMGSSLKVRPVATIPSLLPKDVPQILINREPL

HHLNFDIELLGDCDVIINEILHRLGDGWNHVCQSQQRLTETTDIPNIPKPKEANKDVHTL

LDKSESTVEEKPN-----------------------------------------------

------------------------------------------------------------

------------------------------------------------------------

------------------------------------------------------------

------------------------------------------------------------

------------------------------------------------------------

------------------------------------------------------------

------------------------------------------------------------

-----VTS----------------------------------------------------

-----------------------

>M musculus

MADEVALALQAAGSPSAAAA-MEAASQPADEPLRKRPRRDGPGLGRSPGEPSA-----AV

APAAAGCEAASAAAPAALWREA--AGAAASAEREAPATAVAGDGDNGSGLR---REPRAA

DDFDDDEGEEEDEAA---AAAAAAAIGYRDNLLLTDGLLTNGFHSCESDDDDRTSHASSS

DWTPRPRIGPYTFVQQHLMIGTDPRTILKDLLPETIP-PPELDDMTLWQIVINIL---SE

PPKRKKRKDINTIEDAVKLLQECKKIIVLTGAGVSVSCGIPDFRSRDGIYARLAVDFPDL

PDPQAMFDIEYFRKDPRPFFKFAKEIYPGQFQPSLCHKFIALSDKEGKLLRNYTQNIDTL

EQVAGIQRILQCHGSFATASCLICKYKVDCEAVRGDIFNQVVPRCPRCPADEP-LAIMKP

EIVFFGENLPEQFHRAMKYDKDEVDLLIVIGSSLKVRPVALIPSSIPHEVPQILINREPL

PHLHFDVELLGDCDVIINELCHRLGGEYAKLCCNPVKLSEITE-----KPPRPQKELVHL

SELPPTPLHISED-----------------------------------------------

-------------------------------------------------------SSSPE

RTVPQDSSVIATLVDQATNNNVNDLEVSESS-CVEEKPQEVQTS-RNV-------ENI--

NVENPD-------------------------------------------FKAVGSSTADK

NERTSVAETVRKCWPNRLAKEQISKRLEGNQYLFVPPNRYIFHGAEVYSDSEDDVLSSSS

CGSNSDSGT---CQSPSLEEPLEDESEIEEFYNGLEDDTERPECAGGSGFGADGGDQEVV

NEAIATRQE---------------------------------------------------

------------------------------------------------------------

-----LTDVNYPSDKS--------------------------------------------

SIRT2

>H sapiens

----------------------------------MDFLRNL---FSQTLSLGSQKER---

--LLDELTLEGVARYMQSERC--RRVICLVGAGISTSAGIPDFRSPSTGLYDNLEKYHLP

YPEAIFEISYFKKHPEPFFALAKELYPGQFKPTICHYFMRLLKDKGLLLRCYTQNIDTLE

RIAGLEQEDLVEAHGTFYTSHCVSASCRHEYPLSWMKEKIFSEVTPKCEDCQ--SLVKPD

IVFFGESLPAR------FFSCMQSDFLKVDLLLVMGTSLQVQPFASLISKAPLSTPRLLI

NKEKAGQSDPFLGMIMGLGGGMDFDSKKAYRDVAWLGECDQGCLALAELLGWKKELEDLV

RREHAS-------IDAQSGAGVPNPSTSAS----------PKKSPPPAKDEARTTEREKP

Q-----

>H glaber

--------MRSTRAHTVKDTDLDAEEGASGGEAEMDFLRSL---FSQTLGLGSQKER---

--LLDELTLEGVVRYMQSERC--QRVICLVGAGISTSAGIPDFRSPTTGLYANLEKYHLP

YPEAIFEIGYFKKHPEPFFALAKELYPGQFKPTICHYFIRLLKEKGLLLRCYTQNIDTLE

RVAGLEPEDLVEAHGTFYTSHCTSQLCRHEYTLGWMKEKLFSEVTPKCEKCQ--SVVKPD

IIFFGENLPSR------FFSCLQSDFRKVDLLIIMGTSLQVQPFASLIGKAPLSTPRLLI

NKEKTGQTDPFLGMMMGLGGGMDFDSKKAYRDVAWLGDCDQGCLALADLLGWKKELEDLV

RKEHAH-------IDAQSGSSTPNPTTAAS----------PRTSPPPGKAEAKTTDGEKP

Q-----

>M brandtii

PPASDPLETQAGKVQEAQDSDSDTEEGAAGGEAEMDFLRNL---FSQTLGLGTQKER---

--LLDNLTLEGVAHYMLSERC--RRVICLVGAGISTSAGIPDFRSPSTGLYANLQKYHLP

YPEAIFEIGYFKKHPEPFFALAKELYPGQFKPTLCHYFIRLLKEKGLLRRCYTQNIDTLE

RVAGLEPEDLVEAHGTFYTSHCISPLCRKEYTLSWMKEKIFSEVTPKCENCQ--SVVKPD

IVFFGENLPAR------FFSCIQSDFLNVDLLIIMGTSLQVQPFASLISKAPLSTPRLLI

NKEKTGQTDPFLGMMMGLGGGMDFDSKKAYRDVAWLGDCDQGCLALADLLGWKKELEDLV

RKEHAS-------IDAQSGLGSPNPTTSAS----------PRKSPPAAKEEARTTEGEKP

Q-----

>S franciscanus

------------------------------------------------------------

------------------------------------------------------------

--------------PEPFFTLSKELFPGAFYPTPSHFFIRLLHEKGILQRHYTQNIDTLD

RIAGVPDELIMEAHGSFHTGHCLN--CNEIYTEESMRGKIRHIL----------------

----------------------------------------------MLYKDHISSPQI--

---------SFL------------------------------------------------

------------------------------------------------------------

------

>S purpuratus

--------MSAKNEGAAAGADDKEGGQSESVDSQVESLRNFLGRFHLSAGSSGQEEKPKP

EQLLKELTLEGIADFIKEGKC--KKVIVMSGAGISTSAGIPDFRTPGTGLYDNLQKYNLP

NPQAIFEIGFFKQNPEPFFTLSKELFPGAFYPTPSHFFIHLLHEKGILLRHYTQNIDGLD

RMAGVPDELIMEAHGSFHTGHCLN--CNEMYTEESMREKIMADLIPRCAKCNETGVVKPD

VVFFGESLPPR------FPTLVSEDFPQCDLLIVMGTSLVVQPFASLIDKVPETTPRLLI

NMEKSGKADPMM-MMFGFPSGMDFDSDDKYRDVAYIGPCDEGCEKLAGFVGWKKEMTVLV

NKGEAK-------KKKKGETESPTKDETAKTPTSATKDQKPKTSASPGKKSSASPTKDKK

PSSKKD

>L variegatus

--------------------TYFSGIIRLISIIQSNIISSL---CHNSLPMNNSIMT---

--KFIHLACHVSHAISIPYICIYHLLISQLTCILSIAAGIPDFRSPGTGLYDNLQKYNLP

NPQAIFEIGFFQENPEPFFTLSKELFPGAFFPTPSHFFIRLLHEKGILLRHYTQNIDTLD

RIAGVPDEKIMEAHGSYHTGHCLN--CNEEYTEELMRGKHKKVLNMYIAAMF--------

LAFSGGRVGMKVVIEYCYKTCFFADFPQCDLLIVMGTSLVVQPFASLVDKYVITSTTFPL

MTKTSSYIHQLCSLALKLQIIIAPPPPAYYCNLVY------EVLNFIKSFSFITYFILLI

YANYNSAVNLMKYFTATSFILVTGDSDEMYMKKLVLKCILLFLHSLPESSLNGCPLQ---

------

>M musculus

----------------------------------MDFLRNL---FTQTLGLGSQKER---

--LLDELTLEGVTRYMQSERC--RKVICLVGAGISTSAGIPDFRSPSTGLYANLEKYHLP

YPEAIFEISYFKKHPEPFFALAKELYPGQFKPTICHYFIRLLKEKGLLLRCYTQNIDTLE

RVAGLEPQDLVEAHGTFYTSHCVNTSCRKEYTMGWMKEKIFSEATPRCEQCQ--SVVKPD

IVFFGENLPSR------FFSCMQSDFSKVDLLIIMGTSLQVQPFASLISKAPLATPRLLI

NKEKTGQTDPFLGMMMGLGGGMDFDSKKAYRDVAWLGDCDQGCLALADLLGWKKELEDLV

RREHAN-------IDAQSGSQAPNPSTTIS----------PGKSPPPAKEAARTKEKEEQ

Q-----

TEP1

>H sapiens

MEKLHGHVSAHPDILSLENRCLAMLPDLQPLEKLHQHVSTHSDILSLKNQCLATLPDLKT

MEKPHGYVSAHPDILSLENQCLATLSDLKTMEKPHGHVSAHPDILSLENRCLATLPSLKS

TVSASPLFQSLQ---ISHMTQADLYRVNNSNCLLSEPP-SWRAQHFSKGLDLSTCPIALK

SISATETAQEATLGRWFDSEEK-----KGAETQMPSYSLSLGEEEEV-EDLAVKLTSGDS

ESH--------------PEPTDHVLQEKKMALLSLLCSTLVSEVNMNNTSDPTLAAIFEI

CRELALLEPEFILKASLYARQQLNVRNVANNILAIAAFLPACRPHLRRYFCAIVQLPSDW

IQVAELYQSLAEGDKNKLVPLPACLRTAMTDKFAQFDEYQL--AKYNPRKHRAKRHPRRP

PRSPGMEPPFS--HRCFPRYIGFLREEQRKFEKAGDTVSEKKNPPRFTLKKLVQRLHIHK

PAQHVQALLGYRYPSNLQLFSRSRLPGPWDSSRAGKRMKLSRPETWERELSLRGNKASVW

EELIENGKLPFMAMLRNLCNLLRVGISSRHHELILQRLQHAKSVIHSRQFPFRFLNAHDA

IDALEAQLRNQA----------------------------------------LPF---PS

NITLMRR--------ILTRNEKN-RP---RRRFLCHLSRQQLRMAMRIP----------V

LYEQLKREKLRVHKA---------------------------------------------

--------------------------------------------------RQWKYDGEML

NRYRQALETAVNLSVKHSLPLLPGRTVLVYLTDANADRLCPKSNPQG-------------

--------PPLNYALLLIGMMIT-RAEQVDVVLCGGDTLKTAVLKAEEGILKTAIKL-QA

QVQEFDENDG--------------------------------------------------

------------------------------------------------------------

--------------WSLNTFG---------------------------------------

----------------------------------------KYLLS--LAGQRVPVDRVIL

LGQSM-----DDGMINVAKQLYWQRVNSKCLFVGILLR--RVQYLSTDLNPNDVTLSGCT

DAILKFIAEHGASHLLEHVGQMDKIFKIPPPPGKTGVQSLRPLEEDTPSPLAPVSQQGWR

SIRLFISSTFRDMHGERDLLLRSVLPALQARAAPHRISLHGIDLRWGVTEEETRRNRQLE

VCLGEVENAQLFVGILGSRYGYIPPSY-NLPDHPHFHWAQQYPSGRSVTEMEVMQFLNRN

QRLQPSAQALIYFRDSSFLSSVPDAWKSDFVSESEEAARRISELKSYLSRQKGITCRRYP

CEWGGVAAGRPYVGGLEEFGQLVLQDVWNMIQKLYLQPGALLEQPVSIPDDDLVQATFQQ

LQKPPSPARPRLLQDTVQRLMLPHGRLSLVTGQSGQGKTAFLASLVSALQAPDGAKVAPL

VFFHFSGARPDQGLALTLLRRLCTYL---RGQLKEPGALPS--TYRSLVWELQQRLLPKS

AESLHPGQTQVLIIDGADRLVDQNGQLISDWIPKKLPRCVHLVLSVSSDAGLGETLEQSQ

GAHVLALGPLEASARARLVREELALYGKRLEESPFNN-----------------------

--------------QMRLLLVKRE-SGRPLYLRLVTDHLRLFTLYEQVSERLRTLPATVP

LLLQHILSTLEKEHGPDVLPQALTALEVTRSGLTVDQLHGVLSVWRTLPKGTKSWEEAVA

AGNSGDP-YPMGPFACLVQSLRSLLGEGPLERPGARLCLPDGPLRTAAKRCY--GKRPGL

EDTAHILIAA---QLWKTCDADASGTFRSCPPEALGDLPYHLLQSGNRGLLSKFLTNLHV

VAAHLELGLVSRLLEAHALYASSVP-----KEEQKLPEADVAVFRTFLRQQASILSQYPR

LLPQQAANQPLDSPLCHQASLLSRRWHLQHTLRWLNKPRTMKNQQSSSLSLA-VSSSPTA

VAFSTNGQRAAVGTANGTVYLLDLRTWQEEKSVVSGCDGISACLFLSDDTLFLTAFDGLL

ELWDLQHGCRVLQTKAHQYQITGCCLSPDCRLLATVCLGGCLKLWDTVRGQLAFQHTYPK

SLNCVAFHPEGQVIATGSWAGSISFFQVDGLKVTKDLGAPGASIRTLAFNVPGGVVAVGR

LDSMVELWAWREGARLAAFPAHHGFVAAALFLHAGCQLLTAGEDGKVQVWSGSLGRPRGH

LGSLSLSPALSVALSPDGDRVAVGYRADGIRIYKISSGSQGAQGQALDVAVSALAWLSPK

VLVSGAEDGSLQGWALKECSLQSLWLLSRFQKPVLGLATSQELLASASEDFTVQLWPRQL

LTRPH-KAEDFPCGTELRGHEGPVSCCSFSTDGGSLATGGRDRSLLCWDVRTPKTPVLIH

SFPACHRDWVTGCAWTKDNLLISCSSDGSVGLWDPESGQRLGQFLGHQSAVSAVAAVEEH

VVSVSRDGTLKVWDHQGVELTSIPAHSGPISHCAAAMEPRAAGQPGSELLVVTVGLDGAT

RLWHPLLVCQTHTLLGHSGPVRAAAVSETSGLMLTASEDGSVRLWQVPKEADDTCIPRSS

AAVTAVAWAPDGSMAVSGNQAGELILWQEAKAVATAQAPGHIGALIWSSAHTFFVLSADE

KISEWQVKLRKGSAPGNLSLHLNRILQEDLGVLTSLDWAPDGHFLILAKADLKLLCMKPG

DAPSEIWSSYTENPMILSTHKEYGIFVLQPK-DPGVLSFLRQKESGEFEERLNFDINLEN

PSRTLISITQAKPESESSFLCASSDGILWNLAKCSPEGEWTTGNMWQKKANTPETQTPGT

DPSTCRESDASMDSDASMDSEPTPHLKTRQRRKIHSGSVTALHVLPELLVTASKDRDVKL

WERPSMQLLGLFRCEGSVSCLEPWLGANSTLQLAVGDVQGNVYFLNWE

>H_glaber

MEKHHGPVSVHPDILSLENR------------------------------CLAMLPNLQP

MERPRGYMPAHSDILCLENRCLARISDLKSTGKPHGHGAAHPSILSVENGCLSSIPSVKS

SMSASPLLQCLQ---PPCMMHANVSSLNTSKCLLSEPPSSRRAQC---------------

-VSATETVQGALC---YSKEEKKSEAMEGDGDQMPIYSLSLGEEEEVEEELALQLASGHS

ESH--------------PETSDWDLQEKKMCLMTILCSIVASNVNMNNARDLSR-SIHCI

CSELSSLEPEFILKESLYARQQLNLRDVANKVLAIAALLPACRPHLRRYFCAIVHLPSDW

IQVAEFYQDLAKGDENKLVPLPACLRAAMTDKFAQFDEYQL--AKYNLRQHRAKRRPRRP

PRPPKTEPPFSG-RLHVLGFLWSLTDVQTKFEVAYDA--EKKNPSWFTLKKLVQRLHIHK

PAQHVQALLGYRYPSSLQLFSQSRLPGPWDSSRAGKRMKLPRPETWDRKLSLQGNKASVW

EELIDSGKLPFMAMLRNLCNLLRVGISARHHELILQRLQDTKSVIHSRQFPFRFLNAHDA

IDNLEAQLRNKA----------------------------------------LPF---PS

NTQLMRR--------IMTRNAKSIRK---YPRYLCNLRRSQLRTAMMVP----------V

LYEQLKREKLKIRKA---------------------------------------------

--------------------------------------------------RQWKCDTEML

GRYRQALEVAVNLSVRHSLPPLPGRTLLVYMTDSRADLLCPKSNTQG-------------

--------PPLNYVLLLIGMMMA-RAEQAEVLLCGRGTVKTAVLVAEDAILKTAITL-QA

QVQEMEEVDE--------------------------------------------------

------------------------------------------------------------

--------------CPLTTFE---------------------------------------

----------------------------------------KYLLS--LAVQRVPVDRVVL

FGEML-----RSPIVTVAKHIIWKHVNPKCLFVDVRV---RKPYPYSEFHPNHVTLSGCT

DGILKFIAESGASRLLEHVDQMDRIFRIPPAPGKTQVLTLRPLEVDAPSPLAPVFQHGWR

SIRLFISSTFRDMHGERDLLLRSMLPALQARAAPHRISLHAIDLRWGVTEEETRRNRQLE

VCLGEVENSQLFLGILGSRYGYVPPSY-DLPDHPHFHWVQQYPPGRSVTELEVMQFLNRD

RCAQPSAQALIYLRDSSFLSSVPDMWRPDFVSESEEAAQRISELKSYLSTRKEITCRGYS

CEWGGTAAGRPYAGGLEEFGQLVLQDVWSVIQKLYLQPGAELEQPVSIPDDDLVQATFQE

LRSPARPARLRLLQDTVRQLVLPHGKLSLVTGQSGQGKTTFLASLVSALRAPVGAKVAPL

VFFHFSGARPDQSLALSLLRRLCSYL---HSKLHKVGALPR--THRGLVWELQQKLLPES

AQTLQPGQTLVLIIDGADKLVDQNGQLISDWIPQSLPRWVHLVLSVSSDSGLGETLEQSQ

GAYVVALGPLAPSARAQLVRDELALYGKRLDESPFNN-----------------------

--------------QMQLLLVKRG-SGLPLYLRLVTDHLRLFTLYEQVSERLRTLPATVP

LLLDLILSTLEQEHGHDVLPQTLATLEVTRSGLTEDQLHAVLSAWQILPKGTKSWEEAVA

AGNSAEP-YPMGLFAYLVQSLCSLLGEGPLERPGARLCLRDGALKTAVKRRY--GKRRGL

QHMAHVLIAA---QLWKTCNPDASGSFQSCLPEALGDLPFHLLHSGNHALLAKFLSSLPV

VAAHLDLGLVPQLLEAHTLYASSVP-----EEDQKLPEADVAAFCTFLRQQAPVLSRYPL

LLRQQAANQPADSPLCFQAPLLSQQWHRWPILRWLNKPQTVEGQQSSSLSLV-ISSTPTA

VAISFNGQRAAVGTASGMVYLLDLRTWQEEKSVVSGCDGISSCAFLSDNALFLTAFDGLL

ELWDMQRGCRVLQTKAHQYQITGCCLSPDQRLLATVCLGGCLKLWDTVRGQLSFQHTSAK

PLNCTAFHPEGQAIAIGSWAGSVSFFQVDGLKATQDLGAPGASVRTLAFSATGQLVAVGR

LDRTVELWAWQEGSRLAGFPAHCGFVSAVLFLHAQSQLLTAGEDGKVQVWSSSLGRPWGC

LGSLRLSPALSVALSPDRDWVAVGYRKDGIKIYKIPAGSQQVLCQMVDVAVSALAWLSPK

VLVSGAEDGTLQGWVLKNRVLHSLWLLPRYQRPVLGLAISQELLASASEDFTVRLWPKQL

LTQWH-KTEDFPCGTELRGHEGPVSCCSFSMDGGSLATGGRDRSLLCWDVRTPQSPLLIR

SFPACHRDWVTGCAWTKDNLLISCSSDGSVGLWDPESGQQLGHFLGHQSAVSAVVAVEEH

VVSVGRDGTLKVWDHGGVELTSIPAHSGPISHCAAALDPHPAGQPGSELLVVTVGLDGAP

RLWHPLSVYHTHTLLGHSGSISAAAVSESSGLLLTASEDGSVRLWQVPKQAEGTNVPRSS

AAITAIAWATDGSLAVSGNQAGELTLWQQAKAVATAQAPGRVSAVIWLSAYTFYVLSADE

KISRWDVEFESDLTSTTFSLCLSQVLQEDLGVLTSMDLAPDGHSLILVKANMEILHLQPG

NAPSIIGNRVGIHPILLSTHKEYGVFHLNTRYHLQCLCFLQQKESGEVKDHLDFLLNLEN

PSGKSRKITQAKPESESSFLCASSDGELWRLAGCTFEGEWTTGNIWQKKVAAPEVQESGT

DLSCTS-----MYNWVM-----ELDLKTQLHRKIHSDSVTALHVLPELLVTASKDRDVKL

WDRPSMQLLGLFRCEGAVSCLEPCLRPDSTLQLAVGDTQGNVYFLSWE

>M brandtii

MEKLYGHVSAHPDILSLENR------------------------------CLATLPDVKI

MEKPHGHVSSHPDILSLENRCLATLPDMRLMEKPHGHVSAHPDILSLENRCLATLSILKS

PMSASSFLQCVQ---TSQLVQADLCSLDTSNHLLSEPP-TWRTQCFSEGPGPLTCPRALK

SISATQGAQETALGHWSSSEEKKPGEKKWTEAQMPFYSLSLGEEEEV-EEMALKLTPGDS

ESS--------------SEPTDQVLQEKKMALMSLLCSTVASKVNMKDEANPTKTALLEI

CSELAPLEPEFILKASLYARQQLNIRDIANLTLAIAAFLPVCRPHLRRYFCAIVQLPSDW

IQVAEFYQSLAEGEEDKLVALPACLRAAMTDKFTQFDEYQL--AKYNPRKHRAKRRPHRP

PRPPKTERPFSETQKYLPKYLRFLKNEQEKFEESYNAVPERKKPPRFTLKKLVQRLHIQE

PAQHVHALLGYRYPSNLQVFSRSRLPGPWDSGRAGKRMKLSRPETWERELSLRGNKASVW

EELIDNGKLPFMAMLRNLCNLLRVGISAHHHELVLQRLQQA-----------VFVRGREP

EE--------------------------------------------------LPF---PS

NTQLMRR--------IMIRHSRNVKR-RVYQRQLQNLNRRKLRAAMMMP----------V

FYEQLKREQLRVHRT---------------------------------------------

--------------------------------------------------RQWKYDREML

ARYRQALETAVNLSVKHSLPPLPGRTLLVYLTDADADKICPKSNPEG-------------

--------PPLNYVLLLMGMMIA-RAEHADLLLCGKGALKPAVIKAEDGILRTALEL-QA

QVQELDGDYE--------------------------------------------------

------------------------------------------------------------

--------------WSLHTLG---------------------------------------

----------------------------------------KYLLS--LAAQRVPVDRVIV

FGQAM-----NEKLINKAKQLFWQHVNSKCLFVGVLLR--RTCDTLQDLNPNDVTLSGCT

DGILKFIAERGASRLLEHVGQMDKIFKIPPPPGKTGALAIRPLEENTPSPLAPISQPGWH

SIRIFISSTFRDMHGERDLLLRSVLPALQARVAPHRISLHAIDLRWGITEEETRRNRQLE

VCLGEVENSQLFVGILGSRYGYVPPTY-NLPDHPHFRWAQQYPSGRSVTEMEVMQFLDRG

LRLQPSAQALIYFRDSSFLGSVPDAWKPDFTPESEEAVHRISELKSYLNKQKGVTCHRYP

CEWGGVAAGLPYVGGLEEFGQLVLQDVWSMIQKLYLQPGAQLEQPVSIPDDDVVQATFQQ

LQSPPSSARPRLLQDTVQQLLLHRGRLSLVTGQSGQGKTAFLASLVSALQVPGGAKVAPL

VFFHFSGARPDQGLALTLLRRLCAYL---HRQLQEPSALPS--TYRGLVWELQQRLLPES

AQSLQSGQSLVLIIDGADRLVDQHGQLISDWIPKTLPRWVHLVLSVSSDSSLGETLEQSQ

GAHVVALGPLDPSARARLVREELALYGKRLDESPFNNQRRGCFAVGFVLDWRAGAGGLAV

GRLSGTHVESSESPQMRLLLVKRG-SALPLYLRLVTDHLRLFTIYEQVSERLRTLPATVP

LLLQHILGTLEKEHDPNILSQALATLGVTRSGLTVDQLHGVLSAWKVLPRGTKTWEEAVA

AGNSGDP-YPMGPFAYLVQSLRSLLGEGPLERPGARLCLPAGPLRTVAKGRY--GKRLEL

ESTAHILIAA---QLWKTCDPDASGTFRTCPPEALGDLPYHLLQSGNRGLLAKFLTSLHV

VAAHLELGLLPRLLEAHALYASSVP-----EEEQQLPEADTAAFHTFLKQQAPLLSQYPL

LLPQQAANQPLDSPLCRQASQLSQRWHLQRMLKWLNKPHTMGGQQSSSLSLT-VSSSPTA

VAFSPSGQRAAVGTASGTVYLLDLSTWQEEKSLVSGCDGVSSCLFLSDNSLFLTAFDGLL

ELWDLQHGCRVLQTKAHQYQITSCCLSPDRRLLATVCLGGCLKLWDTVHGQLASQHISPK

PVNCVAFHPEGQVIAIGSWAGSCSFFQVDGLKVTKELGAPGASVCTLAFNVPGRVVAVGR

LDRMVELWAWQEGALLAAFPAHHGFVAAALFLRAGCQLLTAGEDGKVQVWSGSLGRPRGC

LGSLSLSPALSVALSPDGDQVAVGYRADGIRIYKISSGSQGAQCQALDVAVTALAWLSPE

VLVSGAEDGSLQGWALQESSFESLWLMSRYQKPVLGLASSQAFLASASEDFTVRLWPRHL

LTLPQ-KAEHFPCATELRGHTGPVNCCSFSTDGCRLATGGRDR-----------------

---------------------ISCSSDGSVGLWDPELGQQLGHFLGHQSAVSAVVAVEEH

VVSVGRDGTLKVWDHQGVELTSIPAHSGPISHCAAALEPHAACQPGSELLVVTVGLDGLT

RLWHPLLVFQTHTLLGHSGPINAAAVSEASGLLLTSSEDGSIRLWQVPKEVDEANIPRSP

AAITAVAWAPDGSVAVSGNQAGELTLWQEAKAVATVQAPGRISALIWYSANTLVVVSADE

KVSEWQVELRRDSTPRNFSLRLNRVLQEDLGFLTGVGLAPDGHSLILATADLKILHLKPG

DAPSEIWHDYTEQPMMLSTHQDYGVFALQPM-APGVLSVLRQKESGEFEERLDFDLNLEN

PSGTLISVTQAKPESESSYLCASSDGMLWNLAKCSFEGEWTTGNIWQKTVQMPETQTPET

DPSVYSD----MASWPT-----TPCLKTRQRKKIHSGSVTALHVLPELLVTASKDRDVKL

WERPSMQLLGLFRCEGAVSCLEPWLGPDSTLQLAVGDTQGNVYFLSWE

>S franciscanus

MLYFSNP-----------------------------------------------------

------------------------------------------------------------

------------------------------------------------------------

------------------------------------------------------------

----------------------------QQQFINAVSASLIGGPNFHERADGTRKNINRQ

ATAIVDEDPEFILKVRIETYKELNIRTTANFLLALAAMTPDCRPFLRKYFSTSVALPSDW

IDVAELYQVLMKQEVN-YGSLPSALRKAMIDRFPRFDQYQL--GKQLPYPARIIK-ISKG

DKPAEEKPPSD-------------SDSDDEDDEDSDTDEEEMERLSFTLKQLIRKLHIKE

PVSNHEIMKSSRYPEDMETFYQSRLSGTWDTERAGKRMKLPTPETWETQVSLKGNKAAVW

EQLIDNKKLPFMAMLRNLRNMIAAGISEKHHKWVLYKLTDRGSVVYSKQFPFRFFSAYEV

LQSMEYDLDKAKH-------------RSKSKPISSPLVMLFLYLISIHYFFIKHFSELPY

DISILNRYRKALDSAVKIATSYNVKPIPGKTLLLCHVGESMERPCTSAKGLGKKRTV--S

VLLMIKGIMYGIQIALNRSFTLGNLKDGLRNG------------------IIQRVLQKRN

KKGVFRSMKCNKHR--------SKNKSISSPLVMLFLYLISIHYFFIKHFSELPYDISIL

NRYRKALDSAVKIATSYNVKPIPGKTLLLCHVGESMERPCTSAKGLGKKRTVSVLLMIKG

IKYWIQIVSQVLEVGILLGLMCKYSCENCDMLIFGGNSHRTVQLESGTILDNMASVLGQA

QVGHYSGKCQHRSYYISIASPIKEC-------------IHWYKISIIHYALIKHFSELPY

DISILNRYRKALDSAVKIATSYNVKPIPGKTLLLCHVGESMERPCTSAKGLGKKRTVSEL

LIIKGIMCKYSCENCDMLIFGGNSHRTVQLESGTILDNMASVLGQAQVGHYSGSTQLDTY

-----YILIAFSIALQSYIHYKIRIYFAKHYNIPSIHYSYKHSLPC-LLYIFSQLDNLVI

LSNTSMQEGKEAEMISDFLTKYRRVVNENLLYVSVDLSGGRCSTSATPSHPNDIFIAGYS

DQILRFIAERGDTGQVTHVQNIDKAYHLDDIKVRAAAVIVAAAVIKAAAIPARTNPRKHH

EIRVFVSSTFRDFAAEREILIKKVFPELKAKCLDRGVFFYYVDLRWGITEKQTKHGSSIE

ICLQEANRCDLFIGILGDRYGHIPNDYPDMSDNEDFNWLSSHPQSRSITELEM--HHKAL

RHAEGSNKAFFYIRSSEFVKYVFDFFLFFF---------------------------SYP

CHFGGVVEGKPVVVNLEEFGRRVINNVWNHIKQAYPEDGEILDE---VAHERAQHDMFLQ

SQLATFTGRKNQIKQCLEAVKKAESGIIMVTGKQGCGKTALLASVVDTLQ----SKSSQP

IVVHFVGAVPGSSHVSPLLKRLCNEL---IRRFGLQSQVPQ--EYRNLVSEF-PRFLEEA

AAHVDSTNKLVIVIDGISGMDTAHQAHNLDWLPQNIPQNVLFIVSAVMSSPAHAAAVRRQ

-ATMVELGPLDLMDKAEVVRRTLAAHHKTLDESPFNN-----------------------

--------------QVNIILDPEGFSHLLNFANIYTKAILI------VSTRLKAMSHTIG

TLLQEVLKRLEDDHGQDMINMALALLVSTRDGLYEEDLHHLLSLHQQMKDSKYSYKQVIG

TALSPEVMLPQAVMSRLVRGIRSLLAPTG-EHRGRQQTQFLSWFYLLFEQCL----YDNT

NNSSPFSFSGYFTHLAKT--KKSVSPWSPDYPHALSEMPYHLVQAGLFGELKNVLSDPFF

IQAKCVSGLTASLLEDFQANTSTTSKAILREREKFLTNPLIASLRSFVSRQLHILTLCPG

LLWQQAMNEP-DSSLVRHTVLESSGCREKSMVAWRNKREAADE---CSMTLTGFTDACLC

VAIAPGDQVFAVGTKNCQLKLFEMDTGKELKSYIGHSDAVTSCCFVGRTRLPSASRSILL

HL-DLSQPYRVNILKGHRGLVSGCISDPKGVNLASCSWDCRVLIWDGRSGKQTTDITEPR

PISCAQFHPDGSLLVTGSADSTLKIWDAKSKKCVAILRGHHSSVRAVTYSPTGRHIASAS

LDGAVKLWSADTGTQVGSLCGHSQPINHIAFSKNGRELVTVSNDHKTKVWSGNLGKQLAT

FKGKDESASTQATFSANGEFVAMGYHSGQVKVIEVASKREVFTCQDRESRVRGIAWL---

------------------------------------------------------------

------------------------------------------------------------

------------------------------------------------------------

------------------------------------------------------------

------------------------------------------------------------

------------------------------------------------------------

------------------------------------------------------------

------------------------------------------------------------

------------------------------------------------------------

------------------------------------------------------------

------------------------------------------------

>S_purpuratus

MSFLSNPLLAGSTSAKSKASGLT-----------------------------ATKRKASN

GNQAQGSAPSKHPTPGMSLQSSSLLLGSSLLGGNSKFG------LGLQSSNLGTKSPLMA

STGFNSKPQGSSGSGRSLLAGGSLLSSSSYLSLSSSLSSSTYGSSPAYGSSVLPNKQKTR

SLSRSNSNQVDDSKTKKVKSQHHPEQTKVKEDSIIDPTLTTCELPDYDFSRQLPVTRTIV

QEYVPSFEKLDMVNTLSTKPGEDILTELKQQFINAVSASLIGGPNFHERADDTRKNINRQ

ATSIVDKDPEFILKVALYTRKELNIRTTANFLLALAAMTPDCRPFLRKYFSTSVALPSDW

IDVAELYQTFHDKNIN-YGSLPSALRKAMIDRFPRFDQYQL--AKYNKEKKKKKKPVATT

DKPAEEKPPSD-------------SDSDDDEDEDSDTDEEEMERLSFTLKQLIRKLHIKE

PVDSVMCLLGKRYPEDMETFYASRLSGTWDTERAGKRMKLPTPETWETQVSLKGNKAAVW

EQLIDNKKLPFMAMLRNLRNMIAAGISEKHHKWVLYKLTDRGSVVYSKQFPFRFFSAYEV

LTSMEYDLKKADKLKEQLASGIKPPPRKQRGKKEDPIPKWKRAKMD------KKLSELPY

DISILNRYRKALDSAVKIATSYNVKPIPGKTLLLCHVGKSMERPCTSAKGLGKKRTV--T

KYNKEKKKKKKPVATTDKPAEEKPPSDSESDDDKDEDSDTDEEEMERLSFTLKQLIRKLH

IKEPVDSVMCLLGKRYGWMIQTGKKKDLVTPEIPRALPAPQ------QTRSELPYDISIL

NRYRKALDSAVKIATSYNVKPIPGKTLLLCHVGKSMERPCTSAKGLGKKRTKEIWRVANG

ELLVFYAMDNVLEVGVLLGLMCKYSCENCDMLIFGGNSHRTVELESGTILDNMASVLGQA

QVGHYTGVHDNK--LKEQLASGIKPPPRKQRGKKEDPIPKWKRAK-----MDKKLSELPY

DISILNRYRKALDSAVKIATSYNVKPIPGKTLLLCHVGKSMERPCTSAKGLGKKRTVLEV

GVLLGLMCKYSCENCDMLIFGGNSHRTVELESGTILDNMASVLGQAQVMMTQEEQGQGLD

PTILGEILRDRDEVSQQSNAVPGLSFLQDIMPQEKEGQGLDPTILGEMLRDRVELDNLVI

LSNSAMQEGKEADMIKGFLTKYRRVVNENLLYVSVDLSGGRCSLSATPSHPNDIYIAGYS

DQILRFIAERGDTGQVTHVQNIDKAYNLDDIKIQTGKKKDLVTPEIPRALPAPQQTRRHH

TVRVFISSTFRDMHGERDLLTRFVFPELRAKVRTRFVNLYEVDLRWGVSEEQTKHSSSVE

VCLQEANRCDLFIGILGDRYGYVPNDYPDLSDNEDFNWLSTHPKSRSITELEM--YHKAL

RHSEGSTKAFFFIRSSEFVKHVPNKLRSQFEDESSQSKEKLASLKETIRTSGCEVFDGYP

CHFGGVVEGKPVVVNLEEFGRCVINNVWNHIKQAYPEDGEILDE---VAHERAQHDMFLQ

SQLATFTGRKTQVKQCLEAVKKAETGIIMVTGKQGCGKTALMASVVDTLQ----SKSSQP

IIVHFVGAVPGSSHVSPLLKRLCNEL---VRRFGLQSQVPQ--EYKNLVSEF-PRFLEEA

AGHVDSTNKLVIVIDGISGMDAAHQAHNLDWLPQNIPQNVLFIVSAVMSSPAHAAVVRRQ

-ATMVELGPLDLMDKAEVVRRTLAAHHKKLDESPFNN-----------------------

--------------QMKLLVSKRE-ANLPLFLKLACEELRVFGVFEEVSTRLKAMSHTIG

TLLQEVLKRLEDDHGLDMINMALALLVSTRDGLYEEDLHYLLSLHQQMKDSKYTYKQVIG

TALGPEVMLPQAVMSRLVRGIRSLLAPTG-EHSDHRLRVSHGQISQAIIQRYMKGAASEL

QIRTHALLAGYFTHLSRT--KNAVSPWSPHYPHALSEMPYHLVQAGLFGELKNVLSDPFY

VQAKCVSGLTASLLEDFQANTSTTSKAILREREKFLTNPLIASLRSFVSRHLHILTLCPG

LLWQQAMNEA-DSSLVRHTVLESAGCREKGIVAWRNKRETADE---CSMTLTGFTDAVLC

VAIAPGDQVFAVGTKNCQLKLFEMDTGKELKSYIGHSDAVTSCCFVGRTRLVSASRDKTL

SLWDVDQGFRVNILKGHRGLVSGCISDPKGVNLASCGWDCRVLIWDGRSGKQTTDITEPR

PISCLSYHPEGKLLVTGSWDSTLKIWDTFNKKRVAILRGHHSSVRAVTYSPTGRHIASAS

LDGAVKLWSADTGTQVGSLCGHSQPINHIAFSKNGRELVTVSNDHKTKIWSGNLGKQLAT

FKGKDESASTQAAFSADGEFVAMGYHSGEVKVIEVASKREVVTCPDRESRVKGIAWY---

------------------------------------------------------------

------------------------------------------------------------

------------------------------------------------------------

------------------------------------------------------------

------------------------------------------------------------

------------------------------------------------------------

------------------------------------------------------------

------------------------------------------------------------

------------------------------------------------------------

------------------------------------------------------------

------------------------------------------------

>L variegatus

------------------------------------------------------------

-----MFMFVVLESYEIAMKC---------------------------------------

-----SLLQFLI------------------------------------------------

------------------------------------------------------------

---------------------------QQQQFINAVSASLIGGPNFHERGDSTRKNINRQ

ATSIINEDPEFIYQVALYTRKELNIRTTANFLLALAAMTPECRPFLRKYFSSSIALPSDW

IDVAELYQTMHDKNIN-YGSLPSALRKAMIDRFPRFDQYQLGAAKY-LKLKQAIKHAQKC

KKPAECQDLPD--------------RRDIGDSENADYLSEEMERLSFTLKQLIRKLHIND

PVDSVMCLMGYRYPEDMESFYQSRLPGTWDPERAGKRMKLPTPETWETQVSLKGNKAPVW

EQLIDKKKAPFMAMLRNLRNMIAAGISEKHHKWVLYKLTDRGSVVNSKQFPFRFFSAYEV

LQSMERELEKARY------YSIRPIISKQAQLSISPSIGQSKSQEYISSL--LPSSELPY

DITILNRYRKALDTAVKIATSYNVKPIPGKTLLLCNVGSYMDTPCSSAKGLGKKYPLFAA

KYQAIKYHIYQILKLLSIARECLALS---RIDKMSHAIPNLISYYFRLSFTLKQLIRKLH

INDPVDSVMCLLGK---------RCYAYYNWYLLLFSPQPMISHPYLPS-SELPYDITIL

NRYRKALDTAVKIATSYNVKPIPGKTLLLCNVGSYMDTPCSSAKGLGKKRAIPLSQYASI

DSYSYY-IPPIIYVGILLGLMCKYSCEHCDMRIFASSAHSAVELESGTILDNMTSVLSQA

RVSHHRVILNSTTYFPLSFIICMYCFCHNHCGA----LLEYIFLILVCYGI----SELPY

DITILNRYRKALDTAVKIATSYNVKPIPGKTLLLCNVGSYMDTPCSSAKGLGKKRCDCVV

GILLGLMCKYSCEHCDMRIFASSAHSAVELESGTILDNMTSVLSQARVSHTVILNGSLGF

PK---KLLIALNLLLKNSSGFCGSFFFFISCDIFASSF--KFLSLCLLFIILVQLDNLVI

LSTSSAHGGKETEMISEFLSKYRRVVNENMLYVNVDLSGGRCRTSDTPSHPNDIFIAGYS

DQILRFIAERGDTGQVTHVQNIDKAYNLTDCKVFFINKLLEPNVQFAEVKFVNKKTKEKY

VIRVFVSSTFRDFEEERELIIKKVFPELNRLCLDRGYFFTYVDLRWGITEEQTKSGRTIE

ICLREVSRSDMIIGIFGQVYWYLPNDYPDMSDIEDFDWLSAHPKSRSITELEM--YHKAL

RNWGTSNNAFFFIRNSEIIRNVPNKLRSQFIDENTQRKERLASLKDTIRTSGCEVFDGYP

CHFGGVVGGKPVVVNLEEFGRRVINNIWNYIKHAYPEDGEILDE---VVHERAQHDMFLQ

SQLATFTGRKNQFKQCLDIIKKAETGLLMVTGKQGCGKTALLVR----YDKGDGRFEAGS

LSLGRAIIRP------------QNTLADKRSRIKKEFFLKAIDDARNLVSEF-PRFLEEA

AKHVDSANKLVIVIDGVSGMNTAHQAHNLDWLPQNIPQNVVFILSGVASSPAHAAAVRRQ

-ATVIELGPLDLMDKAEMVRRTLAAHHKTLDESPFNN-----------------------

--------------QMKLLVSKRE-ANLPLFLKLACEELRVIGVIE-VSTRLKAMSHTIG

TLLQEVLKRLEDDHGQDMIIVALALLVSTRDGLTPAS-SCLLSLDRQLNQSKYTYNQVIR

TGLGPEVMLPQAEMSRLVRGIKSLLAPTG-EHSDHRLRVSHGQISQAIVQRYMKGAASEL

QIRTHALLAGYFNHLSRT--KNVASPWCTDYPHALTEMPYHLVQAGLFAELKNVLSDPFF

IQAKCCSGLTASLLEDKQANTSTTSKAILREREKFLTNPLITGLRSFISRHLHILTLCPG

LLWQQAMNEP-NKSLVREAMLESAGSGEKGMVAWRNKRETADE---CAMTLTGFTDVCLC

VAIAPGAQVFAVGTKNCQLKLYEMETGQELKSFIGHSDAVTSCCFVGKSRLVSASRDKTL

SLWDVEQGFRVNTLKGHRGLVSGCISDSKGVNLASCGWDCRVLIWDGRSGKQTSDITEPR

PISCLSYHPDGKLLVTGSWDATLKIWDTFNKKRVAILRGHCSSIRAVTYSPTGRHIASAS

LDGAVKLWSADTGTQVSSLCGHSQPINHIAFSQNGRELVTVSNDHKTKVWSGNLGKQLAT

FKGKDQSASTQATFSPYGEYVAMGYHSGEVKVIEVASKREVFASVLRESRVKGIAWG---

------------------------------------------------------------

------------------------------------------------------------

------------------------------------------------------------

------------------------------------------------------------

------------------------------------------------------------

------------------------------------------------------------

------------------------------------------------------------

------------------------------------------------------------

------------------------------------------------------------

------------------------------------------------------------

------------------------------------------------

>M musculus

MEKLCGHVPGHSDILSLKNRCLTMLPDLQPLEKIHGHRSVHSDILSLENQCLTMLSDLQP

TERIDGHISVHPDILSLENRCLTMLPDLQPLEKLCGYMSSNPDVLSLENRCLATLPTVKS

TALTSPLLQGLH---ISHTAQADLHSLKTSNCLLPELP-TKKTPCFSEELDLPPGPRALK

SMSATAQVQEVALGQWCVSKEKEFQEEESTEVPMPLYSLSL-EEEEV-EAPVLKLTSGDS

GFH--------------PETTDQVLQEKKMALLTLLCSALASNVNVKDASDLTRASILEV

CSALASLEPEFILKASLYARQQLNLRDIANTVLAVAALLPACRPHVRRYYSAIVHLPSDW

IQVAEFYQSLAEGDEKKLVSLPACLRAAMTDKFAEFDEYQL--AKYNPRKHRSKRRSRQP

PRPQKTERPFSERGKCFPKSLWPLKNEQITFEAAYNAMPEKNRLPRFTLKKLVEHLHIHK

PAQHVQALLGYRYPATLELFSRSHLPGPWESSRAGQRMKLRRPETWERELSLRGNKASVW

EELIDNGKLPFMAMLRNLCNLLRTGISARHHELILQRLQHEKSVVHSRQFPFRFLNAHDS

IDKLEAQLRSKA----------------------------------------SPF---PS

NTTLMKR--------IMIRNSKKNRR-PASRRHLCTLTRRQLRAAMTIP----------V

MYEQLKREKLRLHKA---------------------------------------------

--------------------------------------------------RQWNCDVELL

ERYRQALETAVNLSVKHNLSPMPGRTLLVYLTDANADRLCPKSHSQG-------------

--------PPLNYVLLLIGMMVA-RAEQVTVCLCGGGFVKTPVLTADEGILKTAIKL-QA

QVQELEGNDE--------------------------------------------------

------------------------------------------------------------

--------------WPLDTFG---------------------------------------

----------------------------------------KYLLS--LAVQRTPIDRVIL

FGQRM-----DTELLKVAKQIIWQHVNSKCLFVGVLLQ--KTQYISPNLNPNDVTLSGCT

DGILKFIAEHGASRLLEHVGQLDKLFKIPPPPGKTQAPSLRPLEENIPGPLGPISQHGWC

NIRLFISSTFRDMHGERDLLMRSVLPALQARVFPHRISLHAIDLRWGITEEETRRNRQLE

VCLGEVENSQLFVGILGSRYGYIPPSY-DLPDHPHFHWTHEYPSGRSVTEMEVMQFLNRG

QRSQPSAQALIYFRDPDFLSSVPDAWKPDFISESEEAAHRVSELKRYLHEQKEVTCRSYS

CEWGGVAAGRPYTGGLEEFGQLVLQDVWSMIQKQHLQPGAQLEQPTSISEDDLIQTSFQQ

LKTPTSPARPRLLQDTVQQLLLPHGRLSLVTGQAGQGKTAFLASLVSALKVPDQPNEPPF

VFFHFAAARPDQCLALNLLRRLCTHL---RQKLGELSALPS--TYRGLVWELQQKLLLKF

AQSLQPAQTLVLIIDGADKLVDRNGQLISDWIPKSLPRRVHLVLSVSSDSGLGETLQQSQ

GAYVVALGSLVPSSRAQLVREELALYGKRLEESPFNN-----------------------

--------------QMRLLLAKQG-SSLPLYLHLVTDYLRLFTLYEQVSERLRTLPATLP

LLLQHILSTLEQEHGHDVLPQALTALEVTRSGLTVDQLHAILSTWLILPKETKSWEEVLA

ASHSGNP-FPLCPFAYLVQSLRSLLGEGPVERPGARLCLSDGPLRTTIKRRY--GKRLGL

EKTAHVLIAA---HLWKTCDPDASGTFRSCPPEALKDLPYHLLQSGNHGLLAEFLTNLHV

VAAYLEVGLVPDLLEAHVLYASSKP-----EANQKLPAADVAVFHTFLRQQASLLTQYPL

LLLQQAASQPEESPVCCQAPLLTQRWHDQFTLKWINKPQTLKGQ--QSLSLT-MSSSPTA

VAFSPNGQRAAVGTASGTIYLLNLKTWQEEKALVSGCDGISSFAFLSDTALFLTTFDGHL

ELWDLQHGCWVFQTKAHQYQITGCCLSPDCRLLATVCLGGYLKLWDTVRGQLAFQYTHSK

SLNCVAFHPEGQVVATGSWAGSITFFQADGLKVTKELGAPGPSVCSLAFNKPGKIVAVGR

IDGTVELWAWQEGARLAAFPAQCGCVSAVLFLHAGDRFLTAGEDGKAQLWSGFLGRPRGC

LGSLPLSPALSVALNPDGDQVAVGYREDGINIYKISSGSQGPQHQELNVAVSALVWLSPS

VLVSGAEDGSLHGWMFKGDSLHSLWLLSRYQKPVLGLAASRELMAAASEDFTVRLWPRQL

LTQPHVHAVELPCCAELRGHEGPVCCCSFSPDGGILATAGRDRNLLCWDMKIAQAPLLIH

TFSSCHRDWITGCAWTKDNILVSCSSDGSVGLWNPEAGQQLGQFSGHQSAVSAVVAVEEH

IVSVSRDGTLKVWDHQGVELTSIPAHSGPISQCAAALEPRPGGQPGSELLVVTVGLDGAT

KLWHPLLVCQIRTLQGHSGPVTAAAASEASGLLLT-SDDSSVQLWQIPKEADDSYKPRSS

VAITAVAWAPDGSMVVSGNEAGELTLWQQAKAVATAQAPGRVSHLIWYSANSFFVLSANE

NVSEWQVGLRKGSTSTSSSLHLKRVLQEDWGVLTGLGLAPDGQSLILMKEDVELLEMKPG

SIPSSICRRYGVHSSILCTSKEYGLFYLQQG-DSGLLSILEQKESGEFEEILDFNLNLNN

PNGSPVSITQAKPESESSLLCATSDGMLWNLSECTPEGEWIVDNIWQKKAKKPKTQTLGT

ELSPHSELDFSMDCWID-----PTHLKAQQCKKIHLGSVTALHVLPGLLVTASKDRDVKL

WERPSMQLLGLFRCEGPVSCLEPWMEPSSPLQLAVGDTQGNLYFLSWE

TERT

>H sapiens

MPRAPRCRAVRSLLRSHYREVLPLATFVRRLGPQGWRLVQRGDPAAFRALVAQCLVCVPW

DARPP-PAAPSFRQVSCLKELVARVLQ---RLCE--RGAKNVLAFGFALLDGARGGPPEA

FTTSVRSYLPNTVTDALRGSGAWGLLLRRVGDDVLVHLLARCALFVLVAPSCA-----YQ

VCGPPLYQLGAATQA--------RPP-PHASGPRRRLGCERAWNHSVREAGVPLGLPAPG

ARRRGGSASRSLPLPKRPRRGAAPEPERTPVGQGSWAHPGRTRGPSDRGFCVVSPARPAE

EATS-LEGALSGTRHSHPSVGRQH------------HAGPPSTSR--PPRPWD----TPC

----PPVYAETKHFLYS--SGDKEQLR-----PSFLLSSLRPSLTGARRLVETIFLGSRP

WMPGTP--RRLPRLPQRYWQMRPLFLELLGNHAQCPYGVLLKTHCPLRAAVTPAAGVCAR

E-----------------------------------------------------------

------------------------------------------------------------

------------------------------------------------------------

------------------------------------------------------------

--------------------------------------KPQGSVAAPEEEDTDPRRLVQL

LRQHSSPWQVYGFVRACLRRLVPPGLWGSRHNERRFLRNTKKFISLGKHAKLSLQELTWK

MSVRDCAWLRRSPGVGCVPAAEHRLREE-ILAKFLHWLMSVYVVELLRSFFYVTETTFQK

NRLFF--YRKSVWSKLQSIGIRQHLKRVQLRELSEAEVRQHREARPALLTSRLRFIPKPD

GLRPIVNMDYVVGA-RTFRREKRAERLTSRVKALFSVLNYERARRPGLLGASVLGLDDIH

RAWRTFVLRVRAQD------PPPELYFVKVDVTGAYDTIPQDRLTEVIASIIK---PQNT

YCVRRYAVVQKAAHGHV--RKAFKSHVSTLTDLQPYMRQFVAHLQ--ETSPLRDAVVIEQ

SSSLNEASSGLFDVFLRFMCHHAVRIRGKSYVQCQGIPQGSILSTLLCSL----------

---CYGDMENKLFAGIRRDGL-------LLRLVDDFLLVTPHLTHAKTFLS---------

------------------------------------------------------YARTSI

RASLTFNRGFKAGRN-MRRKLFGVLRLKCHSLFLD-LQVNSLQTVCTNIYKILLLQAYRF

HACVLQLPFHQQVWKNPTFFLRVISDTASLCYSILKAKNAGMSLGAKGAAGPLPSEAVQW

LCHQAFLLKLTRHRVTYVPLLGSLRTAQTQLSRKLPGTTLTALEAAANPALPSDFKTILD

>H_glaber

MPRAPRCRAVRALLRRRYYEVLPLAAFARRLGPEGARLLRRGDPAAFRALVAQCMVCVPW

DARPMYPGTPSFRQVSSLKELVARVVQ---RLCE--RGARNVLTFGFGLLNEARGGPPLA

FTTNVCHFRPNTVTDSLRGSGAWGLLLSRLGDDVLVHLLSRCALYQLVAPSCA-----YQ

VCGQPLHELGGLTASQR------RAPHP--------------------------------

---RPVLGSLRRAPSCSAGASPTP-PKKPRHGLGNG-----------------EPRVVSP

GAAS-LEGEPSRSRDSRSTTG---------------------------------------

----PREATETKRFLYSRVGGGAERLR-----ASFVLSALPPNMTGARRLVEALFLGPPP

VRPGPPYRSRARRLAPRYWRMRPLFRELLANHARCPYGALLRVHCPLRVSVDP-------

------------------------------------------------------------

------------------------------------------------------------

------------------------------------------------------------

------------------------------------------------------------

------------------------------------------------EGDAGAQRLAQL

LRAHCSSWQVYAFLRACLHLLVPAGLWGSRRNERRFLRCTKRFLSLGKHGKLSLRELTWK

VKVRDCAWLRASPGDACVPAAEHRLQER-LLATFLFWLLDTYVVELLRSFFYVTETTFQK

NRLFF--YRRSLWRELQSIGVRQHFERVQLRELSAAELRQHQEARLALPTSRLRFVPRLS

GLRPIVNMDCMVGTGATLGERKQAQQCTPRIRTLFSVLNYERARWPKLLGASVLGLDDTY

RAWRAFVLRMRAQD------PAPRLYFVKADVSGAYDALPQDKVAEVVASVLR--PWENT

YCVRRYAVVQRAARGHT--RRAFRRHVSTLPDLQPYMSRFVEHLQ--EACRLRDAVVIEQ

SCSLNETGCSLFDFFLRFLRGSILRIGGRCYVQGRGIPQGSILSTLLCSL----------

---CYGDMENMLFTRVQQDGV-------LLRLVDDFLLVTPHLAQARAFLGTLVRGIPEY

GCTLNLQKTLVNFPVEDGVLGGPAPLQLPTHCLFPWCGLLLDTRTLEVRCNYASYARTSI

RASLTFDRGIRAGWS-MRRKLLAILRLKCHGLFLD-LQVNSLRTVLINIYKIFLLQAYRF

HACVLQLPFHQPVRKNAPFFLRIISDSASRCYSILKAKNAGVSLGTKGATGPFPSEAARW

LCHQAFLLKLGHHRATYKCLLGPLRTAQTQLRGKLPKVTMAALEAAADPALTMDFKTILD

>S franciscanus

----------MDILERFYPRVSRLEDFLKDLN-GCPPLYIPKDKAGYRAFLQSTLVGIP-

SCHMNYQGPLDYTQHSDHMEVINRVLLLSDQMCQ--HRVRS------------GGRVTQL

FVKQKRKIMKRRLGMP---VVDLFLVFHKVSCELDQTICREGFISQASSPSTYEPRQMSR

SIGTPLSMLISSLPI--------NTPSPAKTSTIEQGSKKRRKSFQLKDADKGGKVDECD

GKGRNVQGKRKRDEDVGCNQEDAN-VAKFRRGGFMV-----------------EPYLVCS

YQFKFLYFHLFKKKNYFLSLGTAPAHL-------SPSDQGSSITQDFPQSAWVSILMCRV

----LKRSNLLIYMLYNHIRYHASKIKYYCRYPVLIK---EKNIIFFRKL-KLNSILNHQ

CPLNLP--QDSPPSDHTDTSHQECSMEALGKRSHTKKSSLSPPCSSECASREKSLAELRE

E-----------------------------------------------------------

------------------------------------------------------------

------------------------------------------------------------

------------------------------------------------------------

-------------------------------------------------------SMLQG

ISFH----------------------FSGRNFKFEYSKSSVSIVDNTEDITSSTFYYTSV

LQAEDCAWCQLEKFKGRDPPFTSLVKQRQLVTVLFFFGGDGGRC--LCAPLHLNKSKMGS

SILSFILF--------------------------QNDVRKLVSAGLTLGFSRLRFVPKTK

GLRPITRMG------KSVIEEKKVSQ----------------------------------

----------SPND------PVKPAF-------------KNHLYRKTTYSLLSYQDKPEE

YQIQRYVTVTRAASASTGLQKTSRRHVTT--ELSSFHRQLIKMAQ---HGEIKNAIIINQ

VYDYNLITFVLLHNYFEHIICHYFQSGRKHYWRQDGISQGSILSSLLCSF----------

---FYAHLERCYLSDIDQEGVCNHSLVRLLSFLCSFFHASLRKKKPQ--------GVKQY

GCSANPSKTLANFDFMYD-----GQLVPRSAEWFPWCGIVFNTQTLNISNDYTKYNN---

-RLLVRGRGLNPRPPGCEGGRSTDANTPVLWLYCTIFQINSFLTIVINVYHLLLLLAHRF

HSYYHCLASYIKQNTPPLQFYSKTEDYAIYLYYIAFYICIYPSHHIHHAMYFIEIFIFR-

IGLKAFDTKLSKHKGLYHPLIKLVRKHRRKAALKMVIILISQLRCQ--------------

>S purpuratus

----------MDILERFFPRVSCLEDFLRDLN-GCPPLCIPRDKAGYRAFLQSTLVGIP-

SCHISYQGPLDYTQHSDHMEVINRVLL---RLQHQGKGGKNVLLNGFTSWRSEGDYAGIP

PSINV----PNNNSNQLR-IPFWSKLLSRIGDELIMHILENLSIFAAAPPSCY-----IQ

LTGTPLSMMISSLPT--------NTPSPAKTSTVEQRLKQRRRSFQSKDAAKGGKVDRCD

GDRRNAQGKRKRDRDGGCNQEDAN-VAKFRKGEQGMRQPTQTKEAENRKEIKKDPKVLES

MITGLERSYYILNACYSRTVGRTLLKHDLVSKLPASNSGAQMLTQHIFNSPGKSDKTASH

----DGKGRSEIGIQGDDKGRKTKKLQRTCRRLIKVQALLKTFLTQHRKL-KLNSILNRQ

CPLNPP--QDGPPSDHTNTSHQEGSTETQGQRSHTKKTSLPPPCAGEGASCEKSLAELRE

ESMLEGYSPKVFQRKRPKQSVLNNTQVSSVEQFDGTRMEESVIQVDDRRGGIEQKRKDDG

RKGSTKKVGEGIRDGLRDKVGLGETEERVGNVRRKKIGEMVKSTKAQSTTDERKIDEKNG

HSKSHPGSTKDFDVSQSLSARMEMDENATSSTSGDALQETDSIPSQPHMRLRRKTNTETE

TRAGLPSGEVTVDNDDDDDTLTVSGKILSQPDKRLRGKTNMRAKRNTLSIGDGVLRESQP

DRKTQRRTKTKTGRQLPSDTAAPSGHPKRDETPDSMQFQLKRKMKKTTDTKALPNDAATL

IKMKTDPWQVCLFLRQVLLKVVPDELWGSVHNRNVFLKGVKKFVQLGRFERLSLRELTEE

IRAEDCNWCQLEKFRGRDPPFTSLVKQRQLVTDFFHWLMIGYTVPLIMMCFYVTETSSSR

NQLVF--YRKPVWPLLEKIGIQYYVAHGIMKPMKENDVKKLVSAGLTLGFSRLRFVPKTK

GLRPITRMG------KSVIEEKKGLSVRLLLQDLFDVLTYHKINQPSVMGSSLLGIDGIY

HKVLKFMKDRKERK------DTRPLYFVKIDIEKCYDSIKRSKLLQIISTLLQGHDKPDE

YRLQRYVTVTRAASASTGLQKRSHRHVTT--ELSSFHRQLIQMAQ---HGKIKNAIIINQ

VHTVKVTPKELLQRLKQHVMADVVKSGRKYYWRQDGISQGSILSSLLCSF----------

---FYAHLERCYLSDIDQEGL-------MMRLIDDFLLITPHHDKAQRFLQLLLSGVKQY

GCSANPNKTLANFDFMHD-----GQLVPRSKELFPWCGIVFKTQTLNISNDYTKYNNVSI

RYTLTICTGRDTSLHLMRIKLTWSLKAKNVSIFMD-PLINSFLTIVINVYHLLLLLAHRF

HSYYYCLASYIKQNTPPLQFYNIWASCVRIFHACTVSKLRKDDQGERETTFPLTINTVNW

IGLKAFDTKLGKHKGLYHPLIKLVRKHRQKAALKMGKAMLEVLEEMTNPALPDDFMKMRR

>L variegatus

----------MDILERIYPKVSCLEDFLRGLD-GCPPLYLPKDKAG--------------

------------------------------------------------------------

-------------------VSFLFLSMFRIGDELMMHILENLSIFAAAPPSCY-----IQ

LTGEKIFPIIQICK---------GYLDYYYITFVRHLILSKLTVFCLFVLGIKIK-----

-ERKNVAGGNDTDNAAILTIKNQNWCASPIDRPTTVNHPVLSRVLNLMDLALMDKRVKRG

LSFG-SQNLLWRQNDKHIRWNNHNIDHG-----NASSSDFTFKSINIVPKCDVTMSQQTC

WFTVAAAGLSIHIILYN--------LQKWCV-LSLVVTIILRVIKGCRRI-QIYFSEQRR

S-------SLIAHQAHLYSRIKHLAIHIL--HIKSLKRAIYPT-----------------

------------------------------------------------------------

------------------------------------------------------------

------------------------------------------------------------

------------------------------------------------------------

------------------------------------------------AKQHQPHYICNP

AAIK--PWQLTSSISYFILSFF-------------FLYEIVNQESQHQCYTLPL------

IQAEDCEWAQLEKFKGHDPPFTSVVKQRQLVMDFFHWLMVGYTIPLIMVIFYVLETASSR

NQLIF--YRTPVWILLEKIGIQQIPFYSYILMLEKGEVMKRISTGQTLGISRLRFIPKTK

SLRPITRMG------KSVISEKKGSLEEKEIDAKICL---------QVIAQLACSLFDCI

SNDFCFEYDAEKPDLLIDQHNSRPLYFVKVDIEKCYDSIKHRKLLQIISSLLQ-MDKPDE

YFIQRYVTVTKAASTSSGLQKTTHRHVTT--EMSIFHRQLIEMAK---RGQMKNAIIINQ

VLAPKAA--------FEPICSTKFCINAR-------------LSTIKCEFRQRLFCCSIQ

ISSAFGLQINILCTEISLDIF----LILMMRLIDDFLLVTPHLDKAQRFLHLLISGVEQY

GCSANPTKTLVNFDFMFN-----GQMVPRSTELFPWCGIVFNTQTLNISNDYTKYDNIS-

KYIYLIIDGFCEKAN-IDKFRHFTVSKSCA---CADFEINSFRTIVVNVYHLLLLLGYRF

HSYYHCLANCIQKNTRPLQFSSMWASCVRIFHAYIISKLRRDNREDAETAFPLTINTVNW

IGLKAFETKLSQHRGSYHPIIKLVTTHRRKAAQKMFLCQVR-INIYSQKRYSLAAPNIKY

>M_musculus

MTRAPRCPAVRSLLRSRYREVWPLATFVRRLGPEGRRLVQPGDPKIYRTLVAQCLVCMHW

GSQPP-PADLSFHQVSSLKELVARVVQ---RLCE--RNERNVLAFGFELLNEARGGPPMA

FTSSVRSYLPNTVIETLRVSGAWMLLLSRVGDDLLVYLLAHCALYLLVPPSCA-----YQ

VCGSPLYQICATTDIWPSVSASYRPTRPVGRNFTNLRFLQQIKSSSRQEAPKPLALPSRG

TKRHLSLTSTSVPSAKKARCYPVPRVEEGPHRQVLPTPSGKSWVP--------SPARSPE

VPTA-EKDLSSKGKVSDLSLSGSV----------CCKHKPSSTSLLSPPRQN-----AFQ

----LRPFIETRHFLYSR-GDGQERLN-----PSFLLSNLQPNLTGARRLVEIIFLGSRP

RTSGPL--CRTHRLSRRYWQMRPLFQQLLVNHAECQYVRLLRSHCRFRTANQQ-------

------------------------------------------------------------

------------------------------------------------------------

------------------------------------------------------------

------------------------------------------------------------

---------------------------------------------VTDALNTSPPHLMDL

LRLHSSPWQVYGFLRACLCKVVSASLWGTRHNERRFFKNLKKFISLGKYGKLSLQELMWK

MKVEDCHWLRSSPGKDRVPAAEHRLRER-ILATFLFWLMDTYVVQLLRSFFYITESTFQK

NRLFF--YRKSVWSKLQSIGVRQHLERVRLRELSQEEVRHHQDTWLAMPICRLRFIPKPN

GLRPIVNMSYSMGT-RALGRRKQAQHFTQRLKTLFSMLNYERTKHPHLMGSSVLGMNDIY

RTWRAFVLRVRALD------QTPRMYFVKADVTGAYDAIPQGKLVEVVANMIR--HSEST

YCIRQYAVVRRDSQGQV--HKSFRRQVTTLSDLQPYMGQFLKHLQDSDASALRNSVVIEQ

SISMNESSSSLFDFFLHFLRHSVVKIGDRCYTQCQGIPQGSSLSTLLCSL----------

---CFGDMENKLFAEVQRDGL-------LLRFVDDFLLVTPHLDQAKTFLSTLVHGVPEY

GCMINLQKTVVNFPVEPGTLGGAAPYQLPAHCLFPWCGLLLDTQTLEVFCDYSGYAQTSI

KTSLTFQSVFKAGKT-MRNKLLSVLRLKCHGLFLD-LQVNSLQTVCINIYKIFLLQAYRF

HACVIQLPFDQRVRKNLTFFLGIISSQASCCYAILKVKNPGMTL---KASGSFPPEAAHW

LCYQAFLLKLAAHSVIYKCLLGPLRTAQKLLCRKLPEATMTILKAAADPALSTDFQTILD

>SIG

------------------------------------------------------------

------------------------------------------------------------

------------------------------------------------------------

------------------------------------------------------------

-------------------------------+----------------------------

------------------------------------------------------------

--------------------------+---------------------------------

------------------------------------------------------------

------------------------------------------------------------

------------------------------------------------------------

------------------------------------------------------------

------------------------------------------------------------

------------------------------------------------------------

---------------------------------------+--------------------

------------------------------------------------------------

------------------------------------------------------------

------------------------------------------------------------

--------------------+---------------------------------------

------------------------------------------------------------

------------------------------------------------------------

------------------------------------------------------------

------------------------------------------------------------

---------+--------------------------------------------------

-------------------------+----------------------------------

VLDLR

>H sapiens

----------MGTSALWALWLLLALCWAPRESGAT---------GTGRKAKCEPSQFQCT

NGRCITLLWKCDGDEDCVDGSDEKNCVKKTCAESDFVCNN-G------QCVPSRWKCDG-

-DPDCEDGS-----DESPEQCHMRTCRIHEISCGAHSTQCIPVSWRCDGEN-DCDSGEDE

ENCGNITCSPDEFTCSSGRCISRNFVCNGQDDCSDGSDELDCAPPTCGAHEFQCS---TS

SCIPISWVCDDDADCSDQSDESLEQCGRQPVIHTKCPASEIQCGSGECIHKKWRCDGDPD

CKDGSDEVNCPSRT---CRPDQFECEDGSCIHGSRQCNGIRDCVDGSDEVNCKNVNQCLG

PGKFKCRSGECIDISKVCNQEQDCRDWSDEPLKE-CHINECLVNNGGCSHICKDLVIGYE

CDCAAGFELID----RKTCGDIDECQN-PGICSQICINLKGGYKCECSRGYQMDLAT--G

VCKAVGK--EPSLIFTNRRDIRKIGLERKEYIQLVEQLRNTVALD---ADIAAQKLFWAD

LSQKAIFSASIDDKVGRHVKMIDN-VYNPAAIAVDWVYKTIYWTDAASKTISVATLDGTK

RKFLFNSDLREPASIAVDPLS-GFVYWSDWGEPAKIEKAGMNGFDRRPLVTADIQWPNGI

TLDLIKSRLYWLDSKLHML-SSVDLNGQDRRIVLKSLEFLAHPLALTIFEDRVYWIDGEN

EAVYGANKFTGSELATLVNNLNDAQDIIVYHELVQPSGKNWCEEDMENGGCEYLCLPAPQ

INDHSPKYTCSCPSGYNVEENGRDCQSTATTVTYSETKDTNTTEISATSGLVPGGINVTT

AVSEVSVPPKGTSAAWAILPLLLLVMAAVGGYLMWRNWQHKNMKSMNFDNPVYLKTTEED

LSIDIGRHSASVGHTYPAISVVSTDDDLA

>H_glaber

----------MGTSARWALWLLLALSWEPRESSAT---------GTGRKAKCDPSQFQCT

NGRCITLLWKCDGDEDCVDGSDEKNCVKKTCAESDFVCNN-G------QCVPNRWQCDG-

-DPDCEDGS-----DESPEQCHMRTCRINEISCGARSTQCIPVSWRCDGES-DCDSGEDE

ENCGNITCSPDEFTCSSGRCISRNFVCNGQDDCNDGSDELDCAPPTCSAHEFQCS---TS

SCIPLSWVCDDDADCSDQSDESLEQCGRQPVMHTKCPASEIQCGSGECIHKKWRCDGDPD

CKDGSDEVNCPSRT---CRPDQFECEDGSCIHGSRQCNGIRDCVDGSDEVNCKNANQCLG

PGKFKCRSGECIDMIKVCNQEQDCRDWSDEPLKE-CHVNECLINNGGCSHICKDLVIGYE

CDCAAGFELID----RKTCGDIDECQN-PGICSQICINLKGGYKCECSRGYQMDLAT--G

VCKAVGK--EPSLIFTNRRDIRKIGLERKEYIQLVEQLRNTVALD---ADIAAQKLFWAD

LSQKAIFSASIDDKVGRHVKMIDN-VYNPAAIAVDWVYKTIYWTDAASKTISVATLDGTK

RKFLFNSDLREPASIAVDPLS-GFVYWSDWGEPAKIEKAGMNGFDRRPLVTVDIQWPNGI

TLDLIKSRLYWLDSKLHML-SSVDLNGQDRRIVLKSLEFLAHPLALTIFEDRVYWIDGEN

EAVYGANKFTGSELATLVNNLNDAQDIIVYHELVQPSGKNWCDEDMENGGCEYLCLPAPQ

INEHSPKYTCSCPSGYSLEENGRQCQSTATTVAYRESKDTNTTEILPTSGLVLGGINVTT

AIPEVSVPPQGTSAAWAILPLLLLAMAAVGGYLMWRNWQNKNMKSMNFDNPVYLKTTEED

LSIDIGRHSASVGHTYPAISVVSTDDDLA

>M_brandtii

MWASEPLNNGAASPARR---SVREEAEGPETCAAAPARSVDFRDGVGRKAKCDPSQFQCT

NGRCITLLWKCDGDEDCADGSDEKNCEKKTCAESDFVCNN-G------QCIPSRWQCDG-

-DPDCEDGS-----DDSPEQCHMRTCRTNEISCGAHSTQCIPESWRCDGEN-DCDSGEDE

ENCGNITCSSDDFTCSSGRCISRNFVCNGQDDCNDGSDELNCAPPSCGAHEFQCS---AS

SCIPLSWVCDDDADCSDQSDESLEQCGRQPVMHTKCPASEIQCGSGECIHKKWRCDGDPD

CRDGSDEVNCPSRT---CRPDQFECEDGSCIHGSRQCNGIRDCVDGSDEVNCKNVNQCLG

PGKFKCRSGECIDIIKVCNQEQDCRDWSDEPLKE-CHVNECLVNNGGCSHICRDLVIGYE

CDCAAGFELID----RKTCGDIDECQN-PGICSQICINLKGGYKCECSRGYQMDLAT--G

VCKAVGK--EPSLIFTNRRDIRKIGLERKEYIQLVEQLRNTVALD---ADIAAQKLFWAD

LSQKAIFSASLDDKVGRHVKMIDN-VYNPAAIAVDWVYKTLYWTDAASKTISVATLDGTK

RKLLFNSDLREPASIAVDPLS-GFVYWSDWGEPAKIEKAGMNGFDRRPLVAVDIQWPNGI

TLDLIKNRLYWLDSKLHML-SSVDLNGQDRRIVLKSLEFLAHPLALTIFEDRVYWIDGEN

EAVYGANKFTGSELATLVNNLNDAQDIIVYHELVQPSGKNWCEEDMENGGCEYLCLPAPQ

INDHSPKYTCSCPNGFNLRANGRECQSTATTMTYSETKDTNTTEISPTSGLVPGGINVTT

AVSEVSVPPKGTSAAWAILPLLLLAMAAVGGYLMWRNWQHKNMKSMNFDNPVYLKTTEED

LSIDIGRHSASVGHTYPAISIVSTDDDLA

>S franciscanus

--------------------LLLSLLLSSHCITLILL------FYSGSAILCGEYQFRCQ

NGHCIPSYWQCDNYDDCGDNSDEQSCPKMYMYSLEYSYS---------YIIHCRIRIRMI

YYIPCPNPSL----RIATSHAETLTCSPSQFSCG-PGKNCIPLTWRCDRDV-DCPSGADE

HNCDALTCASNQFTCNNKKCVTTRWVCDGDNDCGDGSDEINCPSLTCAPDEFECTGTNET

TCIPNRWKCDGEIDCRDGSDESSTHCSTATTP---CSAGQFQCASGRCIHAAWKCDGERD

CGDGSDEESCCLVTPSPCAASEYRCDDGVCIPGDVECDGTQQCADGSDEANCNAPAECNP

ITHFRCSNSTCLPMVRVCNGIPECPNNEDEPGQEICGKNECLTNNGGCSHTCMDLAIGHV

CTCNAGYALVN----ETQCVDINECKTTLGICSQRCYNLPGSYKCDCDENYTLEHVNDRG

HCKANPEFGTPKLLIANRHDIRIFDLRTSSEDYIRRSLHSAVAVE---FDSASNTVFWTD

VGLEVVAKFGKQILDFFEMSYISD-VLLPDGVAVDWITRNLYWTDSGRDTIEVSRLDGSS

RTVLIDEDLDEPRAIALDPEG-G-MFWSDWGL-GVIERAGMNGQGREAIVTDEIYWPNGL

TLDYTLDMLYWIDAKESSI-SSVDFMGGNRRVVLAGQGRLPHPFGIAVFEDYIYWTDWES

KSIERANKFTGSNQTTILSGLSDAMDVIVVNPLRQAPSK---------------------

------------------------------------------------------------

------------------------------------------------------------

-----------------------------

>S purpuratus

--------------------------MGTYTLSLILLGLLACVFSAVNGNLCGENQFRCD

NNKCIPSVWKCDNEDDCGDNSDETVCGQLTCSSAEFECVGSGAGNGGSTCIPARWRCDG-

-DSDCPDGS-----DEIA--CQTLTCSPSQFSCG-PGKNCIPLTWRCDRDV-DCPSGADE

HNCDALTCASNQFTCNNKKCVTTRWVCDGDNDCGDGSDEINCPSLTCAPDEFECTGHNET

TCIPNRWKCDGDIDCRDGSDESSTHCSGPTVP---CSAGHFQCTSGECVHNSWKCDGERD

CFDGSDEDSCPLVTPSPCAANEYQCDNGVCIPGDVECDGTQQCADGSDEANCNAPAECNP

TTHFKCSNSTCLPMVRVCNGIPECPNNEDEPGQEICGRNECLTNNGGCSHTCLDLAIGHV

CTCNAGYTLVN----ETQCVDINECKTTLGICSQRCYNLPGSYKCDCDENYTLENVNDRG

HCKANPEFGAPKLLIANRHDIRIFDPSTSSEDYIKRSLHSAVAVE---FDSASNTVFWTD

VGLEVVSKTQLQAENEVVPVVVTDKVKTPDGIAVDWINKLLYWTDTGVDNIVVSNFEGTR

HTVLISSNLDEPRAIAVDPES-GLMFWSDWGL-GVIERAGMNGQGRTAIVTEGVSWPNGL

TLDYTLDMLYWIDAKESSI-SCVDFMGGNRRVVVAGQGRISHPFSIAVFEDYIYWTDWTK

ESIERADKFTGSNQTTILSGVSDAMDVIVVNPLRQAPYQ---------------------

------------------------------------------------------------

------------------------------------------------------------

-----------------------------

>L variegatus

--------------------SVIYIVYCPYRVIQSLM------FSFTLGNLCGGNQFTCA

NNKCIPSVWKCDNEDDCGDNSDELVCGKLEASIFILYIILPP------YVAQTKAHAKA-

---KCASPHYGYIHSLIIAHTESLTCAPNQFSCG-PGKNCIPLTWRCDHDV-DCQTGADE

HNCDALTCSANQFTCENKKCITTRWVCDGDNDCGDSSDEVNCPSLTCAPDQFECTGANET

TCIPGRWKCDGDIDCRDGSDESTTHCSDAAIP---CPQGHFQCNSGECVRLSWKCDGEQD

CFDGSDEESCAVITPTPCAPDEFQCANGVCIQGDVECNNVEECADGSDEANCNGPVDCNP

TTHYRCKNSTCLPWVRVCNGIPECPDNEDEPGQEICGEQRI--------HYIYQIIYAKP

YHSKSKYIWSKYHNIQPEVIIIQVPKSIASYYHRPCVKI-------------IHYPKQLI

YCYYHAVRAIPQCQIKMYVSNRISHINQVVCFYCFQYLFEAIFIDCLVFESCIRCL--NA

SGCYLSNSSQLVAENGVEPVMVTNKVKTPDGIAVDWITKNLYWTDAGYDRIEVSNLDGTS

RTVLISSDLDEPRAIAVDPES-GLMFWSDWGL-GVIERAGMNGQHRTAIVTEKIQWPNAL

TVDYTLDRIYWIDAKESSI-SSVSWMGTNRRVILSGHGRLPHPFAIAVFEDYIYWTDWHT

KSIYRANKFTGQDQTIIQGGLHDPMDIHVFHPQRQVPGK---------------------

------------------------------------------------------------

------------------------------------------------------------

-----------------------------

>M_musculus

----------MGTSARWALWLLLALCWAPRDSGAT---------ASGKKAKCDSSQFQCT

NGRCITLLWKCDGDEDCADGSDEKNCVKKTCAESDFVCKN-G------QCVPNRWQCDG-

-DPDCEDGS-----DESPEQCHMRTCRINEISCGARSTQCIPVSWRCDGEN-DCDNGEDE

ENCGNITCSADEFTCSSGRCVSRNFVCNGQDDCDDGSDELDCAPPTCGAHEFQCS---TS

SCIPLSWVCDDDADCSDQSDESLEQCGRQPVIHTKCPTSEIQCGSGECIHKKWRCDGDPD

CKDGSDEVNCPSRT---CRPDQFECEDGSCIHGSRQCNGIRDCVDGSDEVNCKNVNQCLG

PGKFKCRSGECIDMSKVCDQEQDCRDWSDEPLKE-CHINECLVNNGGCSHICKDLVIGYE

CDCAAGFELID----RKTCGDIDECQN-PGICSQICINLKGGYKCECSRGYQMDLAT--G

VCKAVGK--EPSLIFTNRRDIRKIGLERKEYIQLVEQLRNTVALD---ADIAAQKLFWAD

LSQKAIFSASIDDKVGRHFKMIDN-VYNPAAIAVDWVYKTIYWTDAASKTISVATLDGAK

RKFLFNSDLREPASIAVDPLS-GFVYWSDWGEPAKIEKAGMNGFDRRPLVTEDIQWPNGI

TLDLVKSRLYWLDSKLHML-SSVDLNGQDRRIVLKSLEFLAHPLALTIFEDRVYWIDGEN

EAVYGANKFTGSELATLVNNLNDAQDIIVYHELVQPSGKNWCEDDMENGGCEYLCLPAPQ

INDHSPKYTCSCPNGYNLEENGRECQSTSTPVTYSETKDINTTDILRTSGLVPGGINVTT

AVSEVSVPPKGTSAAWAILPLLLLVMAAVGGYLMWRNWQHKNMKSMNFDNPVYLKTTEED

LSIDIGRHSASVGHTYPAISVVSTDDDLA

YWHAG

>H sapiens

------MVDREQLVQKARLAEQAERYDDMAAAMKNVTELNEPLSNEERNLLSVAYKNVVG

ARRSSWRVISSIEQKTSADGNE---------KKIEMVRAYREK-IEKELEAVCQDVLSLL

--DNYLIKNCSE----TQY---------------ESKVFYLKMKGDYYRY--LAEVATGE

K---RATVVESSEKAYSEAHEISKEHMQPTHPIRLGLALNYSVFYYEIQNAPEQACHLAK

TAFDDAIAELDTLNEDSYKDSTLIMQLLRDNLTLWTSDQQDDDGGEGNN--

>H glaber

------MVDREQLVQKARLAEQAERYDDMAAAMKNVTELNEPLSNEERNLLSVAYKNVVG

ARRSSWRVISSIEQKTSADGNE---------KKIEMVRAYREK-IEKELEAVCQDVLSLL

--DNYLIKNCSE----TQY---------------ESKVFYLKMKGDYYRY--LAEVATGE

K---RATVVESSEKAYSEAHEISKEHMQPTHPIRLGLALNYSVFYYEIQNAPEQACHLAK

TAFDDAIAELDTLNEDSYKDSTLIMQLLRDNLTLWTSDQQDDEGGEGNN--

>M brandtii

------MVDREQLVQKARLAEQAERYDDMAAAMKNVTELNEPLSNEERNLLSVAYKNVVG

ARRSSWRVISSIEQKTSADGNE---------KKIEMVRAYREK-IEKELEAVCQDVLSLL

--DNYLIKNCSE----TQY---------------ESKVFYLKMKGDYYRY--LAEVATGE

K---RATVVESSEKAYSEAHEISKEHMQPTHPIRLGLALNYSVFYYEIQNAPEQACHLAK
[truncated: 1,316 more chars]
